# β‑Selective Addition of Pyrroles to Electron-Deficient Alkenes in Both Catalytic and Stoichiometric Modes on B(C_6_F_5_)_3_

**DOI:** 10.1021/acsomega.5c00371

**Published:** 2025-05-19

**Authors:** Seina Sekine, Miho Kashiwa, Maho Kawakami, Takumi Sonoda, Arisa Ono, Teruhisa Tsuchimoto

**Affiliations:** Department of Applied Chemistry, School of Science and Technology, Meiji University, 1-1-1 Higashimita, Tama-ku, Kawasaki 214-8571, Japan

## Abstract

To the best of our knowledge, the addition of pyrroles to electron-deficient alkenes (APEda) via electrophilic aromatic substitution (S_E_Ar) has been reported to occur exclusively at the α-position of the pyrrole without any formation, even in trace amounts, of a β-adduct, β-**3**. In sharp contrast to the prolonged immutable observation, we established an original S_E_Ar-based APEda (S_E_Ar-APEda) system applicable to both the catalytic and stoichiometric synthesis of β-**3**.

## Graphical abstract

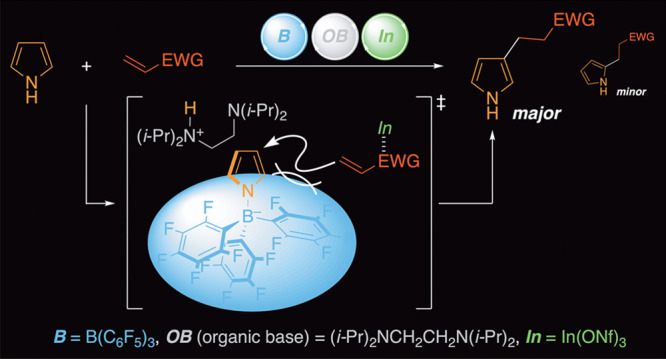

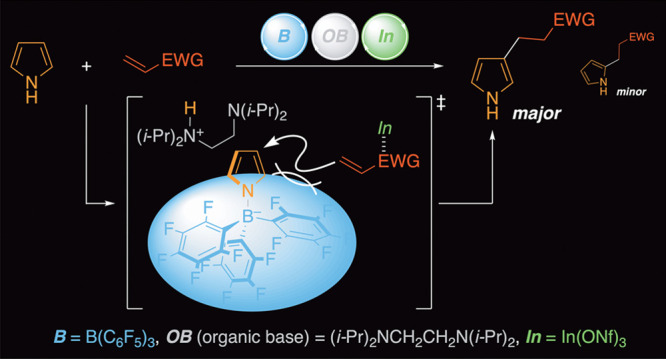

## Introduction

β-Alkylpyrroles, wherein a β-pyrrolyl group is tethered by a two-carbon chain to an electron-withdrawing group (EWG), are crucial units of biofunctional^1^ and bioactive^2^ molecules, as well as optoelectronic materials (Figure 1a).^3^ This structural motif is hence an attractive synthetic target. Retrosynthetic analysis suggests that the S_E_Ar-APEda should be a straightforward route for obtaining β-alkylpyrroles β-**3** in a single step (Figure 1b). However, due to the inherent α-orientation of pyrroles **1** in the S_E_Ar process, access of electrophiles to the β-position of **1** cannot be a major route.^4^ In fact, to the best of our knowledge, an exclusive α-addition has been observed in the preceding S_E_Ar-APEda, and even contamination by β-**3** has thus yet to be observed.^5^ Importantly, to access β-**3** from **1** by a traditional strategy, multiple steps have been necessary (Figure 1c).^2,6^ Despite the strict α-orientation of **1** in the S_E_Ar-APEda, two strategies for the β-addition controlled electronically and sterically by a preinstalled substituent onto the pyrrole ring have been reported, albeit as only two specific reactions: One uses the pyrrole with the acetyl (Ac) group at the α-position (Scheme 1a),^7^ which can be regarded as a so-called *meta*-directing EWG in a S_E_Ar reaction.^8^ The other uses the pyrrole with the bulky *N*-Si­(*i*-Pr)_3_ (*Si*) group (Scheme 1b).^9^ The substrate scope of these strategies is unclear. The precedents in references 4–9 show that directing-group-free β-selective S_E_Ar-APEda of **1** is undoubtedly challenging.

**Figure 1:**
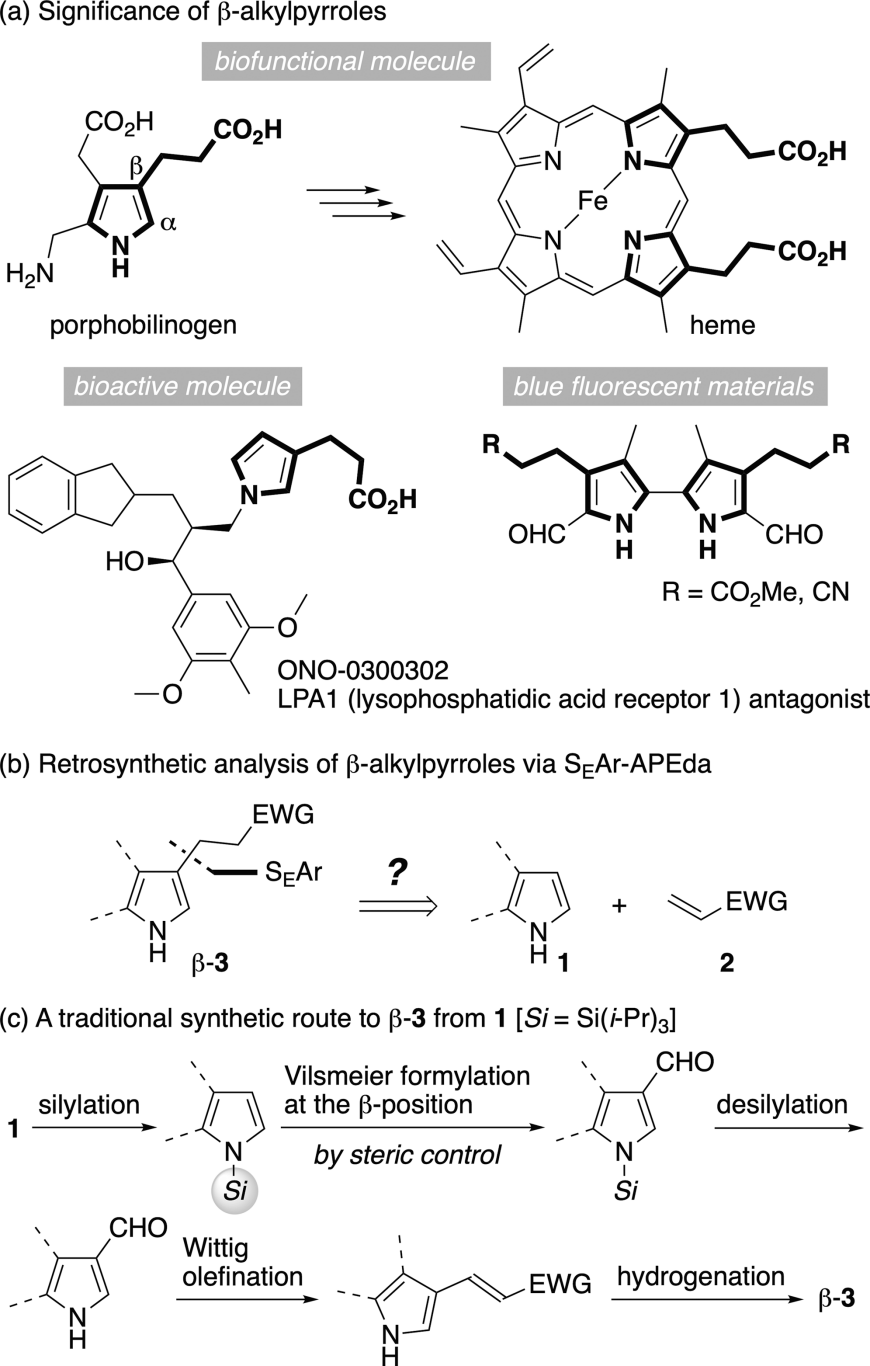
β-Alkylpyrroles: (a) significance and (b) retrosynthetic analysis.

**Scheme 1:**
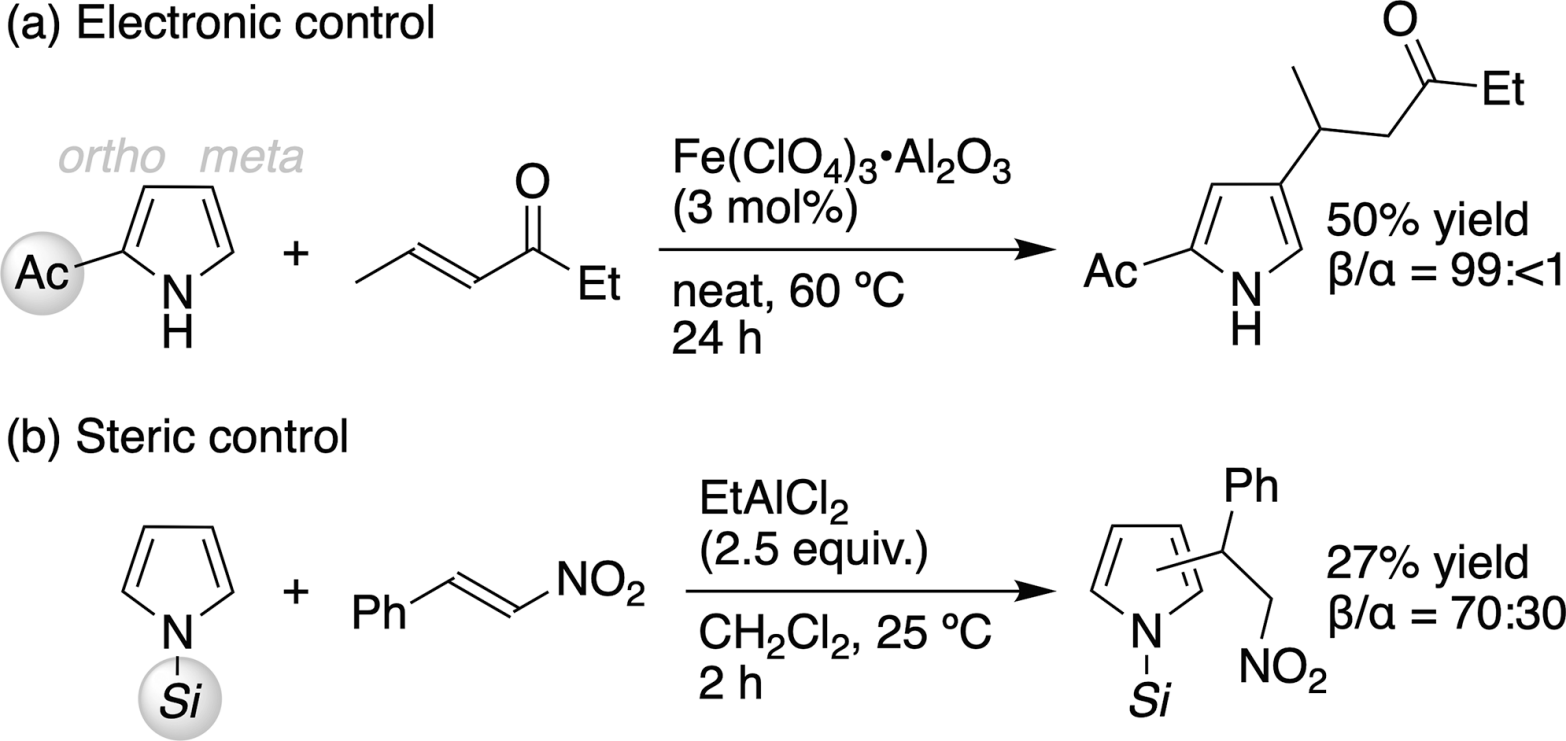
Precedents on β-Selective S_E_Ar-APEda Using Substituted Pyrroles for Electronic and Steric Control

On the other hand, Resconi, Focante and colleagues have reported that treating pyrrole (**1a**) with B­(C_6_F_5_)_3_ and NEt_3_ at room temperature (rt) yielded (*N*-pyrrolyl)­borate complex **4a** (Scheme 2a).^10^ **4a** was attractive to us in that its C_6_F_5_ units could efficiently cover its α-position from the attack of external electrophiles such as CH_2_=CH–EWG **2** (Scheme 2b). We envisaged that **4**, formed catalytically in situ, could lead to the β-selective S_E_Ar-APEda and that this will be the first example of the direct synthesis of β-**3** from a simple pyrrole like **1a** via the S_E_Ar-APEda. Besides the catalytic reaction, we examined how **4** behaves as a stoichiometric reagent. Herein, we report the β-selective S_E_Ar-APEda in both catalytic and stoichiometric modes on B­(C_6_F_5_)_3_. **NOTE**: During this research, we found that B­(C_6_F_5_)_3_ stored in a sample bottle forms a complex with water. Therefore, the chemical formula is represented as H_2_O**·**B­(C_6_F_5_)_3_ hereafter.^11^

**Scheme 2:**
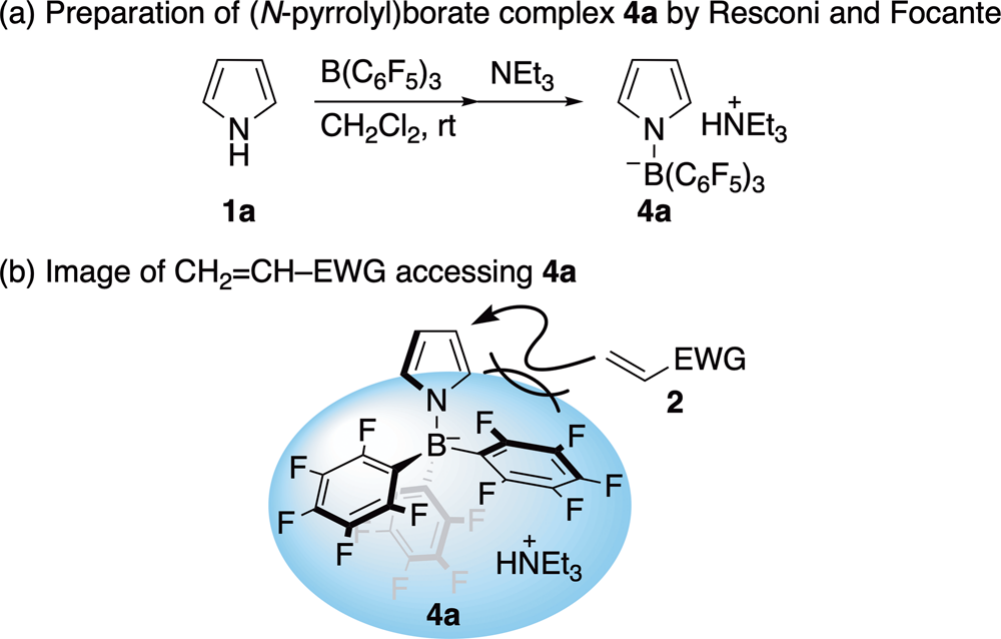
(*N*-Pyrrolyl)­borate Complex **4a**: (a) Preparation and (b) Image of 4a + CH_2_=CH–EWG

## Results and Discussion

This study commenced with investigating the reaction of **1a** (3 equiv) with butyl acrylate (**2a**). After careful examination based on our insights gained through the development of a series of Lewis-acid-catalyzed reactions of heteroarenes,^12^ we found the following important aspects and reaction conditions: Boron, organic base (OB), and indium compounds were all required as catalysts to obtain desired product β-**3a**; if even one was absent, the reaction failed to proceed or yielded only α-**3a**. As a result, we developed the reaction conditions summarized in Scheme 3 ^13^ [see the Supporting Information (SI) for detailed experimental results with different reaction conditions]. Thus, the reaction of **1a** (5 equiv) with **2a** in THF at 70 °C for 24 h in the presence of H_2_O**·**B­(C_6_F_5_)_3_ (19 mol %),^14,15^ T*i*PEDA (25 mol %), and In­(ONf)_3_ (20 mol %) delivered **3a** in a good yield of 74% with 77% β-selectivity. Notably, this is the first achievement in which a simple pyrrole without a preinstalled β-directing group undergoes the S_E_Ar-APEda reaction to yield a β-adduct and, furthermore, to yield the β-adduct preferentially over the α-adduct.

**Scheme 3:**
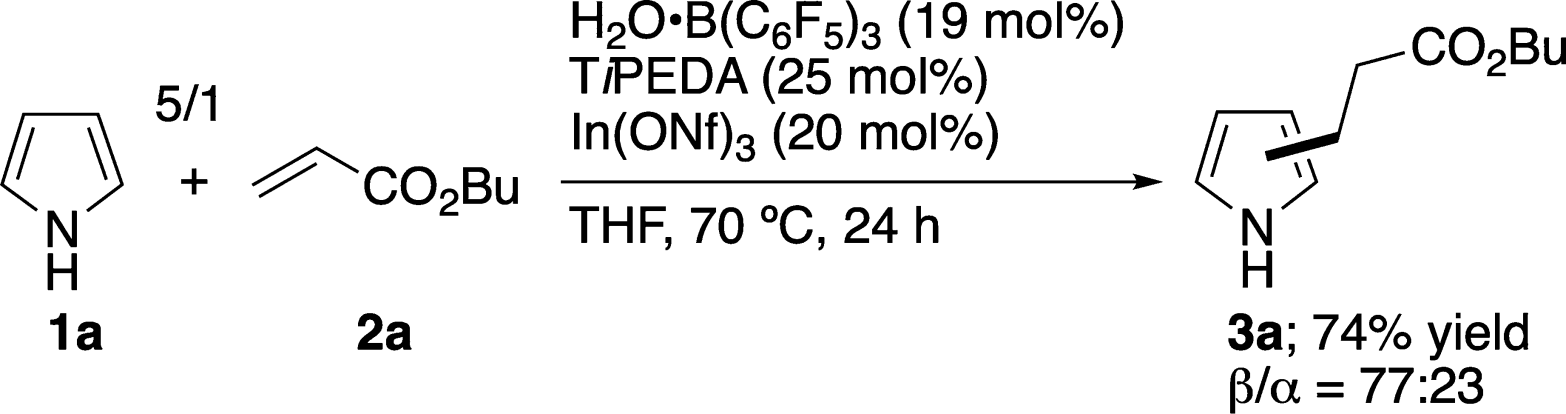
Reaction Conditions for Catalytic Reaction^a^

a Reagents: **1a** (2.50 mmol), **2a** (0.500 mmol), H_2_O**·**B­(C_6_F_5_)_3_ (96.6 μmol), T*i*PEDA (125 μmol), In­(ONf)_3_ (100 μmol), and THF (0.800 mL). Abbreviations: T*i*PEDA = (*i-*Pr)_2_N­(CH_2_)_2_N­(*i-*Pr)_2_; Nf = SO_2_C_4_F_9_. The yield and β/α ratio of **3a** were determined by GC.

We investigated the scope of the catalytic reaction on H_2_O**·**B­(C_6_F_5_)_3_ using the reaction conditions in Scheme 3. The results are summarized in Table 1, where the results of both before and after the purification of **3** are presented, and regioisomers of α-**3** and β-**3** were successfully separated and characterized in all reactions. Similarly to that of **2a**, the reactions of alkyl acrylates **2b**–**2e** proceeded with β-selectivities of over 70%, indicating that the length and size of R^3^ does not significantly affect the β-selectivity (**3a**–**3e**). The yield and selectivity before and after the purification process varied depending on the difficulty in isolating β-**3** from a crude product including α-**3** and a regioisomeric mixture of 1/2 adducts of **1** and **2**. Cyclic ester **2f** also led to β-preferential addition (**3f**). Besides esters **2a**–**2f**, acrylonitrile (**2g**) and *N*-phenylmaleimide (**2h**) afforded the corresponding β-adducts **3g** and **3h** in moderate yields. Other than the reaction of **2h**, 1/2 adducts of **1** and **2** were formed as major byproducts, directly affecting the yields of β-**3**. Unlike the findings of Table 1, usage of 3-methylpyrrole (**1b**), *N*-acryloylmorpholine (**2j**), (*E*)-chalcone (**2k**), and 2-cyclohexen-1-one (**2l**) as the starting substrates yielded poor results (Figure 2): The reason is stated below each substrate.

**Table 1:**
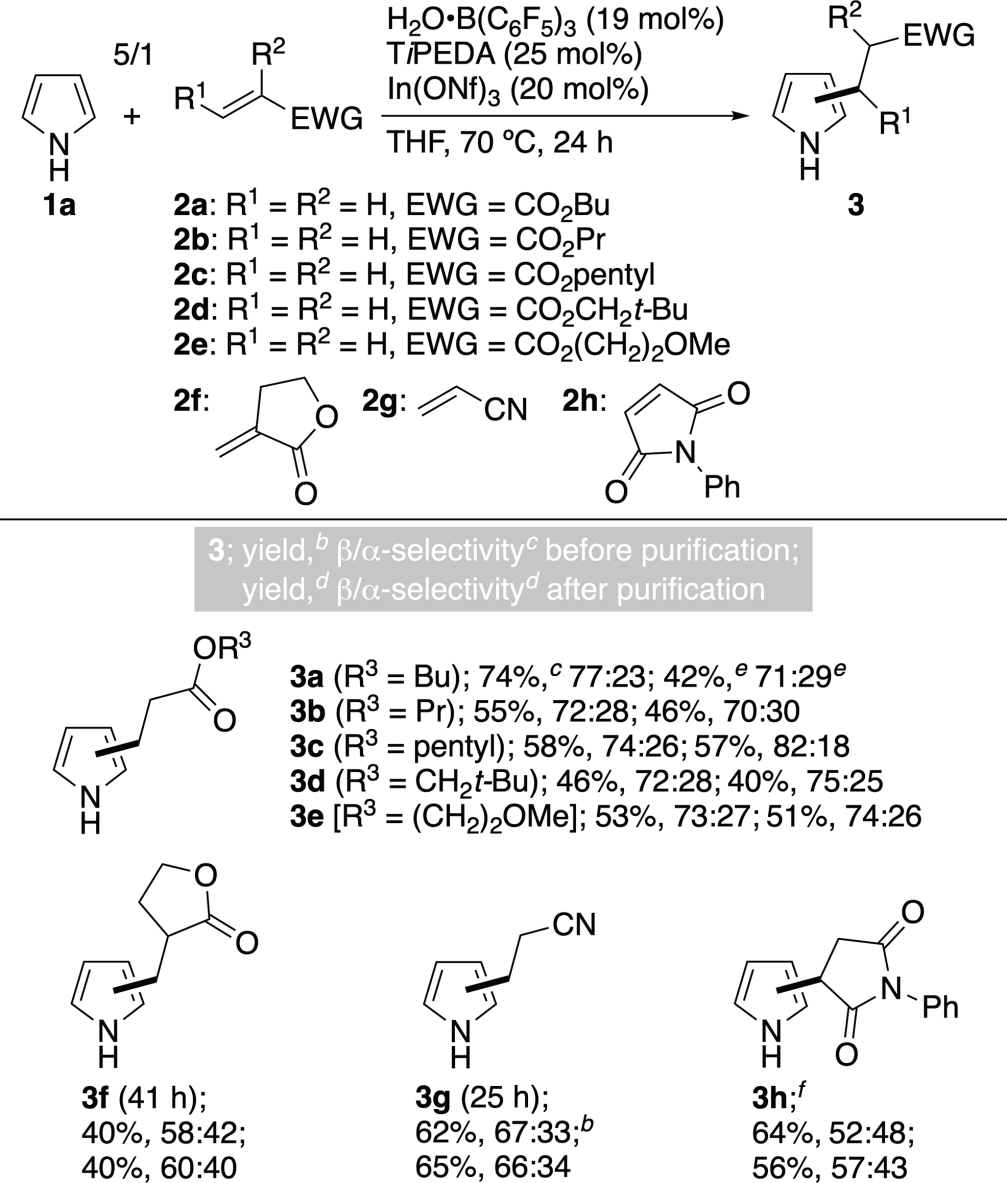
Substrate Scope of Catalytic Reaction Favoring β-Alkylpyrroles^a^

a Reagents: **1a** (2.50 mmol), **2a** (0.500 mmol), H_2_O**·**B­(C_6_F_5_)_3_ (96.6 μmol), T*i*PEDA (125 μmol), In­(ONf)_3_ (100 μmol), and THF (0.800 mL).

b Determined by NMR.

c Determined by GC.

d Determined based on the weight of isolated β-**3** and α-**3**.

e Average of two experiments.

f Performed with **1a** (0.500 mmol) in THF (1.60 mL) at 80 °C.

**Figure 2:**
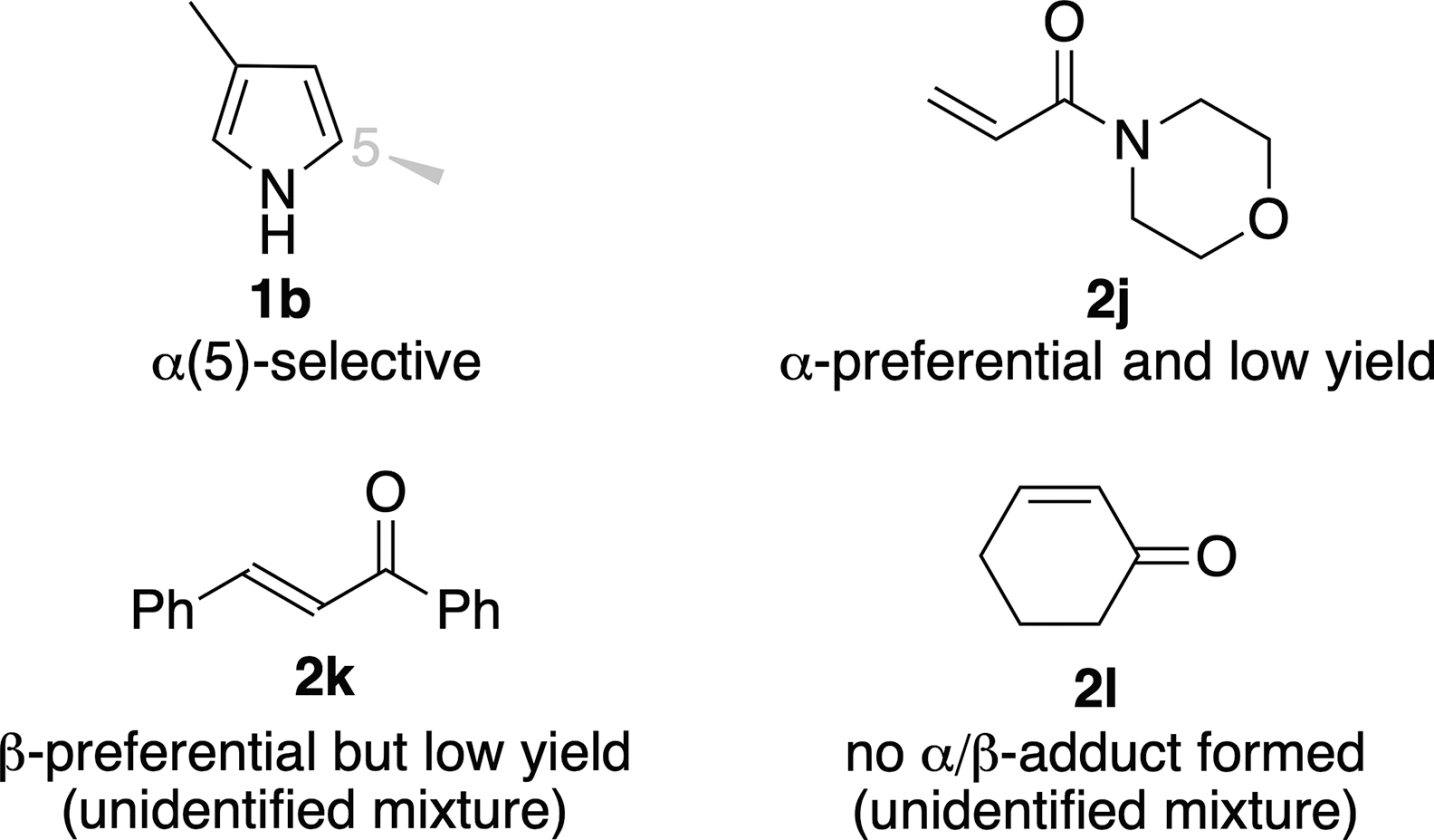
Unsuccessful substrates in catalytic reaction.

Scaling up the catalytic mode was feasible, albeit with somewhat reduced yield and β-selectivity of **3**. For example, a 5.00 mmol scale reaction of **2g** with **1a** (25.0 mmol) delivered **3g** in 58% yield (348 mg) with 57% β-selectivity.

Historically, the typical S_E_Ar-APEda reaction has exclusively yielded α-adducts, as already stated. Hence, it is necessary to uncover why β-**3** is formed in the current ternary system consisting of H_2_O**·**B­(C_6_F_5_)_3_, an organic base (e.g., T*i*PEDA), and In­(ONf)_3_. To confirm whether, as initially expected, (*N*-pyrrolyl)­borate complex **4** is an active species for delivering β-**3**, we aimed to construct **4** in preparation for a control experiment with **2**. We first attempted to prepare the known borate complex **4a** from **1a**, H_2_O**·**B­(C_6_F_5_)_3_, and NEt_3_, following a reported procedure.^16^ However, the reaction failed, mainly yielding [(F_5_C_6_)_3_BOH]^−^HN^+^Et_3_ (**5a**) (Scheme 4a).^17^ We considered that **4a** would not be obtained unless H_2_O coordinating to B­(C_6_F_5_)_3_ is removed. Allyltrimethylsilane is reported to be a reagent for removing the H_2_O, as shown in Scheme 4b.^18^ We therefore attempted to prepare **4a** by conducting the first step in the presence of allyltrimethylsilane. As expected, **4a** was isolated in 86% yield after recrystallization from hexane/CH_2_Cl_2_ (Scheme 4c). The modified recipe was reproducible and reliably applied to the synthesis of T*i*PEDA-based (*N*-pyrrolyl)­borate complex **4b**. The molecular structure of **4b** was confirmed by single-crystal X-ray diffraction analysis.

**Scheme 4:**
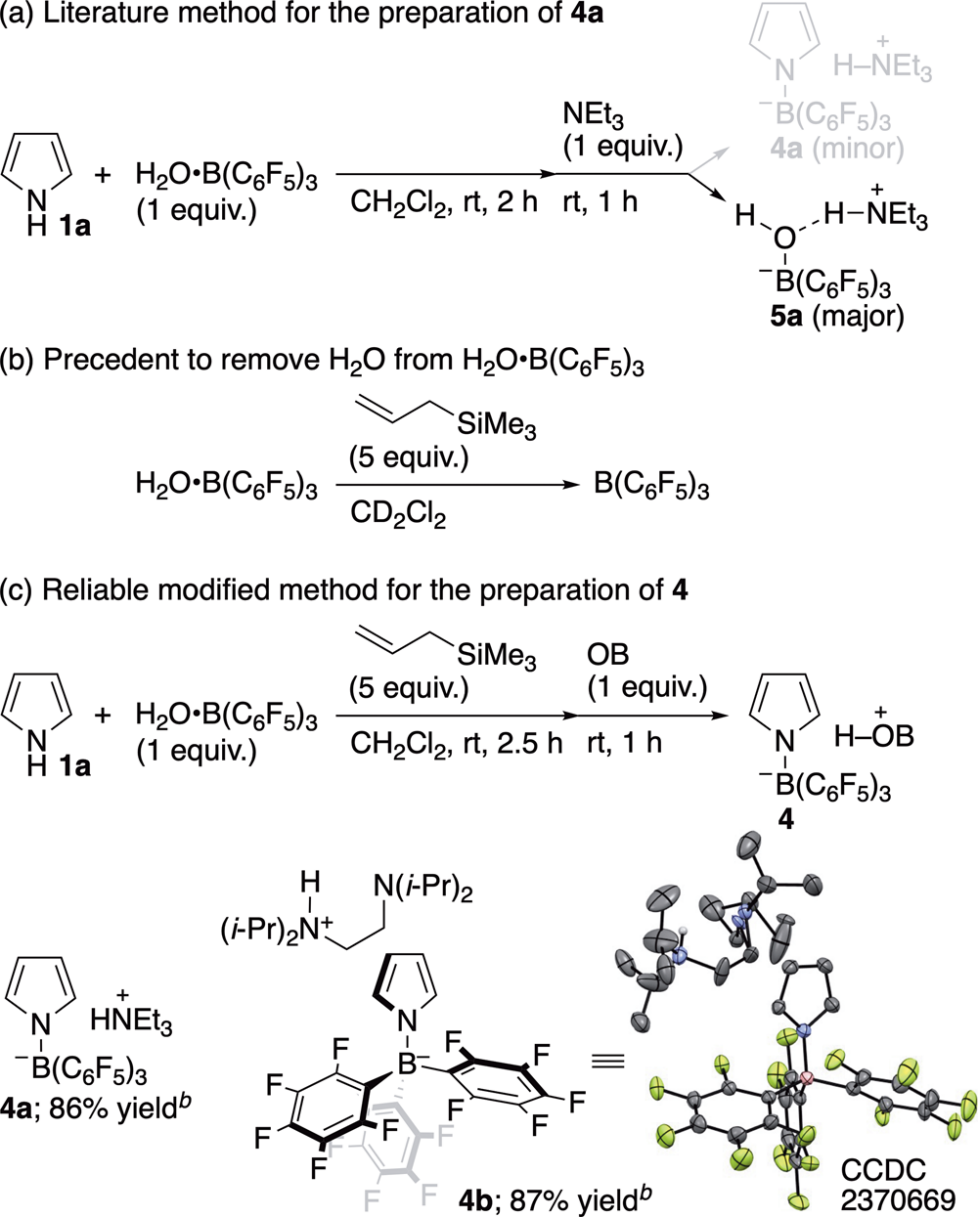
Preparation of (*N*-Pyrrolyl)­borate Complexes^a^

a rt = 22–24 °C.
b Isolated yield based on **1a**. Abbreviations: OB = organic base.

Having successfully obtained **4b**, a stoichiometric control experiment was performed with **2a**, along with catalyst In­(ONf)_3_ (Scheme 5). Compared to the catalytic reaction on H_2_O**·**B­(C_6_F_5_)_3_ (Scheme 3), this reaction proceeded smoothly with a lower loading of In­(ONf)_3_ (10 mol %) and at a much lower reaction temperature of 30 °C. Moreover, only β-**3a** was produced as a single regioisomer.^19^ By contrast, the indium-catalyzed S_E_Ar-APEda of **1a** instead of **4b** gave only α-**3a** as a single regioisomer (Scheme 5). These results are entirely opposite, clearly suggesting that (*N*-pyrrolyl)­borate complex **4** is a crucial active species in the formation of β-**3**.

**Scheme 5:**
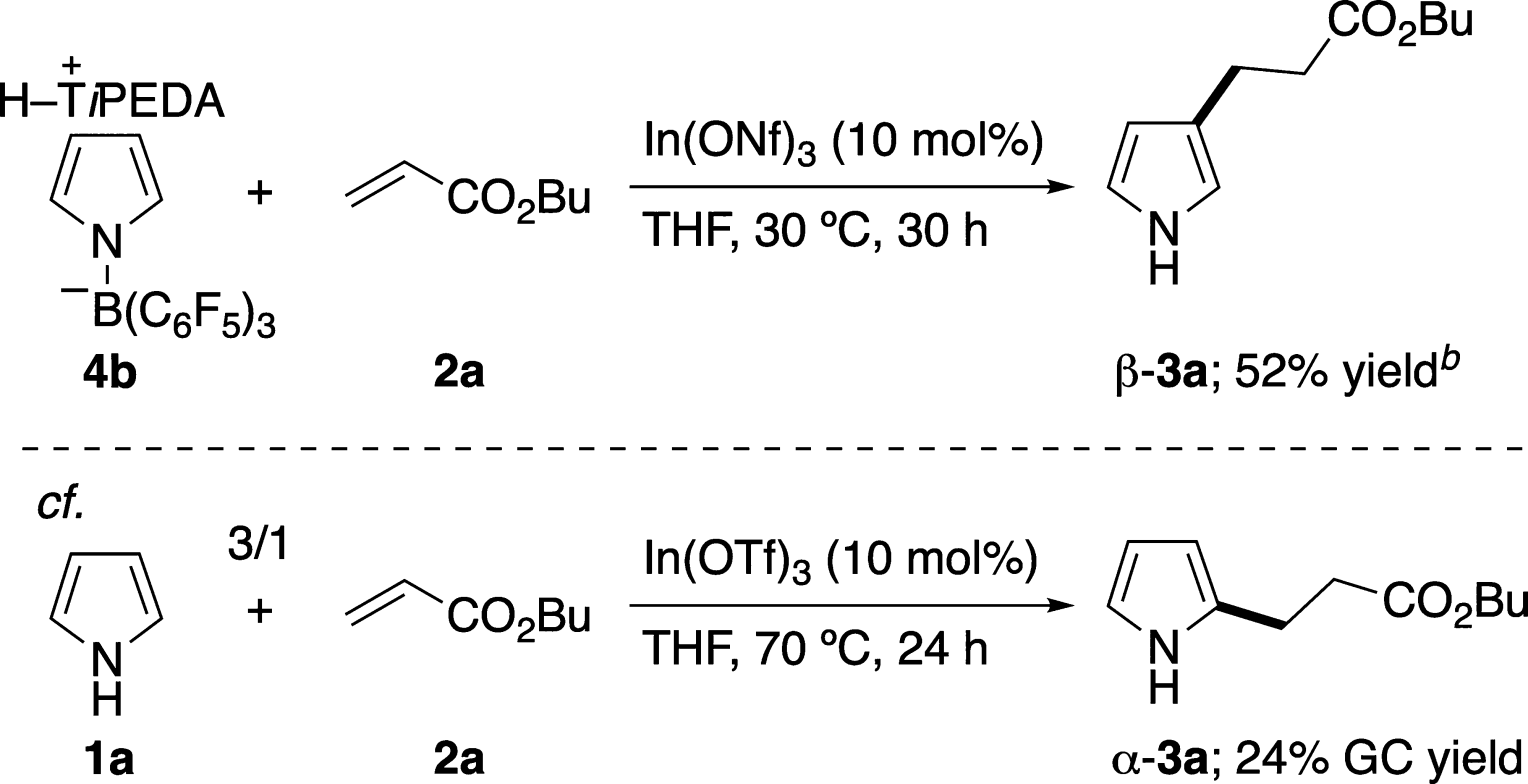
Stoichiometric Control Experiment of T*i*PEDA-Based (*N*-Pyrrolyl)­borate Complex with CH_2_=CHCO_2_Bu under Indium Catalysis^a^

a Reagents: **4b** (0.150 mmol), **2a** (0.100 mmol), In­(ONf)_3_ (10.0 μmol), and THF (1.20 mL).
b Determined by NMR.

As already presented in Scheme 1b, sterically controlled S_E_Ar-APEda has been reported by utilizing the *N*-Si­(*i*-Pr)_3_ group as a bulky β-directing group.^9^ However, its β-selectivity is insufficient, promoter EtAlCl_2_ is necessary in more than a stoichiometric amount, and its scope remains unclear. In contrast to this precedent, the reaction with stoichiometric boron but catalytic indium in Scheme 5 occurred with complete β-selectivity. Inspired by this result as the first example of the sterically controlled β-exclusive S_E_Ar-APEda, we explored the scope of the stoichiometric reaction (Table 2). First, *N*-phenylmaleimide (**2h**) added exclusively at the β-position of **4b**, giving β-**3h** in a much higher yield than that of the corresponding catalytic reaction. *N*-Methylmaleimide (**2i**) similarly furnished only β-**3i**. However, using noncyclic amide **2j** resulted in a low yield of β-**3j**, which could not be isolated in pure form (see the SI for details). In contrast to their incompatibility with the catalytic mode (Figure 2), (*E*)-chalcone (**2k**) and 2-cyclohexen-1-one (**2l**) worked well, regioselectively giving β-**3k** and β-**3l**, respectively. Since keto groups easily react with pyrroles under indium catalysis,^20^ the compatibility of the ketone substrates is noteworthy. *trans*-β-Nitrostyrene (**2m**) also gave β-**3m** exclusively in 87% yield. Unlike the other Michael acceptors listed in Table 2, the use of acrylonitrile (**2g**) led to **3g** with a similar β/α ratio as that in the catalytic reaction, albeit in a much higher yield. The stoichiometric reaction is also applicable to other (*N*-pyrrolyl)­borate complexes **4c** and **4d**, derived from 3-methyl- and 2-phenylpyrroles, respectively. Remarkably, even **4c**,^21^ in which the β-site of the pyrrole ring is premethylated, reacted with **2i** at the sterically congested β-position to give β-**3n**. Exclusive β-addition was also observed when using **4d**, giving a mixture of 2,3- and 2,4-disubstituted pyrroles **3o** in high yields.

**Table 2:**
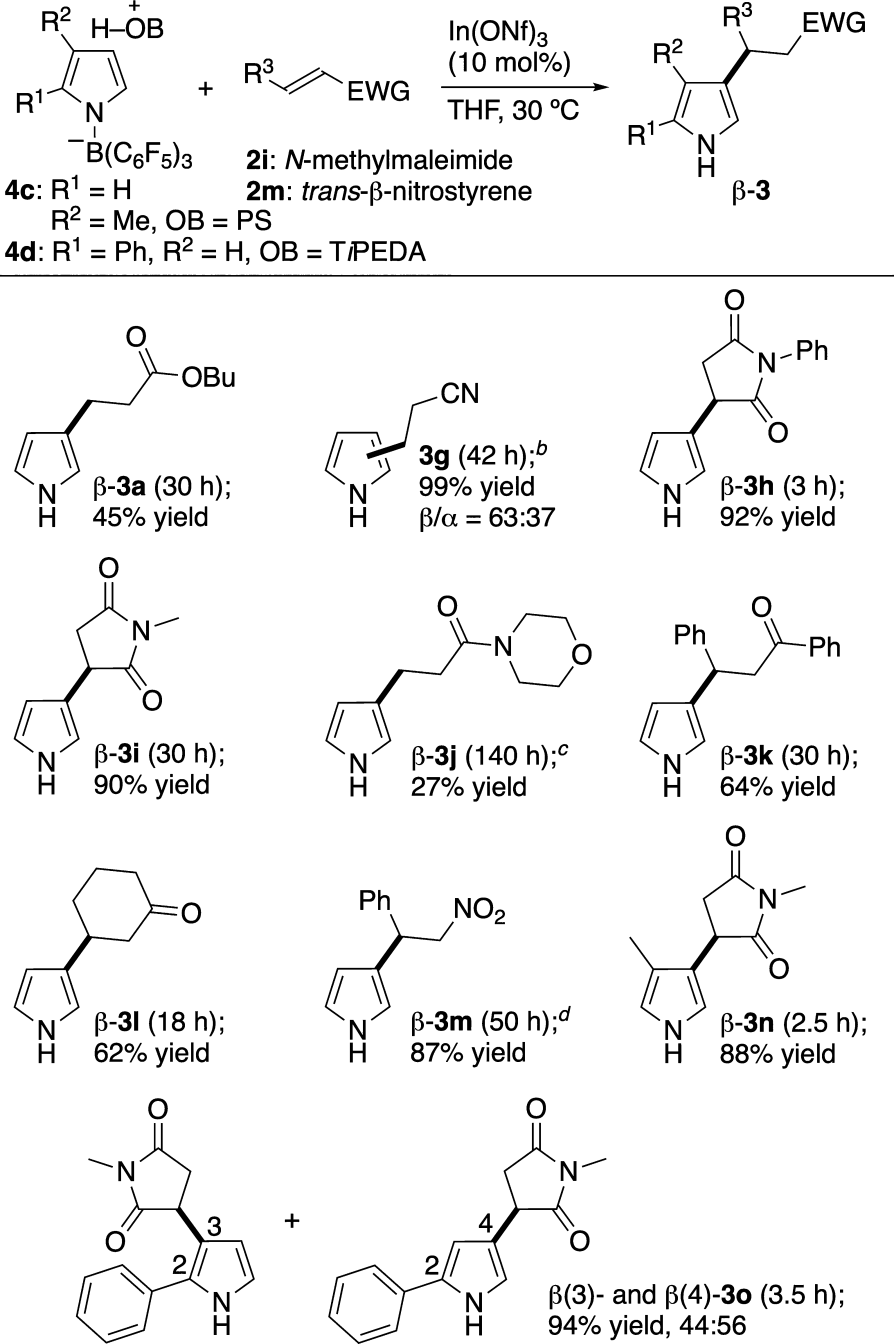
Substrate Scope of Stoichiometric Reaction^a^

a Numbering of substrates **4c, 4d**, **2i**, and **2m** (yet to be assigned) is presented here. Reactions were performed on a 0.100 or 0.200 mmol scale of **2**. Reagents on a 0.100 mmol scale: **4** (0.150 mmol), **2** (0.100 mmol), In­(ONf)_3_ (10.0 μmol), and THF (1.20 mL). Yields of isolated **3** based on **2** are shown here. Abbreviations: PS = proton-sponge [1,8-bis­(dimethylamino)­naphthalene].

b Performed at 22 °C. The yield and selectivity were determined based on the weight of isolated β-**3g** and α-**3g**.

c Performed on a 0.200 mmol scale using In­(ONf)_3_ (20.0 μmol) in THF (0.800 mL) at 40 °C, and the yield determined by NMR is stated.

d Performed on a 0.100 mmol scale in THF (0.600 mL) at 10 °C.

In a S_E_Ar alkylation, a large excess of (hetero)­arenes to alkyl electrophiles is generally used to ensure a high yield of the monoalkylation product, as the reaction otherwise results in multialkylation.^5f,5g^ Despite this inherent issue, a large excess of **4** to **2** is unnecessary in the stoichiometric reaction, being likely attributed to the good stability of a β-**3**-based (*N*-pyrrolyl)­borate complex under the reaction conditions (see the SI for details). This is because multialkylation of the complex seems unfavorable, likely due to the steric congestion around the complex, as shown in Figure 3. In fact, no multialkylation products were observed in the stoichiometric reactions of Table 2, except when using **2a** (see the SI for further details).

**Figure 3:**
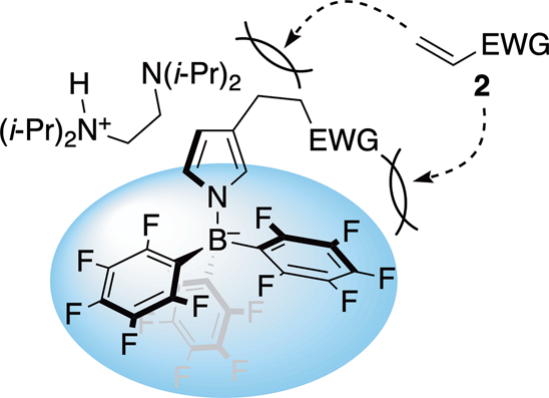
Image of restricted multialkylation in stoichiometric reaction.

The present catalytic S_E_Ar-APEda reaction is the first example of a one-step synthesis of β-**3** from pyrrole. Among the products obtained in Table 1, only the synthetic method for β-**3a** has been disclosed in patents, albeit in three steps.^22,23^ β-**3f** and β-**3g** are known compounds, but there are no reports on their preparation. Meanwhile, β-**3b**–β-**3e** and β-**3h** are thus far unknown. Accordingly, the accessibility of β-**3** in our strategy encouraged us to synthesize other compounds that can be obtained only from β-**3**. For instance, carboxylic acid **6**, alcohol **7**, and pyrrolidine **8** ^24^ were obtained from β-**3a** via simple transformations (Scheme 6).

**Scheme 6:**
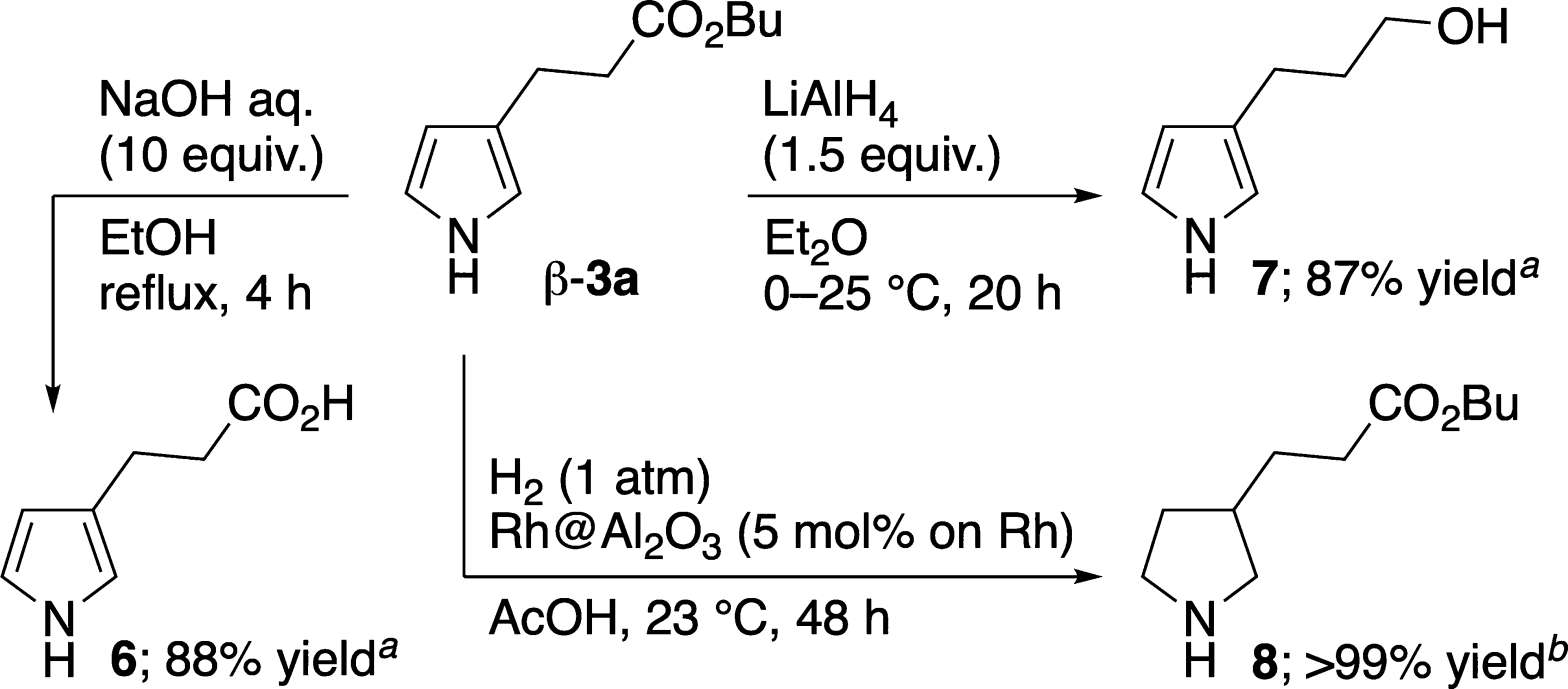
Transformation of β-**3a**

a Isolated yield.
b NMR yield.

To gain insight into the mechanism of the catalytic reaction on H_2_O**·**B­(C_6_F_5_)_3_, several experiments were conducted. First, ^11^B NMR experiments were performed to verify whether **4b** could be formed in situ during the catalytic reaction (Scheme 7). Upon treating **1a** with catalytic amounts of H_2_O**·**B­(C_6_F_5_)_3_, T*i*PEDA, and In­(ONf)_3_ in THF, **4b** was the main boron-containing product (79%) even at a low reaction temperature of 22 °C (Scheme 7a); The ^11^B NMR spectrum of **4b** in THF is given in Scheme 7b. **4b** was also present in the crude reaction mixture after the catalytic reaction (Scheme 7c). Importantly, In­(ONf)_3_ was indispensable for yielding **4b** (Scheme 7a vs 7d). The ^11^B NMR peak observed at – 3.9 ppm in Scheme 7d is likely ascribed to [(F_5_C_6_)_3_BOH]^−^(HT*i*PEDA)^+^ (**5b**) in which no **1a** is contained (Scheme 7d vs 7e). These results confirm that **4b** is formed in situ during the catalytic reaction.

**Scheme 7:**
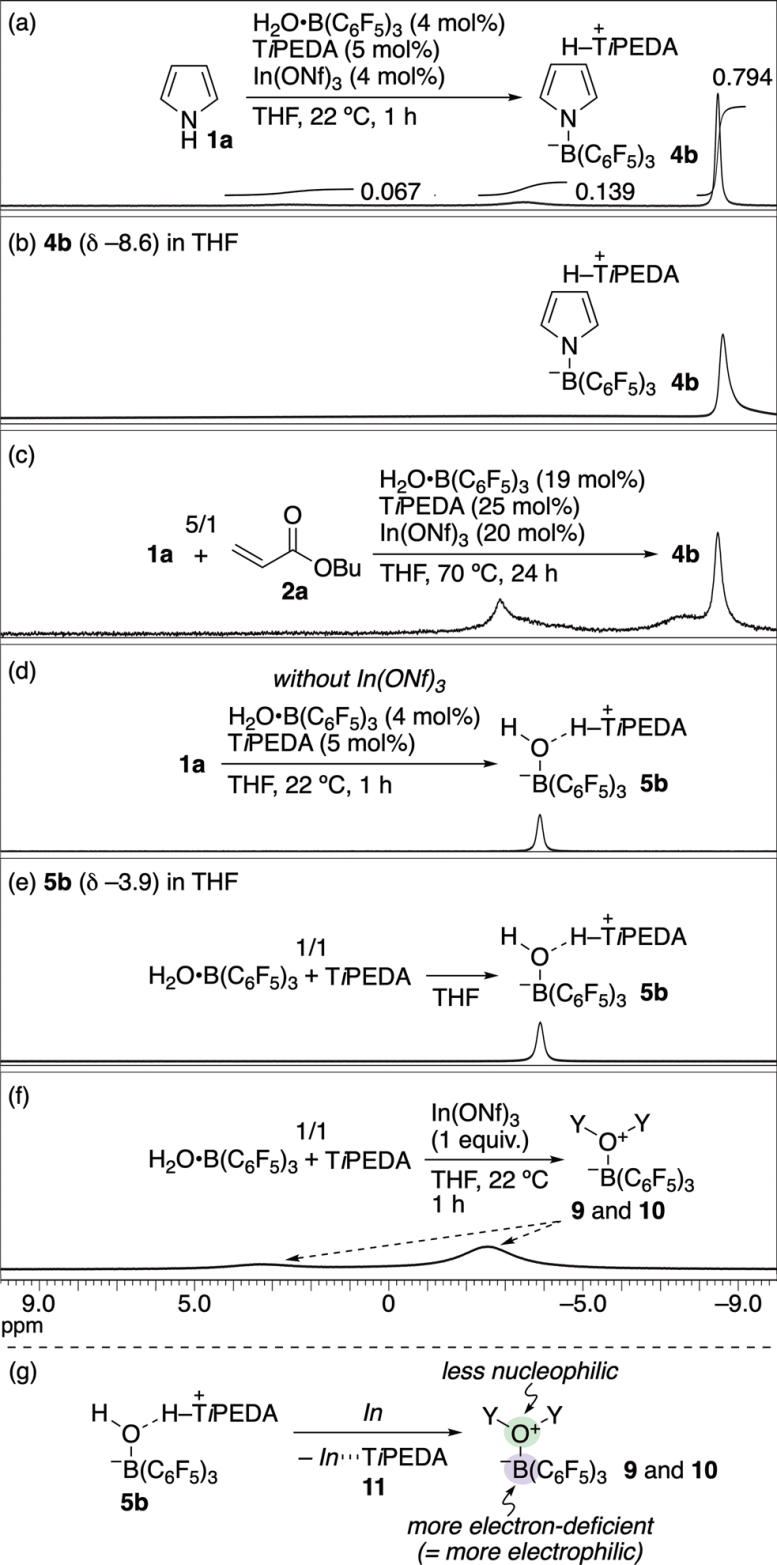
^11^B NMR Analysis for Validation of Formation of (*N*-Pyrrolyl)­borate Complex **4b** in Catalytic Reaction

Based on the results in Scheme 7d, the role of In­(ONf)_3_ in the formation of **4b** was examined. Adding In­(ONf)_3_ to the THF solution of **5b** caused the appearance of the two downfield-shifted broad signals at 3.6 and – 2.6 ppm (Scheme 7f). While the structural assignments of these signals are unclear,^25^ this result reveals that, compared to **5b** coordinated by a nucleophilicity-enhanced oxygen atom due to deprotonation by T*i*PEDA, more electron-deficient B­(*sp* ^3^)-hybridized species **9** and **10** coordinated by less nucleophilic oxygen atoms were formed.^26^ It was assumed that this spectral change might be due to the liberation of T*i*PEDA from **5b** owing to the coordination of T*i*PEDA to In­(ONf)_3_ (*In*) to give **11**, as proposed in Scheme 7g.

To verify the above hypothesis, we continuously monitored the behavior of T*i*PEDA in THF-*d*_8_ by ^1^H NMR spectroscopy (Scheme 8). The ^1^H NMR spectrum of **5b** is shown in Scheme 8b, where all proton signals of H^a^, H^b^ and H^c^ were shifted downfield, compared to the original H^a–c^ signals of T*i*PEDA (Scheme 8a vs 8b). Adding *In* to the solution of **5b** gave one T*i*PEDA-based species and led to a further downfield shift in the H^a–c^ signals (Scheme 8c). This result reveals the generation of distinct species (○) that should be formed by the migration of T*i*PEDA from mild acid **5b** to stronger acid *In* and is well consistent with the result of Scheme 7f as well as the assumption of Scheme 7g. A control experiment by mixing *In* and T*i*PEDA also yielded the same species (○), proposing that the structure is the complex of *In* and T*i*PEDA: *In*···T*i*PEDA (**11**), while giving another *In*···T*i*PEDA species **12** (△) (Scheme 8d). Therefore, all the NMR studies support that *In* works to convert **5b** to more electrophilic boron species **9** and **10**, thereby probably inducing the generation of **4b** in the presence of **1a**.

**Scheme 8:**
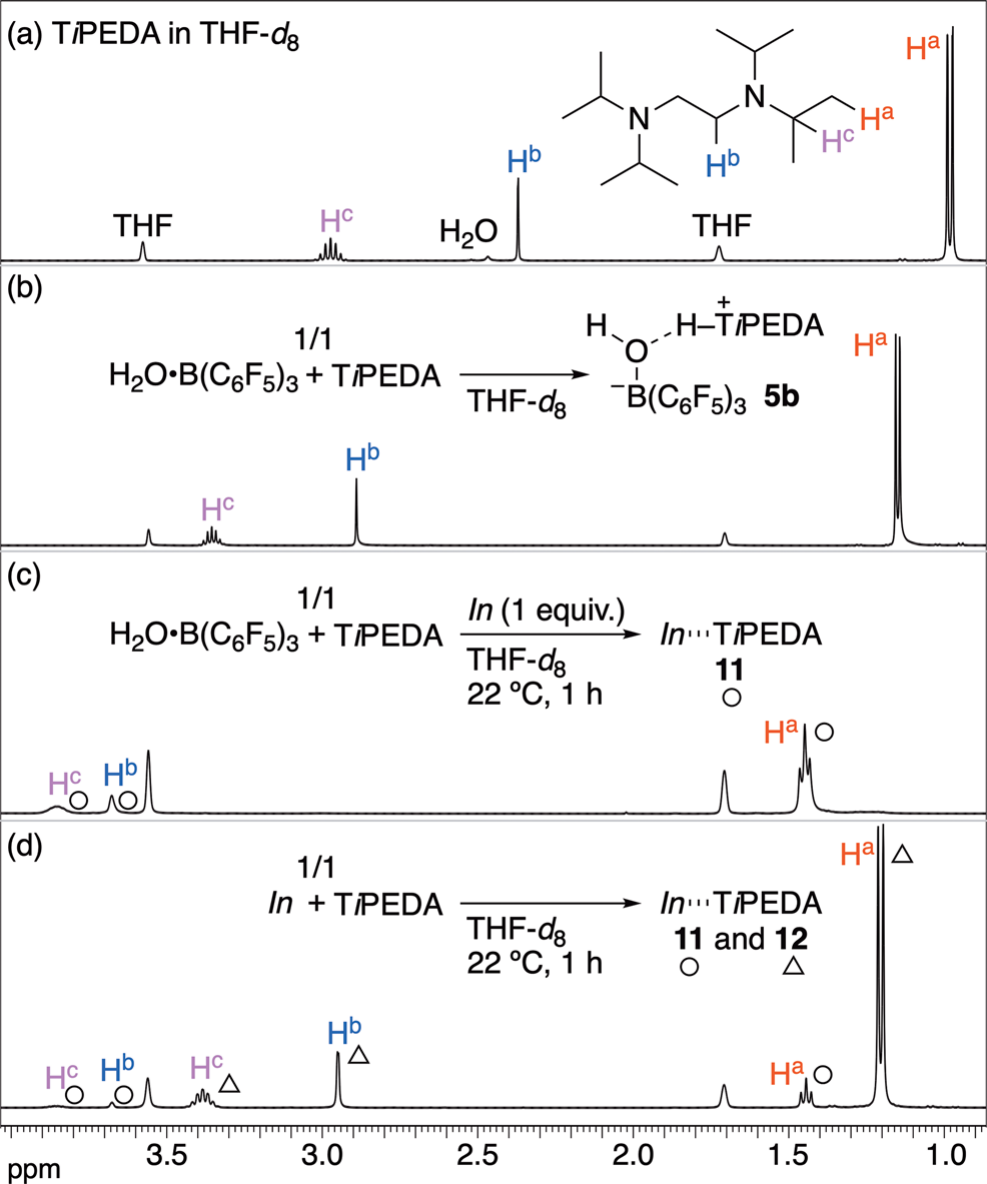
^1^H NMR Analysis for Exploration of Role of In­(ONf)_3_

Based on our insights on the indium–heteroarene π-complex,^12a,12d–12f,12h,12i,20,27^ we predicted that the coordination of **1a** to In­(ONf)_3_ may be another trigger for the formation of **4b**. Hence, **1a** was exposed to catalyst In­(ONf)_3_ and D_2_O in THF at 22 °C for 1 h, giving **1a**-*d* with 17% D at both the C2 and C3 positions (Scheme 9). By contrast, no C2/3-deuterations occurred without In­(ONf)_3_ (*In*). Note that the H–D exchange on the nitrogen atom of **1a** occurred even in the absence of *In*. A possible route for the observed C2/3-deuterations is proposed in Scheme 9, which refers to some precedents regarding: (i) the *In*–heteroarene π-complex, (ii) the α-nucleophilicity of pyrroles,^4^ (iii) the reaction of pyrroles with Lewis acids,^10,16,28^ and (iv) the deuteration of allylindium species (highlighted in pale purple).^12e,29^ Moreover, the proposed route may be extended to the route leading to **4b**, as shown in Scheme 9. Thus, *In* activates **1a** via the formation of π-complex **13** and then reacts with **1a** at the more nucleophilic α-site to yield **14**, followed by isomerization to **15**. The nitrogen atom of **15** should be more nucleophilic than that of **1a** and be able to attack the boron center of Y_2_O**·** *B* species **9** and **10** (Schemes 7f and 7g), followed by the deprotonation of the C(2)–H by T*i*PEDA to provide **4b**. This route fits well with the proposed route for the C2/3-deuterations of **1a**, similarly starting with the activation of **1a** by *In*.

**Scheme 9:**
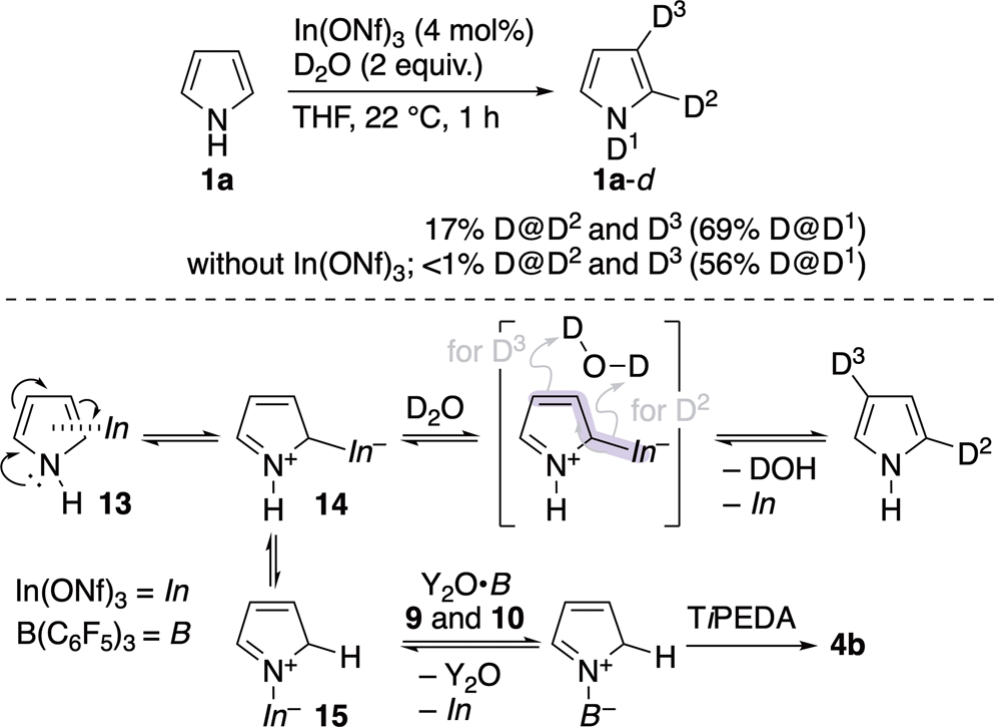
Deuteration of Pyrrole by In­(ONf)_3_ and Its Possible Route

Lastly, we examined the reactivity of **4b** and found that, without In­(ONf)_3_ at 70 °C for 24 h, **4b** itself does not have nucleophilicity to add to **2a** (Scheme 10a). Meanwhile, part of **4b** was thermally decomposed into pyrrole (**1a**) (12%), and **2a** was quantitatively recovered. Since **4b** adds to **2a** even at 30 °C in the presence of In­(ONf)_3_ (Scheme 5), it is considered that In­(ONf)_3_ would act to increase the electrophilicity of **2a**, as previously reported.^12j,30^ As presented in Scheme 10b, the decomposition of **4b** was promoted by In­(ONf)_3_ (1 equiv) at 30 °C. This result proposes that In­(ONf)_3_ also affects the decomposition of a β-**3**-based (*N*-pyrrolyl)­borate complex generated during the reaction, thereby regenerating B­(C_6_F_5_)_3_. This becomes one of the roles of the indium catalyst in the catalytic reaction.

**Scheme 10:**
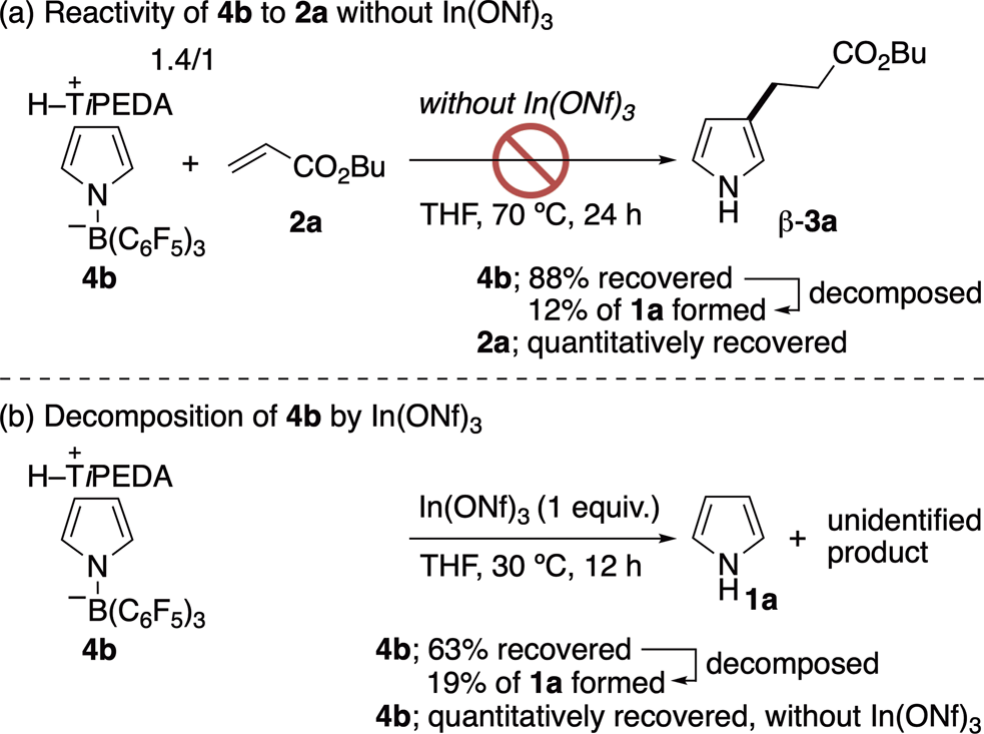
Reactivity and Decomposition of **4b**

Summarizing the results in Schemes 7–10, the indium salt is currently thought to uniquely have four roles in the catalytic reaction: activation of **5b**, activation of **1a**, activation of **2a**, and decomposition of (*N*-pyrrolyl)­borate complexes.

Considering all the above experimental results and key references, we propose a catalytic reaction mechanism, exemplified by the reaction of **1a** with acrylic acid ester **2** (Scheme 11). To begin with, **1a** π-coordinates to *In* to form **13** ^27b^ and then reacts with *In* at the α-position to afford **14**, due to the inherent α-nucleophilicity of pyrroles.^4^ Intermediate **14** bearing the C–metal bond isomerizes to more stable species **15** with the C–H bond.^10,16,28^ In another route, **5b** is generated from the reaction of H_2_O**·** *B* with T*i*PEDA and then reacts with *In* to furnish more electrophilic boron species Y_2_O**·** *B* **9** and **10**. *In* in **15** can thus be replaced with *B* of **9** and **10** to yield **16**. The C(2)–H of **16** is then deprotonated by T*i*PEDA for the aromatization, giving (*N*-pyrrolyl)­borate complex **4b**.^10,16^ Because of the effective steric shield around its α-site, key intermediate **4b** adds at its β-position to **2** activated by *In* via S_E_Ar. The resulting (*N*-pyrrolyl)­borate complex **17** decomposes thermally and/or by the action of *In*, producing β-**3** and regenerating T*i*PEDA and *B*. At this final stage, a proton (H^+^) arising from the complexation between *In* and T*i*PEDA may effectively protonate the N–*B* ^–^ bond in **17** to form β-**3**.

**Scheme 11:**
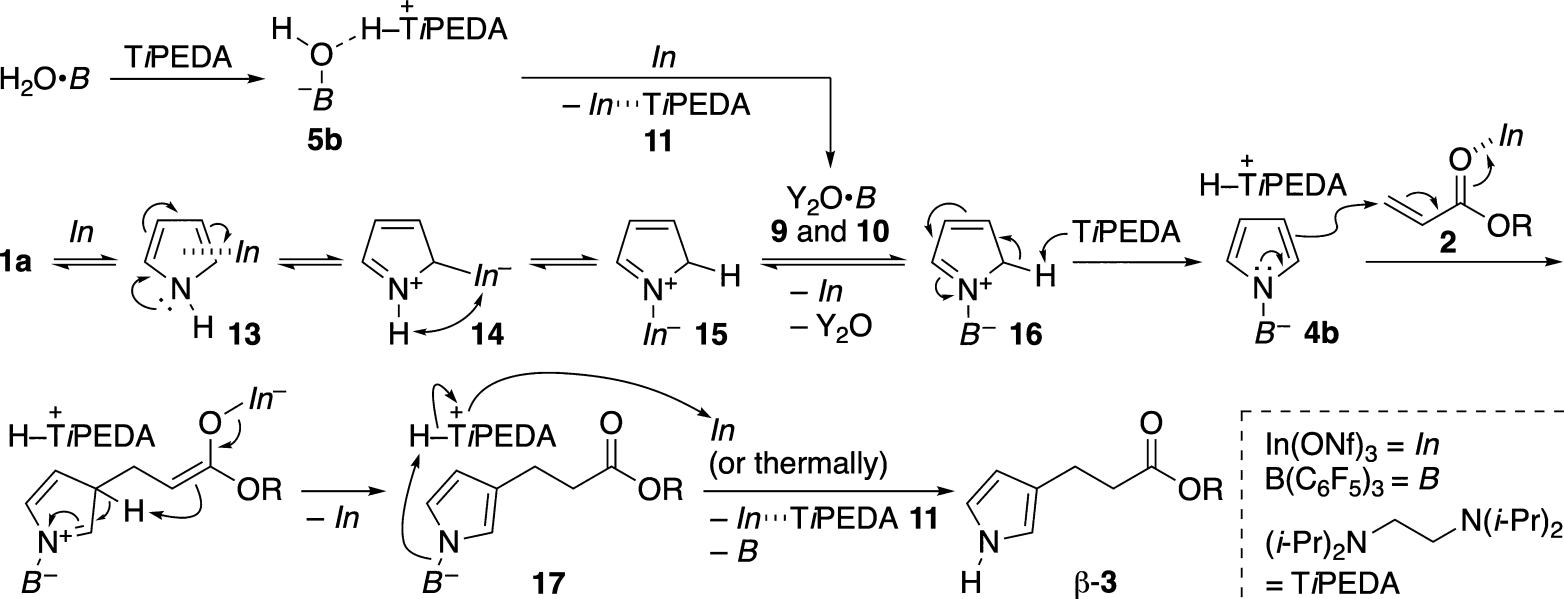
Proposed Catalytic Reaction Mechanism

## Conclusions

In summary, we disclosed the first example of catalytically proceeding β-preferential S_E_Ar-APEda using the ternary system of B­(C_6_F_5_)_3_/T*i*PEDA/In­(ONf)_3_ whereas the pyrrole substrate was limited to pyrrole (**1a**). Unlike the catalytic reaction, when using stoichiometric (*N*-pyrrolyl)­borate complex **4**, pyrrole substrates other than **1a** were available. A range of electron-deficient alkenes **2** could be also used. The complete β-selectivity is particularly noteworthy. This stoichiometric mode can be achieved using a catalytic amount of an indium salt under mild, room-temperature reaction conditions. The mechanistic studies of the catalytic reaction revealed that **4** is a key intermediate, and, interestingly, the indium catalyst plays multiple roles.

Due to the moderate β-selectivity and limited substrate scope in the catalytic reaction using the β-directing-group-free pyrrole, their improvements, possibly by applying a distinct method or strategy, continue to be a significant challenge for future research.

## Supplementary Material





## Notes

Experimental procedures, compound characterization data, and copies of NMR spectra (PDF)
X-ray crystallographic data for **4b** (CIF)
