# Supplementary material for: β‑Selective Addition of Pyrroles to Electron-Deficient Alkenes in Both Catalytic and Stoichiometric Modes on B(C6F5)3
Source: ACS Omega. 2025 May 19;10(21):21510–8. doi: 10.1021/acsomega.5c00371 (PMC12138597; doi:10.1021/acsomega.5c00371)

Supporting Information for

β-Selective Addition of Pyrroles to Electron-Deficient Alkenes in Both Catalytic and Stoichiometric Modes on $B(C_6F_5)_3$

Seina Sekine, Miho Kashiwa, Maho Kawakami, Takumi Sonoda, Arisa Ono, and Teruhisa Tsuchimoto*

Department of Applied Chemistry, School of Science and Technology, Meiji University, 1-1-1 Higashimita, Tama-ku, Kawasaki 214-8571, Japan.

I. General Remarks

Unless otherwise noted, manipulations were conducted with a standard Schlenk technique under an argon atmosphere. Nuclear magnetic resonance (NMR) spectra were taken on a JEOL JNM-ECA 400 [^{1}H (400 MHz); ^{13}C{^{1}H}, ^{13}C{^{19}F} (100 MHz); ^{11}B{^{1}H} (128 MHz); ^{19}F (376 MHz)], a JEOL JNM-ECZ 400S [^{1}H (400 MHz); ^{13}C{^{1}H}, ^{13}C{^{19}F} (100 MHz)], or a JEOL JNM-ECA 500 [^{1}H (500 MHz); ^{13}C{^{1}H} (125 MHz); ^{11}B{^{1}H} (160 MHz); ^{19}F (471 MHz)] spectrometer using tetramethylsilane (^{1}H and ^{13}C{^{1}H}, $\delta = 0.00$) or trichlorofluoromethane (^{19}F, $\delta = 0.00$) as an internal standard, and boron trifluoride diethyl etherate ($BF_3{\bullet}Et_2O$) (^{11}B{^{1}H}, $\delta = 0.00$) as an external standard. Analytical gas chromatography (GC) was performed on a Shimadzu model GC-2014 instrument equipped with a capillary column of InertCap 5 (5% diphenyl- and 95% dimethylpolysiloxane, 30 m × 0.25 mm × 0.25 μm) and with a FID detector, using nitrogen as carrier gas. Gas chromatography-mass spectrometry (GC-MS) analyses were performed with a Shimadzu model GCMS-QP2010 SE instrument equipped with a capillary column of InertCap 5 by electron ionization at 70 eV using helium as carrier gas. Preparative recycling gel permeation chromatography (GPC) was performed with JAI LC-9105 equipped with JAIGEL-1H and JAIGEL-2H columns using chloroform as eluent. Electrospray ionization mass spectroscopy (ESI-MS) was carried out using a Shimadzu model LCMS-2020 instrument, into which acetonitrile was injected directly at a flow rate of 300 μL/min and a pressure of 0.6 MPa. High-resolution mass spectra (HRMS) were obtained with a JEOL JMS-T100GCV spectrometer. Melting points were measured with a Yanaco Micro Melting Point apparatus and are uncorrected. Kugelrohr bulb-to-bulb distillation was carried out with a Sibata glass tube oven GTO-250RS apparatus. Elemental analysis was performed on a Vario EL III elemental analysis instrument.

Single-crystal X-ray diffraction analysis was conducted on a Rigaku MicroMax-007HFM. Tetrahydrofuran (THF), diethyl ether (Et_2O) and 1,2-dimethoxyethane (DME) were distilled from sodium benzophenone ketyl under argon just prior to use. 1,4-Dioxane was distilled from sodium under argon just prior to use. Propionitrile (EtCN) was distilled from P_2O_5 under argon just prior to use. Dichloromethane, chlorobenzene (PhCl), and toluene were distilled from $CaCl_2$ under argon just prior to use. Esters (MeOAc, EtOAc, and PrOAc; Ac = acetyl), 2-propanol, 1,3-dimethyl-2-imidazolidinone (DMI), tetrahydropyran (THP), 1,2-dichloroethane, cyclohexane, amines, pyridine, and allyltrimethylsilane were stored over molecular sieves 4 Å (MS 4Å) under argon. Dehydrated *N*,*N*-dimethylformamide (DMF) (>99.5%, water: 0.001% max) was purchased from Kanto Chemical Co. Inc. and used as received. Pyrrole (**1a**), electron-deficient alkenes **2**, BF_3•Et_2O, BF_3•THF, and acryloyl chloride were distilled under reduced pressure and stored under argon. Proton sponge and H_2O•$B(C_6F_5)_3$ were sublimated at 40 °C/25 Pa and 110 °C/40 Pa, respectively, and stored under argon. The following indium salts, boron compounds, and pyrrole substrates were synthesized according to the respective literature methods: $In(ONf)_3$ (Nf = $SO_2C_4F_9$),[1] $In(NTf_2)_3$ (Tf = SO_2CF_3),[2] tris(2,4,6-trifluorophenyl)borane,[3] 3-methylpyrrole (**1b**),[4] 2-phenylpyrrole.[5] Unless otherwise noted, other substrates and reagents were commercially available and used as received without further purification. *Unless otherwise noted, signals of carbon atoms attached to boron atoms in* $^{13}C\{^1H\}$ *NMR spectra were not observed due to quadrupolar relaxation of boron.*

II. Preparation of Metal Salts, Reagents and Substrates

ZnO + HONf → $Zn(ONf)_2$ [H_2O (45 mL), 100 °C, 22 h]

15.0 mmol 15.0 mmol 97% yield (4.83 g)

---

[1] T. Tsuchimoto, H. Matsubayashi, M. Kaneko, Y. Nagase, T. Miyamura, E. Shirakawa, *J. Am. Chem. Soc.* **2008**, *130*, 15823–15835.
[2] a) C. G. Frost, J. P. Hartley, D. Griffin, *Tetrahedron Lett.* **2002**, *43*, 4789–4791; b) M. Nakamura, K. Endo, E. Nakamura, *Adv. Synth. Catal.* **2005**, *347*, 1681–1686.
[3] I. Khan, M. Manzotti, G. J. Tizzard, S. J. Coles, R. L. Melen, L. C. Morrill, *ACS Catal.* **2017**, *7*, 7748–7752.
[4] S. Nagy, Á. Szigetvári, V. Ilkei, B. Krámos, Z. Béni, C. Szántay Jr., L. Hazai, *Tetrahedron* **2021**, *81* 131827.
[5] J. Wen, R.-Y. Zhang, S.-Y. Chen, J. Zhang, X.-Q. Yu, *J. Org. Chem.* **2012**, *77,* 766–771.

Preparation of Zinc(II) Nonafluorobutanesulfonate [Zn(ONf)$_2$]. The synthesis of Zn(ONf)$_2$ was carried out with reference to the method reported in the literature.[1] ZnO (1.22 g, 15.0 mmol) was placed in a 200 mL two-necked round-bottomed flask equipped with a reflux condenser. To this were added H_2O (45 mL) and HONf (4.50 g, 15.0 mmol), and the resulting mixture was stirred at 100 °C for 22 h. Filtration through a pad of Celite to remove excess ZnO and evaporation of H_2O gave hydrate of Zn(ONf)$_2$. The resulting hydrate was slowly warmed to 80 °C under vacuum (ca. 5 Pa), and the heating at 80 °C was continued overnight. After roughly breaking the solid in the flask into smaller pieces, the temperature was then slowly raised to 150 °C under vacuum (ca. 5 Pa), and the heating at 150 °C was continued for additional 8 h to give Zn(ONf)$_2$ in 97% yield (4.83 g) as a white powder. Zn(ONf)$_2$ was characterized by ^{13}C{^{19}F} and ^{19}F NMR spectroscopy, as follows: ^{13}C{^{19}F} NMR (100 MHz, CD_3CN) δ 118.5, 114.8, 111.8, 110.0. [The chemical shifts were referenced to the CD_3 signal (1.32 ppm) of the solvent.[6]]; ^{19}F NMR (376 MHz, CD_3CN) δ –80.46 to –80.52 (m, 6F), –114.3 to –114.4 (m, 4F), –120.98 to –121.00 (m, 4F), –125.4 (t, *J* = 16.2 Hz, 4F).

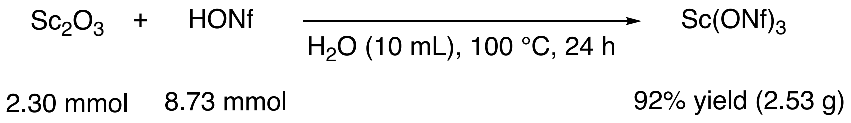

Preparation of Scandium(III) Nonafluorobutanesulfonate [Sc(ONf)$_3$]. The synthesis of Sc(ONf)$_3$ was carried out with reference to the method reported in the literature.[1] Sc_2O_3 (317 mg, 2.30 mmol) was placed in a 50 mL two-necked round-bottomed flask equipped with a reflux condenser. To this were added H_2O (10 mL) and HONf (2.62 g, 8.73 mmol), and the resulting mixture was stirred at 100 °C for 24 h. Filtration through a pad of Celite to remove excess Sc_2O_3 and evaporation of H_2O gave hydrate of Sc(ONf)$_3$. The resulting hydrate was slowly warmed to 80 ºC under vacuum (ca. 5 Pa), and the heating at 80 °C was continued for 7 h. After roughly breaking the solid in the flask into smaller pieces, the temperature was then slowly raised to 150 °C under vacuum (ca. 5 Pa), and the heating at 150 °C was continued for additional 10 h to give Sc(ONf)$_3$ in 92% yield (2.53 g) as a white powder. Sc(ONf)$_3$ was characterized by ^{13}C{^{19}F} and ^{19}F NMR spectroscopy, as follows: ^{13}C{^{19}F} NMR (100 MHz, CD_3CN) δ 114.3, 111.3, 109.8. (One carbon signal is missing due to overlapping with the solvent signal at 118.3 ppm.) [The chemical shifts were referenced to the CD_3 signal (1.32 ppm) of the solvent.[6]]; ^{19}F NMR (471

[6] H. E. Gottlieb, V. Kotlyar, A. Nudelman, *J. Org. Chem.* **1997**, *62*, 7512–7515.

MHz, CD_3CN) δ –80.3 to –80.4 (m, 9F), –113.27 to –113.32 (m, 6F), –120.82 to –120.84 (m, 6F), –125.3 (m, 6F).

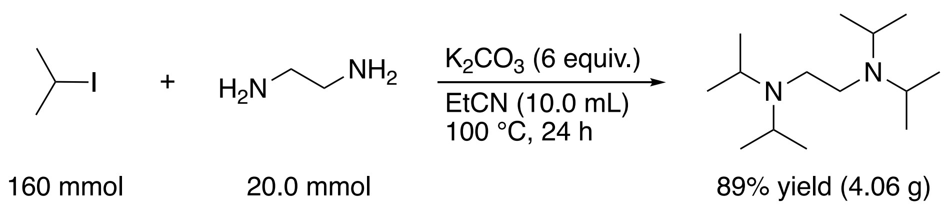

Synthesis of *N,N,N',N'*-Tetraisopropylethylenediamine (T*i*PEDA). T*i*PEDA was prepared according to the following modified literature procedure.[7] Under an argon atmosphere, a flame-dried 200 mL two-necked round-bottomed flask was charged with 2-iodopropane (27.2 g, 160 mmol), ethylenediamine (1.20 g, 20.0 mmol), K_2CO_3 (16.6 g, 120 mmol), and EtCN (10.0 mL). After stirring at 100 °C for 24 h, H_2O (15 mL) was added to the mixture, and the aqueous phase was extracted with Et_2O (30 mL × 3). The combined organic layer was washed with brine (25 mL) and then dried over anhydrous sodium sulfate. Filtration through a cotton plug and evaporation of the solvent followed by short-path distillation under reduced pressure (75 ºC/250 Pa) provided T*i*PEDA in 89% yield (4.06 g) as a colorless oil. ^{1}H NMR (400 MHz, $CDCl_3$) δ 2.97 (sept, J = 6.6 Hz, 4H), 2.37 (s, 4H), 1.01 (d, J = 6.4 Hz, 24H); ^{13}C{^{1}H} NMR (125 MHz, $CDCl_3$) δ 49.2, 47.9, 20.9. HRMS (FI) Calcd for $C_{14}H_{32}N_2$: M, 228.2566. Found: *m/z* 228.2563.

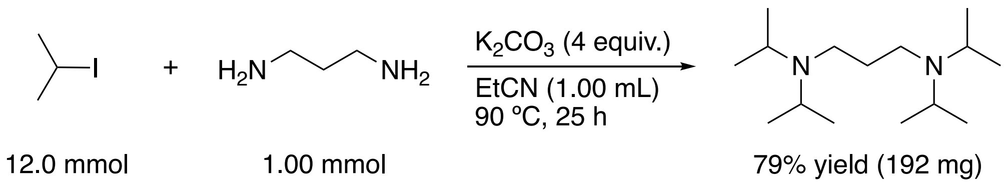

Synthesis of *N,N,N',N'*-Tetraisopropyl-1,3-propanediamine (T*i*PPDA). T*i*PPDA was prepared according to the following modified literature procedure.[7] Under an argon atmosphere, a flame-dried 20 mL Schlenk tube was charged with 2-iodopropane (2.04 g, 12.0 mmol), 1,3-diaminopropane (74.1 mg, 1.00 mmol), K_2CO_3 (553 mg, 4.00 mmol), and EtCN (1.00 mL). After stirring at 90 ºC for 25 h, a 2 M KOH aqueous solution (3 mL) was added to the mixture, and the aqueous phase was extracted with Et_2O (7 mL × 3). The combined organic layer was washed with brine (5 mL) and then dried over anhydrous sodium sulfate. Filtration through a cotton plug and

[7] W. J. Marshall, V. V. Grushin, *Can. J. Chem.* **2005**, *83*, 640–645.

evaporation of the solvent followed by column chromatography on silica gel (hexane/EtOAc/NEt_3 = 100/1/1) gave T*i*PPDA in 79% yield (192 mg) as a colorless oil. ^1H NMR (500 MHz, $CDCl_3$) δ 3.01 (sept, *J* = 6.5 Hz, 4H), 2.38 (t, *J* = 7.7 Hz, 4H), 1.50 (quint, *J* = 7.5 Hz, 2H), 1.00 (d, *J* = 6.3 Hz, 24H); $^{13}C\{^1H\}$ NMR (125 MHz, $CDCl_3$) δ 48.3, 43.3, 32.5, 20.7. HRMS (FI) Calcd for $C_{15}H_{34}N_2$: M, 242.2722. Found: *m/z* 242.2693.

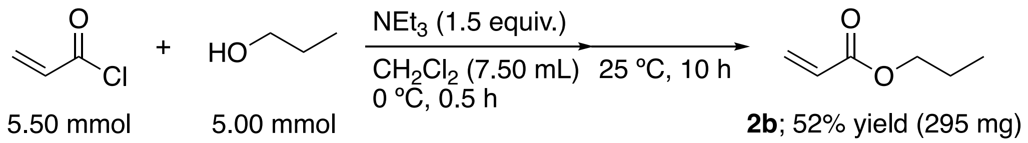

Synthesis of Propyl Acrylate (2b). According to the literature procedure,[8] **2b** was prepared using the following reagents: acryloyl chloride (498 mg, 5.50 mmol), 1-propanol (301 mg, 5.00 mmol), triethylamine (759 mg, 7.50 mmol), and CH_2Cl_2 (7.50 mL). Propyl acrylate (**2b**) was isolated by Kugelrohr bulb-to-bulb distillation under reduced pressure (85 °C/127 hPa) in 52% yield (295 mg) as a colorless oil. ^1H NMR (400 MHz, $CDCl_3$) δ 6.40 (dd, *J* = 17.2, 1.6 Hz, 1H), 6.13 (dd, *J* = 17.2, 10.3 Hz, 1H), 5.82 (dd, *J* = 10.1, 1.4 Hz, 1H), 4.12 (t, *J* = 6.9 Hz, 2H), 1.70 (sext, *J* = 7.1 Hz, 2H), 0.97 (t, *J* = 7.3 Hz, 3H); $^{13}C\{^1H\}$ NMR (100 MHz, $CDCl_3$) δ 166.4, 130.4, 128.7, 66.1, 22.0, 10.4. HRMS (FI) Calcd for $C_6H_{10}O_2$: M, 114.0681. Found: *m/z* 114.0677.

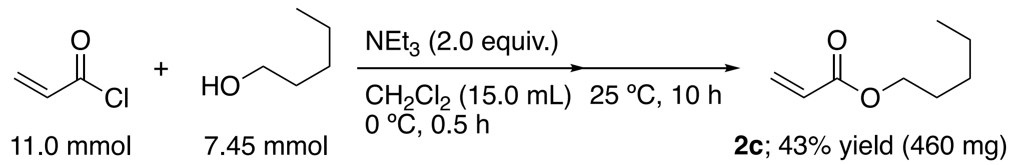

Synthesis of Pentyl Acrylate (2c). According to the literature procedure,[8] **2c** was prepared using the following reagents: acryloyl chloride (996 mg, 11.0 mmol), 1-pentanol (657 mg, 7.45 mmol), triethylamine (1.52 g, 15.0 mmol), and CH_2Cl_2 (15.0 mL). Pentyl acrylate (**2c**) was isolated by Kugelrohr bulb-to-bulb distillation under reduced pressure (80 °C/8 hPa) in 43% yield (460 mg) as a colorless oil. ^1H NMR (400 MHz, $CDCl_3$) δ 6.40 (dd, *J* = 17.2, 1.6 Hz, 1H), 6.12 (dd, *J* = 17.4, 10.5 Hz, 1H), 5.81 (dd, *J* = 10.3, 1.6 Hz, 1H), 4.15 (t, *J* = 6.9 Hz, 2H), 1.71–1.64 (m, 2H), 1.40–1.31 (m, 4H), 0.94–0.89 (m, 3H); $^{13}C\{^1H\}$ NMR (100 MHz, $CDCl_3$) δ 166.4, 130.4, 128.7, 64.7, 28.3, 28.1, 22.3 14.0. HRMS (FI) Calcd for $C_8H_{14}O_2$: M, 142.0994. Found: *m/z*

[8] B. Zhou, P. Ma, H. Chen, C. Wang, *Chem. Commun.* **2014**, *50*, 14558–14561.

142.0975.

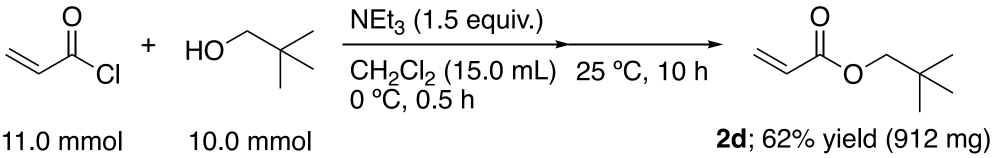

Synthesis of Neopentyl Acrylate (2d). According to the literature procedure,[8] **2d** was prepared using the following reagents: acryloyl chloride (996 mg, 11.0 mmol), 2,2-dimethyl-1-propanol (882 mg, 10.0 mmol), triethylamine (1.52 g, 15.0 mmol), and CH_2Cl_2 (15.0 mL). Neopentyl acrylate (**2d**) was isolated by Kugelrohr bulb-to-bulb distillation under reduced pressure (70 ºC/30 hPa) in 62% yield (912 mg) as a colorless oil. ^{1}H NMR (400 MHz, $CDCl_3$) δ 6.41 (dd, J = 17.4, 1.8 Hz, 1H), 6.15 (dd, J = 17.4, 10.5 Hz, 1H), 5.83 (dd, J = 10.3, 1.6 Hz, 1H), 3.86 (s, 2H), 0.97 (s, 9H); ^{13}C{^{1}H} NMR (100 MHz, $CDCl_3$) δ 166.4, 130.4, 128.7, 73.8, 31.4, 26.5. HRMS (FI) Calcd for $C_8H_{14}O_2$: M, 142.0994. Found: *m/z* 142.0987.

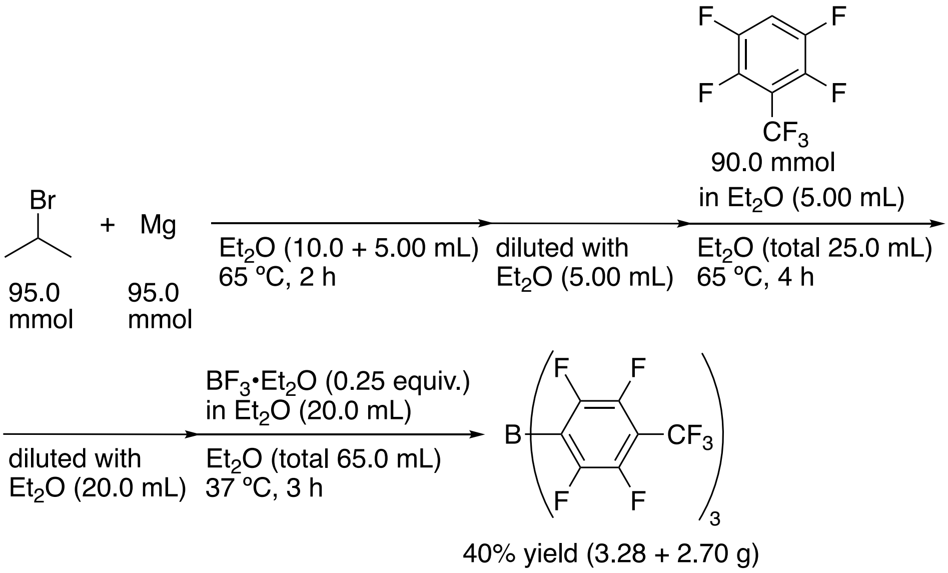

Synthesis of Tris[2,3,5,6-tetrafluoro-4-(trifluoromethyl)phenyl]borane [B(C_6F_4–*p*-CF_3)$_3$]. A 50 mL three-necked round-bottomed flask **A** equipped with a reflux condenser and a dropping funnel was charged with magnesium powder (2.31 g, 95.0 mmol) and heated with a heat gun in vacuo (ca. 5 Pa) for 5 min. After cooling down to room temperature, the flask was filled with argon and charged with Et_2O (10.0 mL). A solution of 2-bromopropane (11.7 g, 95.0 mmol) in Et_2O (5.00 mL) was added dropwise over 1 h from the dropping funnel. The resulting mixture was stirred at 65 ºC for 2 h and then diluted with Et_2O (5.00 mL). Under an argon atmosphere, a

flame-dried 100 mL three-necked round-bottomed flask **B** equipped with a reflux condenser and a dropping funnel **C** was charged with 2,3,5,6-tetrafluorobenzotrifluoride (19.6 g, 90.0 mmol) and Et_2O (5.00 mL). The Et_2O solution of *i*-PrMgBr prepared in the flask **A** was transferred through a cannula into the dropping funnel **C** and then added dropwise over 1 h to the mixture in the flask **B**. After stirring at 65 ºC for 4 h, the resulting mixture was diluted with Et_2O (20.0 mL). Under an argon atmosphere, a flame-dried 200 mL three-necked round-bottomed flask **D** equipped with a reflux condenser and a dropping funnel **E** was charged with BF_3•Et_2O (3.19 g, 22.5 mmol) and Et_2O (20.0 mL). The Et_2O solution prepared in the flask **B** was transferred through a cannula into the dropping funnel **E** and then added dropwise over 1 h to the mixture in the flask **D**. After stirring at 37 ºC for 3 h, the mixture was concentrated under reduced pressure. To the resulting crude mixture was added hexane (400 mL), and the mixture was heated to the boiling point of hexane and then decanted. Concentration of the resulting hexane solution followed by sublimation (110 °C/10 Pa) gave $B(C_6F_4$–*p*-$CF_3)_3$ in 22% yield (3.28 g). Besides this, the residue remained after the decantation was recrystallized from hexane to afford another crop of $B(C_6F_4$–*p*-$CF_3)_3$ in 18% yield (2.70 g). $B(C_6F_4$–*p*-$CF_3)_3$ (3.28 + 2.70 g) is further sublimated, as necessary. Although $B(C_6F_4$–*p*-$CF_3)_3$ has already appeared in the literature,[9] its spectral and analytical data were not in good agreement with those reported. Accordingly, $B(C_6F_4$–*p*-$CF_3)_3$ was characterized by $^{13}C\{^{19}F\}$, ^{19}F, and $^{11}B\{^1H\}$ NMR spectroscopy and HRMS. **NOTE**: The ^{11}B NMR signal was observed not at a typical range of B(*sp*^{2})-hybridized species but at a typical range of B(*sp*^{3})-hybridized species. Accordingly, the structure of the boron compound characterized by NMR spectroscopy here is considered to be appropriately represented by H_2O•$B(C_6F_4$–*p*-$CF_3)_3$, just as $B(C_6F_5)_3$ is represented by H_2O•$B(C_6F_5)_3$. A white solid (mp 165–167 °C). $^{13}C\{^{19}F\}$ NMR (100 MHz, $CDCl_3$) δ 148.0, 143.8, 121.0, 109.2 [The chemical shifts were referenced to the solvent carbon signal (77.16 ppm).[6]]; ^{19}F NMR (376 MHz, $CDCl_3$) δ –56.9 (t, J = 20.6 Hz, 9F), –133.6 to –133.8 (m, 6F), –141.4 to –141.8 (m, 6F); $^{11}B\{^1H\}$ NMR (160 MHz, $CDCl_3$) δ –1.92. HRMS (FD) Calcd for $C_{21}BF_{21}$: M, 661.9758. Found: *m/z* 661.9787.

III. Examination of Suitable Reaction Conditions for β-Preferential Addition of Pyrrole to Butyl Acrylate in Catalytic Mode on Boron [$B(C_6F_5)_3$]

[9] L. A. Körte, J. Schwabedissen, M. Soffner, S. Blomeyer, C. G. Reuter, Y. V. Vishnevskiy, B. Neumann, H.-G. Stammler, N. W. Mitzel, *Angew. Chem. Int. Ed.* **2017**, *56*, 8578–8582.

In this section, the promising results in each table are highlighted with a gray background.

The results of **Table S1** shows that all the boron, amine, and indium compounds are required for the formation of β-**3a**.

Table S1. Effect with/without H_2O•$B(C_6F_5)_3$, NEt_3, and/or $In(OTf)_3$[a]

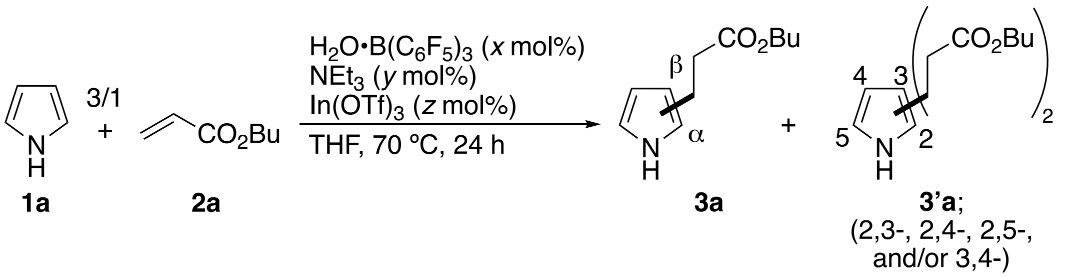

x	*y*	*z*	conv. (%) of **2a**[b]	yield (%) of **3a** (β/α)[b]	yield (%) of **3'a**[c]
10	10	0	<1	<1	<1
**10**	**10**	**10**	**21**	**10 (4:96)**	**11**
0	10	10	>99	68 (<1:99)	13
0	0	10	72	24 (<1:99)	19
10	0	0	2	<1	<1

[a]Reagents: **1a** (1.50 mmol), **2a** (0.500 mmol), H_2O•$B(C_6F_5)_3$ (0 or 48.3 μmol), NEt_3 (0 or 50.0 μmol), $In(OTf)_3$ (0 or 50.0 μmol), THF (0.800 mL). [b]Determined by GC. [c]Determined by NMR.

Encouraged by the above results, we continued to carefully explore superior reaction conditions and found that the more suitable amounts of H_2O•$B(C_6F_5)_3$, NEt_3, and $In(OTf)_3$ are 19, 50, and 20 mol%, respectively (**Table S2**). With this reaction conditions, the β/α selectivity was inverted, yielding β-**3a** in 60% selectivity.

Table S2. Effect of catalytic amounts of H_2O•$B(C_6F_5)_3$, NEt_3, and $In(OTf)_3$[a]

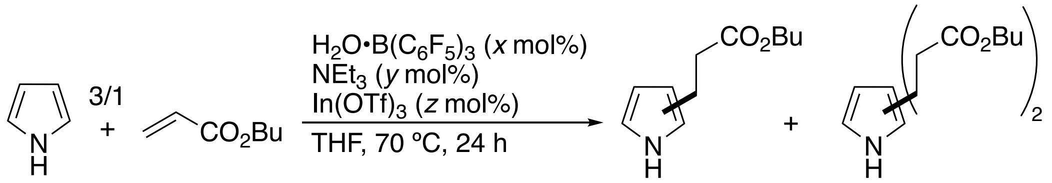

1a **2a** **3a** **3'a**; (2,3-, 2,4-, 2,5-, and/or 3,4-)

x	*y*	*z*	conv. (%) of **2a**[b]	yield (%) of **3a** (β/α)[b]	yield (%) of **3'a**[c]
5	5	5	14	6 (2:98)	3
10	10	10	21	10 (4:96)	11
19	20	20	82	21 (6:94)	22
29	30	30	>99	13 (7:93)	29
19	30	20	69	17 (9:91)	18
19	40	20	87	31 (22:78)	17
**19**	**50**	**20**	**58**	**26 (60:40)**	**17**
19	60	20	49	26 (59:41)	6

[a]Reagents: **1a** (1.50 mmol), **2a** (0.500 mmol), H_2O•$B(C_6F_5)_3$ (24.2, 48.3, 96.6, or 145 μmol), NEt_3 (25.0, 50.0, 100, 150, 200, 250, or 300 μmol), $In(OTf)_3$ (25.0, 50.0, 100, or 150 μmol), THF (0.800 mL). [b]Determined by GC. [c]Determined by NMR.

As shown in **Table S3**, increasing the amount of **1a** (5 equiv.) raised the yield of **3a** to 52% as well as the β-selectivity to 66%.

Table S3. Effect of amount of **1a**[a]

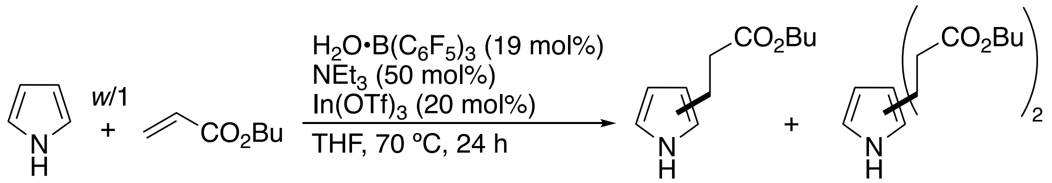

1a **2a** **3a** **3'a**; (2,3-, 2,4-, 2,5-, and/or 3,4-)

w	conv. (%) of **2a**[b]	yield (%) of **3a** (β/α)[b]	yield (%) of **3'a**[c]
3	58	26 (60:40)	17
4	60	30 (64:36)	7
**5**	**98**	**52 (66:34)**	**11**
6	98	67 (55:45)	13

[a]Reagents: **1a** (1.50, 2.00, 2.50, or 3.00 mmol), **2a** (0.500 mmol), H_2O•$B(C_6F_5)_3$ (96.6 μmol), NEt_3

(0.250 mmol), In(OTf)$_3$ (0.100 mmol), THF (0.800 mL). [b]Determined by GC. [c]Determined by NMR.

The results of screening Lewis acid catalysts are summarized in **Table S4**. The use of other metal triflates gave no improvements, but when using In(ONf)$_3$, there was a slight increase in both the yield and β-selectivity.

Table S4. Effect of Lewis acids[a]

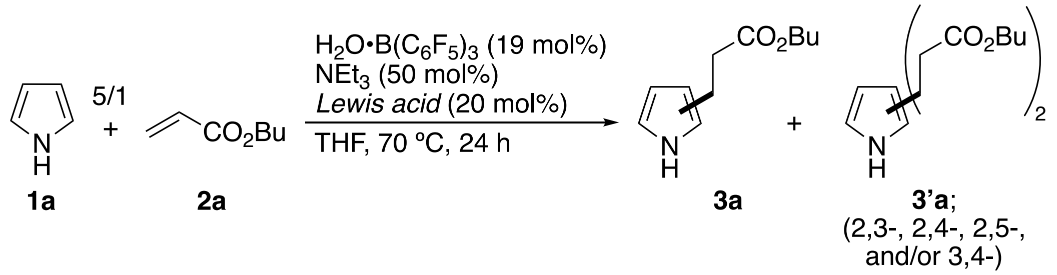

Lewis acid	conv. (%) of **2a**[b]	yield (%) of **3a** (β/α)[b]	yield (%) of **3'a**[c]	*Lewis acid*	conv. (%) of **2a**[b]	yield (%) of **3a** (β/α)[b]	yield (%) of **3'a**[c]
In(OTf)$_3$	98	52 (66:34)	11	Sm(OTf)$_3$	<1	<1	<1
Sc(OTf)$_3$	19	10 (57:43)	1	Yb(OTf)$_3$	1	<1	<1
Zn(OTf)$_3$	20	16 (<1:99)	<1	Bi(OTf)$_3$	14	<1	<1
Cu(OTf)$_3$	11	<1	<1	**In(ONf)$_3$**	**94**	**54 (69:31)**	**11**
Y(OTf)$_3$	<1	<1	<1	In(NTf$_2$)$_3$	29	12 (<1:99)	<1
Zr(OTf)$_4$	15	1 (<1:99)	1	InCl$_3$	3	<1	<1
AgOTf	4	<1	<1	InBr$_3$	<1	<1	<1

[a]Reagents: **1a** (2.50 mmol), **2a** (0.500 mmol), H_2O•$B(C_6F_5)_3$ (96.6 μmol), NEt_3 (0.250 mmol), Lewis acid (0.100 mmol), THF (0.800 mL). [b]Determined by GC. [c]Determined by NMR.

As shown in **Table S5**, when using $PhNEt_2$ and pyridine instead of NEt_3, the major adduct was α-**3a**. In the case of diamines such as $Me_2NCH_2CH_2NMe_2$ (TMEDA), adjusting the number of nitrogen atoms to 50 mol% was crucial, and the β-selectivity was elevated to 78% without lowering the yield. Switching TMEDA to (i-Pr)$_2$NCH$_2$CH$_2$N(i-Pr)$_2$ with the bulkier i-Pr groups made the yield higher to 74%. (i-Pr)$_2$NCH$_2$CH$_2$CH$_2$N(i-Pr)$_2$ with the longer carbon tether and 1,8-bis(dimethylamino)naphthalene were also effective, albeit with the lower yields and β-selectivities.

Table S5. Effect of organic bases[a]

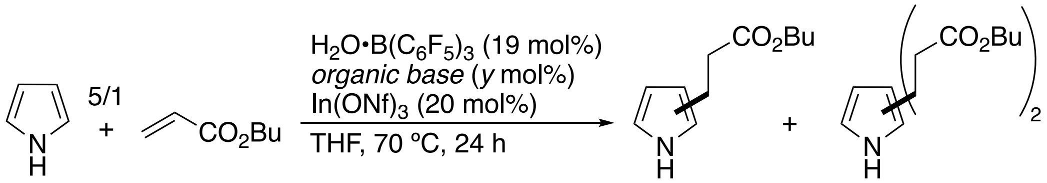

1a **2a** **3a** **3'a**; (2,3-, 2,4-, 2,5-, and/or 3,4-)

organic base	*y*	conv. (%) of **2a**[b]	yield (%) of **3a** (β/α)[b]	yield (%) of **3'a**[c]
NEt_3	50	94	54 (69:31)	11
$PhNEt_2$	50	>99	46 (28:72)	9
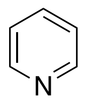	50	52	13 (<1:99)	8
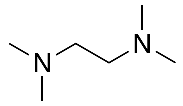	50	>99	74 (12:88)	13
	25	99	54 (78:22)	12
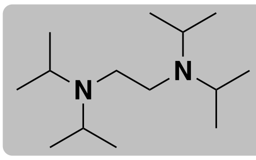	**25**	**>99**	**74 (77:23)**	**8**
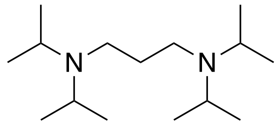	25	>99	66 (74:26)	19
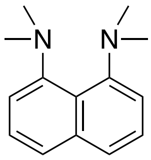	50	81	53 (72:28)	8

[a]Reagents: **1a** (2.50 mmol), **2a** (0.500 mmol), H_2O•$B(C_6F_5)_3$ (96.6 μmol), organic base (0.125 or 0.250 mmol), $In(ONf)_3$ (0.100 mmol), THF (0.800 mL). [b]Determined by GC. [c]Determined by NMR.

Among the solvents tested in **Table S6**, only EtOAc other than THF preferentially delivered β-**3a** but was less effective. The inability of DMF may be due to the strong coordinating nature to the boron/indium Lewis acids. Less polar ($ClCH_2CH_2Cl$) and nonpolar (cyclohexane) solvents were ineffective.

Table S6. Effect of solvents[a]

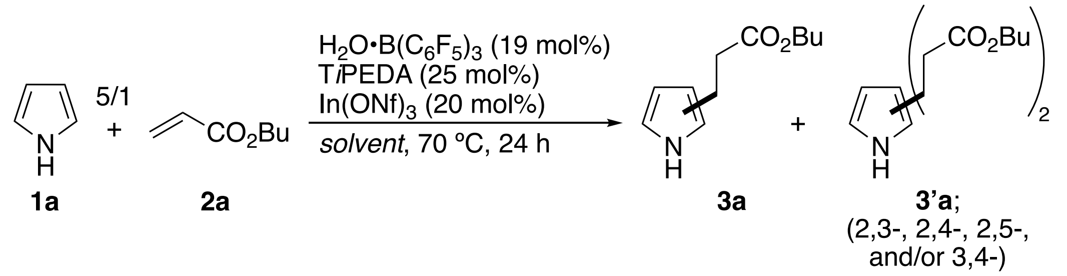

solvent	conv. (%) of **2a**[b]	yield (%) of **3a** (β/α)[b]	yield (%) of **3'a**[c]	*solvent*	conv. (%) of **2a**[b]	yield (%) of **3a** (β/α)[b]	yield (%) of **3'a**[c]
**THF**	**>99**	**74 (77:23)**	**8**	DMI	7	<1	<1
THP	37	20 (28:72)	9	*i*-PrOH	31	23 (37:63)	1
1,4-dioxane	58	29 (31:69)	5	$ClCH_2CH_2Cl$	90	57 (28:72)	10
EtOAc	77	40 (62:38)	8	PhCl	92	53 (24:76)	11
EtCN	16	2 (7:93)	<1	toluene	39	21 (4:96)	4
DMF	10	<1	<1	cyclohexane	92	47 (11:89)	8

[a]Reagents: **1a** (2.50 mmol), **2a** (0.500 mmol), $H_2O•B(C_6F_5)_3$ (96.6 μmol), T*i*PEDA (0.125 mmol), $In(ONf)_3$ (0.100 mmol), solvent (0.800 mL). [b]Determined by GC. [c]Determined by NMR.

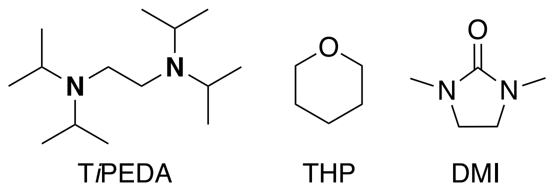

We also tested boron compounds other than $H_2O•B(C_6F_5)_3$ as shown in **Table S7** but was found that $H_2O•B(C_6F_5)_3$ is the boron catalyst of choice in this reaction.

Table S7. Effect of boron compounds[a]

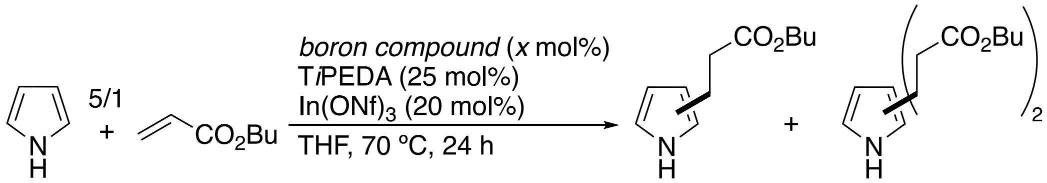

1a **2a** **3a** **3'a**; (2,3-, 2,4-, 2,5-, and/or 3,4-)

boron compound	*x*	conv. (%) of **2a**[b]	yield (%) of **3a** (β/α)[b]	yield (%) of **3'a**[c]
BF_3•THF	20	>99	23 (<1:99)	8
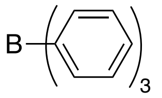	20	>99	39 (<1:99)	13
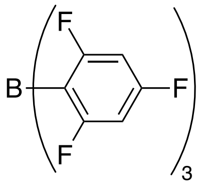	20	>99	20 (34:66)	8
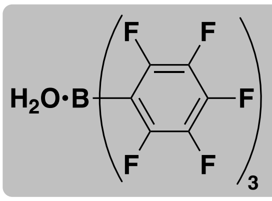	**19**	**>99**	**74 (77:23)**	**8**
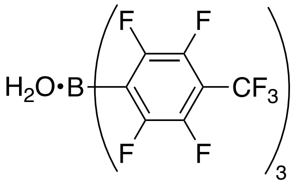	19	>99	37 (41:59)	18

[a]Reagents: **1a** (2.50 mmol), **2a** (0.500 mmol), boron compound (0.100 mmol or 96.6 μmol), T*i*PEDA (0.125 mmol), In(ONf)$_3$ (0.100 mmol), THF (0.800 mL). [b]Determined by GC. [c]Determined by NMR.

Spectral and Analytical Data of 1/2 Adducts 3'a. Through the examination of the reaction conditions for the catalytic reaction of **1a** with **2a** shown in **Tables S1–7**, 1/2 adducts **3'a** of **1a** and **2a** were detected as four regioisomers. Their spectral and analytical data are summarized below.

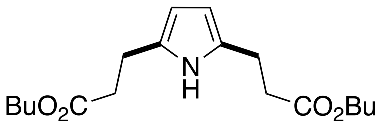

2,5-3'a

Compound 2,5-3'a. Among the four isomers, this 2,5-isomer was formed as a major one. Accordingly, **2,5-3'a** was characterized by ^1H and $^{13}C\{^1H\}$ NMR spectroscopy and HRMS.

Besides these data, GC-MS data are provided below and were referred to the identification of other isomers. ^{1}H NMR (400 MHz, $CDCl_3$) δ 8.54 (br s, 1H), 5.76 (d, *J* = 2.7 Hz, 2H), 4.10 (t, *J* = 6.6 Hz, 4H), 2.86 (t, *J* = 7.1 Hz, 4H), 2.60 (t, *J* = 6.9 Hz, 4H), 1.64–1.57 (m, 4H), 1.37 (sext, *J* = 7.5 Hz, 4H), 0.93 (t, *J* = 7.3 Hz, 6H); $^{13}C\{^1H\}$ NMR (100 MHz, $CDCl_3$) δ 173.9, 130.1, 105.1, 64.6, 34.5, 30.7, 22.8, 19.1, 13.7. HRMS (FI) Calcd for $C_{18}H_{29}NO_4$: M, 323.2097. Found: *m/z* 323.2124. MS (GC-MS, EI) Calcd for $C_{18}H_{29}NO_4$: M^+, 323. Found: *m/z* (relative intensity) 323(27) $[M]^+$, 250 (1), 222 (6), 208 (100), 148 (8), 106 (36), 94 (3), 41 (8).

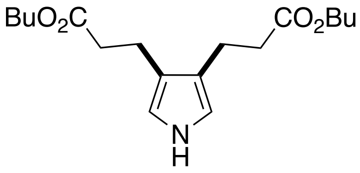

3,4-3’a

Compound 3,4-3’a. This isomer was also produced in the stoichiometric reaction (*vide infra*) and could be isolated as a pure form therefrom. Accordingly, **3,4-3’a** was analyzed by ^{1}H and $^{13}C\{^1H\}$ NMR spectroscopy and HRMS, along with the GC-MS data. ^{1}H NMR (500 MHz, $CDCl_3$) δ 7.88 (br s, 1H), 6.52 (d, *J* = 2.9 Hz, 2H), 4.08 (t, *J* = 6.6 Hz, 4H), 2.77 (dd, *J* = 8.6, 6.6 Hz, 4H), 2.58 (dd, *J* = 8.6, 6.9 Hz, 4H), 1.63–1.57 (m, 4H), 1.36 (sext, *J* = 7.6 Hz, 4H), 0.93 (t, *J* = 7.5 Hz, 6H); $^{13}C\{^1H\}$ NMR (125 MHz, $CDCl_3$) δ 173.5, 121.0, 115.2, 64.3, 35.1, 30.7, 20.6, 19.1, 13.7. HRMS (FI) Calcd for $C_{18}H_{29}NO_4$: M, 323.2097. Found: *m/z* 323.2097. MS (GC-MS, EI) Calcd for $C_{18}H_{29}NO_4$: M^+, 323. Found: *m/z* (relative intensity) 323 (55) $[M^+]$, 250 (16), 222 (34), 208 (55), 148 (26), 106 (100), 94 (47), 41 (17).

The other two isomers could not be separated as pure forms from the catalytic reaction, due to their small amounts formed. The generation of the two isomers was evaluated by comparison with the GC-MS data of **2,5-3’a** and **3,4-3’a** presented above. Moreover, proton signals on pyrrole rings and other distinct proton signals in ^{1}H NMR spectra were assigned. However, it has not been determined which isomer corresponds to which spectral/analytical data.

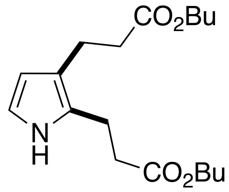

2,3-3’a

or

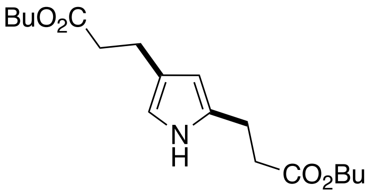

2,4-3’a

Compound 2,3-3’a or 2,4-3’a. ^{1}H NMR (400 MHz, $CDCl_3$) δ 8.50 (br s, 1H), 6.59 (t, *J* = 2.8 Hz, 1H), 5.97 (t, *J* = 2.8 Hz, 1H), 4.08 (q, *J* = 6.7 Hz, 4H), 2.88–2.84 (m, 2H), 2.73 (dd, *J* = 8.7, 6.9 Hz 2H), 2.59–2.51 (m, 4H), 1.63–1.56 (m, 4H), 1.40–1.31 (m, 4H), 0.92 (t, *J* = 7.3 Hz, 6H). MS (GC-MS, EI) Calcd for $C_{18}H_{29}NO_4$: M^+, 323. Found: *m/z* (relative intensity) 323(44) $[M]^+$, 250 (8), 222 (12), 208 (100), 148 (6), 106 (58), 94 (29) 41 (15).

^{1}H NMR (400 MHz, $CDCl_3$) δ 6.45–6.43 (m, 1H), 5.78–5.76 (m, 1H). MS (GC-MS, EI)

Calcd for $C_{18}H_{29}NO_4$: M^+, 323. Found: *m/z* (relative intensity) 323(72) $[M]^+$, 250 (16), 222 (97), 208 (100), 148 (34), 106 (99), 94 (11), 41 (21).

IV. β-Preferential Addition of Pyrrole to Electron-Deficient Alkenes in Catalytic Mode on Boron [$B(C_6F_5)_3$]: A General Procedure for Table 1

$In(ONf)_3$ (101 mg, 100 μmol) was placed in a 20 mL Schlenk tube. Under a reduced pressure of ca. 5 Pa, the tube was heated at 50 °C for 20 min, 70 °C for 20 min, 90 °C for 20 min, 120 °C for 20 min, and 150 °C for 20 min. After cooling down to room temperature (rt), the tube was filled with argon. To this were added $H_2O•B(C_6F_5)_3$ (51.2 mg, 96.6 μmol), THF (0.800 or 1.60 mL), pyrrole (**1a**) [(168 mg, 2.50 mmol) or (33.5 mg, 0.500 mmol)], electron-deficient alkene **2** (0.500 mmol), and T*i*PEDA (28.6 mg, 125 μmol), and the resulting mixture was stirred at 70 or 80 °C. After the time specified in Table 1, CH_3COOH (63 μL) and EtOAc (2 mL) were added to the mixture. After stirring at rt for 3 min, a saturated $NaHCO_3$ aqueous solution (1 mL) and EtOAc (1 mL) were added, and the aqueous phase was extracted with EtOAc (5 mL × 3). The combined organic layer was washed with brine (1 mL) and then dried over anhydrous sodium sulfate. Filtration through a cotton plug and evaporation of the solvent followed by purification gave product **3**. Unless otherwise noted, products **3** synthesized in this section were fully characterized by ^1H and $^{13}C\{^1H\}$ NMR spectroscopy and HRMS.

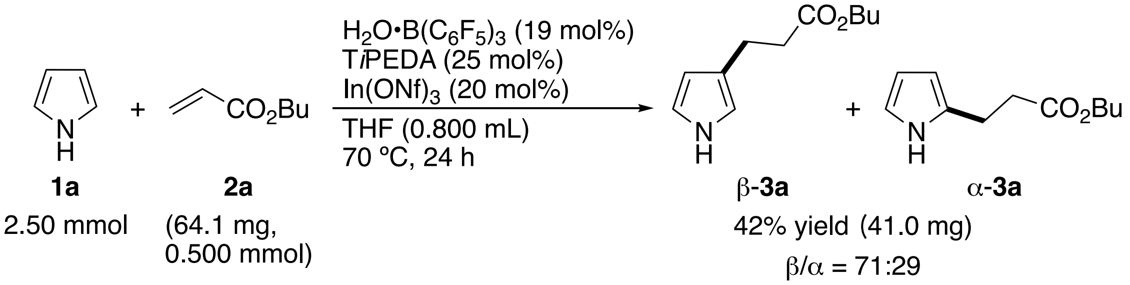

Compound β-3a. Compound β-**3a** was isolated as a colorless oil by Kugelrohr bulb-to-bulb distillation under reduced pressure (140 °C/20 Pa) after column chromatography on silica gel (hexane/EtOAc/Et_2O = 4/0.5/0.4). The Kugelrohr bulb-to-bulb distillation was performed to separate β-**3a** from **3'a** (*vide supra*). ^1H NMR (400 MHz, $CDCl_3$) δ 8.06 (br s, 1H), 6.71 (dd, *J* = 4.8, 2.5 Hz, 1H), 6.60–6.59 (m, 1H), 6.09 (dd, *J* = 4.4, 2.5 Hz, 1H), 4.08 (t, *J* = 6.6 Hz, 2H), 2.84 (t, *J* = 7.8 Hz, 2H), 2.58 (dd, *J* = 8.5, 7.1 Hz, 2H), 1.64–1.56 (m, 2H), 1.41–1.32 (m, 2H), 0.93 (t,

J = 7.3 Hz, 3H); $^{13}C\{^1H\}$ NMR (125 MHz, $CDCl_3$) δ 173.6, 122.5, 117.8, 115.1, 108.3, 64.2, 36.0, 30.7, 22.4, 19.1, 13.7. HRMS (FI) Calcd for $C_{11}H_{17}NO_2$: M, 195.1259. Found: *m/z* 195.1246.

Compound α-3a. Compound α-**3a** was isolated as a colorless oil by column chromatography on silica gel (hexane/EtOAc/Et_2O = 4/0.5/0.4). ^1H NMR (500 MHz, $CDCl_3$) δ 8.55 (br s, 1H), 6.67 (dd, J = 4.3, 2.6 Hz, 1H), 6.10 (dd, J = 6.0, 3.2 Hz, 1H), 5.93–5.90 (m, 1H), 4.10 (t, J = 6.9 Hz, 2H), 2.91 (t, J = 6.6 Hz, 2H), 2.63 (t, J = 6.6 Hz, 2H), 1.60 (quint, J = 7.2 Hz, 2H), 1.37 (sext, J = 7.4 Hz, 2H), 0.93 (t, J = 7.5 Hz, 3H); $^{13}C\{^1H\}$ NMR (125 MHz, $CDCl_3$) δ 174.3, 131.1, 116.8, 107.9, 105.4, 64.7, 34.5, 30.6, 22.5, 19.1, 13.7. HRMS (FI) Calcd for $C_{11}H_{17}NO_2$: M, 195.1259. Found: *m/z* 195.1248.

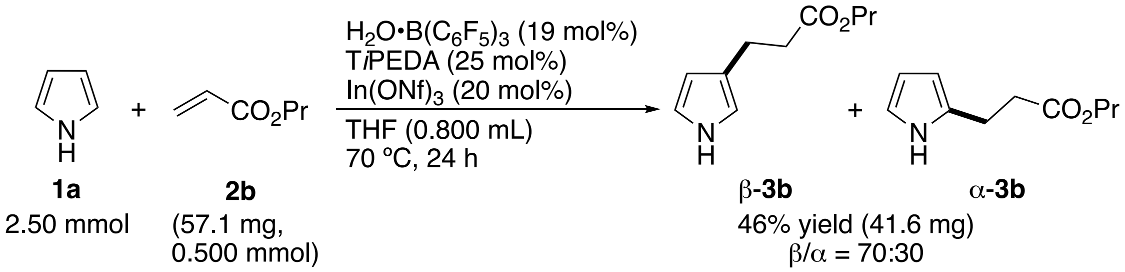

Compound β-3b. Compound β-**3b** was isolated as a colorless oil by column chromatography on silica gel (hexane/EtOAc/Et_2O = 4/0.5/0.4). ^1H NMR (500 MHz, $CDCl_3$) δ 8.01 (br s, 1H), 6.71 (dd, J = 4.6, 2.9 Hz, 1H), 6.62–6.59 (m, 1H), 6.11–6.08 (m, 1H), 4.04 (t, J = 6.6 Hz, 2H), 2.84 (t, J = 7.7 Hz, 2H), 2.59 (t, J = 8.0 Hz, 2H), 1.64 (sext, J = 7.1 Hz, 2H), 0.93 (t, J = 7.5 Hz, 3H); $^{13}C\{^1H\}$ NMR (125 MHz, $CDCl_3$) δ 173.6, 122.5, 117.8, 115.1, 108.4, 65.9, 36.0, 22.5, 22.0, 10.4. HRMS (FI) Calcd for $C_{10}H_{15}NO_2$: M, 181.1103. Found: *m/z* 181.1108.

Compound α-3b. Compound α-**3b** was isolated as a colorless oil by column chromatography on silica gel (hexane/EtOAc/Et_2O = 4/0.5/0.4). ^1H NMR (400 MHz, $CDCl_3$) δ 8.55 (br s, 1H), 6.68–6.66 (m, 1H), 6.10 (dd, J = 6.0, 2.7 Hz, 1H), 5.93–5.91 (m, 1H), 4.06 (t, J = 6.6 Hz, 2H), 2.91 (t, J = 6.9 Hz, 2H), 2.64 (t, J = 6.9 Hz, 2H), 1.65 (sext, J = 7.1 Hz, 2H), 0.93 (t, J = 7.3 Hz, 3H); $^{13}C\{^1H\}$ NMR (100 MHz, $CDCl_3$) δ 174.3, 131.1, 116.8, 108.0, 105.5, 66.4, 34.5, 22.5, 21.9, 10.4. HRMS (FI) Calcd for $C_{10}H_{15}NO_2$: M, 181.1103. Found: *m/z* 181.1106.

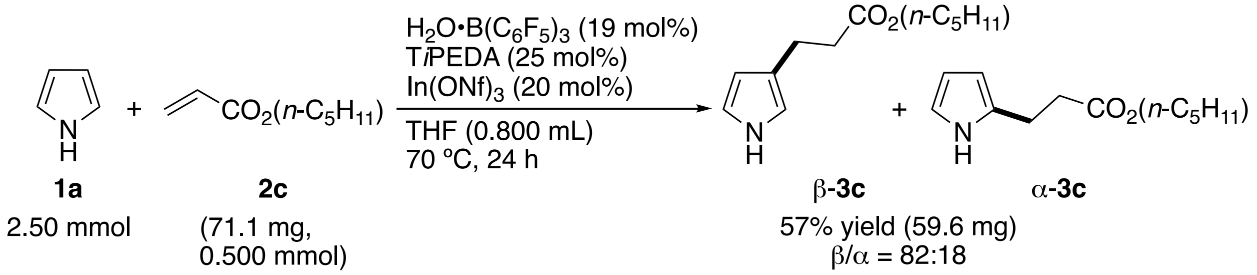

Compound β-3c. Compound β-**3c** was isolated as a colorless oil by column chromatography on silica gel (hexane/EtOAc/Et_2O = 6/1/0.1). ^{1}H NMR (400 MHz, $CDCl_3$) δ 8.02 (br s, 1H), 6.72 (dd, J = 4.8, 2.5 Hz, 1H), 6.61–6.59 (m, 1H), 6.09 (dd, J = 4.1, 2.7 Hz, 1H), 4.07 (t, J = 6.6 Hz, 2H), 2.84 (t, J = 7.8 Hz, 2H), 2.59 (dd, J = 8.5, 7.1 Hz, 1H), 1.65–1.58 (m, 2H), 1.39–1.26 (m, 4H), 0.90 (t, J = 6.9 Hz, 3H); ^{13}C{^{1}H} NMR (125 MHz, $CDCl_3$) δ 173.6, 122.6, 117.8, 115.1, 108.4, 64.5, 36.0, 28.4, 28.1, 22.5, 22.3, 14.0. HRMS (FI) Calcd for $C_{12}H_{19}NO_2$: M, 209.1416. Found: *m/z* 209.1407.

Compound α-3c. Compound α-**3c** was isolated as a colorless oil by Kugelrohr bulb-to-bulb distillation under reduced pressure (150 ºC/80 Pa) after column chromatography on silica gel (hexane/EtOAc/Et_2O = 6/1/0.1). The Kugelrohr bulb-to-bulb distillation was performed to separate α-**3c** from a regioisomeric mixture of 1/2 adducts in which **3c** further reacted with **2c**. ^{1}H NMR (500 MHz, $CDCl_3$) δ 8.54 (br s, 1H), 6.67–6.66 (m, 1H), 6.10 (dd, J = 5.7, 2.9 Hz, 1H), 5.92–5.91 (m, 1H), 4.09 (t, J = 6.9 Hz, 2H), 2.91 (t, J = 6.9 Hz, 2H), 2.64–2.62 (m, 2H), 1.62 (quint, J = 7.0 Hz, 2H), 1.37–1.27 (m, 4H), 0.90 (t, J = 7.2 Hz, 3H); ^{13}C{^{1}H} NMR (100 MHz, $CDCl_3$) δ 174.3, 131.1, 116.8, 108.0, 105.5, 64.9, 34.6, 28.3, 28.0, 22.5, 22.3, 14.0. HRMS (FD) Calcd for $C_{12}H_{19}NO_2$: M, 209.1416. Found: *m/z* 209.1419.

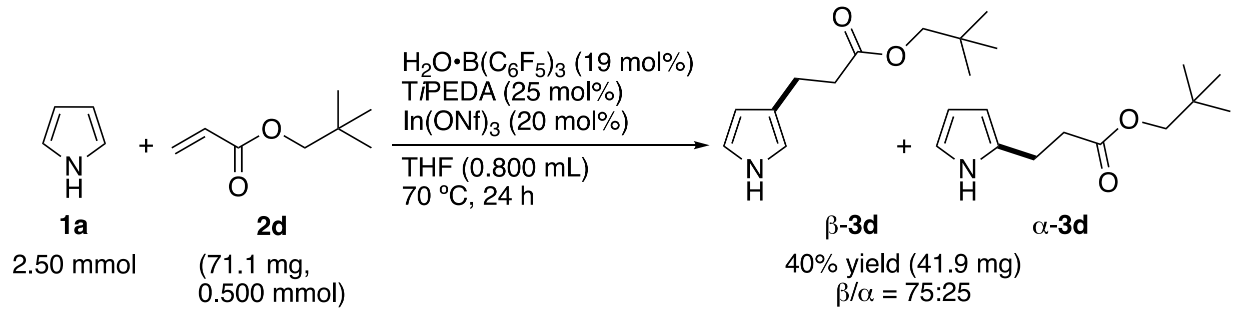

Compound β-3d. Compound β-**3d** was isolated as a colorless oil by Kugelrohr bulb-to-bulb distillation under reduced pressure (180 ºC/100 Pa) after column chromatography on silica gel (hexane/EtOAc/Et_2O = 5/0.5/0.4). The Kugelrohr bulb-to-bulb distillation was performed to

separate from a regioisomeric mixture of 1/2 adducts in which **3d** further reacted with **2d**. ^{1}H NMR (400 MHz, $CDCl_3$) δ 8.04 (br s, 1H), 6.71 (dd, *J* = 4.8, 2.5 Hz, 1H), 6.61–6.60 (m, 1H), 6.10 (dd, *J* = 4.4, 2.5 Hz, 1H), 3.77 (s, 2H), 2.86 (t, *J* = 7.8 Hz, 2H), 2,62 (dd, *J* = 8.5, 7.1 Hz, 2H), 0.92 (s, 9H); $^{13}C\{^1H\}$ NMR (125 MHz, $CDCl_3$) δ 173.6, 122.5, 117.9, 115.1, 108.4, 73.6, 36.0, 31.3, 26.4, 22.5. HRMS (FI) Calcd for $C_{12}H_{19}NO_2$: M, 209.1416. Found: *m/z* 209.1429.

Compound α-3d. Compound α-**3d** was isolated as a colorless oil by column chromatography on silica gel (hexane/EtOAc/Et_2O = 5/0.5/0.4). ^{1}H NMR (400 MHz, $CDCl_3$) δ 8.54 (br s, 1H), 6.67 (dd, *J* = 4.1, 2.7 Hz, 1H), 6.10 (dd, *J* = 5.5, 2.7 Hz, 1H), 5.93–5.91 (m, 1H), 3.80 (s, 2H), 2.93 (t, *J* = 6.9 Hz, 2H), 2.67 (t, *J* = 6.9 Hz, 2H), 0.92 (s, 9H); $^{13}C\{^1H\}$ NMR (100 MHz, $CDCl_3$) δ 174.3, 131.1, 116.8, 108.0, 105.5, 74.1, 34.5, 31.3, 26.4, 22.6. HRMS (FI) Calcd for $C_{12}H_{19}NO_2$: M, 209.1416. Found: *m/z* 209.1423.

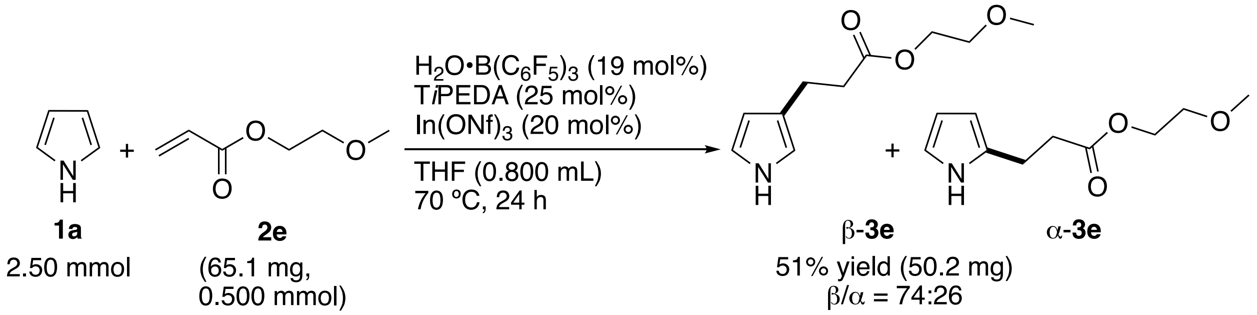

Compound β-3e. Compound β-**3e** was isolated as a colorless oil by recycling GPC after column chromatography on silica gel twice (first: hexane/EtOAc/Et_2O = 10/5/1; second: hexane/EtOAc = 2/1). ^{1}H NMR (500 MHz, $CDCl_3$) δ 8.08 (br s, 1H), 6.71 (dd, *J* = 4.9, 2.6 Hz, 1H), 6.61–6.60 (m, 1H), 6.09 (dd, *J* = 4.3, 2.6 Hz, 1H), 4.24 (t, *J* = 4.6 Hz, 2H), 3.59 (t, *J* = 4.6 Hz, 2H), 3.39 (s, 3H), 2.85 (t, *J* = 7.7 Hz, 2H), 2.64 (t, *J* = 7.7 Hz, 2H); $^{13}C\{^1H\}$ NMR (125 MHz, $CDCl_3$) δ 173.5, 122.3, 117.8, 115.1, 108.3, 70.5, 63.4, 59.0, 35.7, 22.3. HRMS (FI) Calcd for $C_{10}H_{15}NO_3$: M, 197.1052. Found: *m/z* 197.1042.

Compound α-3e. Compound α-**3e** was isolated as a colorless oil by recycling GPC after column chromatography on silica gel twice (first: hexane/EtOAc/Et_2O = 10/5/1; second: hexane/EtOAc = 2/1). ^{1}H NMR (400 MHz, $CDCl_3$) δ 8.76 (br s, 1H), 6.66 (dd, *J* = 3.9, 2.5 Hz, 1H), 6.09 (dd, *J* = 5.5, 2.7 Hz, 1H), 5.92–5.91 (m, 1H), 4.28 (t, *J* = 4.6 Hz, 2H), 3.62 (t, *J* = 4.6 Hz, 2H), 3.41 (s, 3H), 2.93 (t, *J* = 6.6 Hz, 2H), 2.67 (t, *J* = 6.6 Hz, 2H); $^{13}C\{^1H\}$ NMR (125 MHz, $CDCl_3$) δ 173.7, 130.9, 116.9, 107.9, 105.7, 70.4, 63.5, 59.0, 34.9, 22.6. HRMS (FI) Calcd for $C_{10}H_{15}NO_3$: M, 197.1052. Found: *m/z* 197.1043.

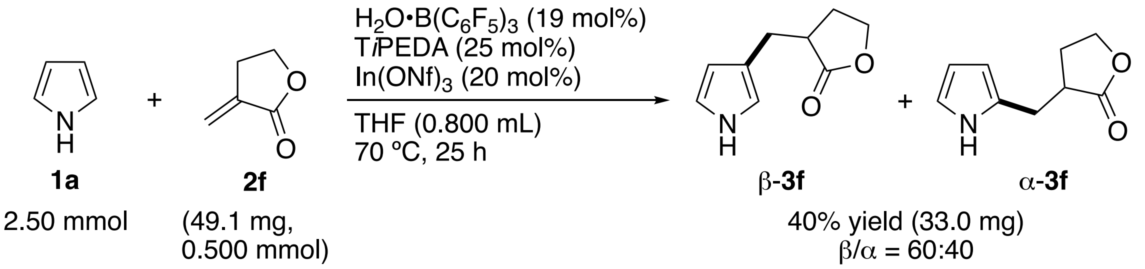

Compound β-3f. Compound **β-3f** was isolated as a colorless oil by column chromatography on silica gel twice (first: hexane/EtOAc/Et_2O = 5/5/1; second: hexane/EtOAc = 2/1). ^{1}H NMR (500 MHz, $CDCl_3$) δ 8.09 (br s, 1H), 6.73 (dd, J = 4.9, 2.6 Hz, 1H), 6.64 (dd, J = 3.7, 2.0 Hz, 1H), 6.09 (dd, J = 4.0, 2.9 Hz, 1H), 4.21–4.12 (m, 2H), 3.05–2.98 (m, 1H), 2.82–2.75 (m, 2H), 2.35–2.28 (m, 1H), 2.10–2.02 (m, 1H); ^{13}C{^{1}H} NMR (125 MHz, $CDCl_3$) δ 179.4, 119.8, 118.1, 116.2, 109.1, 66.6, 41.0, 27.9, 27.5. HRMS (FD) Calcd for $C_9H_{11}NO_2$: M, 165.0790. Found: *m/z* 165.0796.

Compound α-3f. Compound **α-3f** was isolated as a colorless oil by column chromatography on silica gel twice (first: hexane/EtOAc/Et_2O = 5/5/1; second: hexane/EtOAc = 2/1). ^{1}H NMR (500 MHz, $CDCl_3$) δ 8.70 (br s, 1H), 6.71–6.69 (m, 1H), 6.10 (dd, J = 5.7, 2.9 Hz, 1H), 5.96–5.95 (m, 1H), 4.26 (td, J = 8.7, 2.1 Hz, 1H), 4.21–4.16 (m, 1H), 3.05 (dd, J = 15.2, 4.9 Hz, 1H), 2.96 (dd, J = 14.9, 6.3 Hz, 1H), 2.88–2.82 (m, 1H), 2.39–2.33 (m, 1H), 2.13–2.04 (m, 1H); ^{13}C{^{1}H} NMR (125 MHz, $CDCl_3$) δ 179.4, 119.8, 118.1, 116.2, 109.1, 66.6, 41.0, 27.9, 27.5. HRMS (FI) Calcd for $C_9H_{11}NO_2$: M, 165.0790. Found: *m/z* 165.0787.

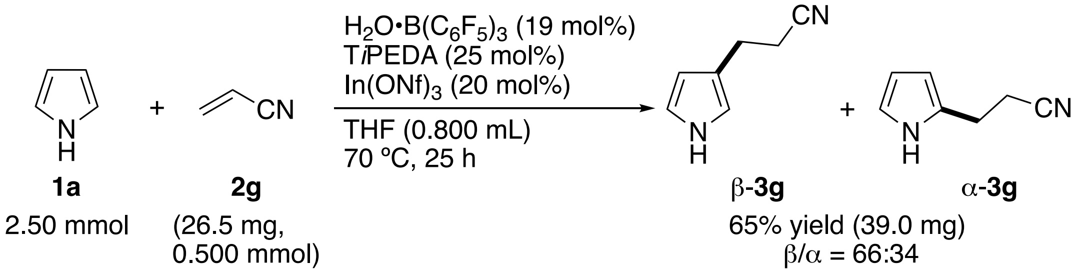

This reaction was also carried out on a 10-fold larger scale (5.00 mmol), and the reaction scheme is shown below.

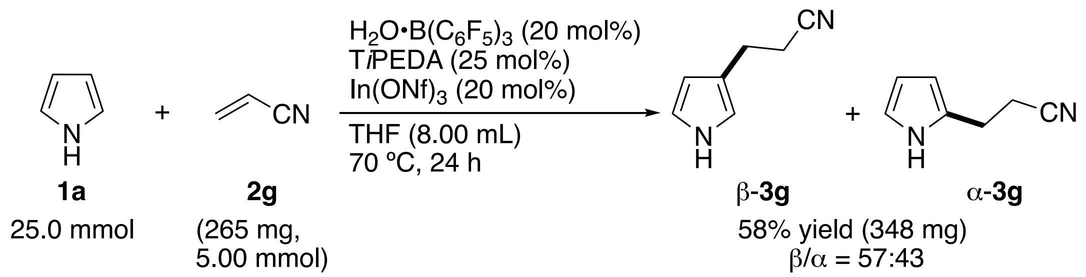

Compound β-3g. Compound β-**3g** was isolated as a colorless oil by column chromatography on silica gel twice (first: hexane/EtOAc/Et_2O = 20/10/1; second: hexane/EtOAc = 2.5/1). ^1H NMR (500 MHz, $CDCl_3$) δ 8.12 (br s, 1H), 6.75 (dd, J = 5.0, 2.4 Hz, 1H), 6.71–6.70 (m, 1H), 6.13 (dd, J = 4.4, 2.4 Hz, 1H), 2.86 (t, J = 7.2 Hz, 2H), 2.57 (t, J = 7.2 Hz, 2H); $^{13}C\{^1H\}$ NMR (125 MHz, $CDCl_3$) δ 120.3, 119.9, 118.4, 115.6, 108.1, 23.3, 19.6. HRMS (FI) Calcd for $C_7H_8N_2$: M, 120.0688. Found: *m/z* 120.0660.

Compound α-3g. Compound α-**3g** was isolated as a colorless oil by column chromatography on silica gel three times (first: hexane/EtOAc/Et_2O = 20/10/1; second: hexane/EtOAc = 2.5/1, third: hexane/EtOAc/Et_2O = 4/1/1). ^1H NMR (400 MHz, $CDCl_3$) δ 8.15 (br s, 1H), 6.73 (dd, J = 4.1, 2.3 Hz, 1H), 6.16 (dd, J = 5.7, 3.0 Hz, 1H), 6.04–6.02 (m, 1H), 2.98 (t, J = 7.1 Hz, 2H), 2.63 (t, J = 7.1 Hz, 2H); $^{13}C\{^1H\}$ NMR (125 MHz, $CDCl_3$) δ 128.1, 119.6, 117.7, 108.7, 106.4, 24.0, 18.4. HRMS (FI) Calcd for $C_7H_8N_2$: M, 120.0688. Found: *m/z* 120.0700.

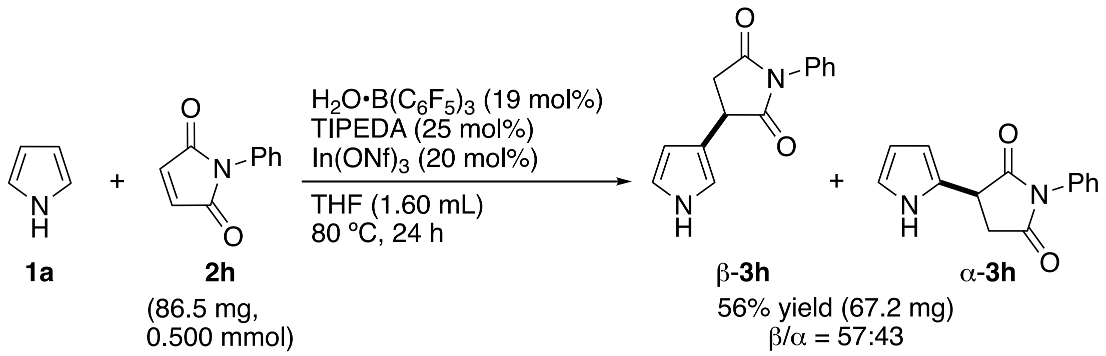

Compound β-3h. Compound β-**3h** was isolated as a colorless oil by column chromatography on silica gel (hexane/EtOAc = 2/1). ^1H NMR (400 MHz, $CDCl_3$) δ 8.31 (br s, 1H), 7.49–7.45 (m, 2H), 7.39 (tt, J = 7.3, 1.7 Hz, 1H), 7.32–7.29 (m, 2H), 6.86–6.82 (m, 1H), 6.81–6.77 (m, 1H), 6.23 (dd, J = 4.1, 2.7 Hz, 1H), 4.18 (dd, J = 9.6, 4.6 Hz, 1H), 3.33 (dd, J = 18.3, 9.2 Hz, 1H), 3.00 (dd, J = 18.3, 4.6 Hz, 1H); $^{13}C\{^1H\}$ NMR (100 MHz, $CDCl_3$) δ 177.5,

175.6, 132.1, 129.2, 128.6, 126.5, 119.0, 118.9, 115.8, 107.0, 38.9, 36.9. HRMS (FD) Calcd for $C_{14}H_{12}N_2O_2$: M, 240.0899. Found: *m/z* 240.0905.

Compound α-3h. Compound α-**3h** was isolated as a colorless oil by column chromatography on silica gel (hexane/EtOAc = 2/1). Compound α-**3h** has already appeared in the literature,[10] and its spectral and analytical data are in good agreement with those reported. Accordingly, only ^{1}H NMR data are provided here. ^{1}H NMR (400 MHz, $CDCl_3$) δ 9.11 (br s, 1H), 7.51–7.46 (m, 2H), 7.41 (tt, *J* = 7.6, 1.7 Hz, 1H), 7.29–7.27 (m, 2H), 6.86–6.84 (m, 1H), 6.22 (dd, *J* = 6.0, 2.8 Hz, 1H), 6.11–6.09 (m, 1H), 4.27 (dd, *J* = 9.6, 5.5 Hz, 1H), 3.38 (dd, *J* = 18.1, 9.4 Hz, 1H), 3.23 (dd, *J* = 18.3, 5.5 Hz, 1H).

V. Synthesis of (*N*-Pyrrolyl)borate Complexes

The synthesis of **4b**, which is derived from pyrrole (**1a**), $H_2O•B(C_6F_5)_3$, and T*i*PEDA, is described below as a representative experimental procedure: A flame-dried 50 mL two-necked round-bottomed flask equipped with a three-way stopcock was charged with $H_2O•B(C_6F_5)_3$ (256 mg, 0.483 mmol) and was evacuated under vacuum (ca. 5 Pa) at 24 ºC for 30 min. Under an argon atmosphere, to this were added CH_2Cl_2 (5.00 mL) and allyltrimethylsilane (286 mg, 2.50 mmol). After stirring at 24 ºC for 30 min (**NOTE**: Since propene gas is generated during the reaction, the flask must not be in a closed system. Propene gas produced in situ was released through a gas outlet terminating in an oil bubbler connected to the three-way stopcock.), **1a** (33.5 mg, 0.500 mmol) was added to the mixture, and the resulting solution was stirred at 24 ºC for 2 h. To this was successively added T*i*PEDA (114 mg, 0.500 mmol), and the mixture was stirred at 24 ºC for 1 h. Filtration and evaporation of the solvent followed by recrystallization from CH_2Cl_2/hexane upon cooling to rt and then –20 ºC gave **4b** in 91% yield (355 mg) as a white solid (mp 147–149 °C).

Products **4** prepared in this section were fully characterized by ^{1}H, ^{13}C{^{1}H}, ^{19}F and ^{11}B{^{1}H} NMR spectroscopy, as well as LC-MS and HRMS. Molecular ion peaks of (*N*-pyrrolyl)borate complexes **4** were not directly detected in HRMS analyses using FD and EI methods. However, exact masses of their constituent species, namely boryl anions and ammonium cations, were successfully detected, respectively. Moreover, exact masses of boryl anion species with H^+ instead

[10] M. Avalos, R. Babiano, J. L. Bravo, P. Cintas, J. L. Jiménez, J. C. Palacios, M. A. Silva, *Green Chem.* **2001**, *3*, 26–29.

of ammonium cations were observed in the FD method of the HRMS analysis.

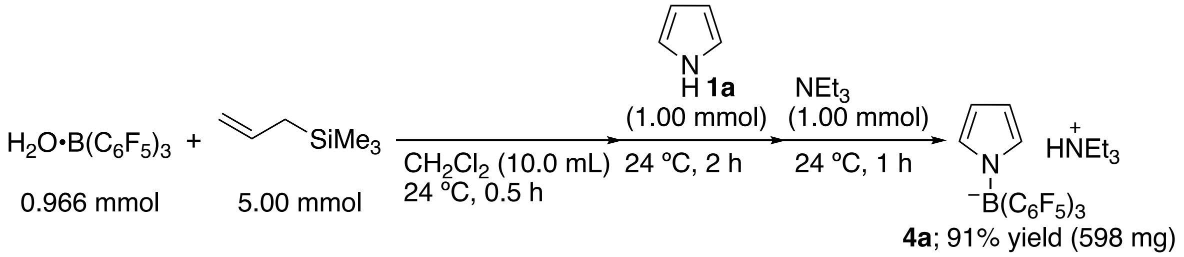

(*N*-Pyrrolyl)borate Complex 4a. (*N*-Pyrrolyl)borate complex **4a** was isolated in 91% yield (598 mg) as a white solid (mp 138–140 °C) by recrystallization from CH_2Cl_2/hexane. ^{1}H NMR (500 MHz, CD_2Cl_2) δ 6.78–6.73 (m, 2H), 5.93–5.92 (m, 2H), 3.03 (q, *J* = 7.3 Hz, 6H), 1.25 (t, *J* = 7.7 Hz, 9H) (The signal of *H*–N^+ was not observed); $^{13}C\{^1H\}$ NMR (125 MHz, CD_2Cl_2) δ 148.4 (dm, *J* = 239 Hz, *o*-C in –C_6F_5), 139.1 (dm, *J* = 254 Hz, *p*-C in –C_6F_5), 137.1 (dm, *J* = 250 Hz, *m*-C in –C_6F_5), 127.1, 123.5 (br, *ipso*-C in –C_6F_5), 104.9, 47.5, 8.9; ^{19}F NMR (376 MHz, CD_2Cl_2) δ –132.8 (d, *J* = 16.2 Hz, 6F, *o*-F), –161.8 (t, *J* = 20.3 Hz, 3F, *p*-F), –166.2 (t, *J* = 20.3 Hz, 6F, *m*-F); $^{11}B\{^1H\}$ NMR (128 MHz, CD_2Cl_2) δ –7.87. MS (LC-MS, ESI, positive mode): Calcd for $C_6H_{16}N^+$ (HN^+Et_3): 102. Found: *m/z* 102. MS (LC-MS, ESI, negative mode): Calcd for $C_{22}H_4BF_{15}N^-$ [$(F_5C_6)_3B^-$–(1-pyrrolyl)]: 578. Found: *m/z* 578. HRMS (FD) Calcd for $C_6H_{16}N^+$ (HN^+Et_3): 102.1283. Found: *m/z* 102.1301. Calcd for $C_{22}H_5BF_{15}N$ [$(F_5C_6)_3B^-H^+$–(1-pyrrolyl)]: 579.0276. Found: *m/z* 579.0269. HRMS (EI) Calcd for $C_{22}H_4BF_{15}N^-$ [$(F_5C_6)_3B^-$–(1-pyrrolyl)]: 578.0197. Found: *m/z* 578.0201.

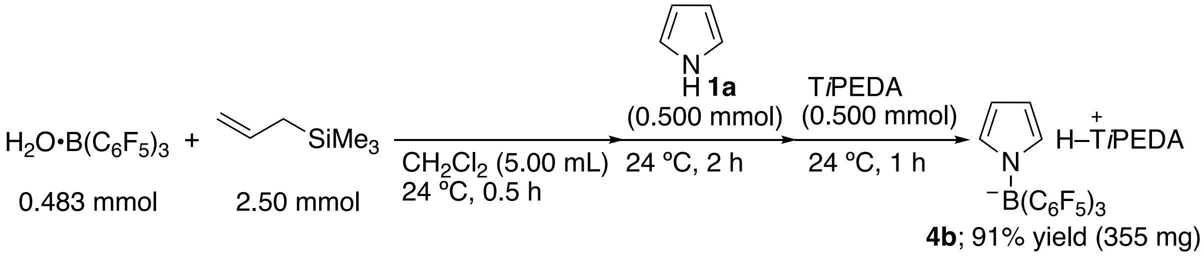

(*N*-Pyrrolyl)borate Complex 4b. ^{1}H NMR (500 MHz, CD_2Cl_2) δ 6.59–6.56 (m, 2H), 5.88 (t, *J* = 1.7 Hz, 2H), 5.36 (br s, 1H), 3.33 (sept, *J* = 6.7 Hz, 4H), 2.86 (s, 4H), 1.21 (d, *J* = 6.9 Hz, 24H); $^{13}C\{^1H\}$ NMR (125 MHz, CD_2Cl_2) δ 148.4 (dm, *J* = 239 Hz, *o*-C in –C_6F_5), 138.9 (dm, *J* = 244 Hz, *p*-C in –C_6F_5), 137.0 (dm, *J* = 242 Hz, *m*-C in –C_6F_5), 125.1, 105.8, 50.4, 41.1, 20.0; ^{19}F NMR (376 MHz, CD_2Cl_2) δ –132.4 (d, *J* = 24.4 Hz, 6F, *o*-F), –162.9 (t, *J* = 20.2 Hz, 3F, *p*-F), –166.8 (t, *J* = 20.3 Hz, 6F, *m*-F); $^{11}B\{^1H\}$ NMR (128 MHz, CD_2Cl_2) δ –8.87. MS (LC-MS, ESI,

positive mode): Calcd for $C_{14}H_{33}N_2^+$ (H–T*i*PEDA$^+$): 229. Found: *m/z* 229. MS (LC-MS, ESI, negative mode): Calcd for $C_{22}H_4BF_{15}N^-$ [$(F_5C_6)_3B^-$–(1-pyrrolyl)]: 578. Found: *m/z* 578. HRMS (FD) Calcd for $C_{14}H_{33}N_2^+$ (H–T*i*PEDA$^+$): 229.2644. Found: *m/z* 229.2632. Calcd for $C_{22}H_5BF_{15}N$ [$(F_5C_6)_3B^-H^+$–(1-pyrrolyl)]: 579.0276. Found: *m/z* 579.0281. HRMS (EI) Calcd for $C_{22}H_4BF_{15}N^-$ [$(F_5C_6)_3B^-$–(1-pyrrolyl)]: 578.0197. Found: *m/z* 578.0200.

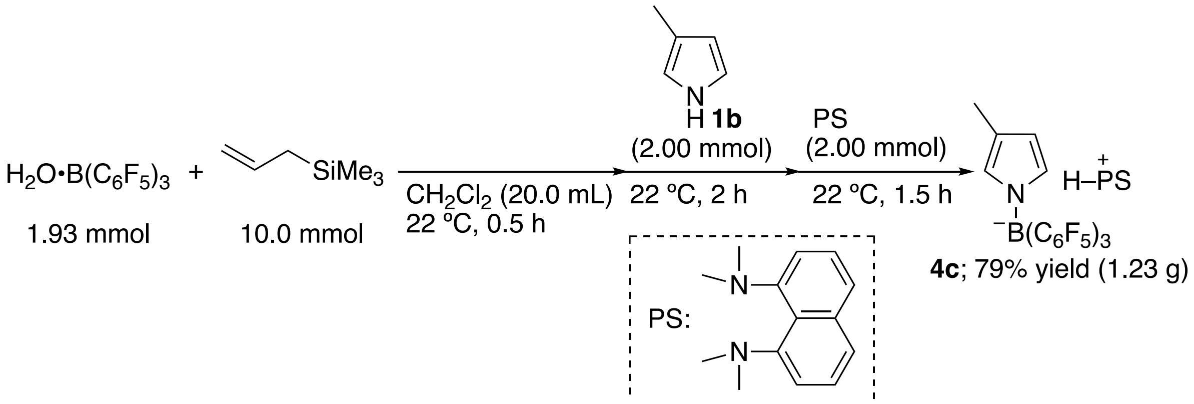

(*N*-Pyrrolyl)borate Complex 4c. (*N*-Pyrrolyl)borate complex **4c** was isolated in 79% yield (1.23 g) as an off white solid (mp 169–171 °C) by recrystallization from CH_2Cl_2/hexane. ^{1}H NMR (500 MHz, CD_2Cl_2) δ 19.2 (br s, 1H), 8.03 (d, *J* = 8.0 Hz, 2H), 7.73 (d, *J* = 6.9 Hz, 2H), 7.70 (t, *J* = 8.3 Hz, 2H), 6.47–6.44 (m, 1H), 6.36–6.33 (m, 1H), 5.69–5.67 (m, 1H), 3.09 (d, *J* = 2.3 Hz, 12H), 1.98 (s, 3H); $^{13}C\{^1H\}$ NMR (125 MHz, CD_2Cl_2) δ 148.4 (dm, *J* = 239 Hz, *o*-C in –C_6F_5), 143.5, 138.9 (dm, *J* = 245 Hz, *p*-C in –C_6F_5), 137.0 (dm, *J* = 233 Hz, *m*-C in –C_6F_5), 136.1, 130.5, 127.7, 125.1, 123.4, 121.5, 119.1, 118.8, 116.4, 116.0, 107.5, 46.8, 12.5; ^{19}F NMR (471 MHz, CD_2Cl_2) δ –132.3 (d, *J* = 20.6 Hz, 6F, *o*-F), –162.9 (t, *J* = 20.6 Hz, 3F, *p*-F), –166.7 to –166.8 (m, 6F, *m*-F); $^{11}B\{^1H\}$ NMR (128 MHz, CD_2Cl_2) δ –8.17. MS (LC-MS, ESI, positive mode): Calcd for $C_{14}H_{19}N_2^+$ (H–PS$^+$): 215. Found: *m/z* 215. MS (LC-MS, ESI, negative mode): Calcd for $C_{23}H_6BF_{15}N^-$ [$(F_5C_6)_3B^-$–(3-methyl-1-pyrrolyl)]: 592. Found: *m/z* 592. HRMS (FD) Calcd for $C_{14}H_{19}N_2^+$ (H–PS$^+$): 215.1548. Found: *m/z* 215.1576. Calcd for $C_{23}H_7BF_{15}N$ [$(F_5C_6)_3B^-H^+$–(3-methyl-1-pyrrolyl)]: 593.0432. Found: *m/z* 593.0440. HRMS (EI) Calcd for $C_{23}H_6BF_{15}N^-$ [$(F_5C_6)_3B^-$–(3-methyl-1-pyrrolyl)]: 592.0354. Found: *m/z* 592.0374.

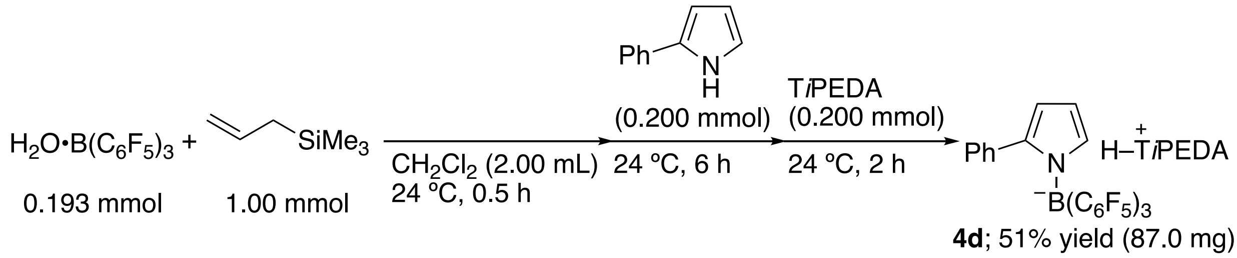

(*N*-Pyrrolyl)borate Complex 4d. (*N*-Pyrrolyl)borate complex **4d** was isolated in 51% yield (87.0 mg) as a light brown solid (mp 150–152 °C) by recrystallization from chlorobenzene/hexane. Although the brown solid was finally dried up in vacuo (ca. 5 Pa) at 60 ºC for 12 h, 7% of chlorobenzene and 2% of hexane, which were estimated by ^{1}H NMR spectroscopy, could not be removed from the solid. This solid including the volatiles was used for the characterization and for the stoichiometric reaction on $B(C_6F_5)_3$ (see section **VIII**). ^{1}H NMR (400 MHz, CD_2Cl_2) δ 6.99–6.90 (m, 5H), 6.81–6.76 (m, 1H), 5.98–5.97 (m, 1H), 5.91–5.89 (m, 1H), 3.33 (sept, J = 6.7 Hz, 4H), 2.88 (s, 4H), 1.22 (d, J = 6.7 Hz, 24H); ^{13}C{^{1}H} NMR (100 MHz, CD_2Cl_2) δ 148.8 (dm, J = 241 Hz, *o*-C in $–C_6F_5$), 140.5–137.5 (m, *p*-C in $–C_6F_5$), 139.0, 138.7, 138.2–135.1 (m, *m*-C in $–C_6F_5$), 129.6, 128.2, 127.0, 125.4, 110.8, 105.2, 50.4, 41.2, 19.9; Due likely to the influence of the Ph group, the three C_6F_5 groups were observed in a 1/2 ratio as distinct signals in ^{19}F NMR spectroscopy: ^{19}F NMR (376 MHz, CD_2Cl_2) δ –125.4 (d, J = 24 Hz, 2F, *o*-F), –133.6 (d, J = 24 Hz, 4F, *o*-F), –161.6 (t, J = 20 Hz, 1F, *p*-F), –163.4 (t, J = 20 Hz, 2F, *p*-F), –166.0 (t, J = 24 Hz, 2F, *m*-F), –168.4 (t, J = 24 Hz, 4F, *m*-F); ^{11}B{^{1}H} NMR (128 MHz, CD_2Cl_2) δ –8.14. MS (LC-MS, ESI, positive mode): Calcd for $C_{14}H_{33}N_2^+$ (H–T*i*PEDA$^+$): 229. Found: *m/z* 229. MS (LC-MS, ESI, negative mode): Calcd for $C_{28}H_8BF_{15}N^-$ [$(F_5C_6)_3B^-$–(2-phenyl-1-pyrrolyl)]: 654. Found: *m/z* 654. HRMS (FD) Calcd for $C_{14}H_{33}N_2^+$ (H–T*i*PEDA$^+$): 229.2644. Found: *m/z* 229.2642. Calcd for $C_{28}H_9BF_{15}N$ [$(F_5C_6)_3B^-H^+$–(2-phenyl-1-pyrrolyl)]: 655.0589. Found: *m/z* 655.0608. HRMS (EI) Calcd for $C_{28}H_8BF_{15}N^-$ [$(F_5C_6)_3B^-$–(2-phenyl-1-pyrrolyl)]: 654.0510. Found: *m/z* 654.0532.

VI. Single-Crystal X-ray Diffraction Analysis of (*N*-Pyrrolyl)borate Complex 4b

The single-crystal X-ray diffraction data of **4b** (CCDC 2370669) were collected on a Rigaku MicroMax-007HFM MoKα (λ = 0.71070 Å) rotating anode X-ray generator equipped with VariMax optics, an AFC10 goniometer, and a Saturn 724+ detector. The reflection data for **4b** were integrated, scaled, and averaged using the CrysAlis Pro program package. The structures

were solved by a direct method (ShelXT) and refined by full-matrix least-squares methods on F^2 for all reflections (ShelXL). The non-hydrogen atoms were refined anisotropically and hydrogen atoms were placed at idealized positions and refined using the riding model. All calculations were performed using the program Olex2, and illustrations were drawn using Mercury.

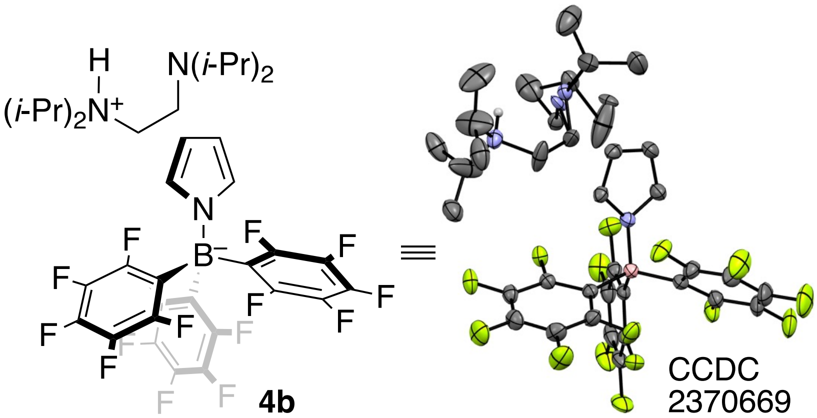

4b

CCDC 2370669

Empirical formula	$C_{36}H_{37}BF_{15}N_3$	
Formula weight	807.50 g/mol	
Temperature	123 K	
Wavelength	0.71073 Å	
Crystal system	monoclinic	
Space group	$P2_1/c$	
Unit cell dimensions	a = 9.1801(4) Å	α = 90°
	b = 25.8209(13) Å	β = 93.512(3)°
	c = 15.4554(5) Å	γ = 90°
Volume	3656.6(3) $Å^3$	
Z	4	
Density (ρ) (calculated)	1.467 g/cm^3	
Absorption coefficient (μ)	0.139 mm^{-1}	
F(000)	1656.0	
Crystal size	0.2 x 0.2 x 0.05 mm^3	
Theta range for data collection	1.538 to 31.219°	
Index ranges	–13 ≤ h ≤ 13, –27 ≤ k ≤ 36, –22 ≤ l ≤ 11	
Data/restraints/parameters	10490/0/543	
Goodness-of-fit on F^2	1.047	
Final R indexes [I≥2σ (I)]	R_1 = 0.0898, wR_2 = 0.2565	
Largest diff. peak and hole	1.181 and –1.290 e.$Å^{-3}$	

VII. Examination of Suitable Reaction Conditions for β-Selective Addition of (*N*-Pyrrolyl)borate Complex 4b to Butyl Acrylate: Stoichiometric Mode on $B(C_6F_5)_3$

In this section, the promising results in each table are highlighted with a gray background.

After performing the stoichiometric reaction on $B(C_6F_5)_3$, the formation of a considerable amount of **17b**, which still has the N–B bond, in addition to **3a** was observed in a crude reaction mixture. Accordingly, the appropriate choice of quenching agents is significant to efficiently collect **3a** after the reaction. The results of investigating the effect of quenching agents are summarized in **Table S9**.

Table S9. Effect of quenching agents.[a]

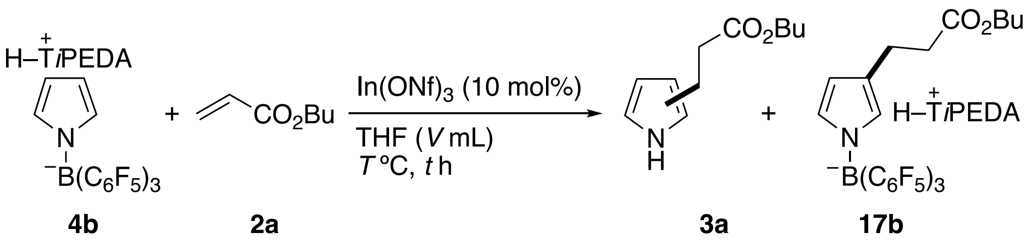

entry	*quenching agent*	*V*	*T*	*t*	conv. (%) of **2a**[b]	yield (%) of **3a**[c] (β/α)[b]	yield (%) of **17b**[c]
1	0.1 N HCl aq.	0.60	40	18	92	30 (95:5)	<1
2	sat. NH_4Cl aq.	0.60	40	18	77	12 (>99:1)	15
3	CH_3CO_2H	0.60	40	18	83	30 (>99:1)	<1
4	CH_3CO_2H then NEt_3	1.20	30	42	67	30 (>99:1)	18
5	$HSiPh_2Me$	1.20	30	42	58	23 (>99:1)	19
6	$HSiEt_3$	1.20	30	42	64	24 (>99:1)	18
7	**4.5 M KHF_2 aq.**	**1.20**	**30**	**42**	**61**	**47 (>99:1)**	**<1**

[a]Reagents: **4b** (0.150 mmol), **2a** (0.100 mmol), $In(ONf)_3$ (10.0 μmol), THF (0.600 mL or 1.20 mL). [b]Determined by GC. [c]Determined by NMR.

The respective work-up procedures for entries 1–7 of **Table S9** are summarized in **Figure S1**.

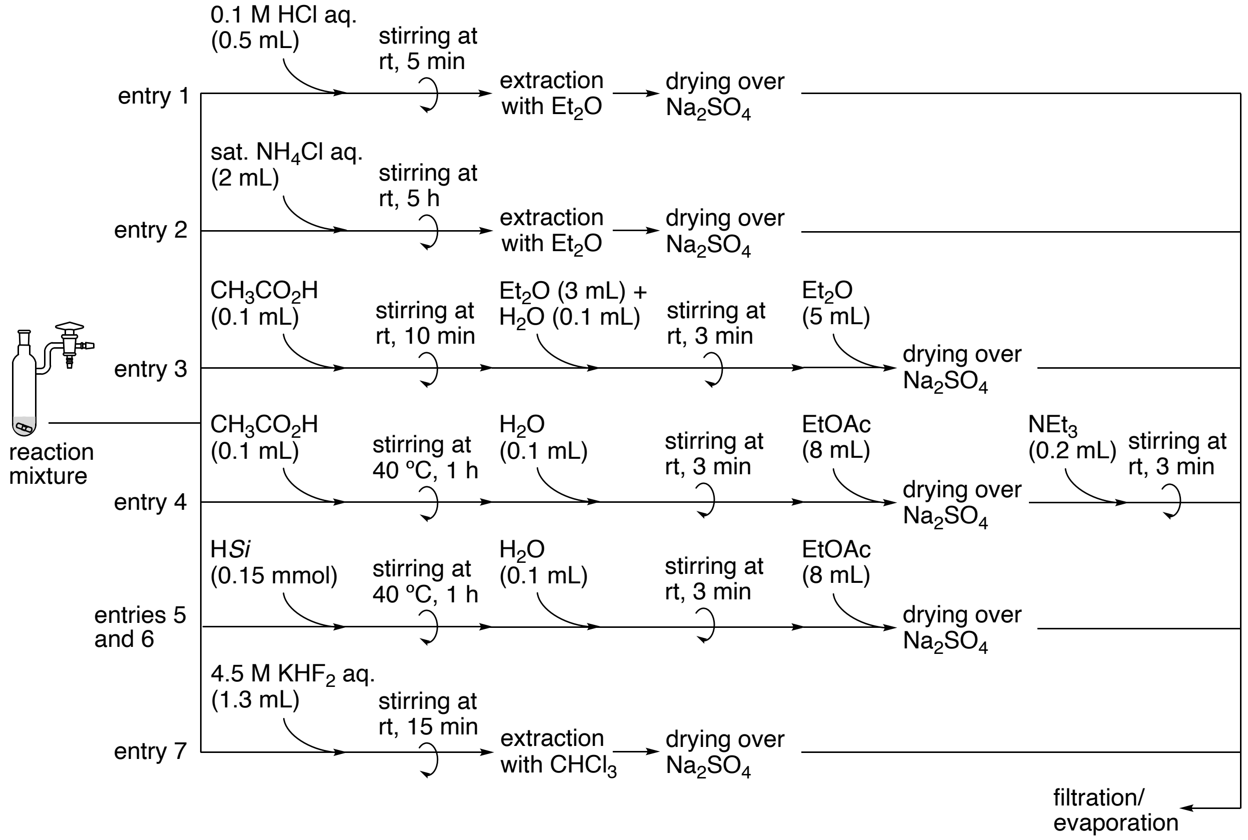

Figure S1. Work-up procedures for entries 1–7 of **Table S9**.

The ^{1}H NMR spectrum regarding the H$^{\alpha}$ and H$^{\beta}$ signals of (*N*-pyrrolyl)borate complex **17b** possibly observed in the crude reaction mixture after performing the reaction of **4b** with **2a** is given in **Figure S2**-[a]. The ^{1}H NMR spectra regarding the proton signals of other related compounds confirmed in the crude reaction mixture are also provided as reference information. **Figure S2** shows that (*N*-pyrrolyl)borate complexes **17b** and **4b** have the good stability that can survive under the reaction conditions, while they partly decomposed into β-**3a** and pyrrole (**1a**), respectively, during the reaction. These NMR spectra indicate that the H$^{\alpha}$ and H$^{\beta}$ signals of **17b** as well as the proton signals of **4b** disappear after treating the crude reaction mixture with a 4.5 M KHF_2 aqueous solution.

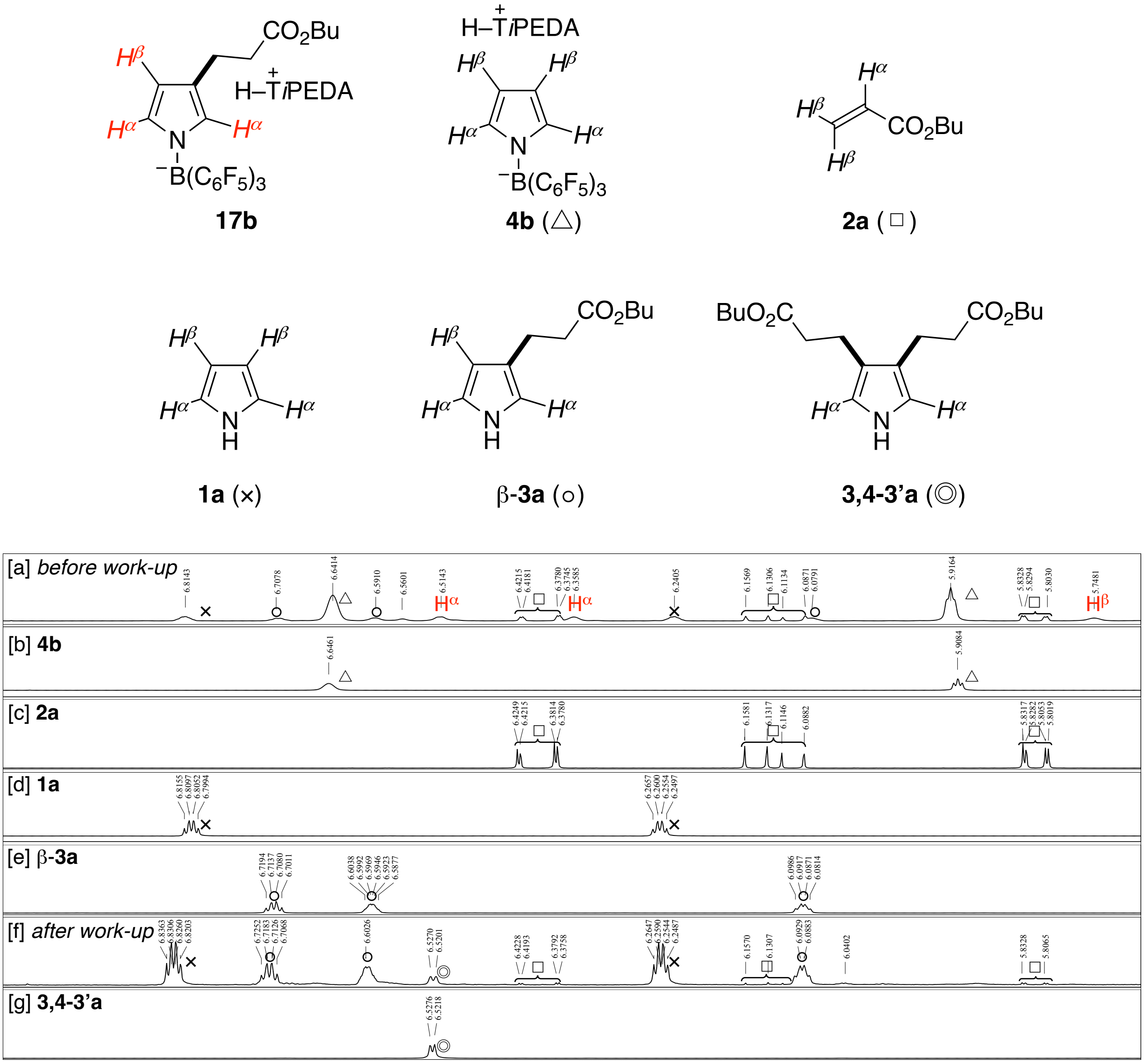

Figure S2. ^{1}H NMR spectra in $CDCl_3$: [a] a crude reaction mixture before work-up; [b] **4b** (△); [c] **2a** (□); [d] **1a** (×); [e] β-**3a** (○); [f] a crude reaction mixture after work-up including the treatment by a 4.5 M KHF_2 aqueous solution; [g] **3,4-3'a** (◎).

Based on the results of **Table S9**, a 4.5 M KHF_2 aqueous solution was used as a quenching agent in the following investigations.

Table S10. Effect of Lewis acids[a]

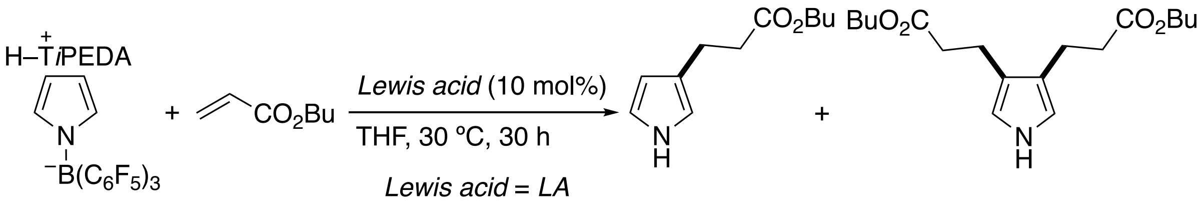

4b **2a** β-**3a** **3,4-3'a**

LA	conv. (%) of **2a**[b]	yield (%) of **3a**[c] (β/α)[b]	yield (%) of **3,4-3'a**[c]
**In(ONf)$_3$**	**53**	**52 (>99:1)**	**9**
In(OTf)$_3$	47	34 (>99:1)	6
In(NTf$_2$)$_3$	35	27 (>99:1)	3
InBr$_3$	4	<1	<1
Sc(OTf)$_3$	51	31 (>99:1)	4
Y(OTf)$_3$	21	13 (98:2)	<1
Nd(OTf)$_3$	3	9 (>99:1)	<1
Sm(OTf)$_3$	6	5 (98:2)	<1
Yb(OTf)$_3$	52	29 (98:2)	<1
Cu(OTf)$_2$	<1	<1	<1
Zn(OTf)$_2$	26	15 (99:1)	<1
Bi(OTf)$_3$	53	38 (>99:1)	4
Zn(ONf)$_2$	21	13 (>99:1)	<1
Sc(ONf)$_3$	56	43 (>99:1)	6
H_2O•B(C$_6$F$_5$)$_3$	8	<1	<1
effect of a catalytic amount of In(ONf)$_3$			
In(ONf)$_3$[d]	73	47 (>99:1)	12
In(ONf)$_3$[e]	82	50 (>99:1)	14

[a]Reagents: **4b** (0.150 mmol), **2a** (0.100 mmol), LA (10.0 μmol), THF (1.20 mL). [b]Determined by GC. [c]Determined by NMR. [d]In(ONf)$_3$ (20.0 μmol) was used. [e]In(ONf)$_3$ (30.0 μmol) was used.

Table S11. Effect of solvents[a]

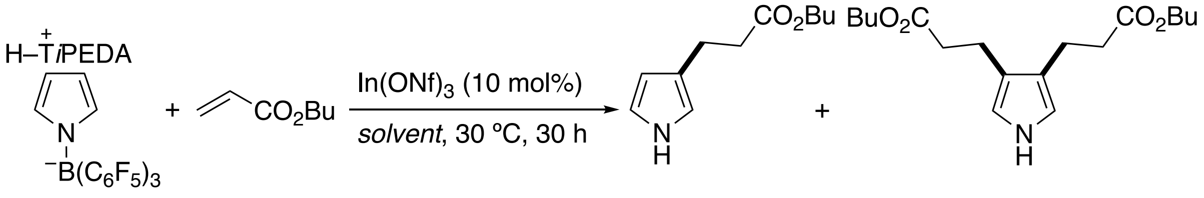

4b **2a** β-**3a** **3,4-3'a**

solvent	conv. (%) of **2a**[b]	yield (%) of **3a**[c] (β/α)[b]	yield (%) of **3,4-3'a**[c]
**THF**	**53**	**52 (>99:1)**	**9**
1,4-dioxane	4	<1	<1
DME	85	29 (98:2)	5
(CH$_2$Cl)$_2$	16	9 (93:7)	<1
PhCl	31	17 (84:16)	5
MeOAc	25	14 (95: 5)	2
EtOAc	8	8 (95: 5)	<1
PrOAc	30	17 (95: 5)	3

[a]Reagents: **4b** (0.150 mmol), **2a** (0.100 mmol), In(ONf)$_3$ (10.0 μmol), solvent (1.20 mL). [b]Determined by GC. [c]Determined by NMR. Abbreviation: DME = 1,2-dimethoxyethane; Ac = acetyl.

Table S12. Effect of an amount of solvent, temperature and reaction time[a]

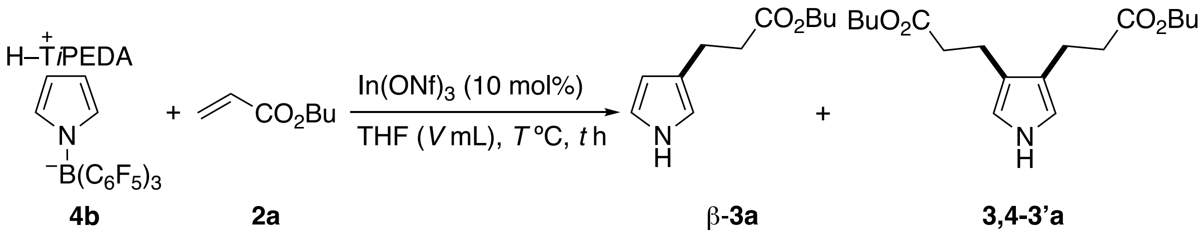

V	*T*	*t*	conv. (%) of **2a**[b]	yield (%) of **3a**[c] (β/α)[b]	yield (%) of **3,4-3'a**[c]
0.60	30	30	77	35 (99:1)	17
**1.20**	**30**	**30**	**53**	**52 (>99:1)**	**9**
1.20	30	72	83	55 (>99:1)	10
1.20	40	30	71	51 (>99:1)	12
1.60	30	30	24	23 (>99:1)	2

[a]Reagents: **4b** (0.150 mmol), **2a** (0.100 mmol), In(ONf)$_3$ (10.0 μmol), THF (0.600, 1.20 or 1.60 mL). [b]Determined by GC. [c]Determined by NMR.

VIII. β-Selective Addition of (*N*-Pyrrolyl)borate Complexes to Electron-Deficient Alkenes in Stoichiometric Mode on Boron [B(C_6F_5)$_3$]: A General Procedure for Table 2 Performed on a 0.1 mmol Scale of Electron-Deficient Alkenes

In(ONf)$_3$ (10.1 mg, 10.0 μmol) was placed in a 20 mL Schlenk tube. Under a reduced pressure of ca. 5 Pa, the tube was heated at 50 °C for 20 min, 70 °C for 20 min, 90 °C for 20 min, 120 °C for 20 min, and 150 °C for 20 min. After cooling down to room temperature (rt), the tube was filled with argon. To this was placed **4** (0.150 mmol), and the tube was successively evacuated under vacuum (ca. 5 Pa) at rt for 1 h. To this were added THF (0.600 or 1.20 mL) and electron-deficient alkene **2** (0.100 mmol), and the resulting mixture was stirred at the temperature specified in Table 2. After the time specified in Table 2, a 4.5 M KHF_2 aqueous solution (refer to the respective reaction schemes shown below for the amount used) was added, and the resulting solution was stirred for the time specified in each reaction scheme (*vide infra*) and extracted with $CHCl_3$ (5 mL × 3). The combined organic layer was washed with brine (1 mL) and then dried over anhydrous sodium sulfate. Filtration through a cotton plug and evaporation of the solvent followed by purification gave product **3**. Unless otherwise noted, products **3** synthesized in this section were fully characterized by ^{1}H and ^{13}C{^{1}H} NMR spectroscopy and HRMS.

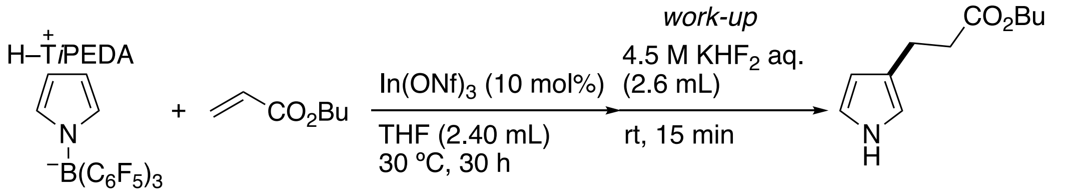

4b (242 mg, 0.300 mmol) **2a** (25.6 mg, 0.200 mmol) β-**3a** 45% yield (17.6 mg)

Compound β-3a. Compound β-**3a** was isolated as a colorless oil by Kugelrohr bulb-to-bulb distillation under reduced pressure (160 ºC/70 Pa). Compound β-**3a** already appears in Section IV of this Supporting Information. Its spectral and analytical data are thus collected there.

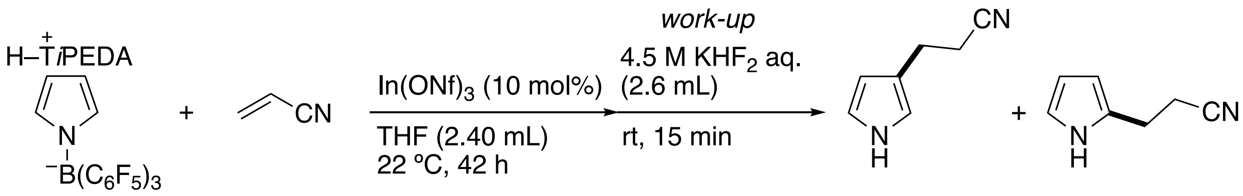

4b (242 mg, 0.300 mmol) **2g** (10.6 mg, 0.200 mmol) β-**3g** α-**3g** 99% yield (23.8 mg) β/α = 63:37

Compound 3g. A mixture of β-**3g** and α-**3g** was isolated as a colorless oil by column chromatography on silica gel (hexane/EtOAc = 5/1). Compounds β-**3g** and α-**3g** already appear in Section IV of this Supporting Information. Their spectral and analytical data are thus collected there, respectively.

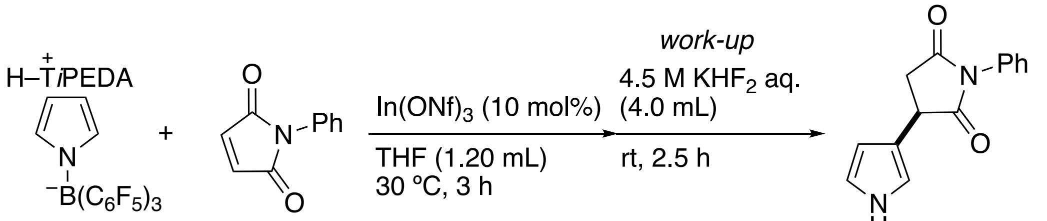

4b (121 mg, 0.150 mmol) **2h** (17.3 mg, 0.100 mmol) β-**3h** 92% yield (22.1 mg)

Compound β-3h. Compound β-**3h** was isolated as a dark yellow solid (mp 141–143 °C) by column chromatography on silica gel (hexane/EtOAc = 1.5/1). Compound β-**3h** already appears in Section IV of this Supporting Information. Its spectral and analytical data are thus collected there.

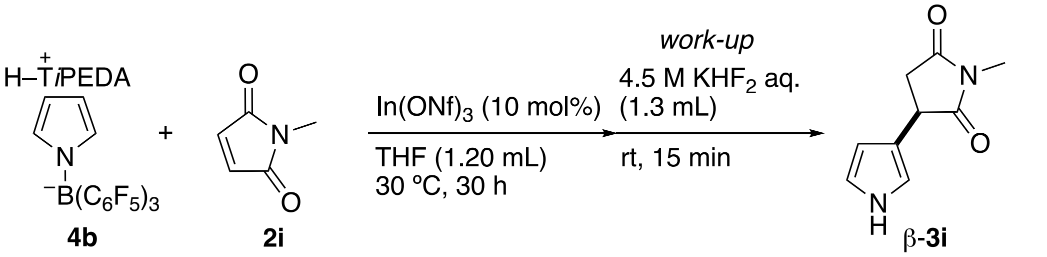

4b (121 mg, 0.150 mmol) **2i** (11.1 mg, 0.100 mmol) β-**3i** 90% yield (16.0 mg)

Compound β-3i. Compound β-**3i** was isolated as a colorless oil by column chromatography on silica gel (hexane/EtOAc = 1.5/1). ^{1}H NMR (400 MHz, CDCl$_3$) δ 8.28 (br s, 1H), 6.80–6.77 (m, 2H), 6.15 (dd, *J* = 4.4, 2.5 Hz, 1H), 4.01 (dd, *J* = 9.2, 4.6 Hz, 1H), 3.16 (dd, *J* = 18.1, 9.4 Hz, 1H), 3.03 (s, 3H), 2.82 (dd, *J* = 18.3, 4.6 Hz, 1H); ^{13}C{^{1}H} NMR (100 MHz, CDCl$_3$) δ 178.7, 176.7, 118.9, 115.8, 107.0, 38.8, 36.8, 25.0 (One carbon signal is missing due to overlapping). HRMS (FD) Calcd for $C_9H_{10}N_2O_2$: M, 178.0742. Found: *m/z* 178.0743.

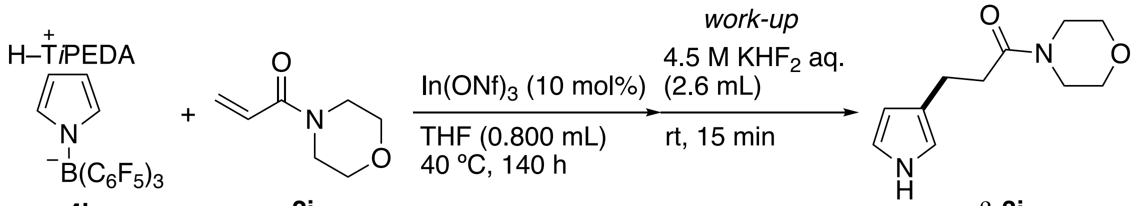

4b (242 mg, 0.300 mmol) **2j** (28.2 mg, 0.200 mmol) β-**3j** 27% NMR yield (determined using CH_3NO_2 as an internal standard)

Compound β-3j. Purification of β-**3j** was carried out by column chromatography on silica gel (hexane/EtOAc = 3/1), Kugelrohr bulb-to-bulb distillation under reduced pressure, and GPC. However, β-**3j** could not be obtained as a spectrally pure form. These results are likely to be responsible for the instability of β-**3j**. Even so, among the purification methods, the purification by GPC performed twice resulted in the recovery of β-**3j** that seemed to have the highest purity. Therefore, the structure of β-**3j** was assigned by ^{1}H and ^{13}C{^{1}H} NMR spectroscopy measured after the purification by GPC. The formation of β-**3j** was also confirmed by HRMS analysis. ^{1}H NMR (500 MHz, CD_2Cl_2) δ 8.14 (br s, 1H), 6.70 (dd, *J* = 4.6, 2.3 Hz, 1H), 6.61–6.59 (m, 1H), 6.06 (dd, *J* = 4.3, 2.6 Hz, 1H), 3.61 (t, *J* = 4.6 Hz, 2H), 3.57–3.53 (m, 4H), 3.40 (t, *J* = 4.9 Hz, 2H), 2.77 (t, *J* = 7.7 Hz, 2H), 2.55–2.52 (m, 2H); ^{13}C{^{1}H} NMR (100 MHz, CD_2Cl_2) δ 171.6, 123.2, 118.1, 115.5, 108.7, 67.2 (or 67.3), 67.0, 46.4, 42.2, 35.1, 23.1 (Two ^{13}C{^{1}H} NMR spectra are adopted in Section **XI** because two carbon signals were not observed in one of the two ^{13}C{^{1}H}

NMR spectra due to a low signal-to-noise ratio). HRMS (FD) Calcd for $C_{11}H_{16}N_2O_2$: M, 208.1212. Found: *m/z* 208.1199.

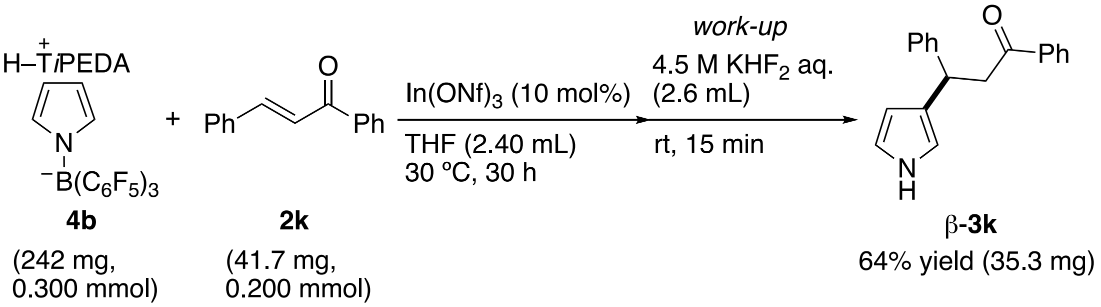

Compound β-3k. Compound β-**3k** was isolated as a pale yellow solid (mp 85–87 °C) by column chromatography on silica gel (hexane/EtOAc = 5/1). ^{1}H NMR (400 MHz, $CDCl_3$) δ 8.00 (br s, 1H), 7.93 (d, *J* = 7.3 Hz, 2H), 7.53 (t, *J* = 7.6 Hz, 1H), 7.43 (t, *J* = 7.6 Hz, 2H), 7.31 (t, *J* = 8.5 Hz, 2H), 7.27–7.25 (m, 2H), 7.16 (t, *J* = 7.1 Hz, 1H), 6.70 (dd, *J* = 4.8, 2.5 Hz, 1H), 6.53 (dd, *J* = 4.1, 2.3 Hz, 1H), 6.08 (dd, *J* = 4.4, 2.5 Hz, 1H), 4.73 (t, *J* = 7.3 Hz, 1H), 3.69 (dd, *J* = 16.5, 7.3 Hz, 1H), 3.58 (dd, *J* = 16.9, 7.3 Hz, 1H); ^{13}C{^{1}H} NMR (100 MHz, $CDCl_3$) δ 198.8, 145.4, 137.3, 132.9, 128.5, 128.4, 128.1, 127.8, 127.2, 126.1, 118.0, 115.2, 107.9, 46.0, 39.2. HRMS (FD) Calcd for $C_{19}H_{17}NO$: M, 275.1310. Found: *m/z* 275.1315.

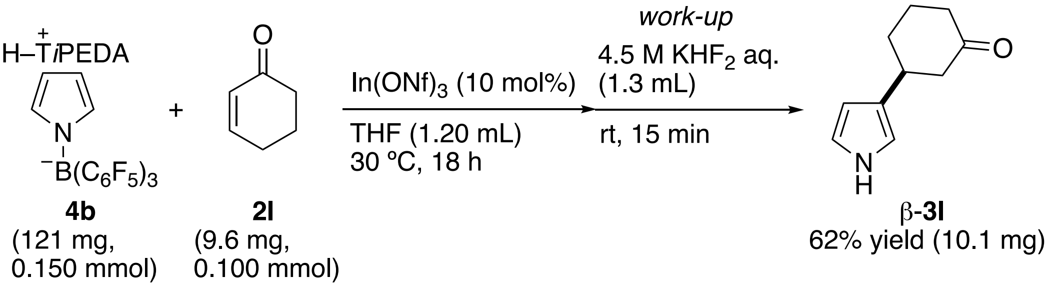

Compound β-3l. Compound β-**3l** was isolated as a colorless oil by column chromatography on silica gel (hexane/EtOAc = 2/1). ^{1}H NMR (400 MHz, $CDCl_3$) δ 8.07 (br s, 1H), 6.76 (dd, *J* = 4.8, 2.5 Hz, 1H), 6.60 (dd, *J* = 4.4, 2.5 Hz, 1H), 6.13 (dd, *J* = 4.4, 2.5 Hz, 1H), 3.10–3.02 (m, 1H), 2.67 (ddt, *J* = 14.0, 4.3, 2.0 Hz, 1H), 2.47 (ddd, *J* = 13.9, 11.5, 1.1 Hz, 1H), 2.44–2.29 (m, 2H), 2.18–2.03 (m, 2H), 1.82–1.70 (m, 2H); ^{13}C{^{1}H} NMR (100 MHz, $CDCl_3$) δ 211.9, 127.6, 118.1, 114.0, 106.8, 49.0, 41.4, 37.0, 32.8, 25.1. HRMS (FD) Calcd for $C_{10}H_{13}NO$: M, 163.0997. Found: *m/z* 163.1001.

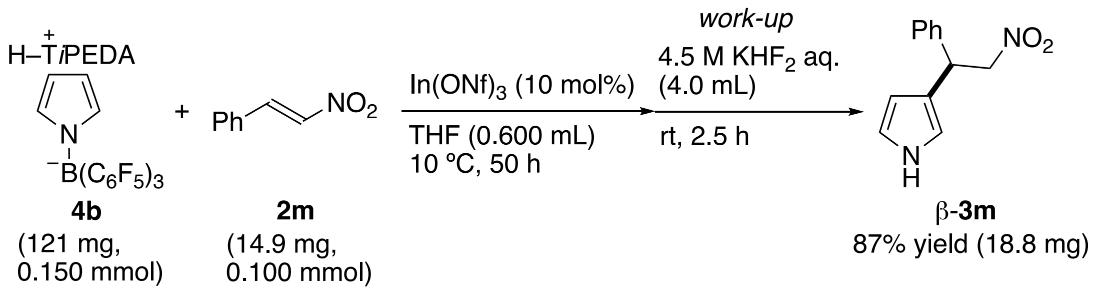

Compound β-3m. Compound β-**3m** was isolated as a dark yellow solid (mp 87–89 °C) by column chromatography on silica gel (hexane/EtOAc = 3/1). ^{1}H NMR (500 MHz, $CDCl_3$) δ 8.11 (br s, 1H), 7.34–7.24 (m, 5H), 6.76 (dd, J = 4.6, 2.9 Hz, 1H), 6.58 (dd, J = 4.0, 1.7 Hz, 1H), 6.10 (dd, J = 4.6, 2.9 Hz, 1H), 4.93–4.81 (m, 3H); $^{13}C\{^{1}H\}$ NMR (125 MHz, $CDCl_3$) δ 140.3, 128.8, 127.7, 127.3, 122.1, 118.7, 115.6, 107.5, 80.5, 42.6. HRMS (FD) Calcd for $C_{12}H_{12}N_2O_2$: M, 216.0899. Found: *m/z* 216.0921.

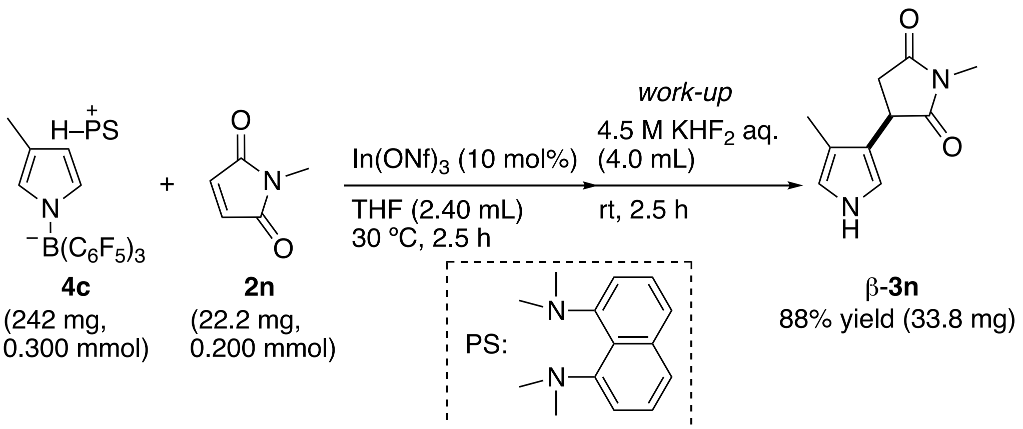

Compound β-3n. Compound β-**3n** was isolated as a pale pink solid (mp 123–125 °C) by column chromatography on silica gel (hexane/EtOAc = 2/1). ^{1}H NMR (500 MHz, $CDCl_3$) δ 7.97 (br s, 1H), 6.61 (t, J = 2.3 Hz, 1H), 6.58–6.56 (m, 1H), 4.00 (dd, J = 9.5, 4.9 Hz, 1H), 3.16 (dd, J = 18.3, 9.2 Hz, 1H), 3.05 (s, 3H), 2.72 (dd, J = 18.3, 5.2 Hz, 1H), 2.05 (s, 3H); $^{13}C\{^{1}H\}$ NMR (100 MHz, $CDCl_3$) δ 178.7, 176.7, 118.5, 117.18, 117.15, 115.5, 38.0, 37.0, 25.0, 10.4. HRMS (FD) Calcd for $C_{10}H_{12}N_2O_2$: M, 192.0899. Found: *m/z* 192.0900.

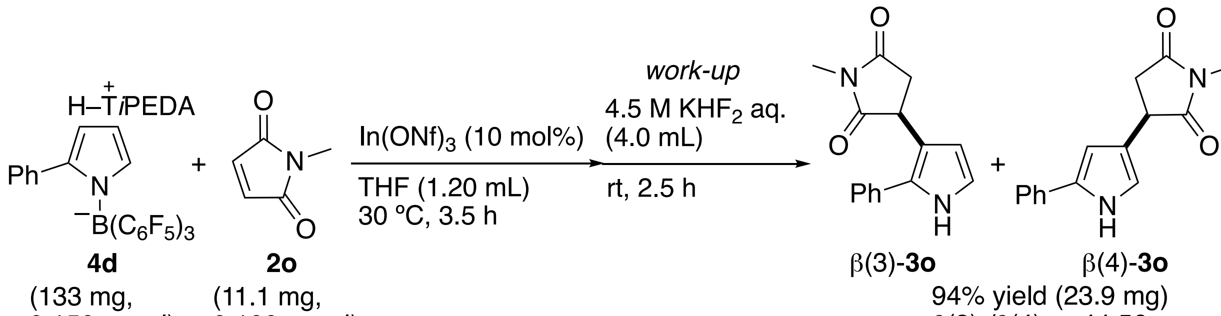

4d (133 mg, 0.150 mmol) + 2o (11.1 mg, 0.100 mmol) → β(3)-**3o** + β(4)-**3o**

94% yield (23.9 mg)

β(3)-/β(4)- = 44:56

Compound β(3)-3o. Compound β(3)-**3o** was isolated as a brown oil by column chromatography on silica gel (hexane/EtOAc = 2/1). ^{1}H NMR (500 MHz, $CDCl_3$) δ 8.23 (br s, 1H), 7.56–7.54 (m, 2H), 7.44 (t, J = 7.5 Hz, 2H), 7.37–7.33 (m, 1H), 6.85–6.84 (m, 1H), 6.08–6.07 (m, 1H), 4.15 (dd, J = 9.7, 4.6 Hz, 1H), 3.13–3.07 (m, 1H), 3.07 (s, 3H), 2.80 (dd, J = 18.3, 5.2 Hz, 1H); ^{13}C{^{1}H} NMR (100 MHz, $CDCl_3$) δ 179.2, 176.8, 132.3, 131.2, 129.0, 127.9, 127.6, 118.6, 116.1, 107.2, 38.1, 37.9, 25.1. HRMS (FD) Calcd for $C_{15}H_{14}N_2O_2$: M, 254.1055. Found: *m/z* 254.1045.

The regiochemistry of products, that is, the structure of β(3)-**3o** was supported by a ^{1}H–^{1}H NOESY NMR experiment.

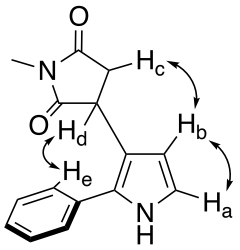

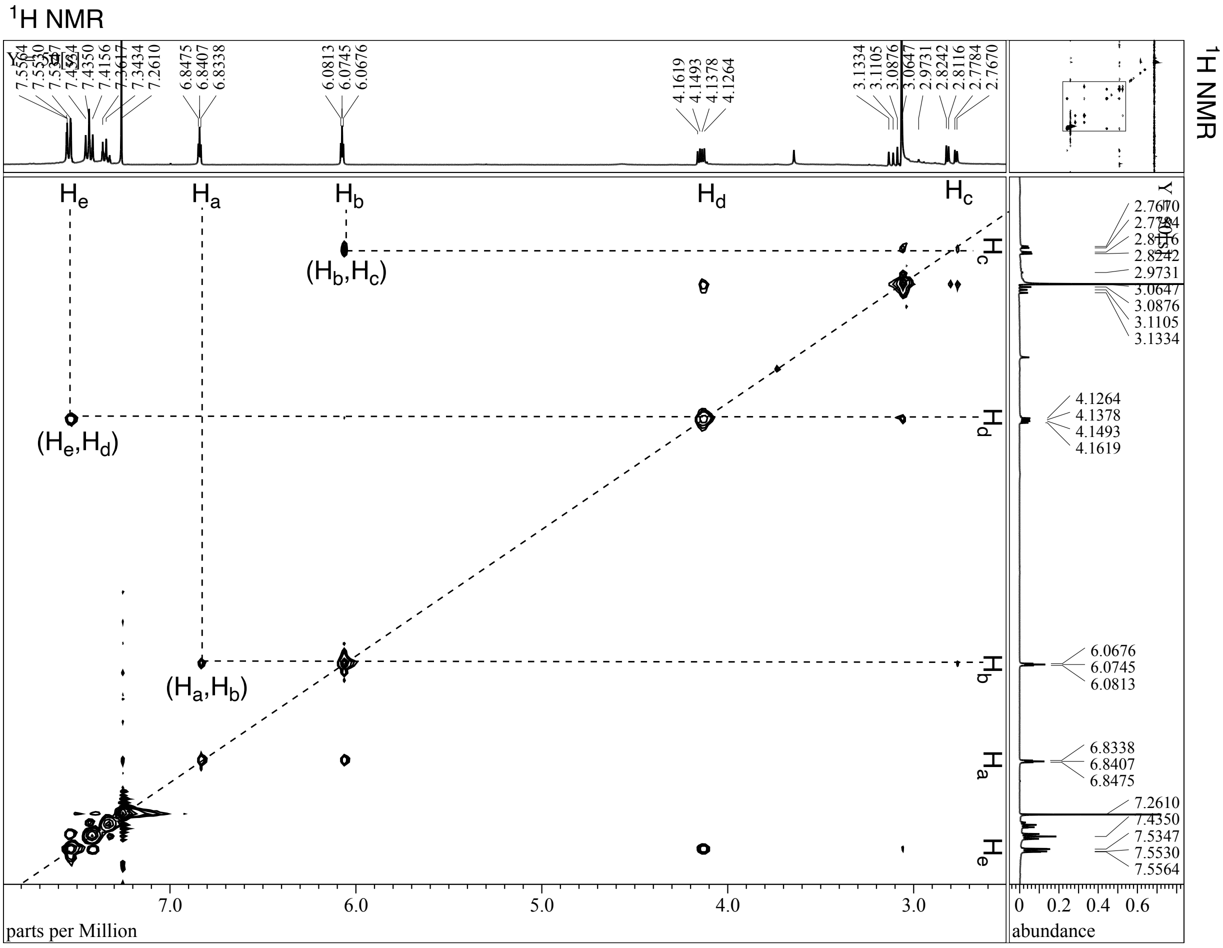

Figure S3. A ^{1}H–^{1}H NOESY NMR spectrum of β(3)-**3o**.

Compound β(4)-3o. Compound β(4)-**3o** was isolated as a brown solid [mp 160 °C (decomp.)] by column chromatography on silica gel (hexane/EtOAc = 2/1). ^{1}H NMR (500 MHz, $CDCl_3$) δ 8.41 (br s, 1H), 7.45–7.43 (m, 2H), 7.38–7.35 (m, 2H), 7.23 (tt, J = 7.2, 1.4 Hz, 1H), 6.86–6.85 (m, 1H), 6.43 (t, J = 2.3 Hz, 1H), 4.03 (dd, J = 9.2, 4.6 Hz, 1H), 3.19 (dd, J = 18.3, 9.7 Hz, 1H), 3.05 (s, 3H), 2.87 (dd, J = 13.2, 5.2 Hz, 1H); ^{13}C{^{1}H} NMR (100 MHz, $CDCl_3$) δ 178.5, 176.6, 133.2, 132.2, 128.9, 126.7, 123.9, 120.7, 116.6, 104.5, 38.8, 36.6, 25.1. HRMS (FD) Calcd for $C_{15}H_{14}N_2O_2$: M, 254.1055. Found: *m/z* 254.1062.

The regiochemistry of products, that is, the structure of β(4)-**3o** was supported by a ^{1}H–^{1}H NOESY NMR experiment.

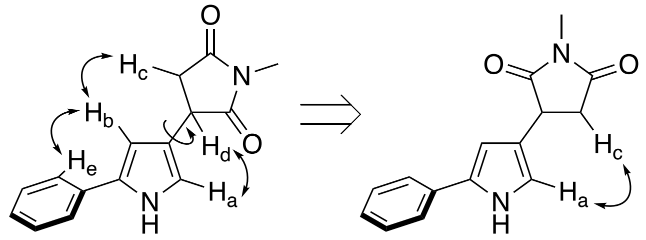

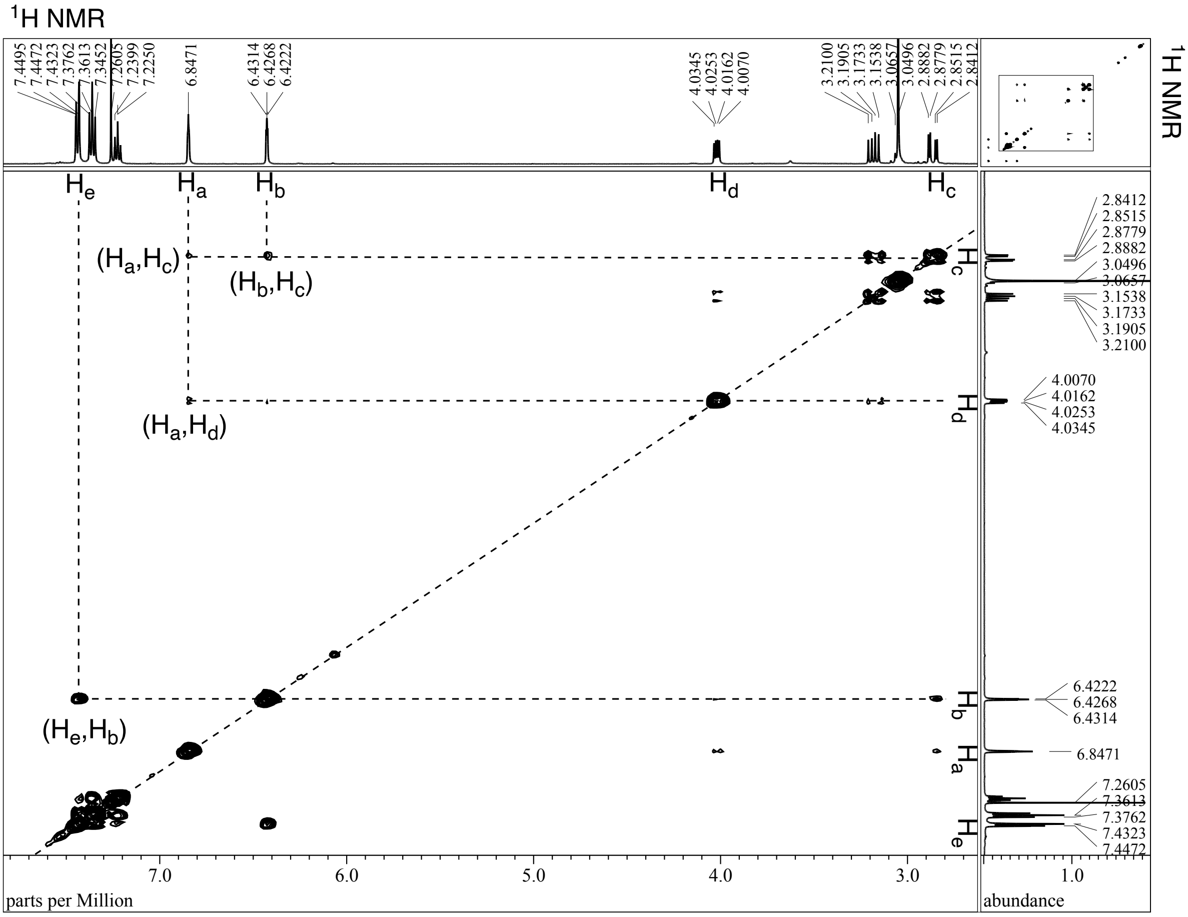

Figure S4. A ^{1}H–^{1}H NOESY NMR spectrum of β(4)-**3o**.

IX. Transformation of β-3a

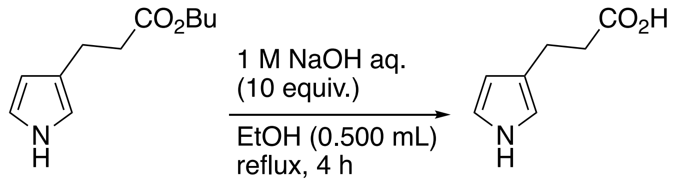

β-**3a**
(19.5 mg,
0.100 mmol)

6
88% yield (12.2 mg)

Saponification of β-3a. Under an air atmosphere, a 20 mL Schlenk tube was charged with EtOH (0.500 mL), β-**3a** (19.5 mg, 0.100 mmol), a 1 M NaOH aqueous solution (1.00 mL, 1.00 mmol). After refluxing for 4 h, a 1 M HCl aqueous solution was added to the mixture until pH of the solution reached 5. The resulting solution was extracted with CH_2Cl_2 (5 mL × 3), and the combined organic layer was dried over anhydrous sodium sulfate. Filtration through a cotton plug and evaporation of the solvent followed by drying in vacuo (15 hPa) provided analytically pure **6** in 88% yield (12.2 mg) as a colorless oil. ^{1}H NMR (500 MHz, $CDCl_3$) δ 8.04 (br s, 1H), 6.73 (dd, *J* = 4.6, 2.9 Hz, 1H), 6.63–6.62 (m, 1H), 6.11 (dd, *J* = 4.3, 2.6 Hz, 1H), 2.85 (t, *J* = 7.5 Hz, 2H), 2.66–2.63 (m, 2H) (A signal of CO_2H is not observed.); ^{13}C{^{1}H} NMR (125 MHz, $CDCl_3$) δ 178.3, 122.1, 118.0, 115.2, 108.3, 35.4, 22.1. HRMS (FD) Calcd for $C_7H_9NO_2$: M, 139.0633. Found: *m/z* 139.0628.

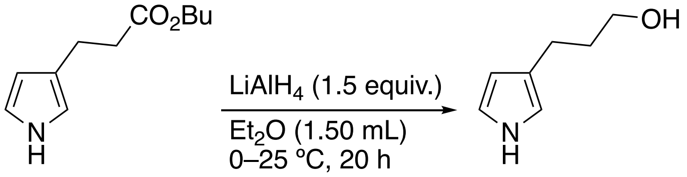

β-**3a**
(39.1 mg,
0.200 mmol

7
87% yield (21.8 mg)

Reduction of β-3a. Under an argon atmosphere, a flame-dried 20 mL Schlenk tube was charged with $LiAlH_4$ (11.4 mg, 0.300 mmol) and Et_2O (1.50 mL). The mixture was cooled to 0 °C, followed by adding β-**3a** (39.1 mg, 0.200 mmol). The resulting suspension was allowed to warm to 25 °C and stirred for 20 h. A 1 M NaOH aqueous solution was added to the mixture at 0 °C until pH of the solution reached 7, and the organic layer was decanted. The remaining salt was washed with Et_2O (5 mL × 3), and the combined organic layer was dried over anhydrous sodium sulfate. Filtration through a cotton plug and evaporation (300 Pa) of the solvent followed by column chromatography on silica gel (hexane/EtOAc = 2/1) gave alcohol **7** in 87% yield (21.8

mg) as a colorless oil. ^{1}H NMR (400 MHz, $CDCl_3$) δ 8.06 (br s, 1H), 6.74 (dd, *J* = 4.8, 2.5 Hz, 1H), 6.61–6.59 (m, 1H), 6.11 (dd, *J* = 4.1, 2.3 Hz, 1H), 3.70 (t, *J* = 6.2 Hz, 2H), 2.60 (t, *J* = 7.6 Hz, 2H), 1.87 (quint, *J* = 7.0 Hz, 2H), 1.33 (br s, 1H); $^{13}C\{^1H\}$ NMR (125 MHz, $CDCl_3$) δ 123.4, 117.9, 115.1, 108.4, 62.8, 33.9, 23.3. HRMS (FI) Calcd for $C_7H_{11}NO$: M, 125.0841. Found: *m/z* 125.0847.

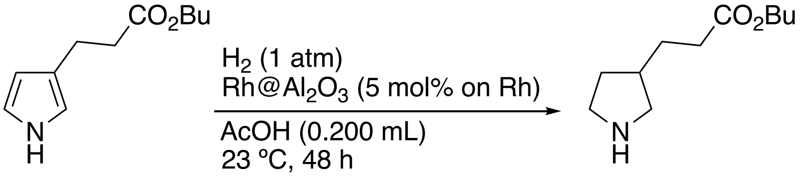

β-**3a**
(39.1 mg,
0.200 mmol)

8
>99% NMR yield
(determined using CH_3NO_2
as an internal standard)

Hydrogenation of β-3a. This reaction was performed according to the literature procedure[11] with the following reagents: β-**3a** (39.1 mg, 0.200 mmol), Rh@Al_2O_3 (5 mol% on Rh, 8.50 mg) and AcOH (0.200 mL). Product **8** was obtained quantitatively as a spectrally pure form without purification. **NOTE**: Compound **8** partially decomposed even after the measurement of the ^{1}H NMR spectrum followed by removal of the solvent and then one week of storage in a freezer (–20 °C). Therefore, **8** is likely to be bench-unstable. ^{1}H NMR (400 MHz, $CDCl_3$) δ 4.07 (t, *J* = 6.9 Hz, 2H), 3.08 (dd, *J* = 10.5, 7.3 Hz, 1H), 2.98–2.87 (m, 2H), 2.46 (dd, *J* = 10.8, 7.1 Hz, 1H), 2.35–2.30 (m, 2H), 2.16 (br s, 1H), 2.06–1.89 (m, 2H), 1.74–1.66 (m, 2H), 1.64–1.57 (m, 2H), 1.43–1.28 (m, 3H), 0.94 (t, *J* = 7.6 Hz, 3H); $^{13}C\{^1H\}$ NMR (100 MHz, $CDCl_3$) δ 173.7, 64.3, 52.9, 46.8, 39.2, 33.5, 32.3, 30.7, 29.7, 19.2, 13.7. HRMS (FI) Calcd for $C_{11}H_{21}NO_2$: M, 199.1572. Found: *m/z* 199.1572.

X. Mechanistic Studies

An NMR Spectrum of $H_2O•B(C_6F_5)_3$ in CH_2Cl_2. The following $^{11}B\{^1H\}$ NMR spectrum was obtained by dissolving a sublimed commercial source in CH_2Cl_2.

[11] S. E. Denmark, H. Matsuhashi, *J. Org. Chem.* **2002**, *67*, 3479–3486.

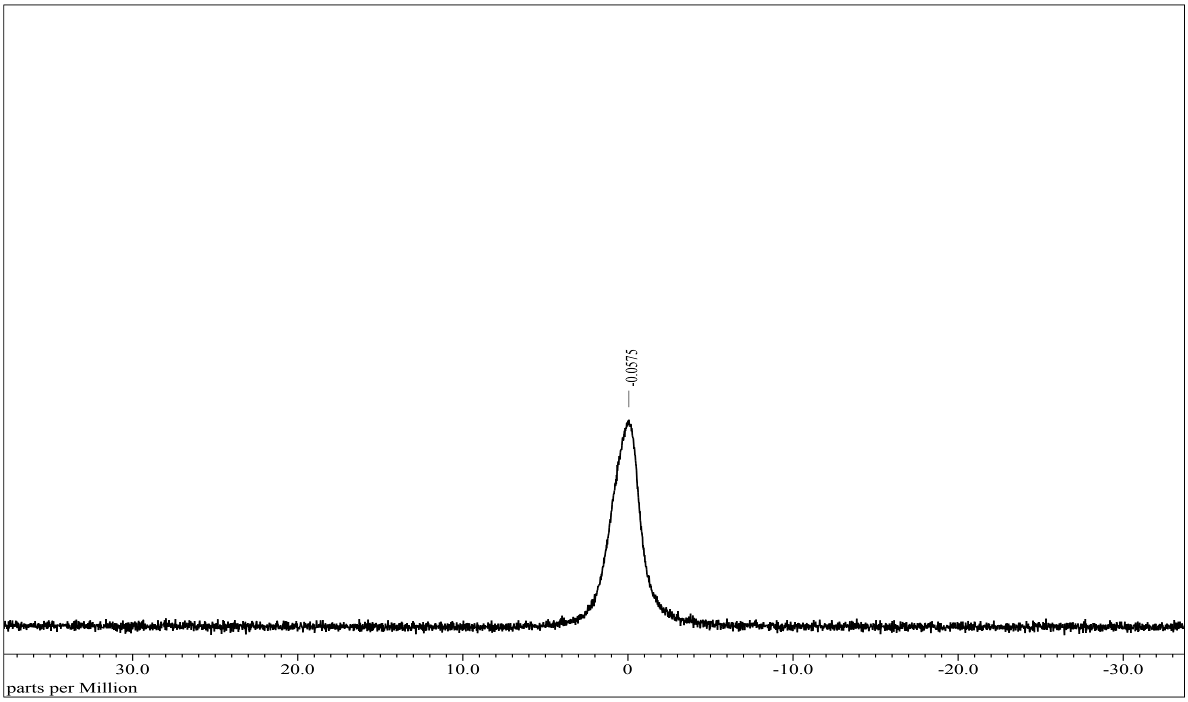

Figure S5. An NMR Spectrum of H_2O•$B(C_6F_5)_3$ in CH_2Cl_2.

An NMR Spectrum of THF•$B(C_6F_5)_3$ in THF. According to the report to obtain H_2O-free $B(C_6F_5)_3$ in CH_2Cl_2,[12] the following $^{11}B\{^1H\}$ NMR spectrum was obtained after treating H_2O •$B(C_6F_5)_3$ with allyltrimethylsilane (5 equiv.) in THF at rt for 1 h.

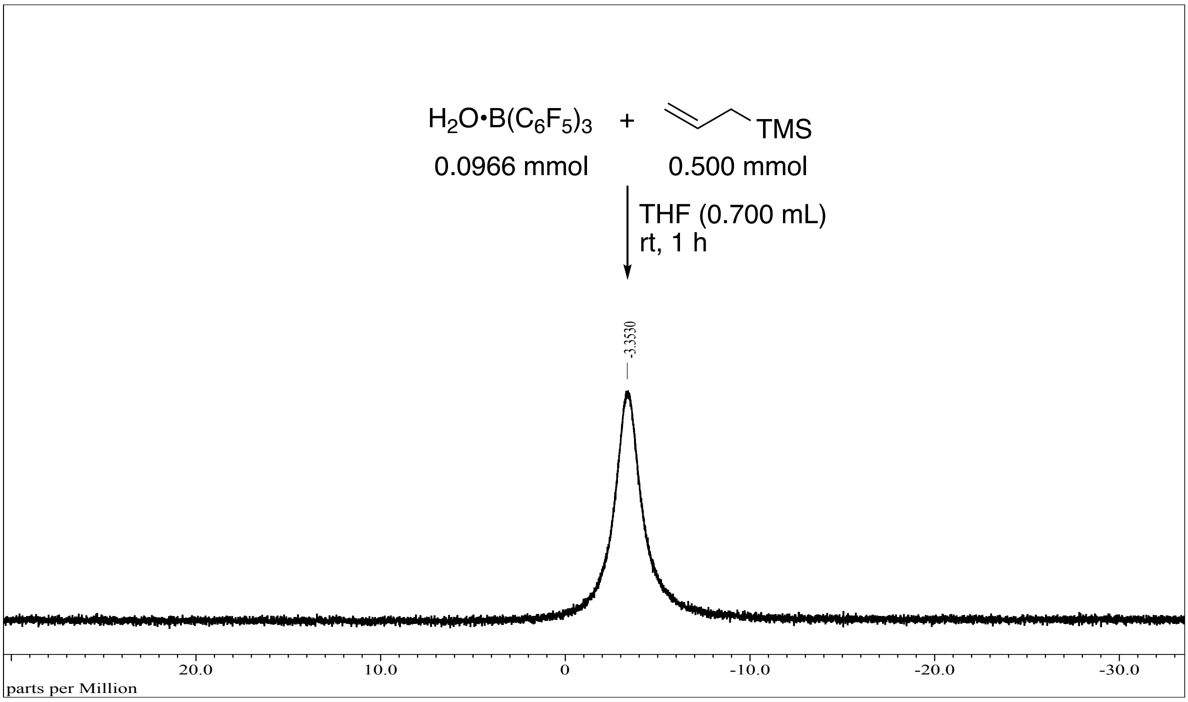

[12] M. Shibuya, M. Okamoto, S. Fujita, M. Abe, Y. Yamamoto, *ACS Catal.* **2018**, *8*, 4189–4193.

Figure S6. An NMR Spectrum of THF•B(C_6F_5)$_3$ in THF.

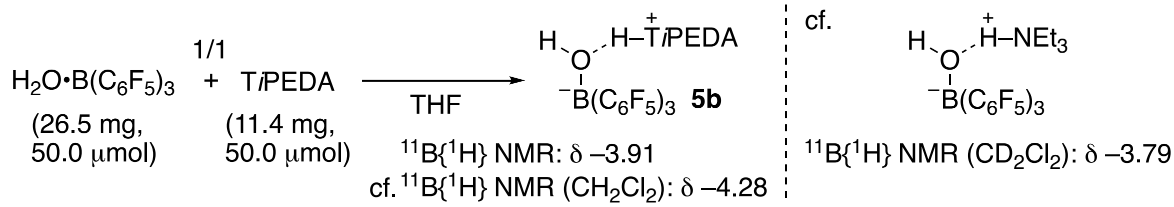

Compound 5b (Schemes 6 and 7). A THF-d_8 (for ^{1}H, ^{13}C{^{1}H}, and ^{19}F NMR spectra) or THF (for a ^{11}B{^{1}H} NMR spectrum) solution of H_2O•B(C_6F_5)$_3$ (26.5 mg, 50.0 μmol) was treated directly with T*i*PEDA (11.4 mg, 50.0 μmol) in an NMR tube. When ^{11}B{^{1}H} NMR spectroscopy of the same mixture, H_2O•B(C_6F_5)$_3$ and T*i*PEDA, was measured in CH_2Cl_2, the ^{11}B signal appeared at δ –4.28. This signal is close to that observed at δ –3.79 for [HOB(C_6F_5)$_3$]$^-$(HNEt$_3$)$^+$, which is reportedly derived from B(C_6F_5)$_3$, H_2O, and NEt$_3$ in CD_2Cl_2.[13] Accordingly, the structural formula produced from H_2O•B(C_6F_5)$_3$ and T*i*PEDA in THF was considered to be **5b**, as shown in the above. ^{1}H NMR (500 MHz, THF-d_8) δ 4.52 (br s, 2H), 3.37 (sept, J = 6.6 Hz, 4H), 2.91 (s, 4H), 1.17 (d, J = 6.3 Hz, 24H); ^{13}C{^{1}H} NMR (125 MHz, THF-d_8) δ 148.9 (dm, J = 238 Hz, *o*-C in –C_6F_5), 139.3 (dm, J = 245 Hz, *p*-C in –C_6F_5), 137.4 (dm, J = 242 Hz, *m*-C in –C_6F_5), 127.1 (br, *ipso*-C in –C_6F_5), 51.9, 45.0, 19.7; ^{19}F NMR (471 MHz, THF-d_8) δ –134.6 (d, J = 20.6 Hz, 6F, *o*-F), –163.4 (t, J = 20.6 Hz, 3F, *p*-F), –166.7 to –166.8 (m, 6F, *m*-F); ^{11}B{^{1}H} NMR (128 MHz, THF) δ –3.91. MS (LC-MS, ESI, positive mode): Calcd for $C_{14}H_{33}N_2^+$ (H–T*i*PEDA$^+$): 229. Found: *m/z* 229. MS (LC-MS, ESI, negative mode): Calcd for $C_{18}HBF_{15}O^-$ [(F_5C_6)$_3$B$^-$–OH]: 528. Found: *m/z* 528. HRMS (FD) Calcd for $C_{14}H_{33}N_2^+$ (H–T*i*PEDA$^+$): 229.2644. Found: *m/z* 229.2627. HRMS (EI) Calcd for $C_{18}BF_{15}$ [(F_5C_6)$_3$B]: 511.9854. Found: *m/z* 511.9838.

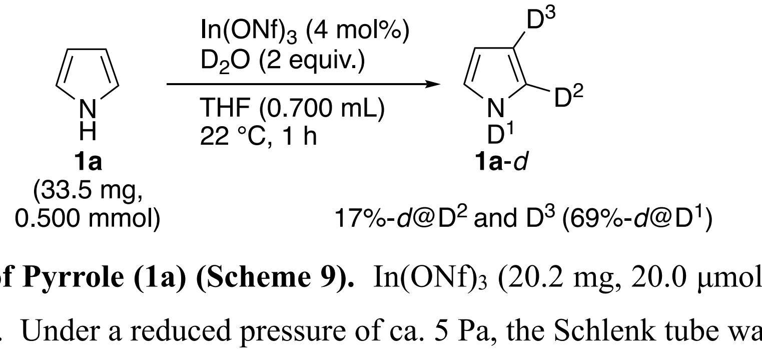

Deuteration of Pyrrole (1a) (Scheme 9). In(ONf)$_3$ (20.2 mg, 20.0 μmol) was placed in a 20 mL Schlenk tube. Under a reduced pressure of ca. 5 Pa, the Schlenk tube was heated at 50 °C

[13] A. Di Saverio, F. Focante, I. Camurati, L. Resconi, T. Beringhelli, G. D'Alfonso, D. Donghi, D. Maggioni, P. Mercandelli, A. Sironi, *Inorg. Chem.* **2005**, *44*, 5030–5041.

for 20 min, 70 °C for 20 min, 90 °C for 20 min, 120 °C for 20 min, and 150 °C for 20 min. After cooling down to room temperature (rt), the Schlenk tube was filled with argon. To this was added THF (0.700 mL), and the mixture was stirred at rt for 1 min to obtain a homogeneous solution, in which $In(ONf)_3$ was thus dissolved in THF. Successively, **1a** (33.5 mg, 0.500 mmol) and D_2O (20.0 mg, 1.00 mmol) were added to the THF solution, and the resulting mixture was stirred at 22 °C for 30 min so that NMR spectroscopy could be measured 1 h later. To this were added CH_2Br_2 (86.9 mg, 0.500 mmol) as an internal standard for estimating the degree of deuteration and tetramethylsilane as an internal reference standard (0.00 ppm) for NMR spectroscopy. A part of the resulting solution was transferred through a cannula into an NMR tube equipped with an adapter connected to a dual vacuum/argon manifold. After measuring no-deuterium proton NMR spectroscopy, the solution in the NMR tube was transferred back to the Schlenk tube while being washed with EtOAc (4 mL). A saturated $NaHCO_3$ aqueous solution (25.0 μL) was added to quench the reaction, followed by adding *o*-dichlorobenzene (73.5 mg, 0.500 mmol) as an internal standard for GC analysis, indicating that the conversion (%) of **1a** was 3%. Based on the conversion (%) of **1a**, the degree of deuteration of **1a** was determined to be the following: 69% at D^1, 17% at D^2, and 17% at D^3.

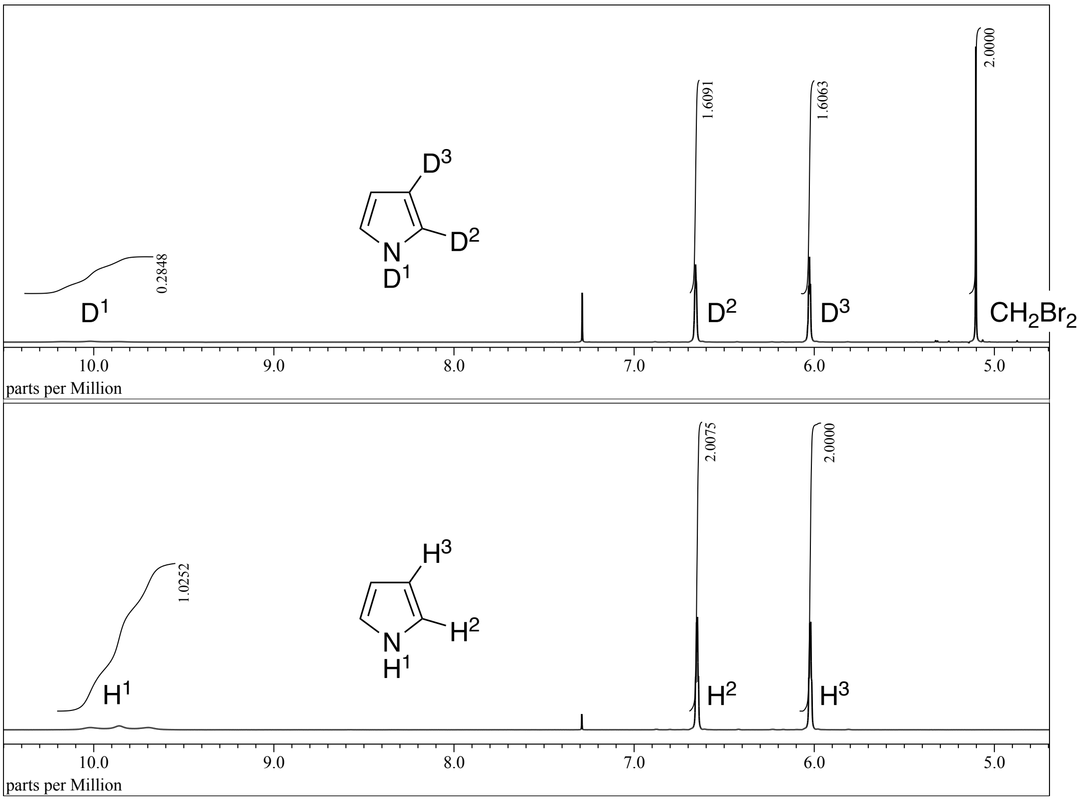

Figure S7. NMR spectrum of deuterated and non-deuterated pyrrole.

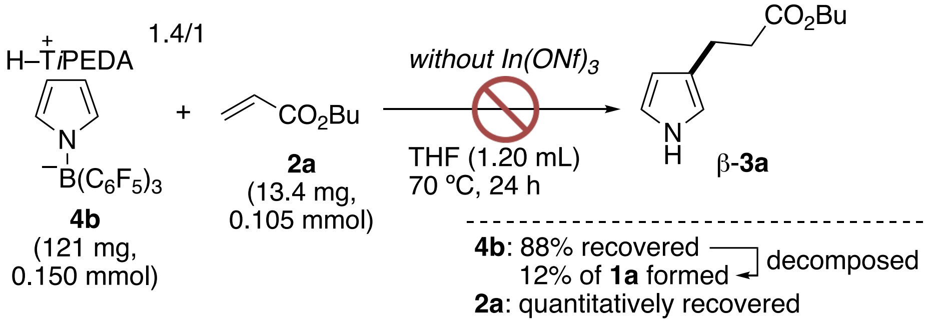

Reaction of 4b with 2a in the Absence of In(ONf)$_3$ (Scheme 10a). A flame-dried 20 mL Schlenk tube was charged with **4b** (121 mg, 0.150 mmol), evacuated under vacuum (ca. 5 Pa) at rt for 1 h, and then filled with argon. To this was added THF (1.20 mL), and the mixture was stirred at rt for 1 min. Successively, **2a** (13.4 mg, 0.105 mmol) was added to the THF solution, and the resulting mixture was stirred at 70 °C for 24 h. To this was added CH_3NO_2 (7.3 mg, 0.12 mmol) as an internal standard for estimating the conversion (%) of **4b** and **2a** as well as the yield (%) of **1a** by NMR spectroscopy. Tetramethylsilane was also added as an internal reference standard (0.00 ppm) for NMR spectroscopy. The conversion (%) of **4b** and **2a** as well as the yield (%) of **1a** based on **4b** were determined by measuring no-deuterium proton NMR spectroscopy (see below), and the results are summarized in the above scheme. **NOTE**: In comparison to the NMR signals of **4b** independently measured in THF, those of **4b** in the reaction mixture were shifted downfield by approximately 0.04 ppm. This may be due partly to contamination by a significant amount of silicone grease used for the Schlenk tube when performing the reaction.

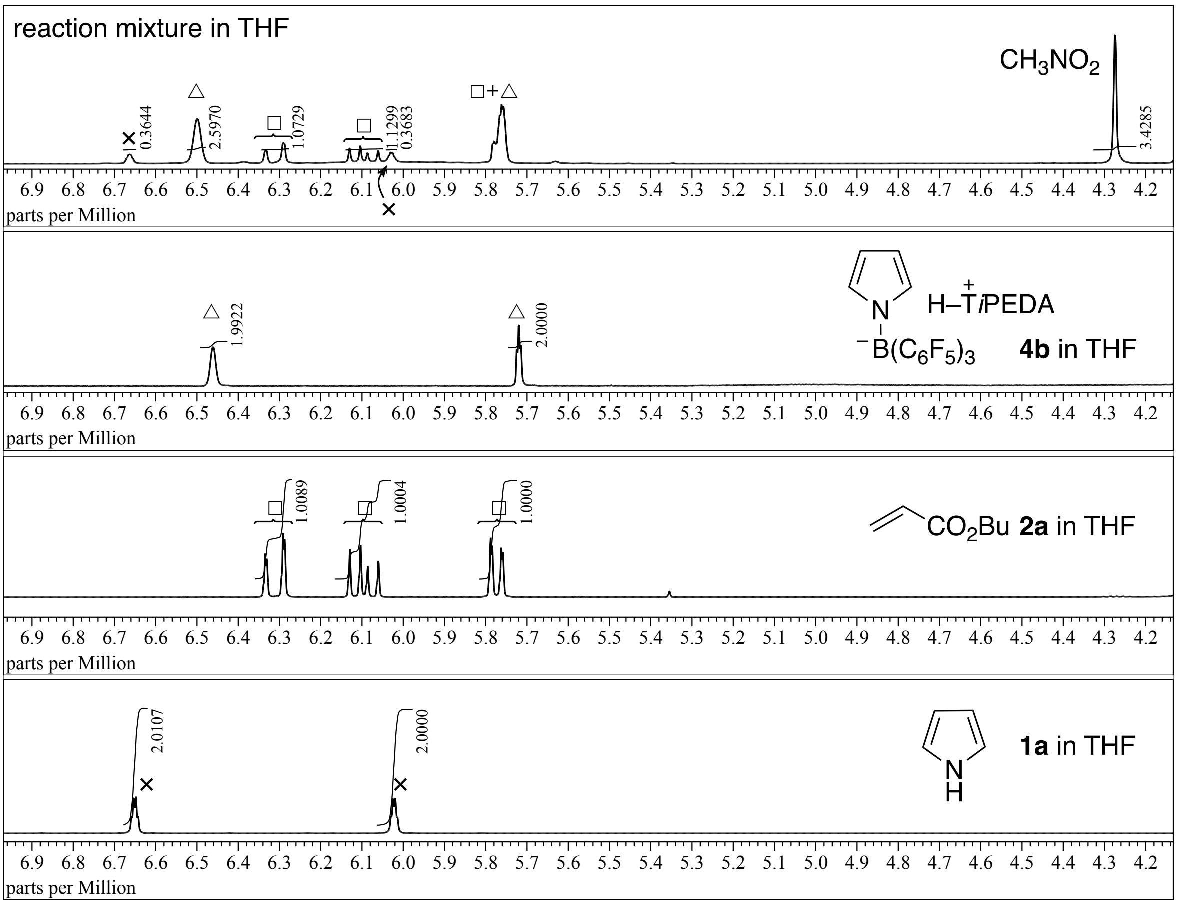

Figure S8. Comparison of NMR spectrum.

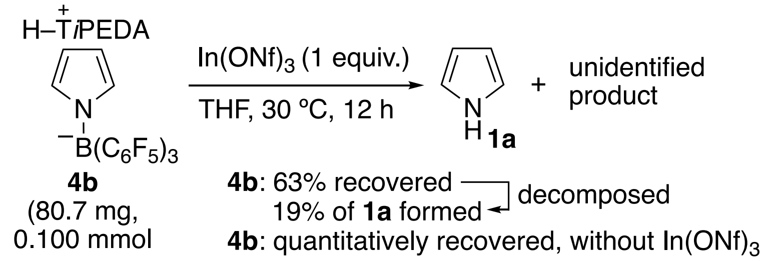

Reaction of 4b with In(ONf)$_3$ (Scheme 10b). In(ONf)$_3$ (101 mg, 0.100 mmol) was placed in a 20 mL Schlenk tube. Under a reduced pressure of ca. 5 Pa, the Schlenk tube was heated at 50 °C for 20 min, 70 °C for 20 min, 90 °C for 20 min, 120 °C for 20 min, and 150 °C for 20 min. After cooling down to rt, the Schlenk tube was filled with argon. To this was placed **4b** (80.6 mg, 0.0998 mmol), and the Schlenk tube was evacuated under vacuum (ca. 5 Pa) at rt for 1 h, and then filled with argon. To this was added THF (1.20 mL), and the resulting solution was stirred at 30 °C for 12 h. To this was added CH_3NO_2 (6.0 mg, 0.098 mmol) as an internal standard for estimating

the conversion (%) of **4b** and the yield (%) of **1a** by NMR spectroscopy. Tetramethylsilane was also added as an internal reference standard (0.00 ppm) for NMR spectroscopy. The conversion (%) of **4b** and the yield (%) of **1a** based on **4b** were determined by measuring no-deuterium proton NMR spectroscopy (see below), and the results are summarized in the above scheme.

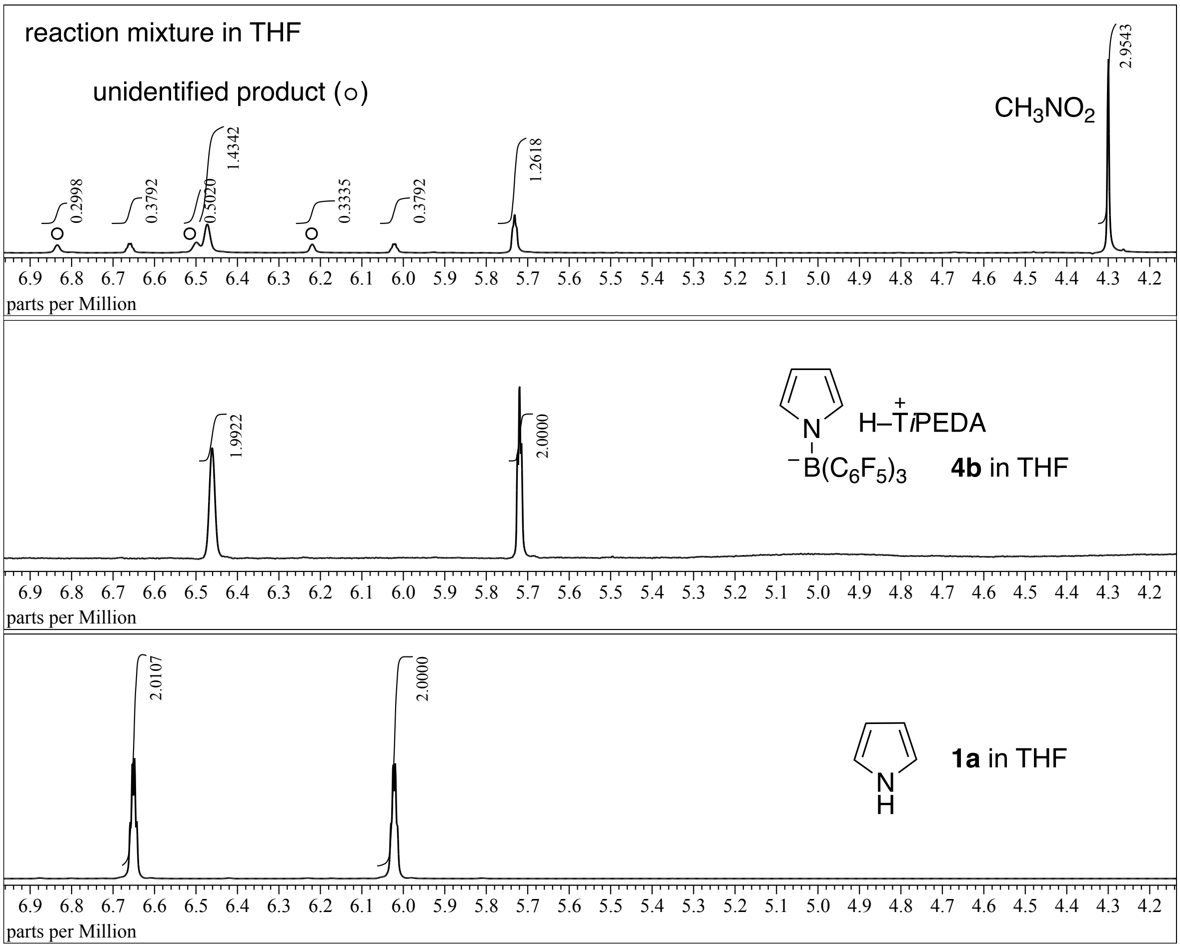

Figure S9. Comparison of NMR spectrum.

Reaction of 4b without In(ONf)$_3$ (Scheme 10b). The experiment without using In(ONf)$_3$ was started by placing **4b** (80.9 mg, 0.100 mmol) to a flame-dried 20 mL Schlenk tube, omitting the drying of In(ONf)$_3$ described at the beginning of the above experimental procedure. The subsequent procedure was then carried out in the same manner as detailed in the procedure above. The conversion (%) of **4b** and the yield (%) of **1a** were determined by measuring no-deuterium proton NMR spectroscopy based on CH_3NO_2 (6.5 mg, 0.11 mmol) as an internal standard, showing that **4b** was recovered quantitatively, and no **1a** was thus formed. This result is summarized in the above scheme.

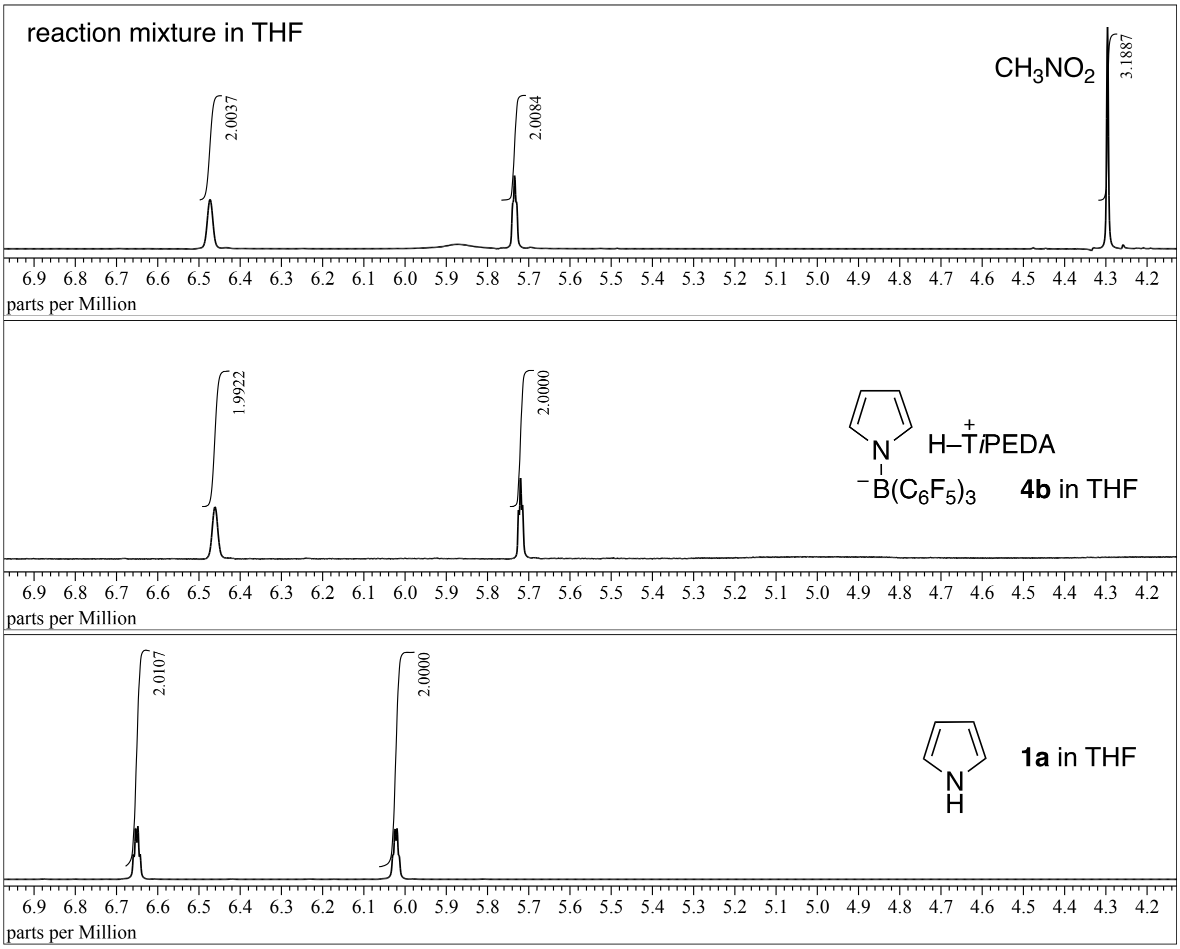

Figure S10. Comparison of NMR spectrum.

XI. ^{1}H, ^{13}C{^{1}H}, ^{13}C{^{19}F}, ^{19}F and/or ^{11}B{H} NMR Spectra

NMR spectra of substrates and products are collected in the following pages. Only a ^{1}H NMR spectrum is provided in the case of a compound for which ^{1}H NMR, ^{13}C{^{1}H} NMR and HRMS or elemental analysis data have been already reported in the literature.

$^{13}C\{^{19}F\}$ NMR (100 MHz, CD_3CN)

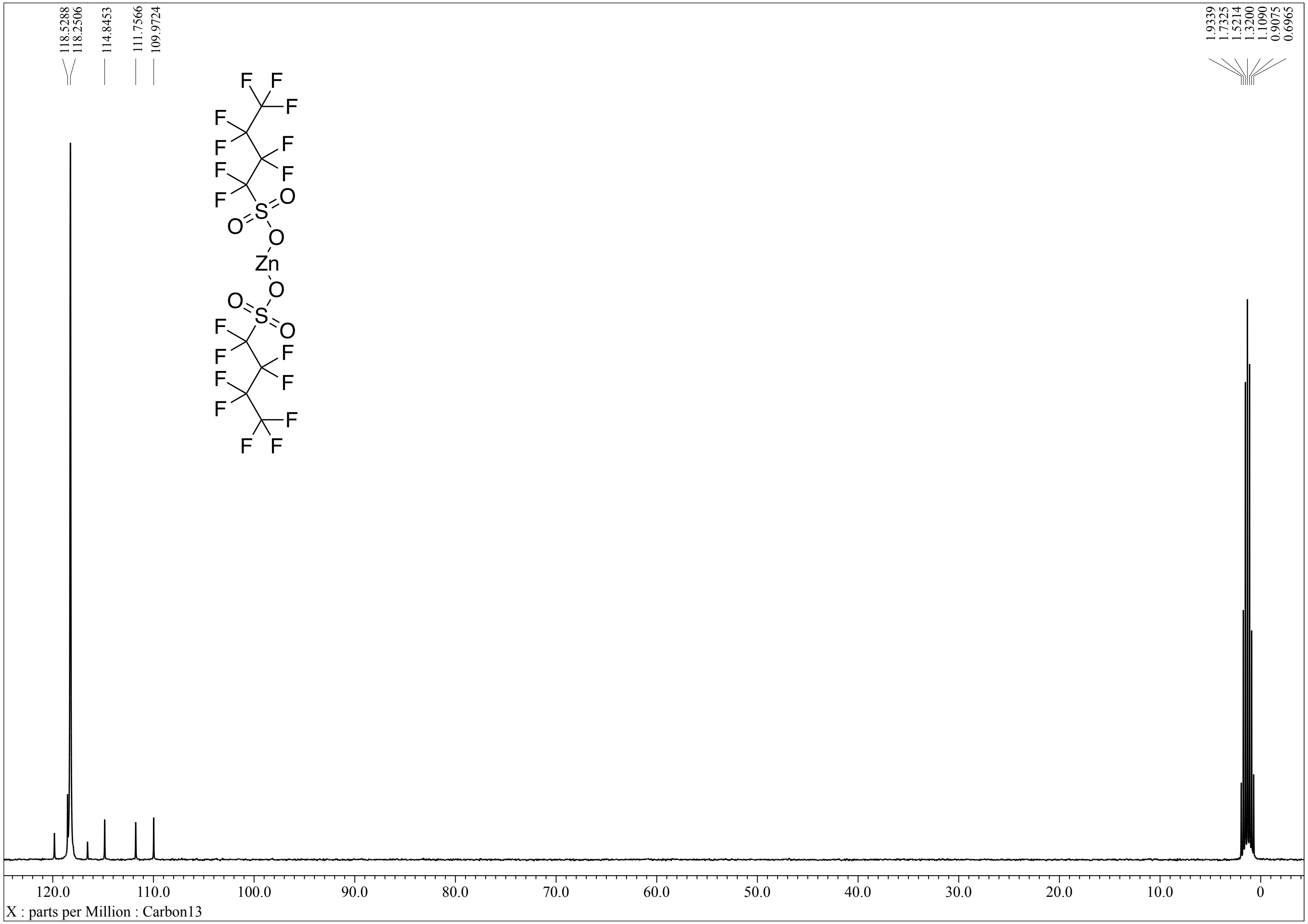

^{19}F NMR (376 MHz, CD_3CN)

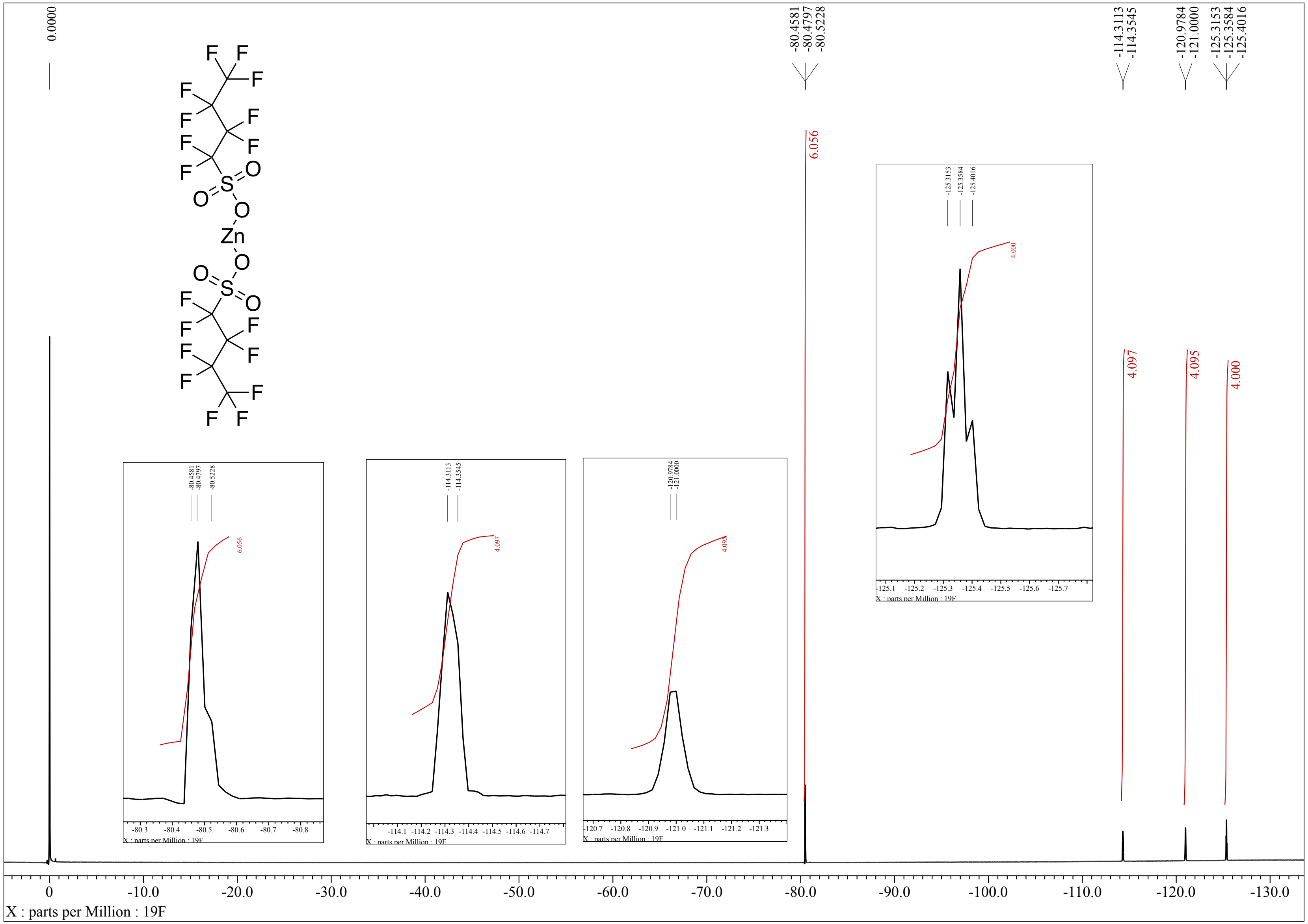

$^{13}C\{^{19}F\}$ NMR (100 MHz, CD_3CN)

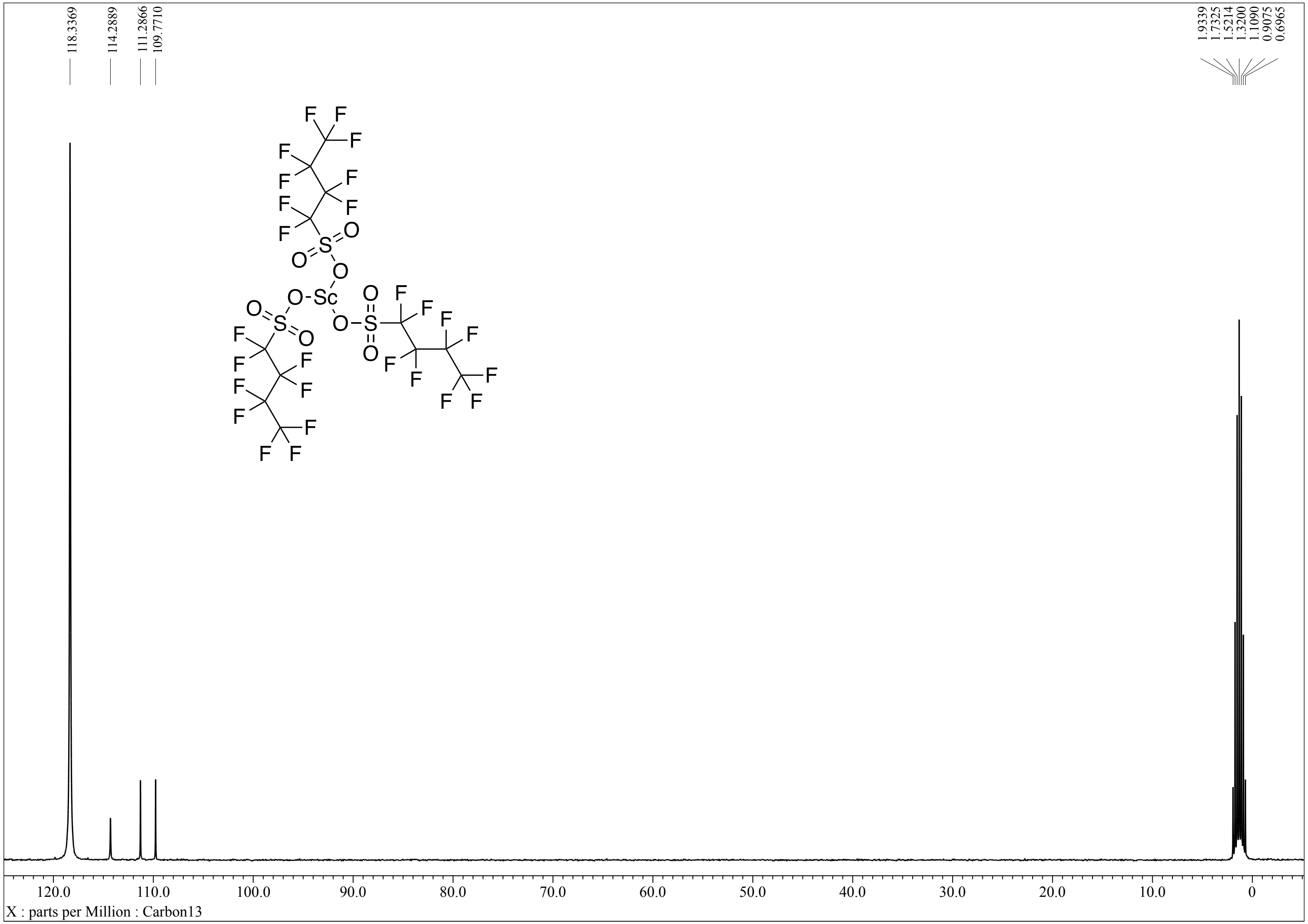

^{19}F NMR (471 MHz, CD_3CN)

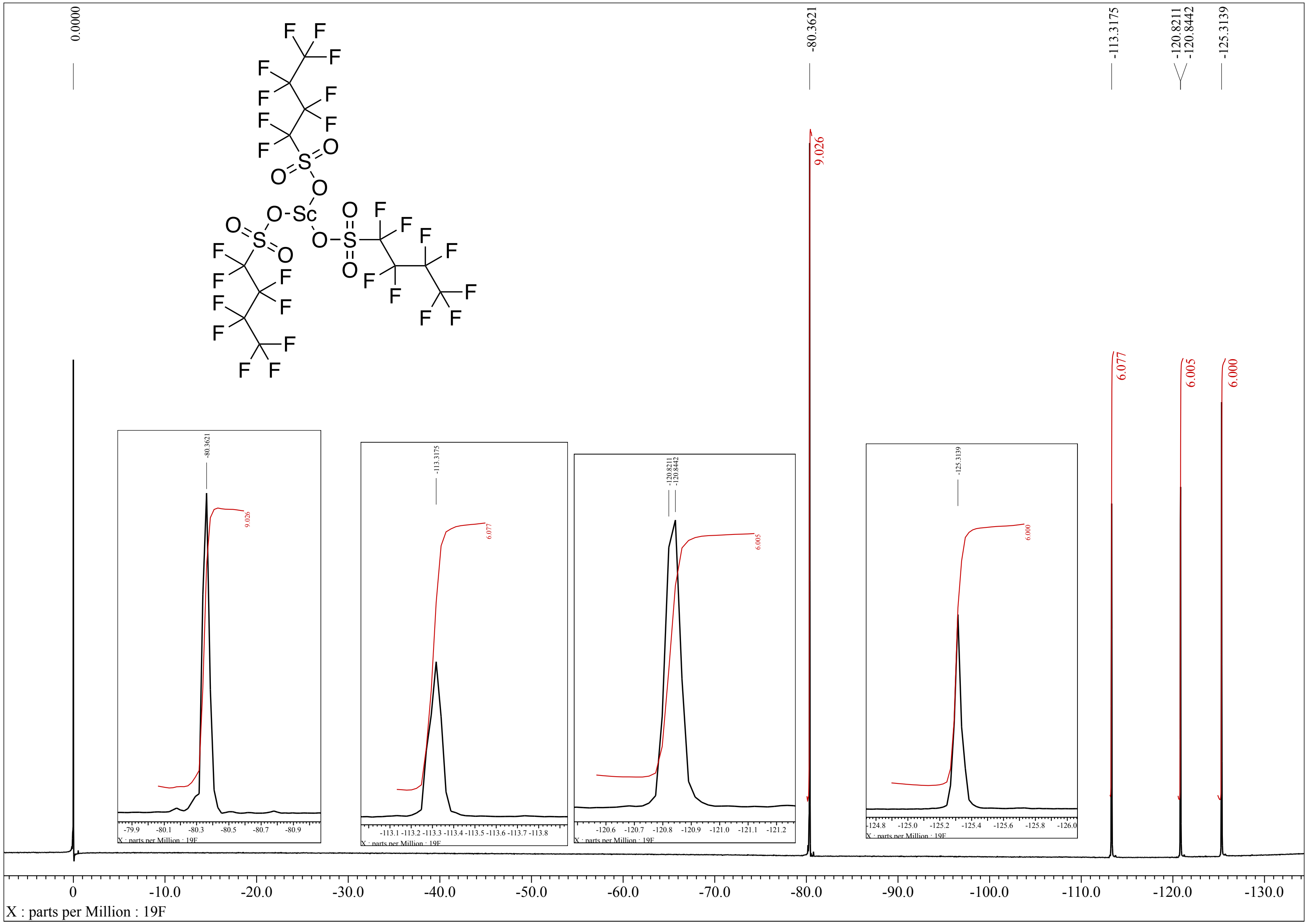

^{1}H NMR (400 MHz, $CDCl_3$)

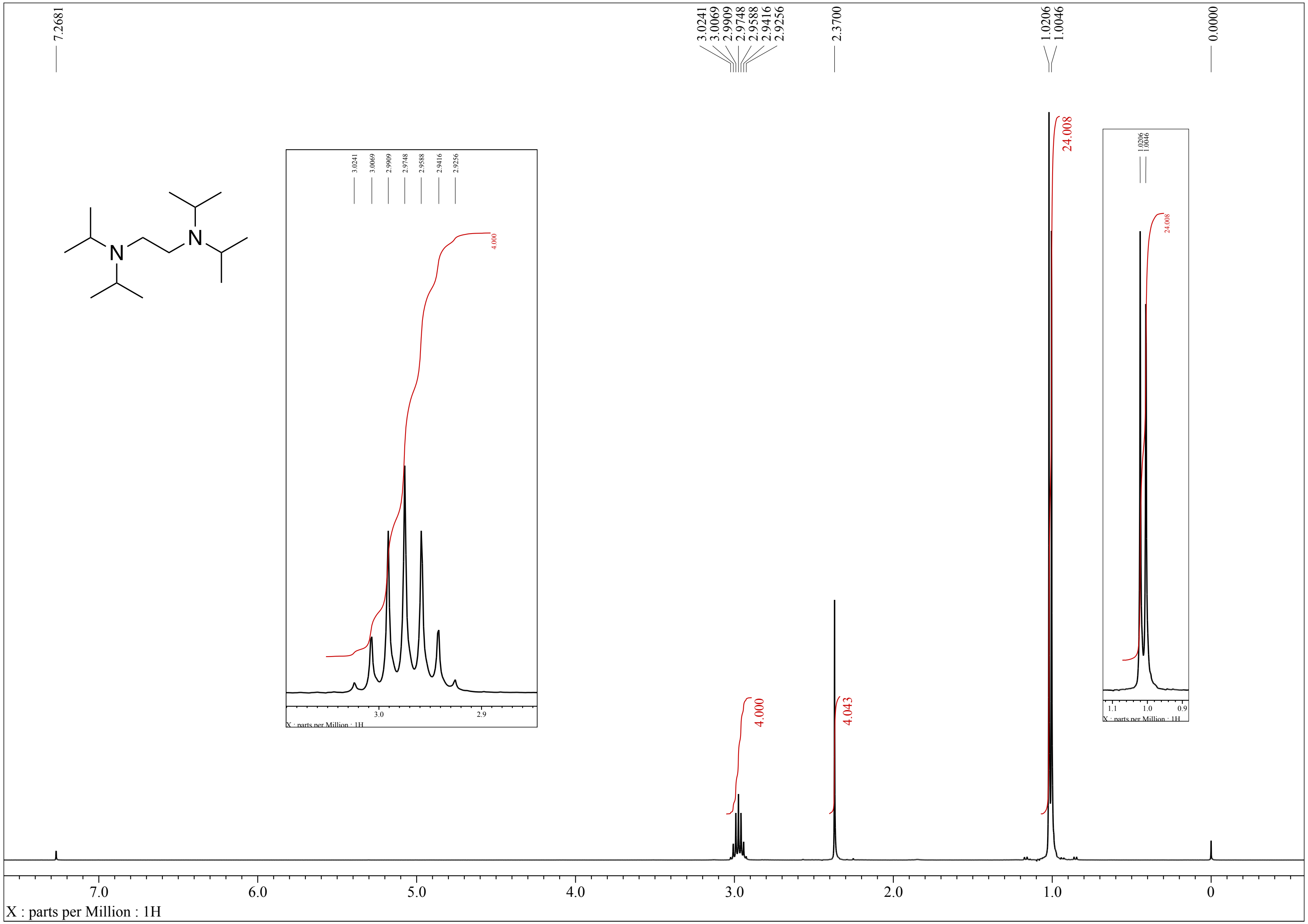

$^{13}C\{^{1}H\}$ NMR (125 MHz, $CDCl_3$)

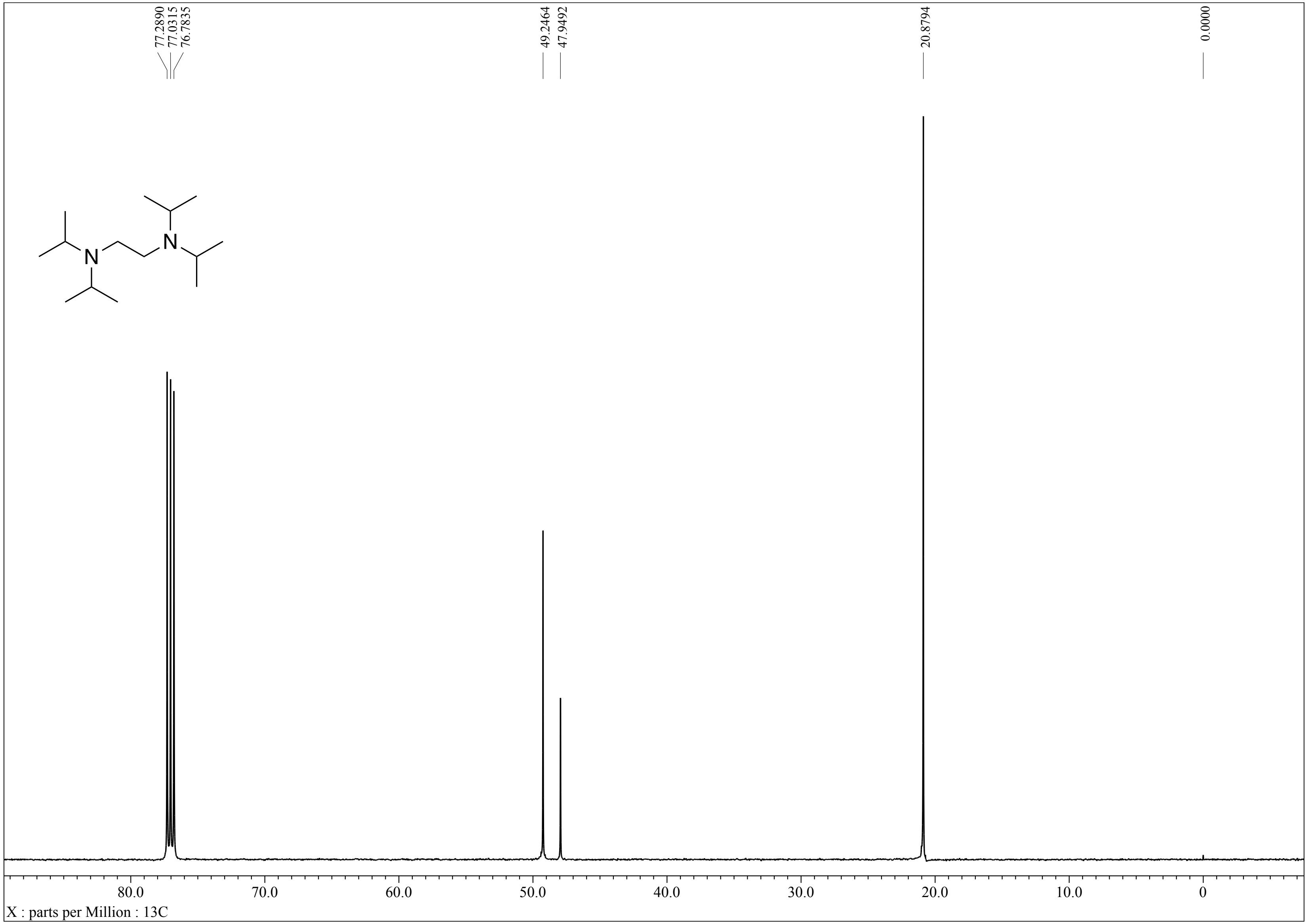

^{1}H NMR (500 MHz, $CDCl_3$)

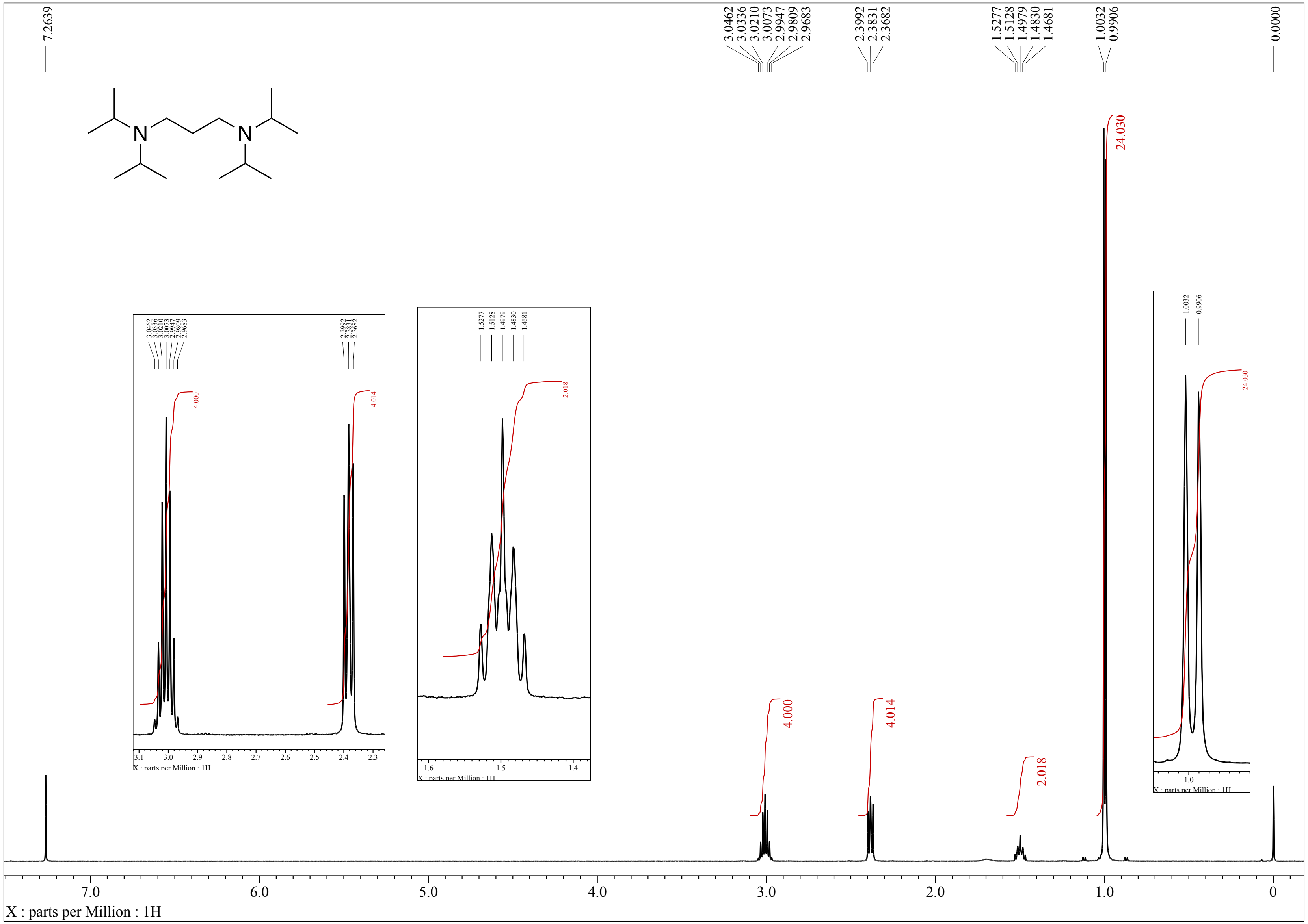

$^{13}C\{^1H\}$ NMR (125 MHz, $CDCl_3$)

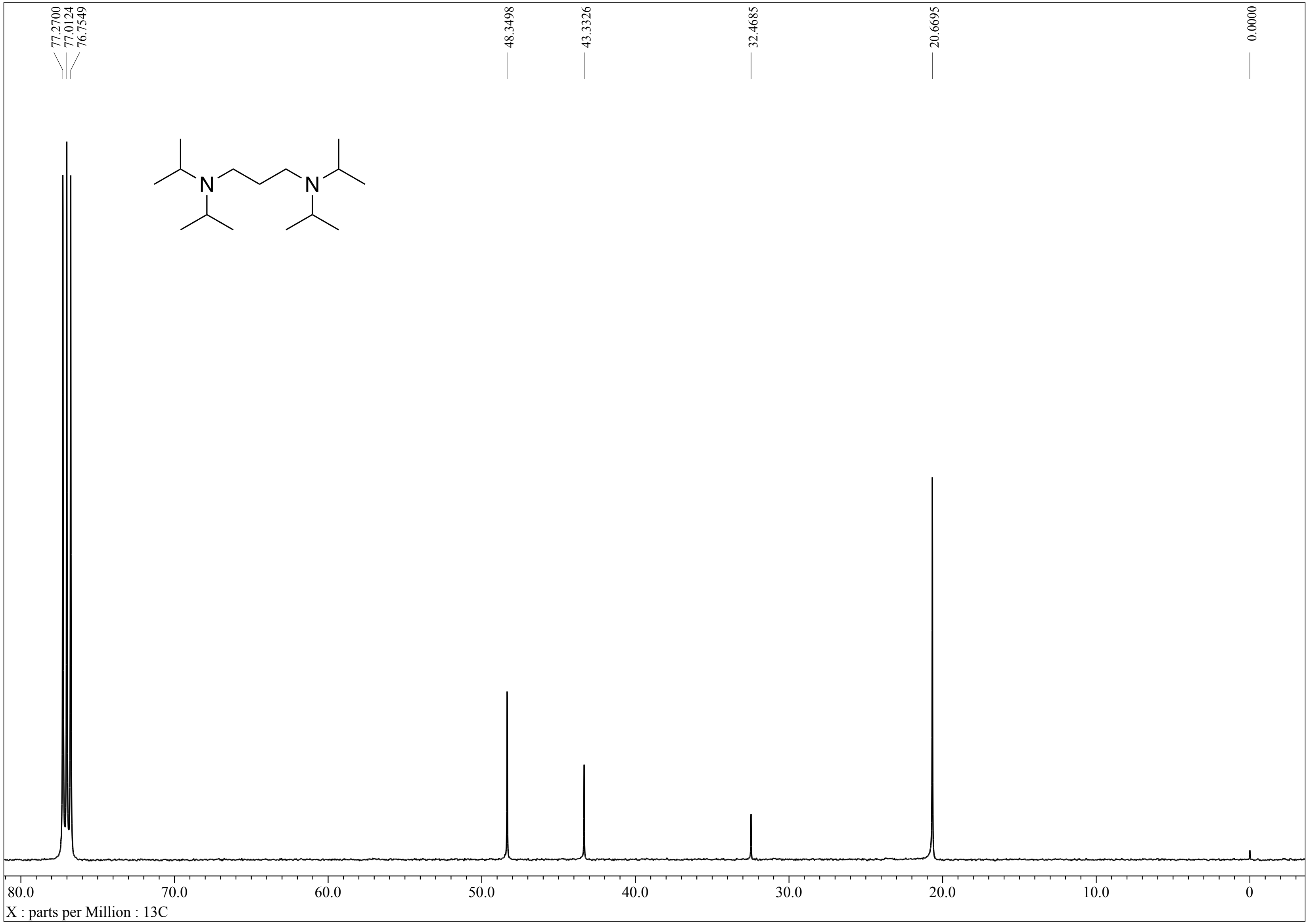

^{1}H NMR (400 MHz, $CDCl_3$)

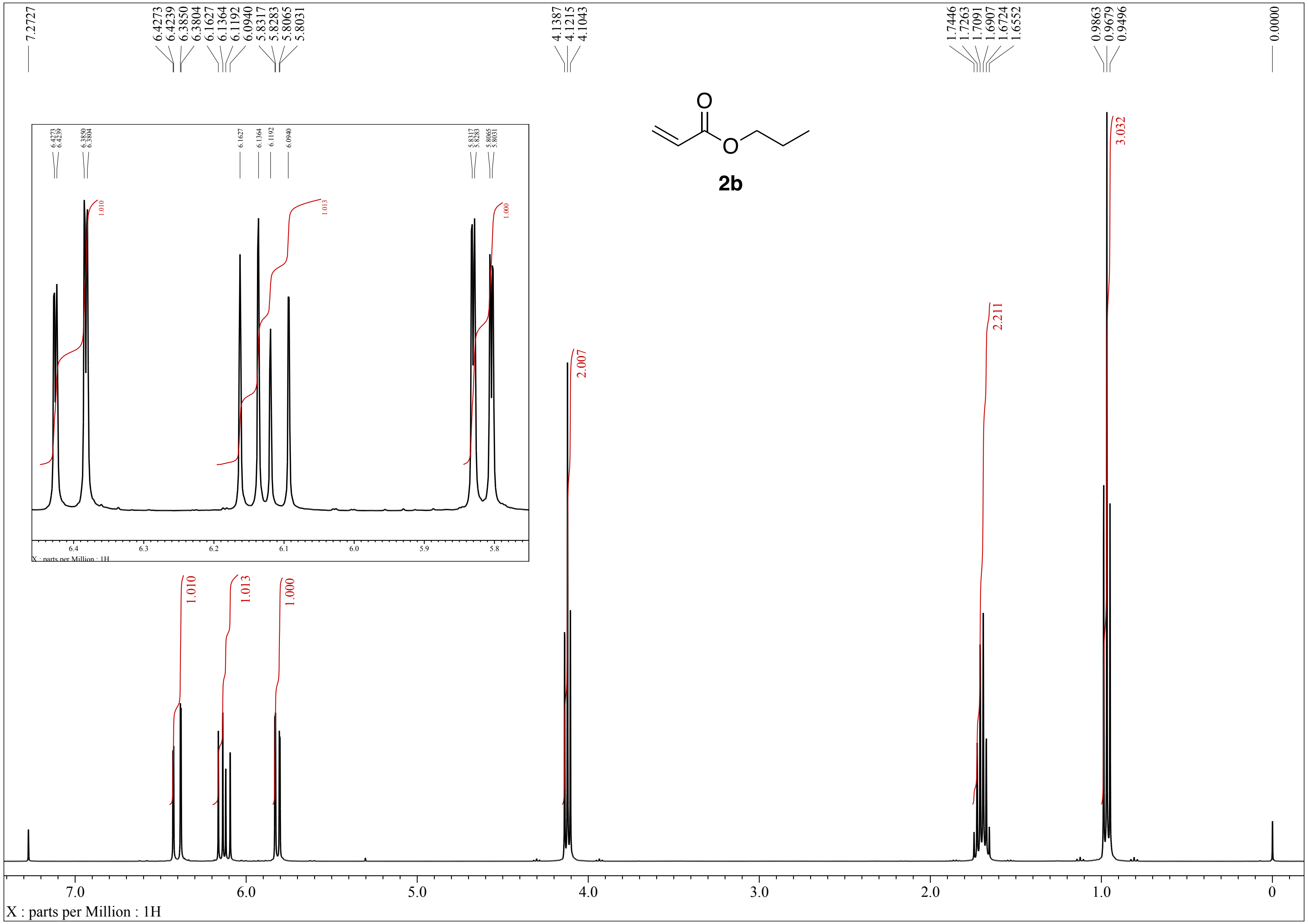

$^{13}C\{^{1}H\}$ NMR (100 MHz, $CDCl_3$)

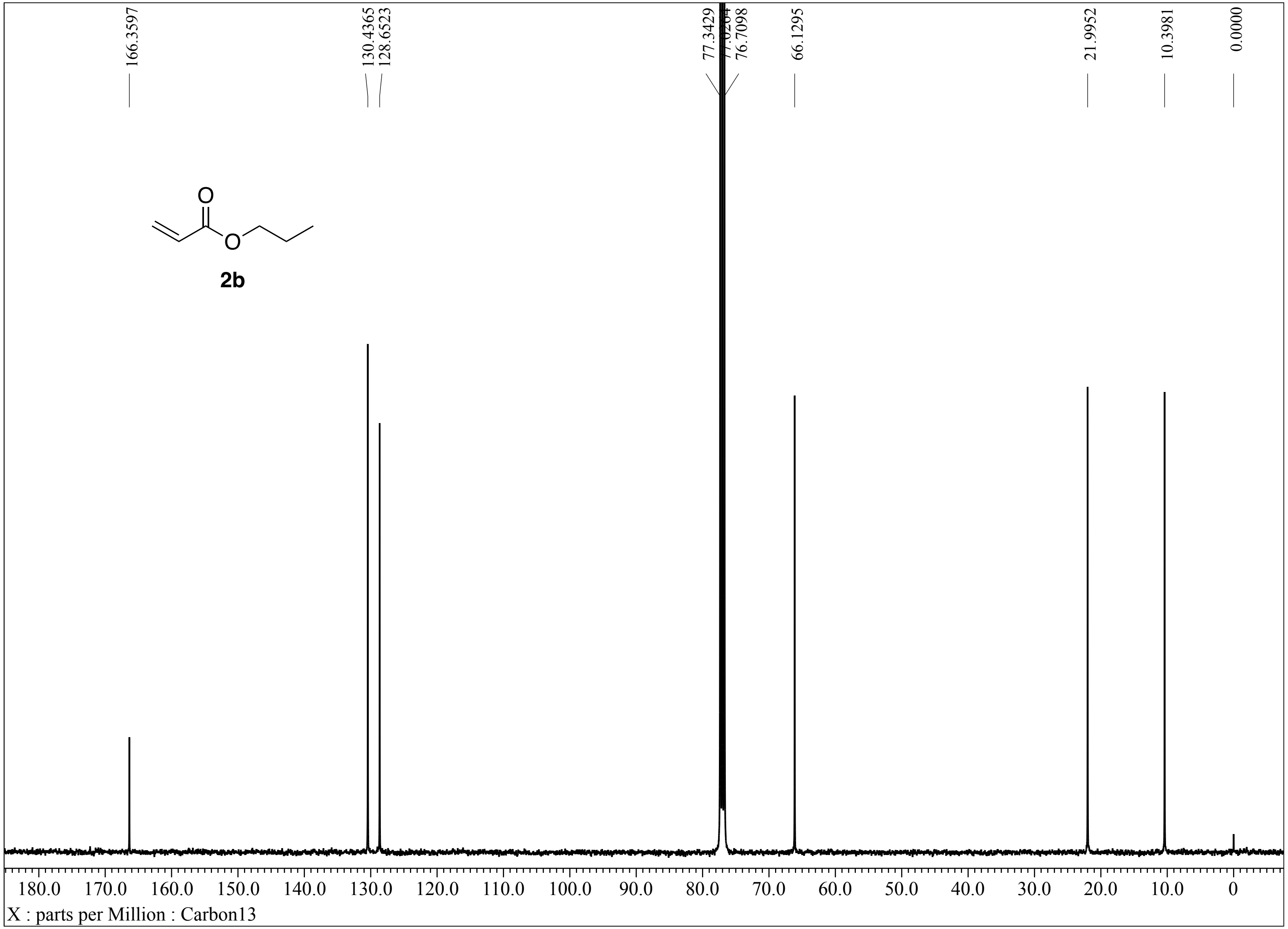

^{1}H NMR (400 MHz, $CDCl_3$)

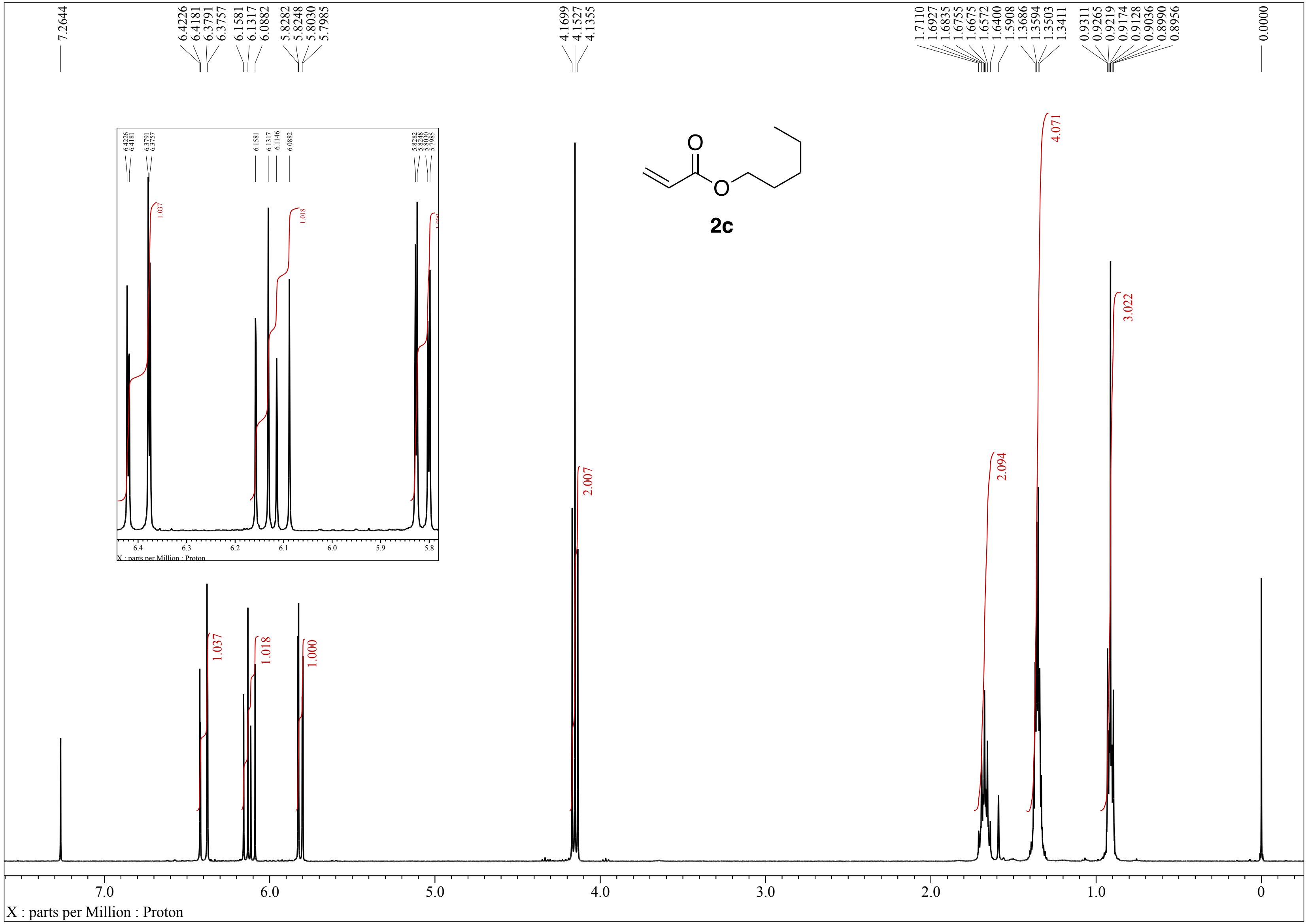

$^{13}C\{^{1}H\}$ NMR (100 MHz, $CDCl_3$)

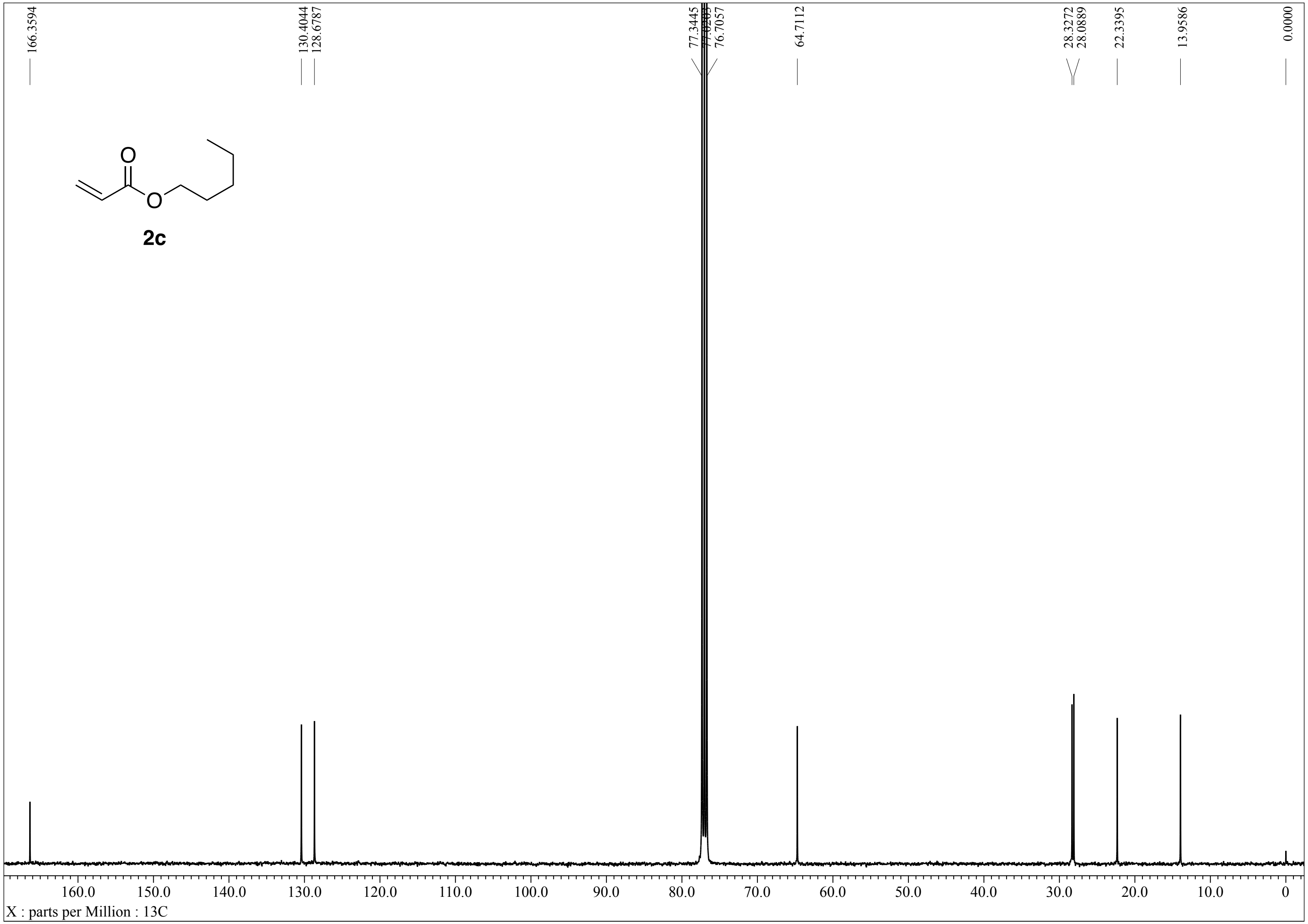

^{1}H NMR (400 MHz, $CDCl_3$)

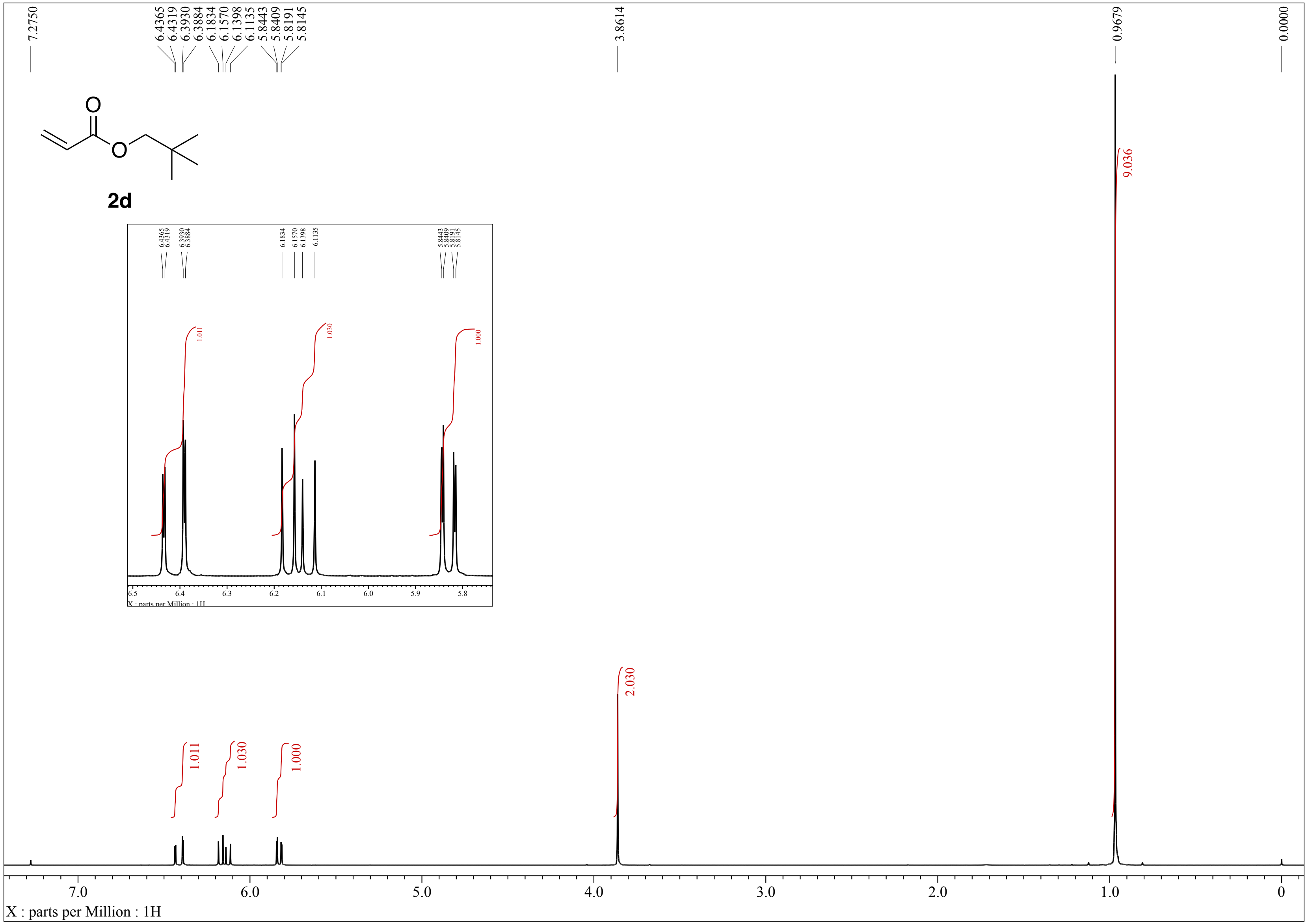

$^{13}C\{^1H\}$ NMR (100 MHz, $CDCl_3$)

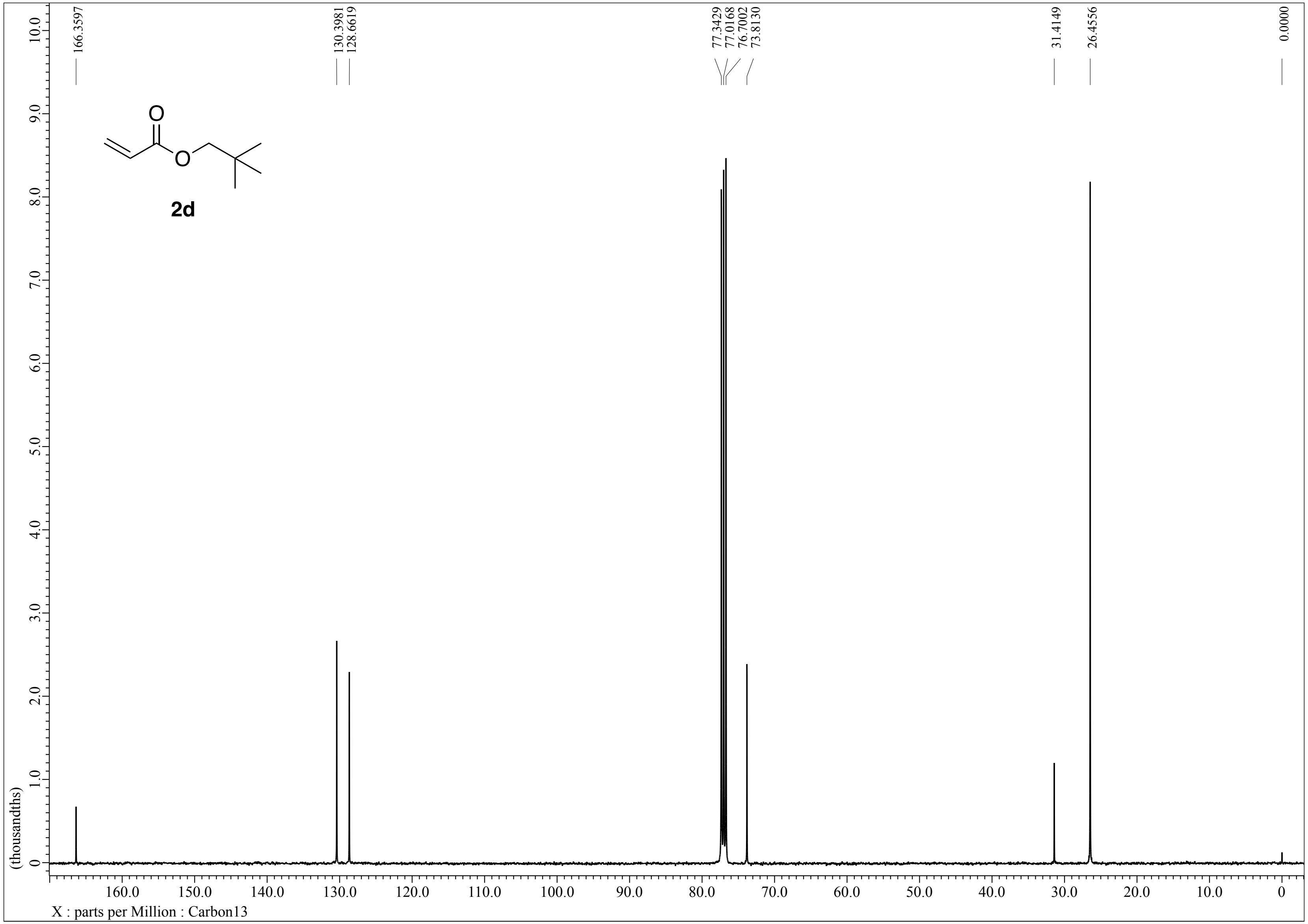

$^{13}C\{^{19}F\}$ NMR (100 MHz, $CDCl_3$)

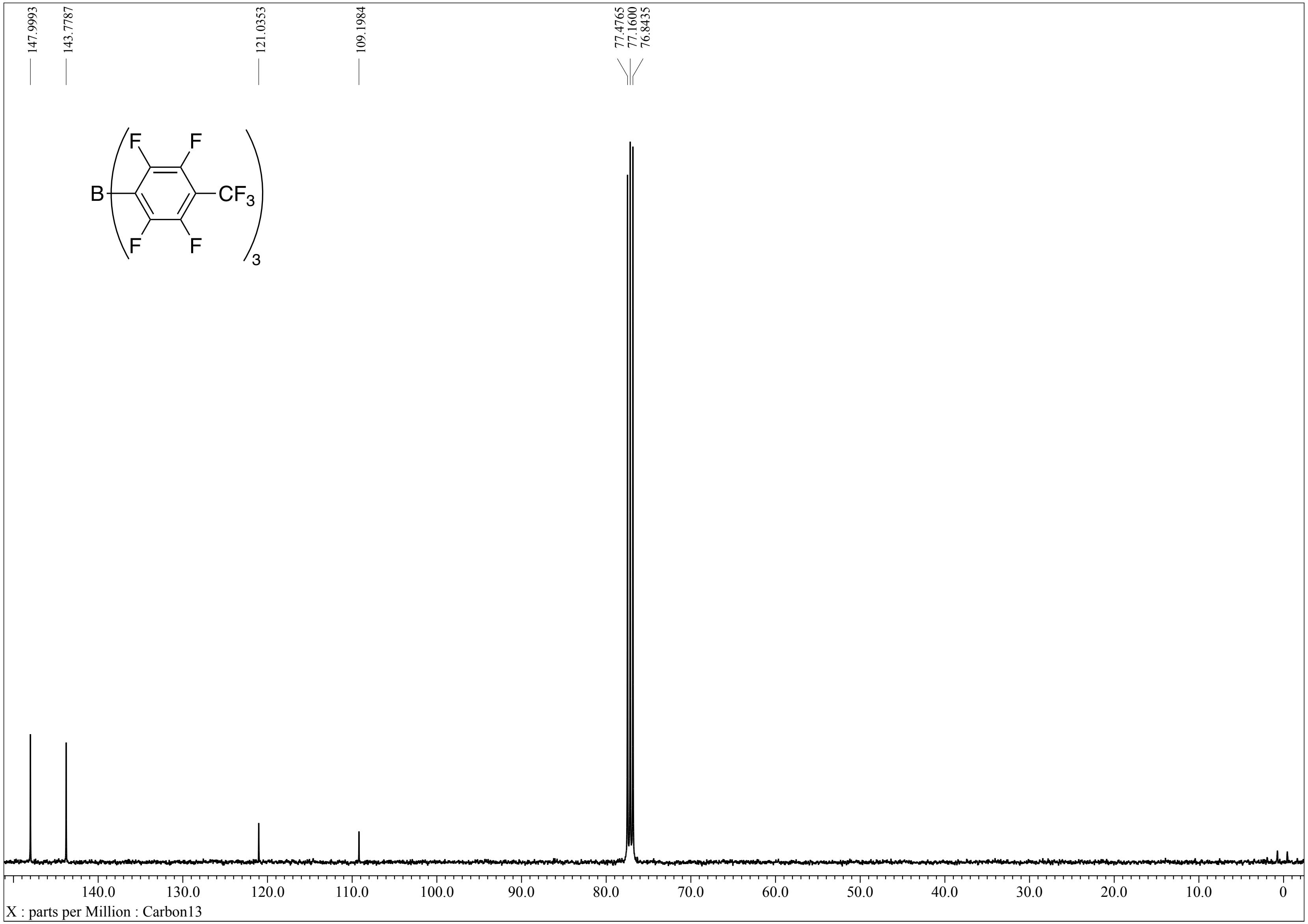

^{19}F NMR (376 MHz, $CDCl_3$)

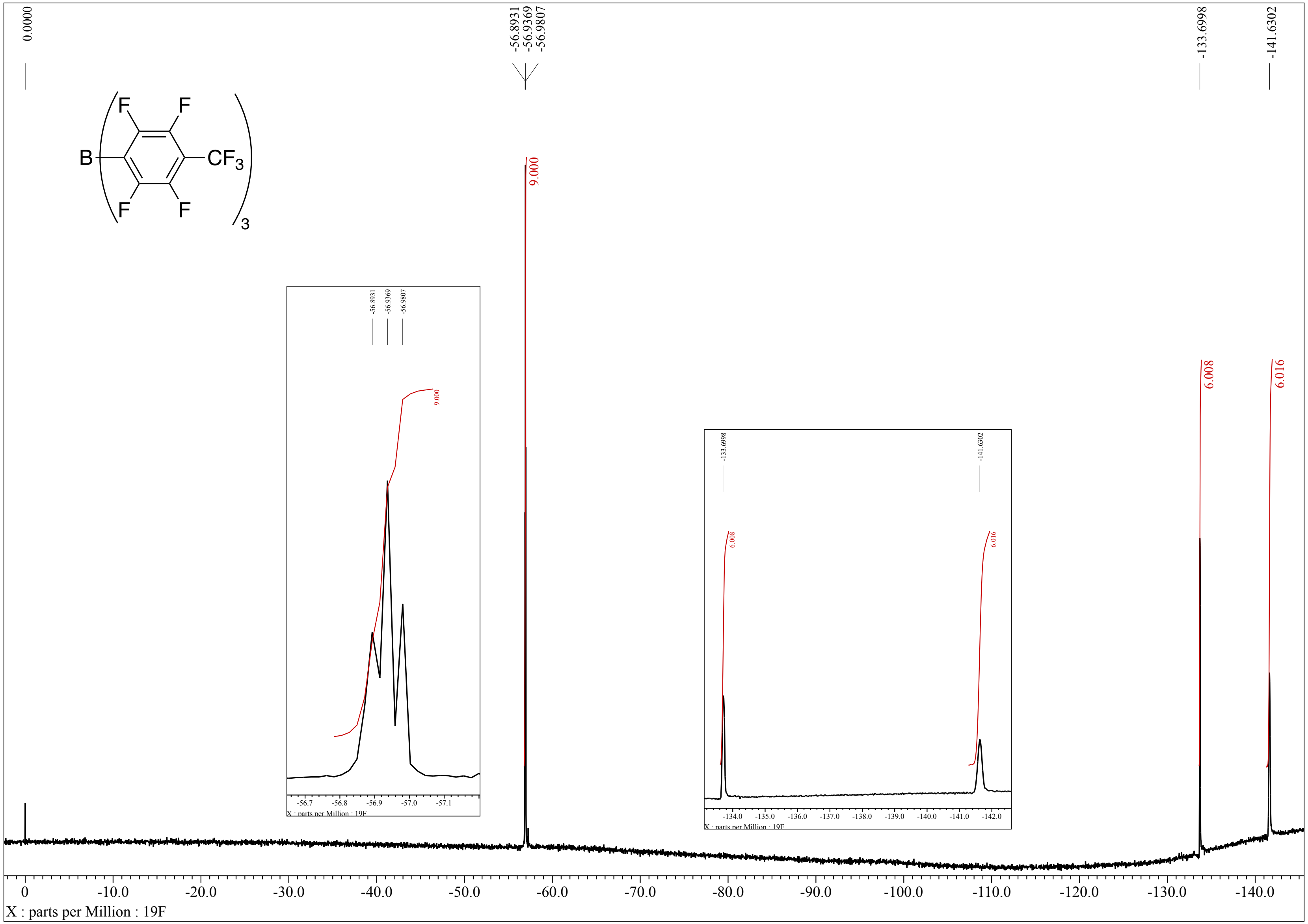

^{11}B NMR (160 MHz, $CDCl_3$)

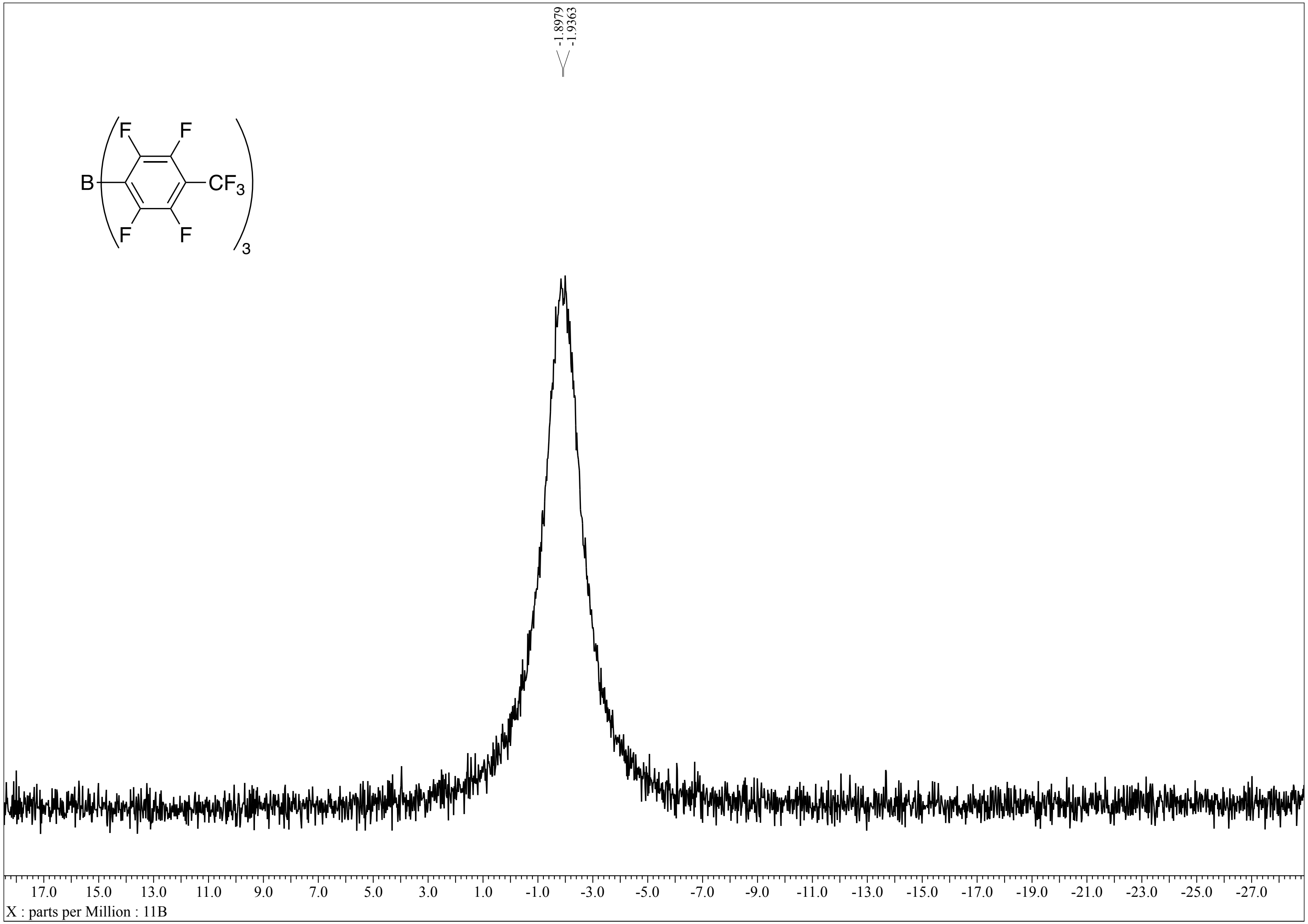

^{1}H NMR (400 MHz, $CDCl_3$)

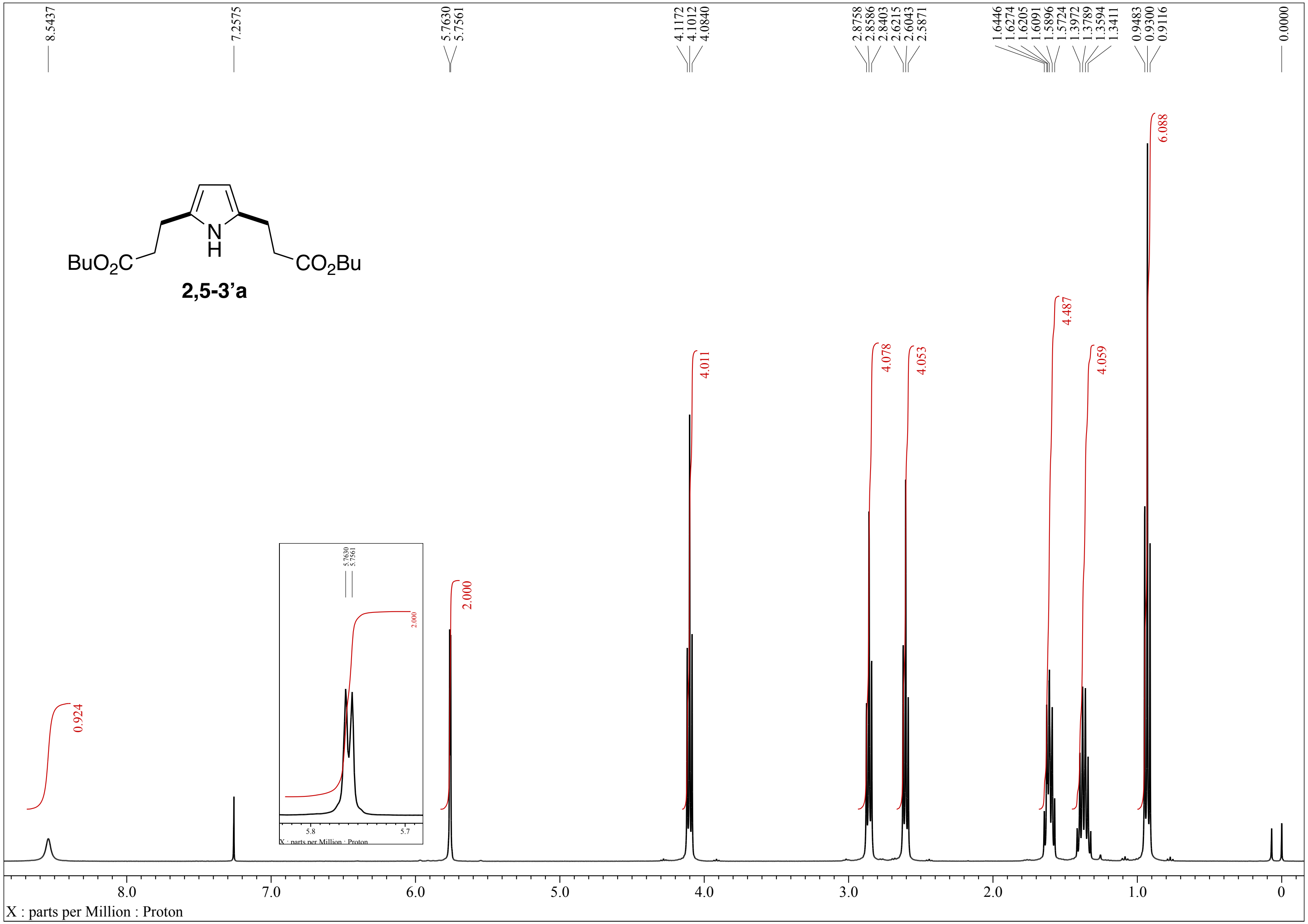

$^{13}C\{^{1}H\}$ NMR (100 MHz, $CDCl_3$)

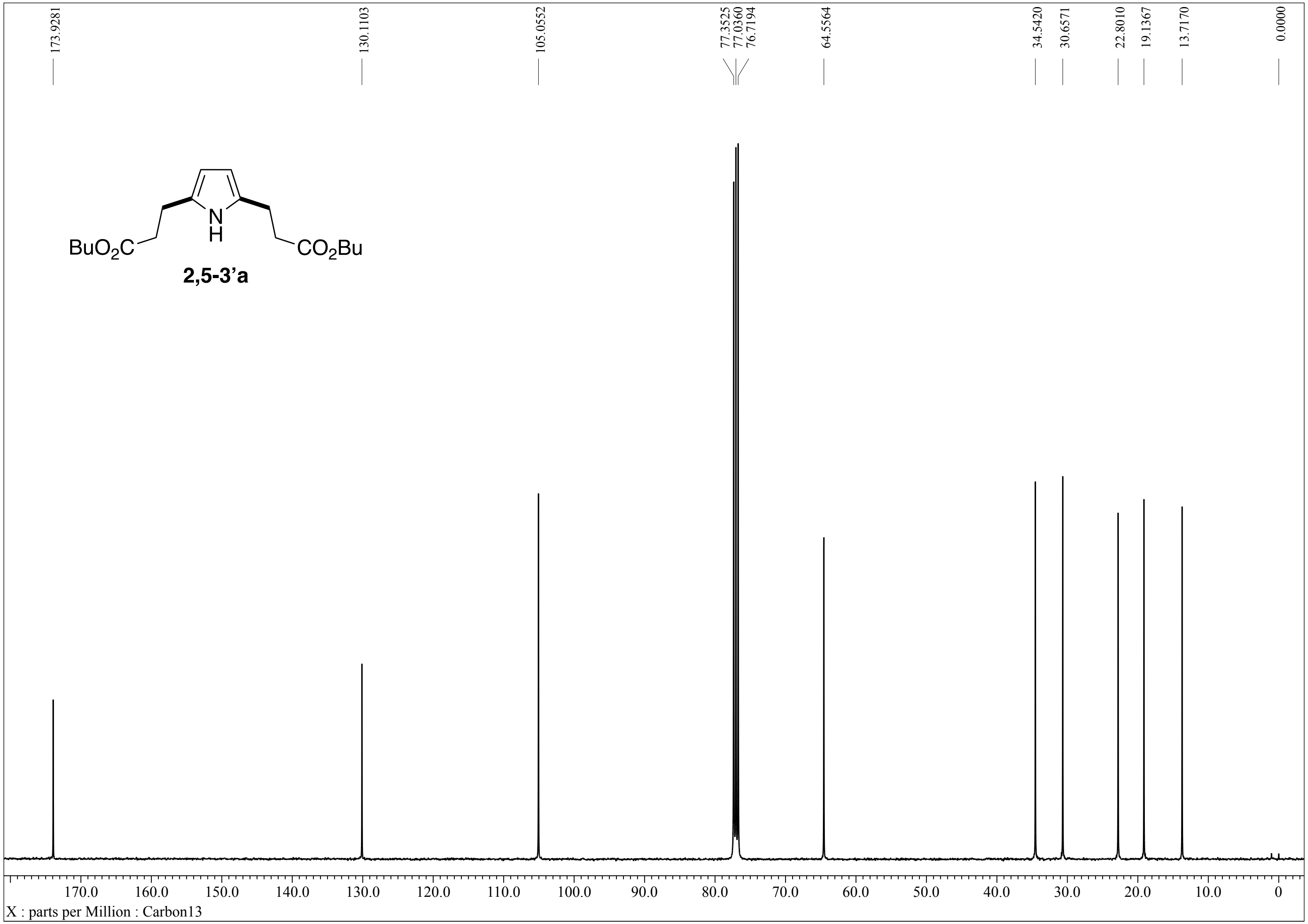

^{1}H NMR (500 MHz, $CDCl_3$)

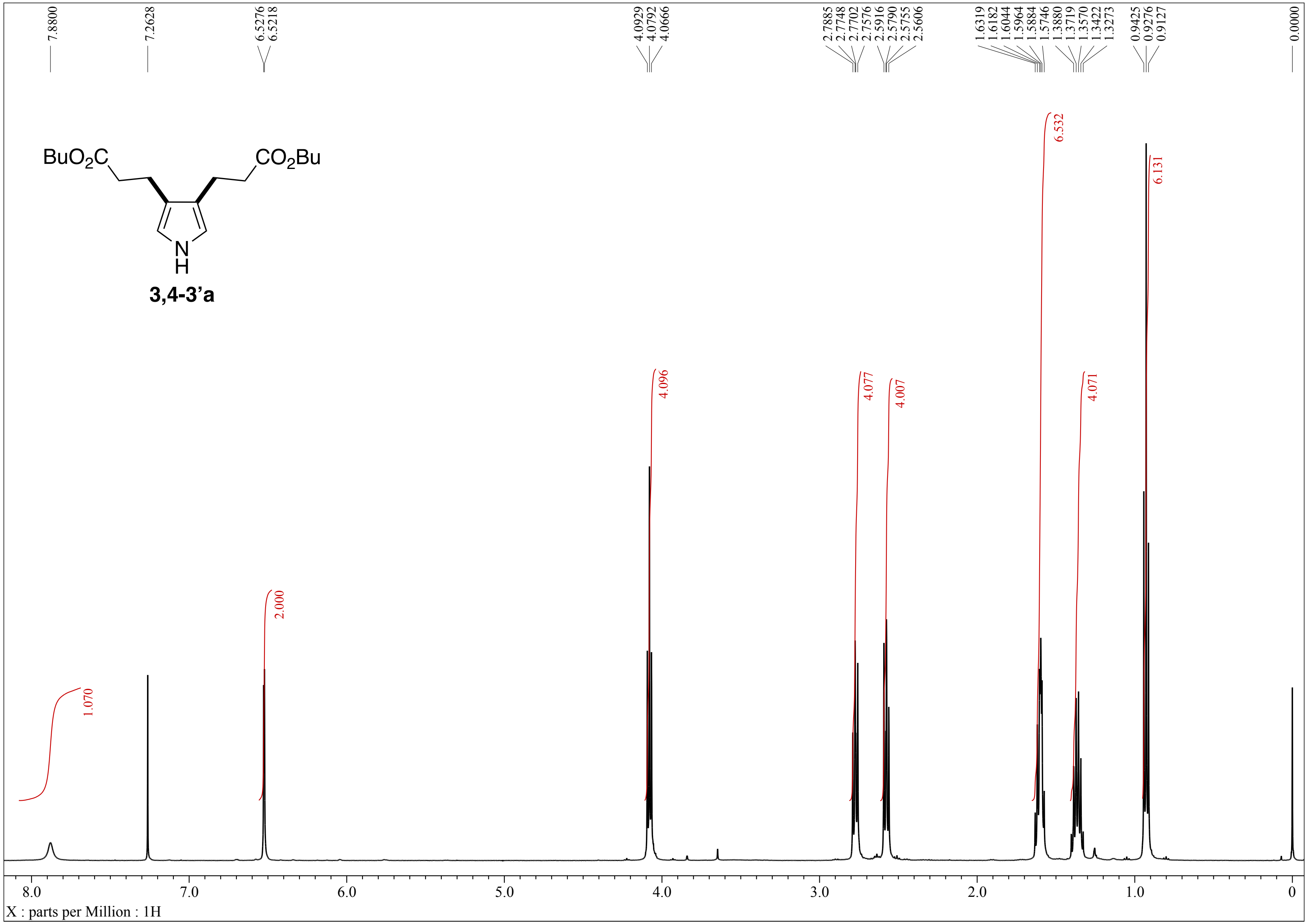

$^{13}C\{^{1}H\}$ NMR (125 MHz, $CDCl_3$)

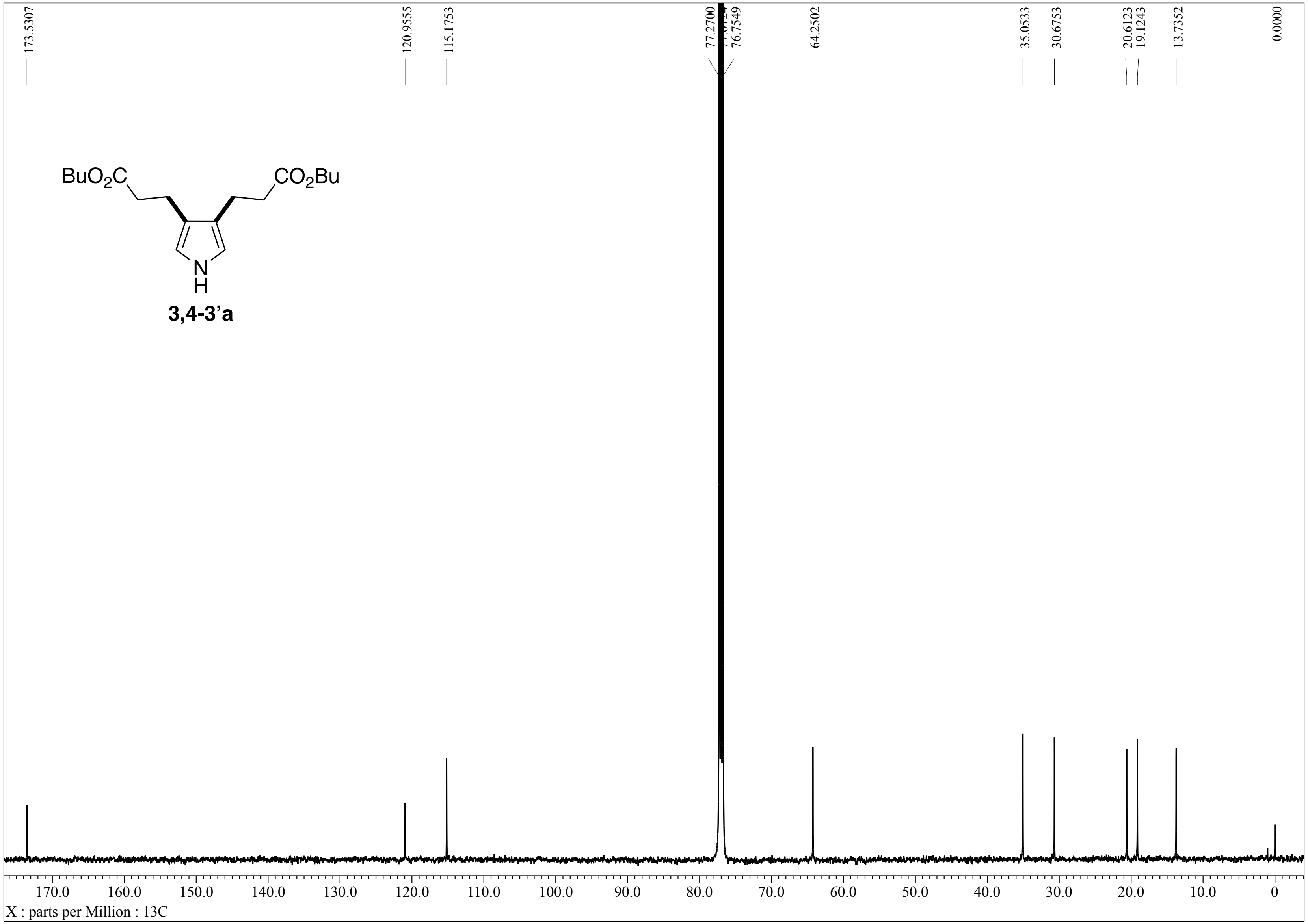

^{1}H NMR (400 MHz, $CDCl_3$)

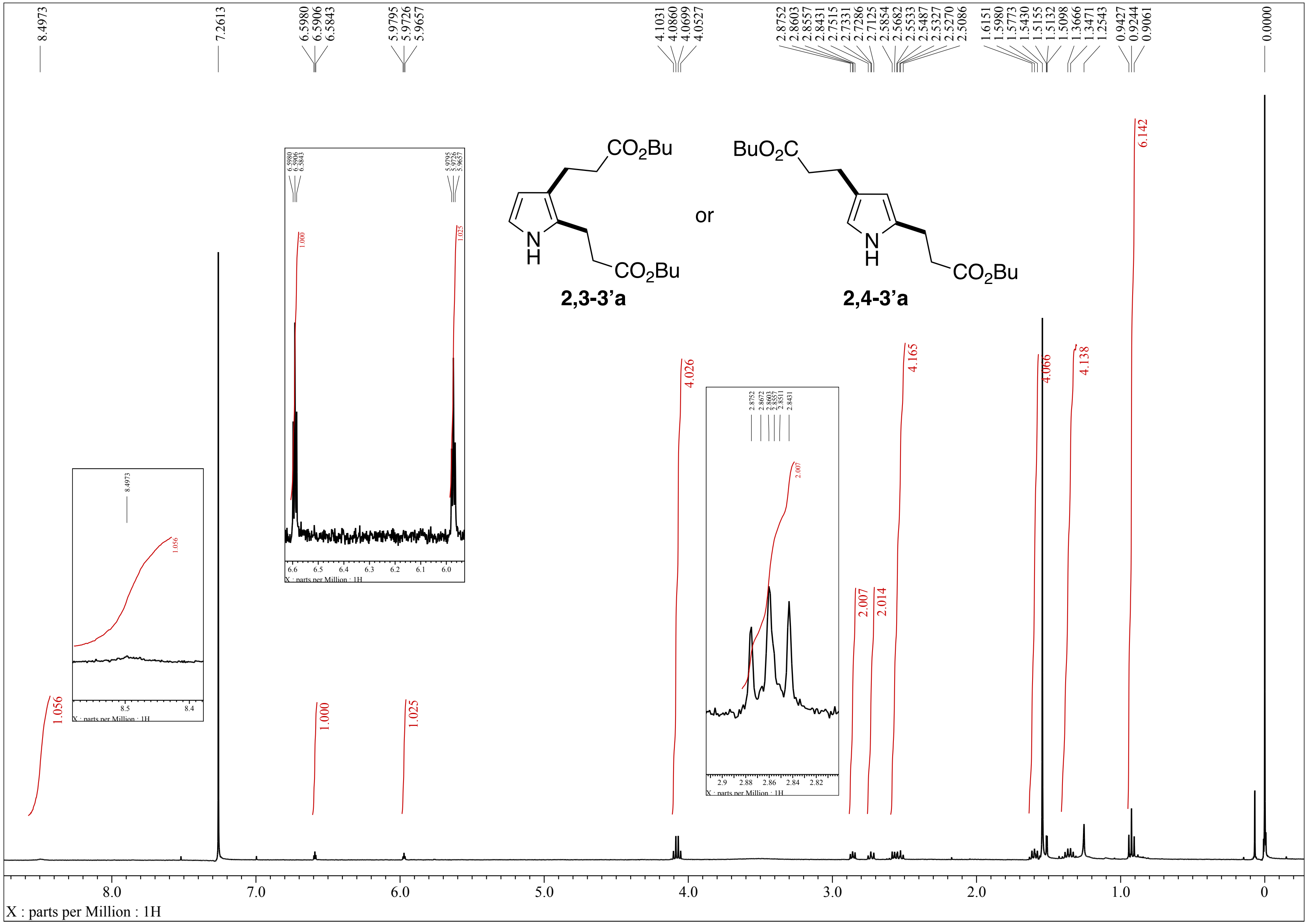

^{1}H NMR (400 MHz, $CDCl_3$)

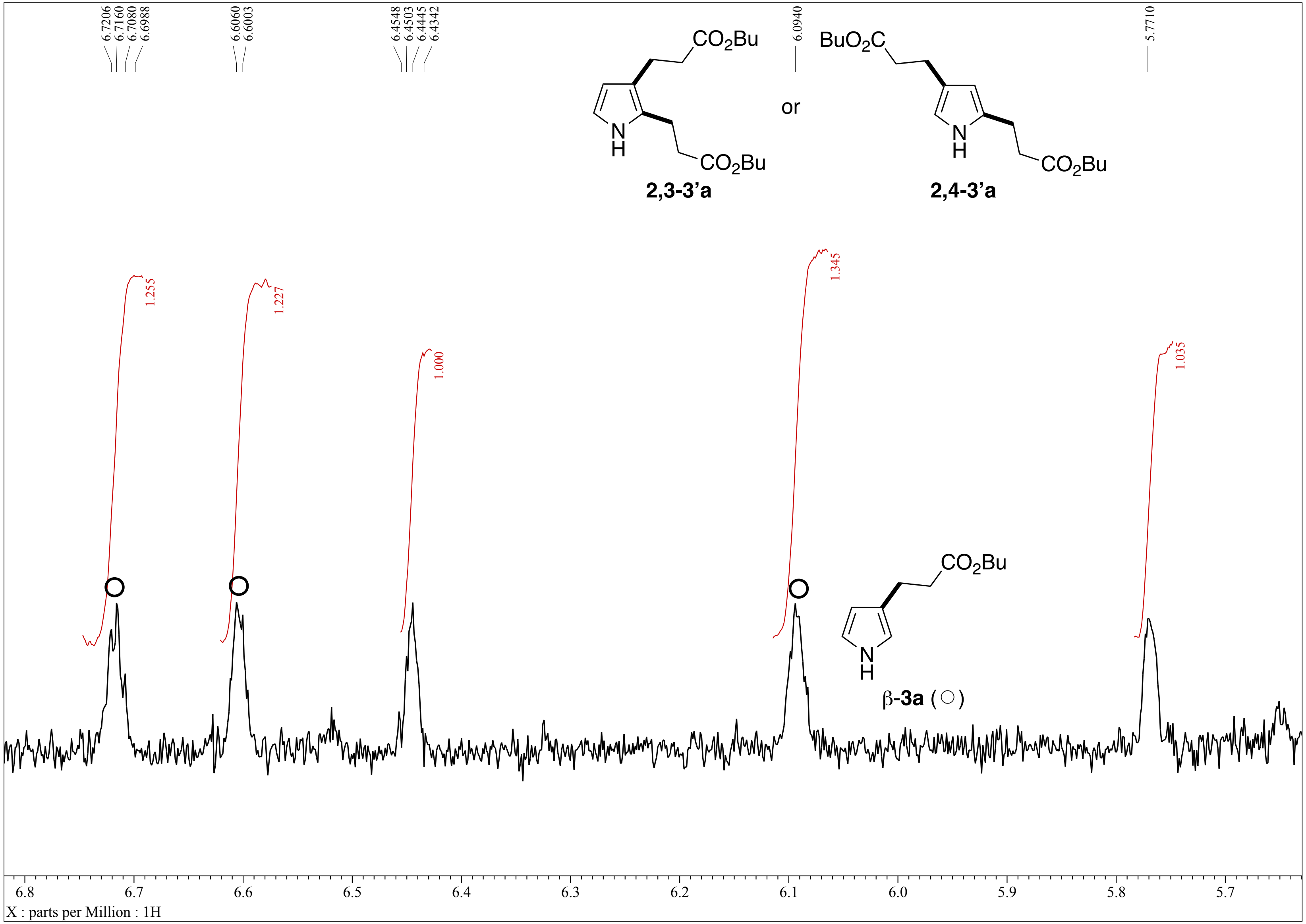

^{1}H NMR (400 MHz, $CDCl_3$)

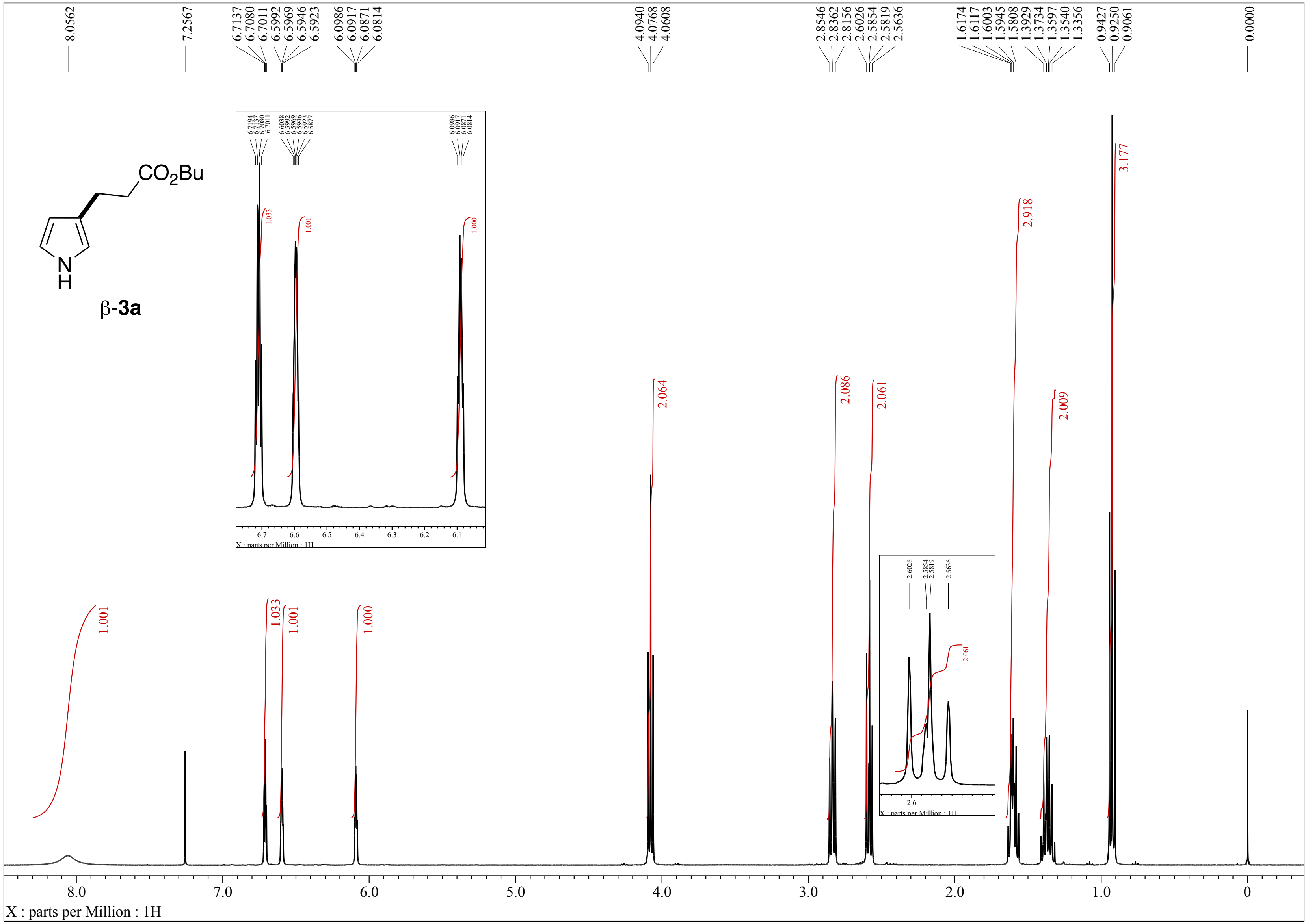

$^{13}C\{^1H\}$ NMR (125 MHz, $CDCl_3$)

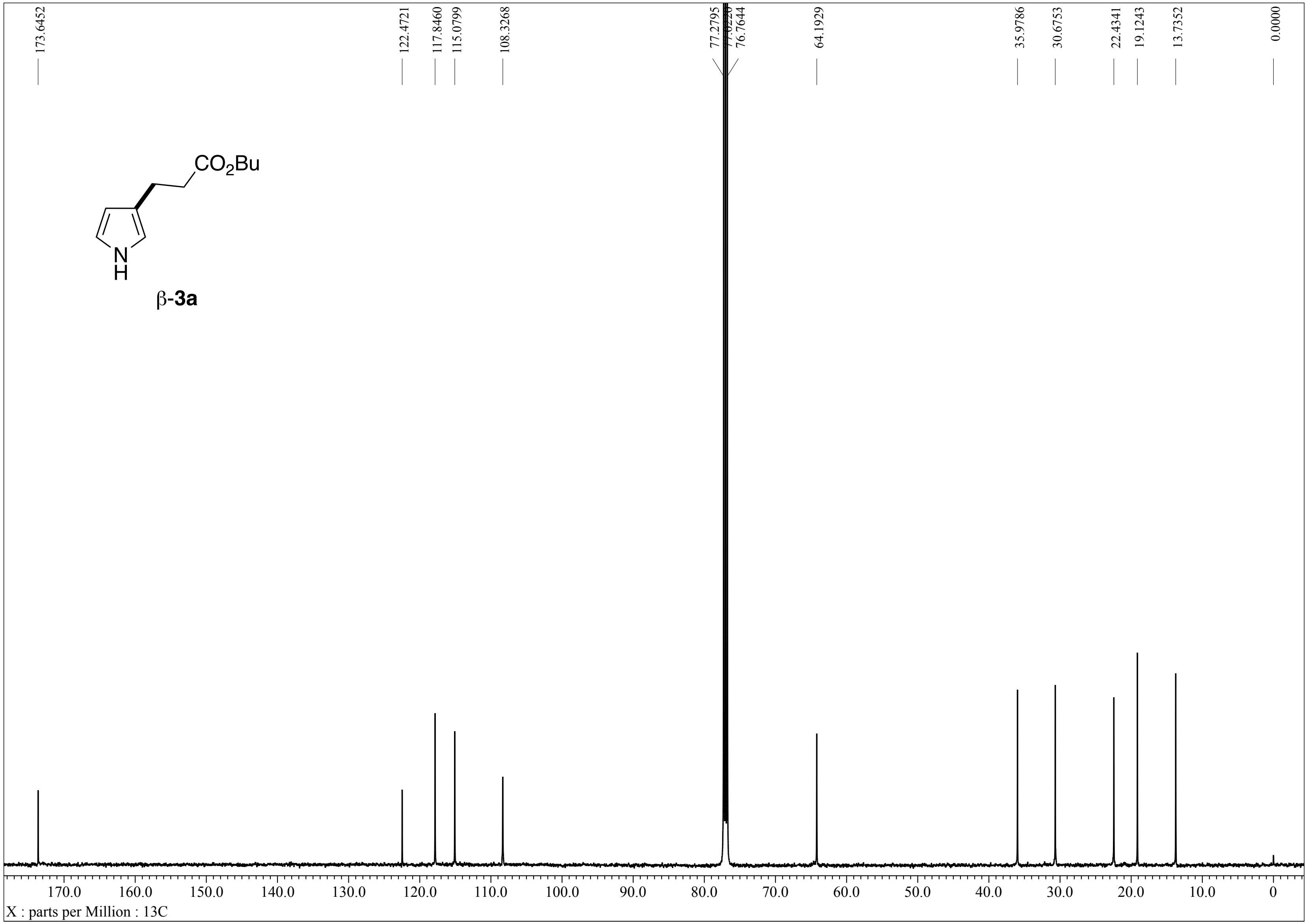

^{1}H NMR (500 MHz, $CDCl_3$)

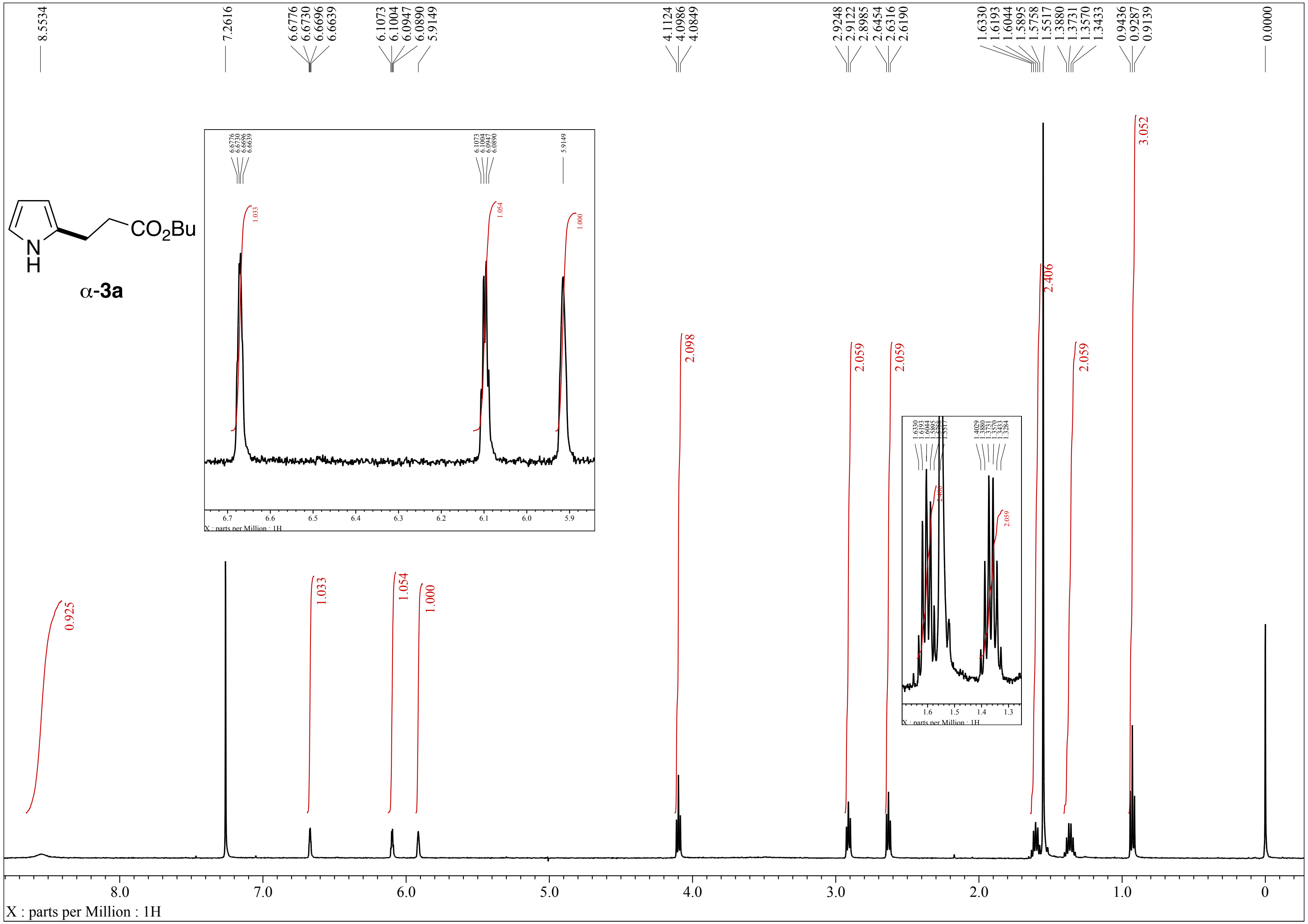

$^{13}C\{^1H\}$ NMR (125 MHz, $CDCl_3$)

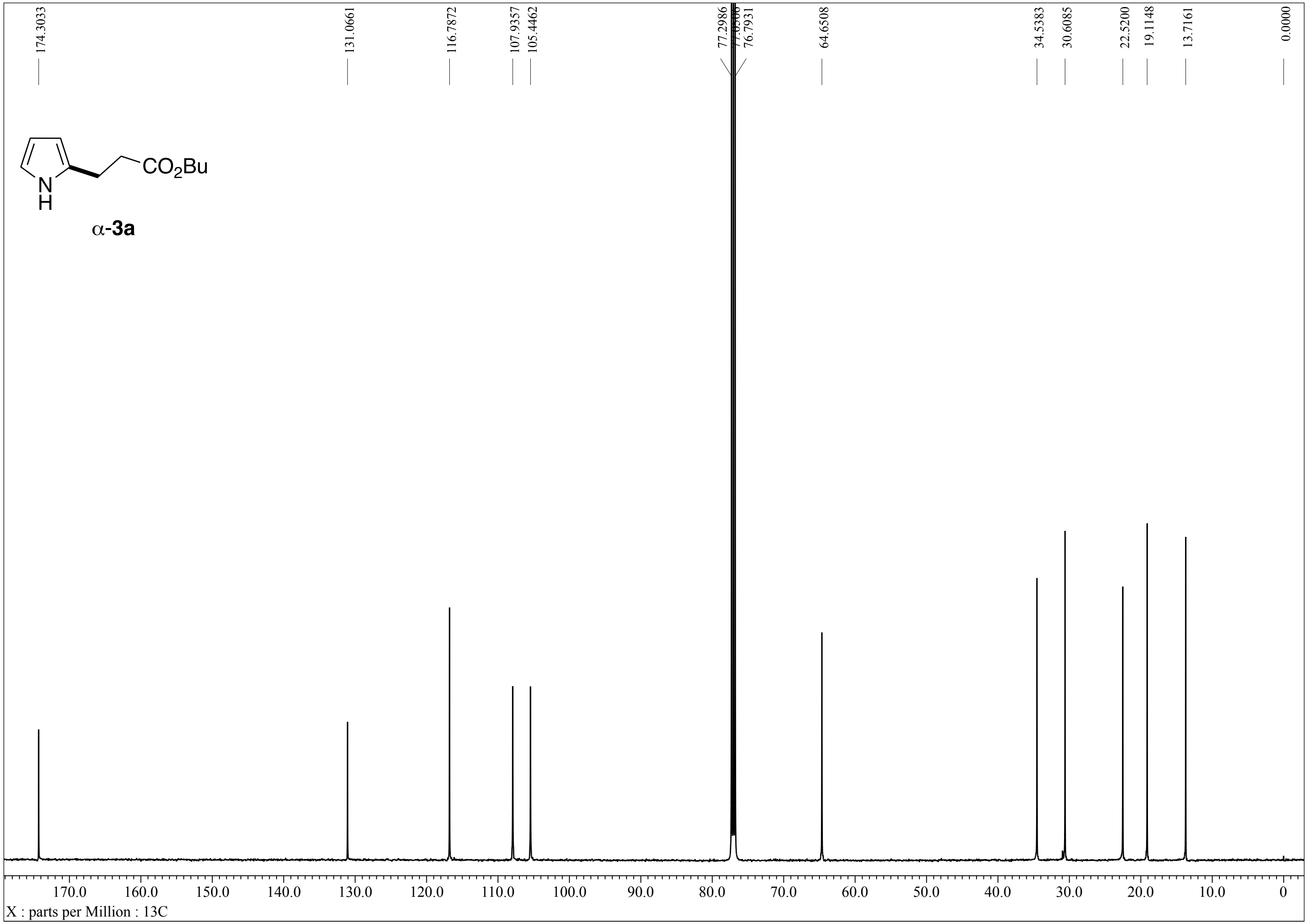

^{1}H NMR (500 MHz, $CDCl_3$)

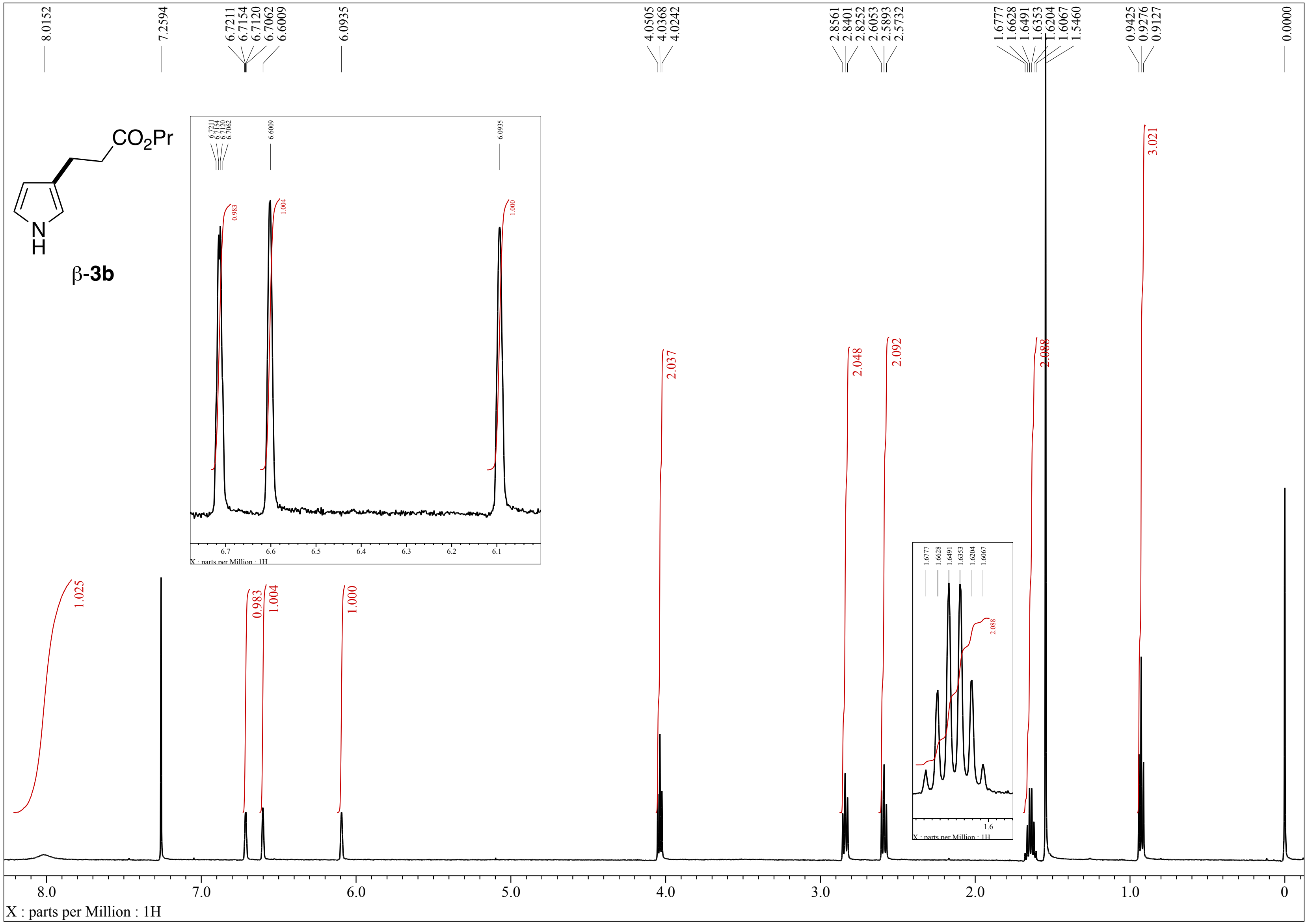

$^{13}C\{^1H\}$ NMR (125 MHz, $CDCl_3$)

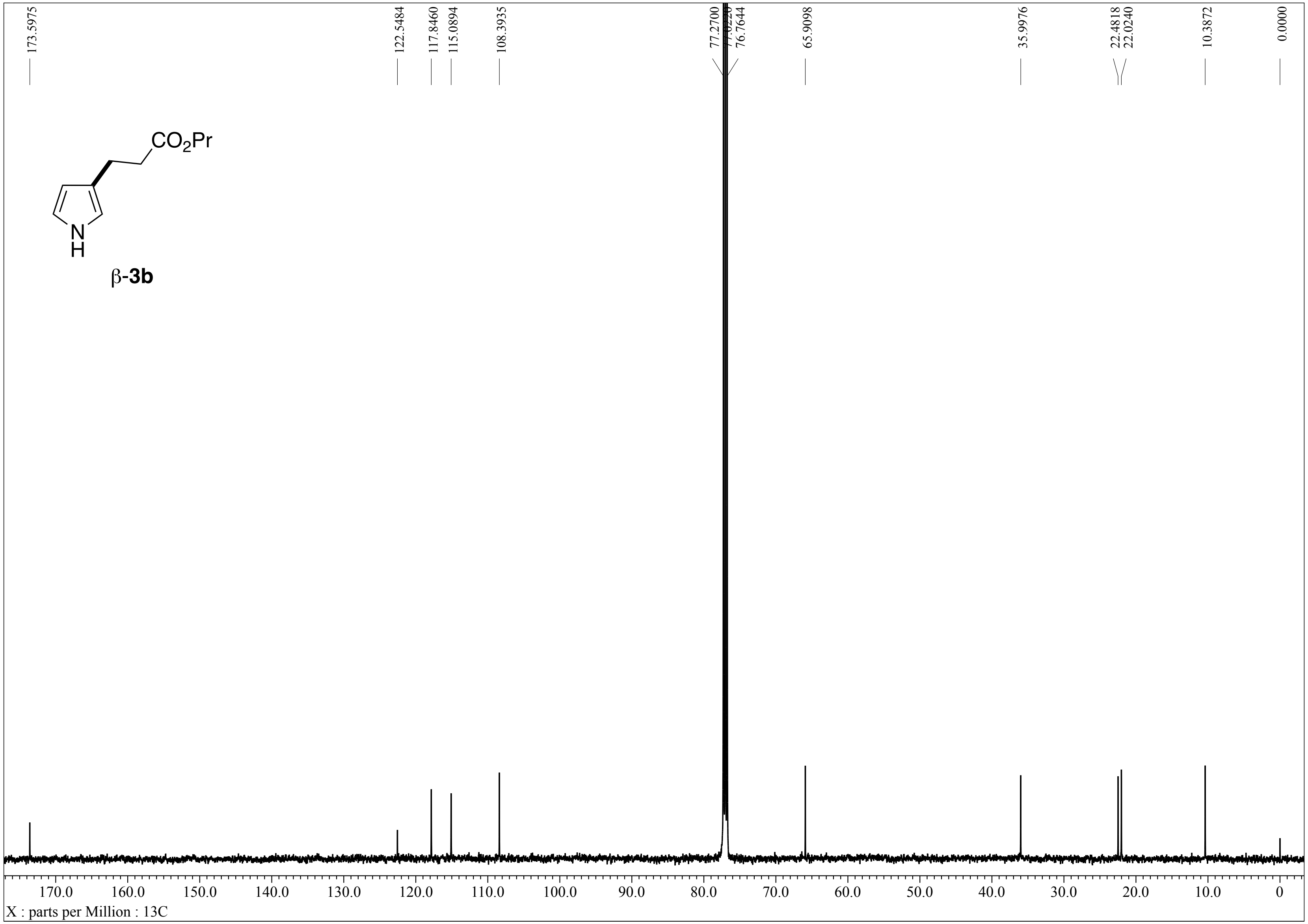

^1H NMR (400 MHz, $CDCl_3$)

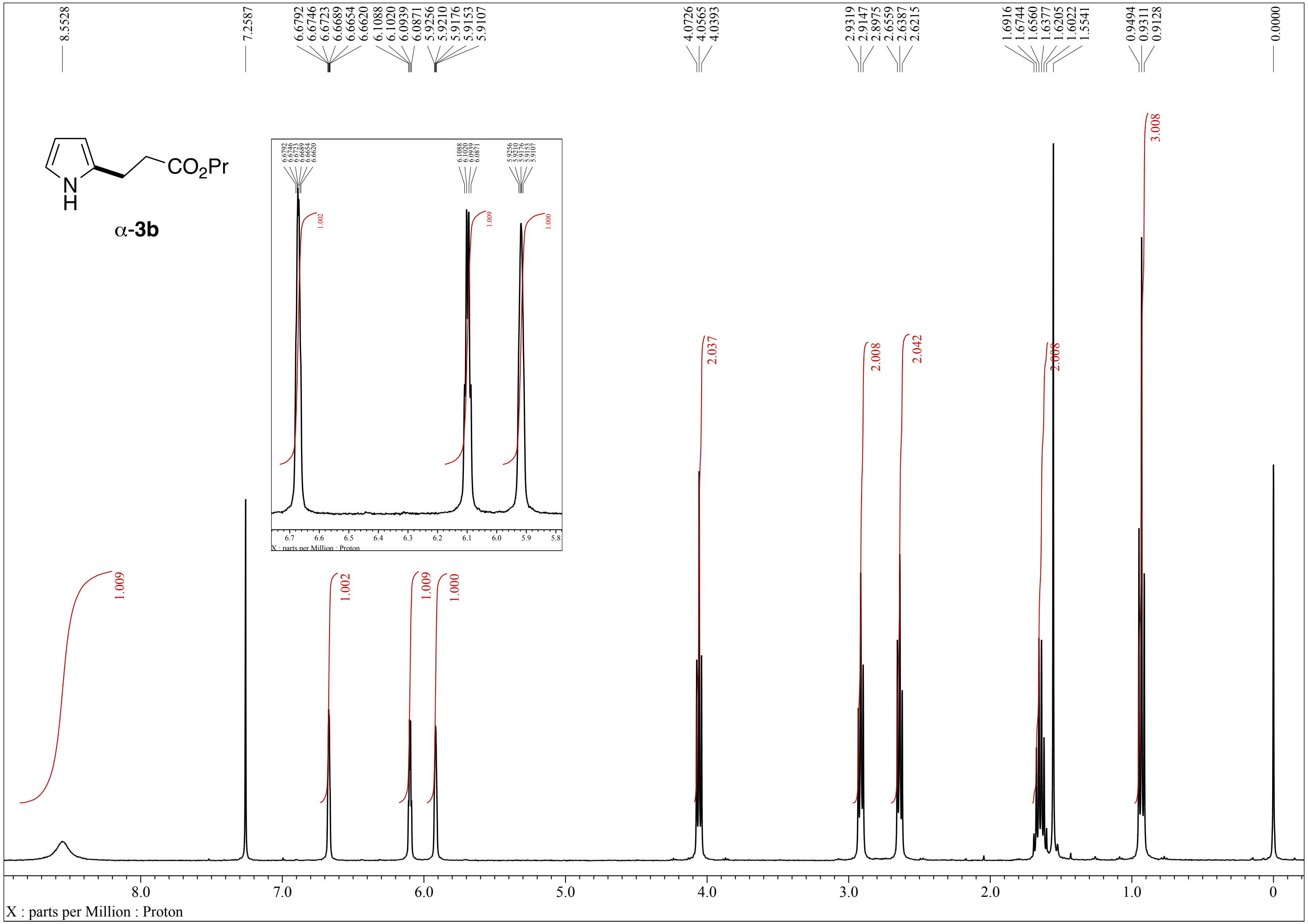

$^{13}C\{^1H\}$ NMR (100 MHz, $CDCl_3$)

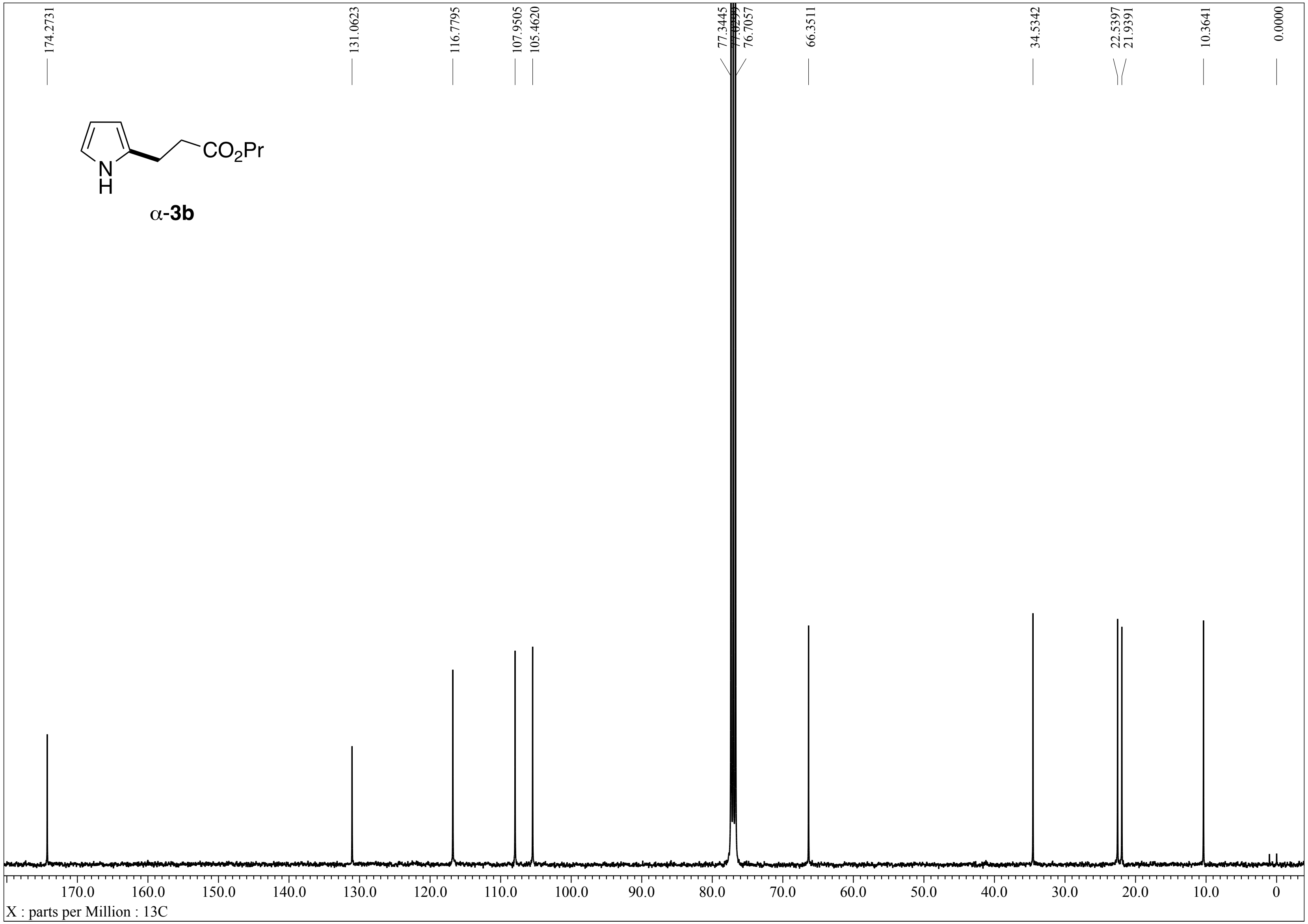

^{1}H NMR (400 MHz, $CDCl_3$)

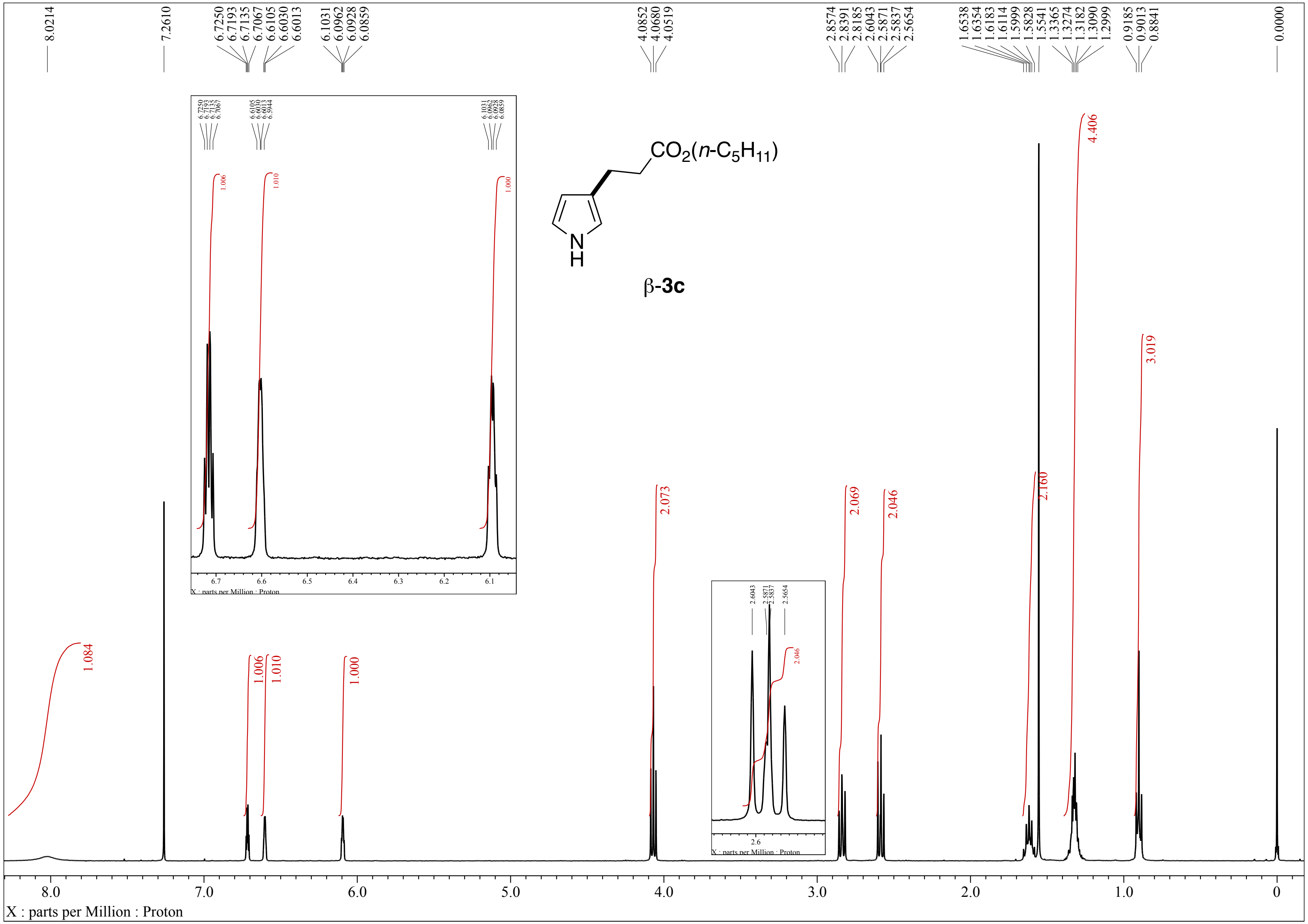

$^{13}C\{^{1}H\}$ NMR (125 MHz, $CDCl_3$)

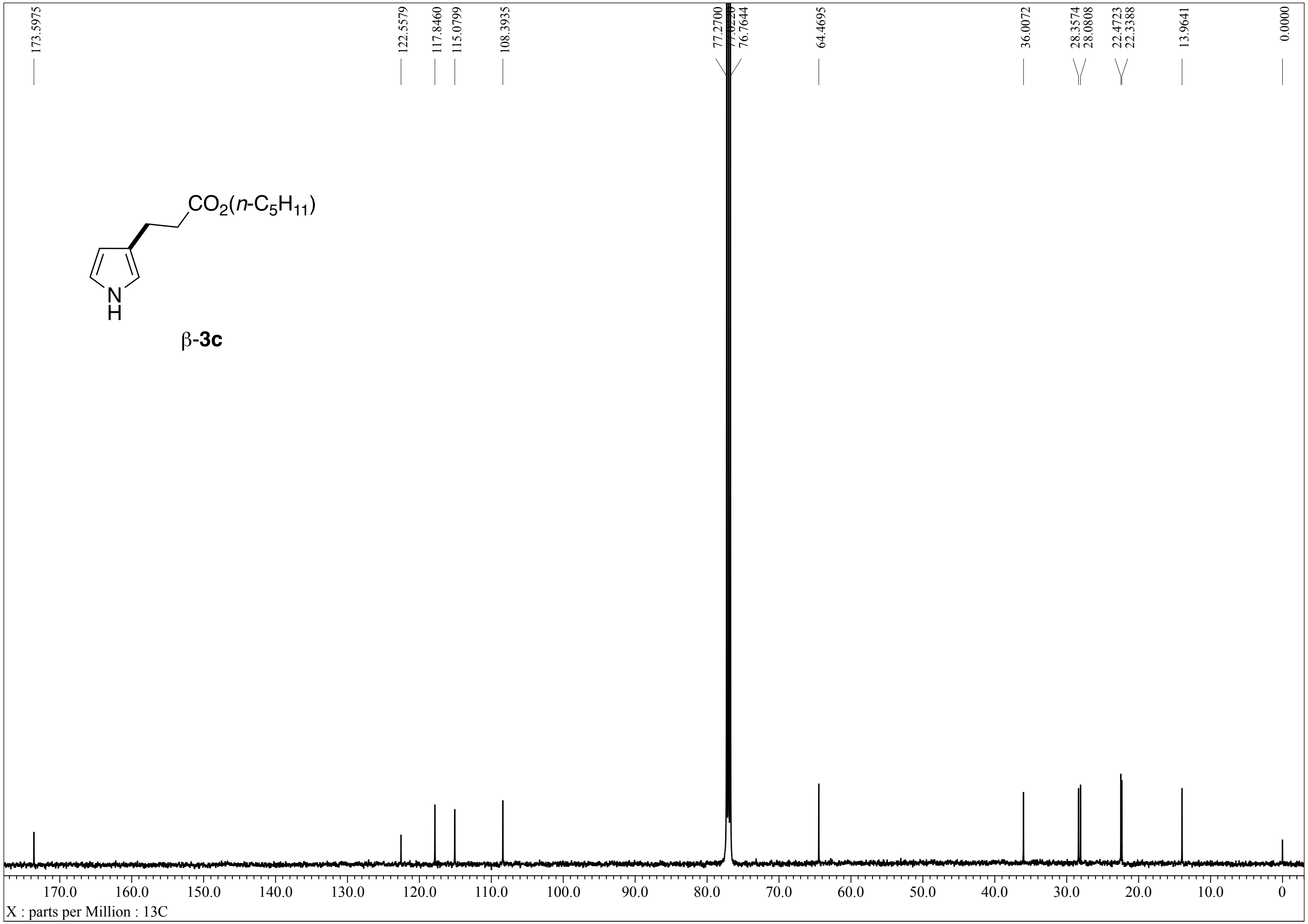

^{1}H NMR (500 MHz, $CDCl_3$)

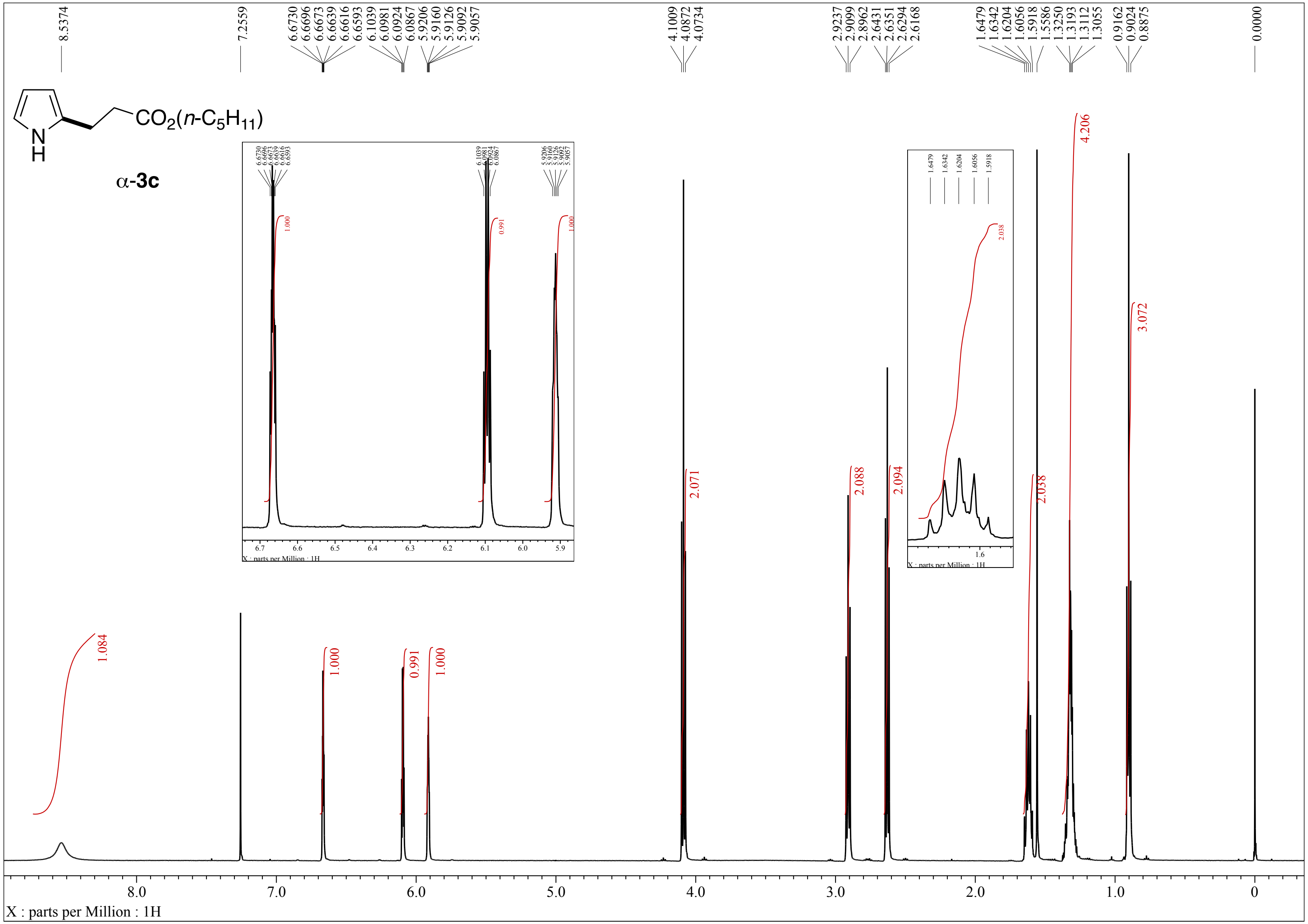

$^{13}C\{^1H\}$ NMR (100 MHz, $CDCl_3$)

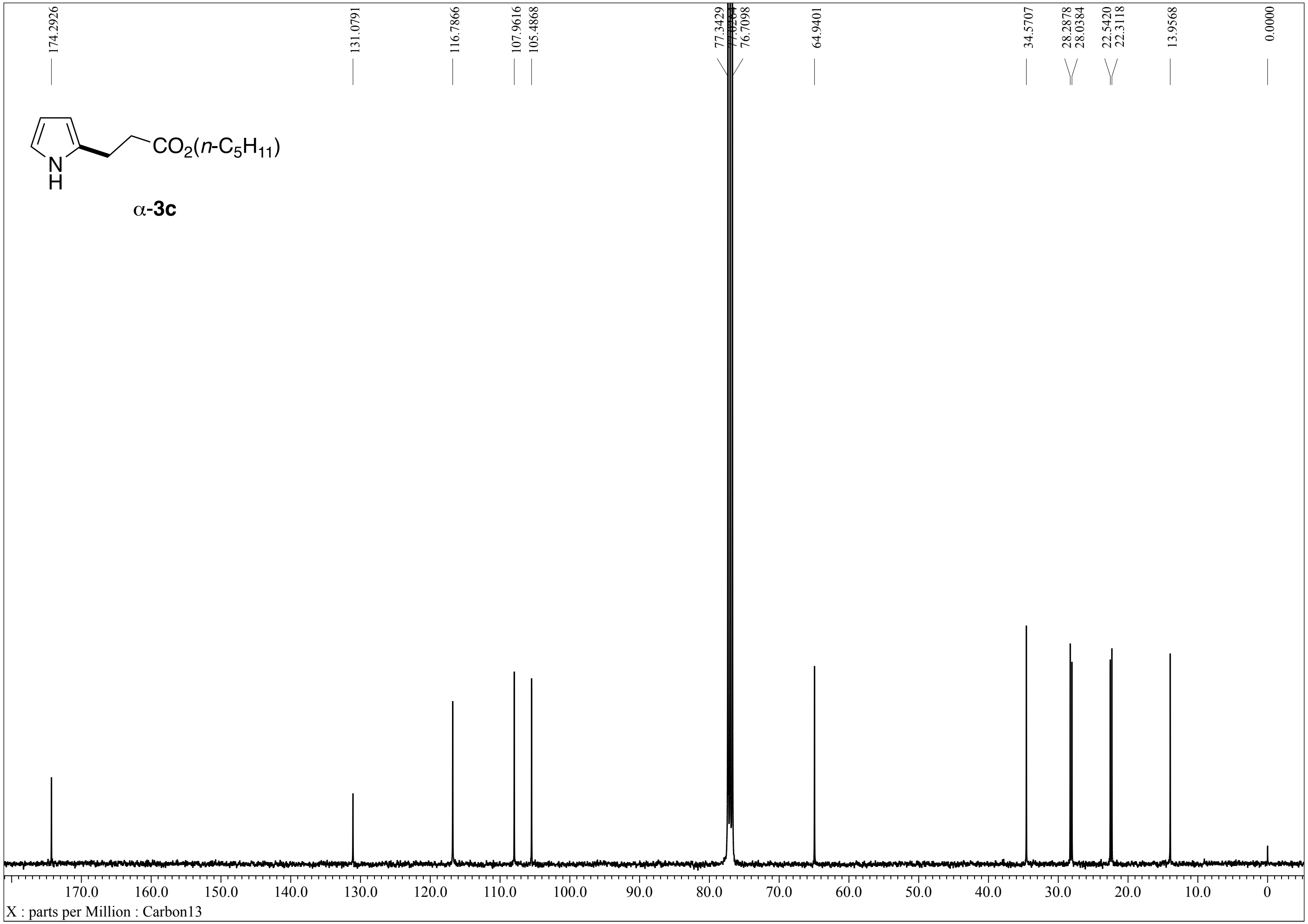

^{1}H NMR (400 MHz, $CDCl_3$)

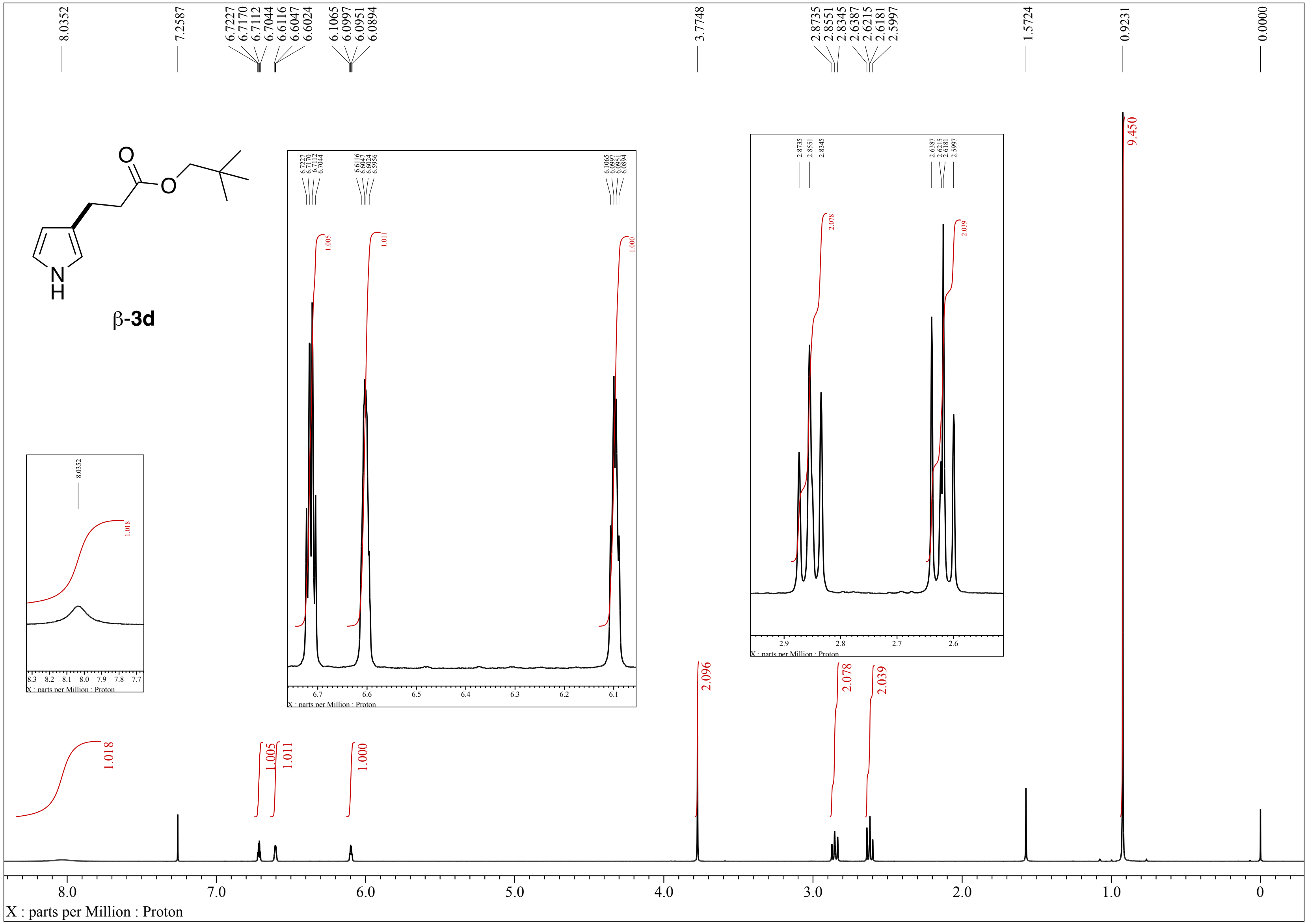

$^{13}C\{^{1}H\}$ NMR (125 MHz, $CDCl_3$)

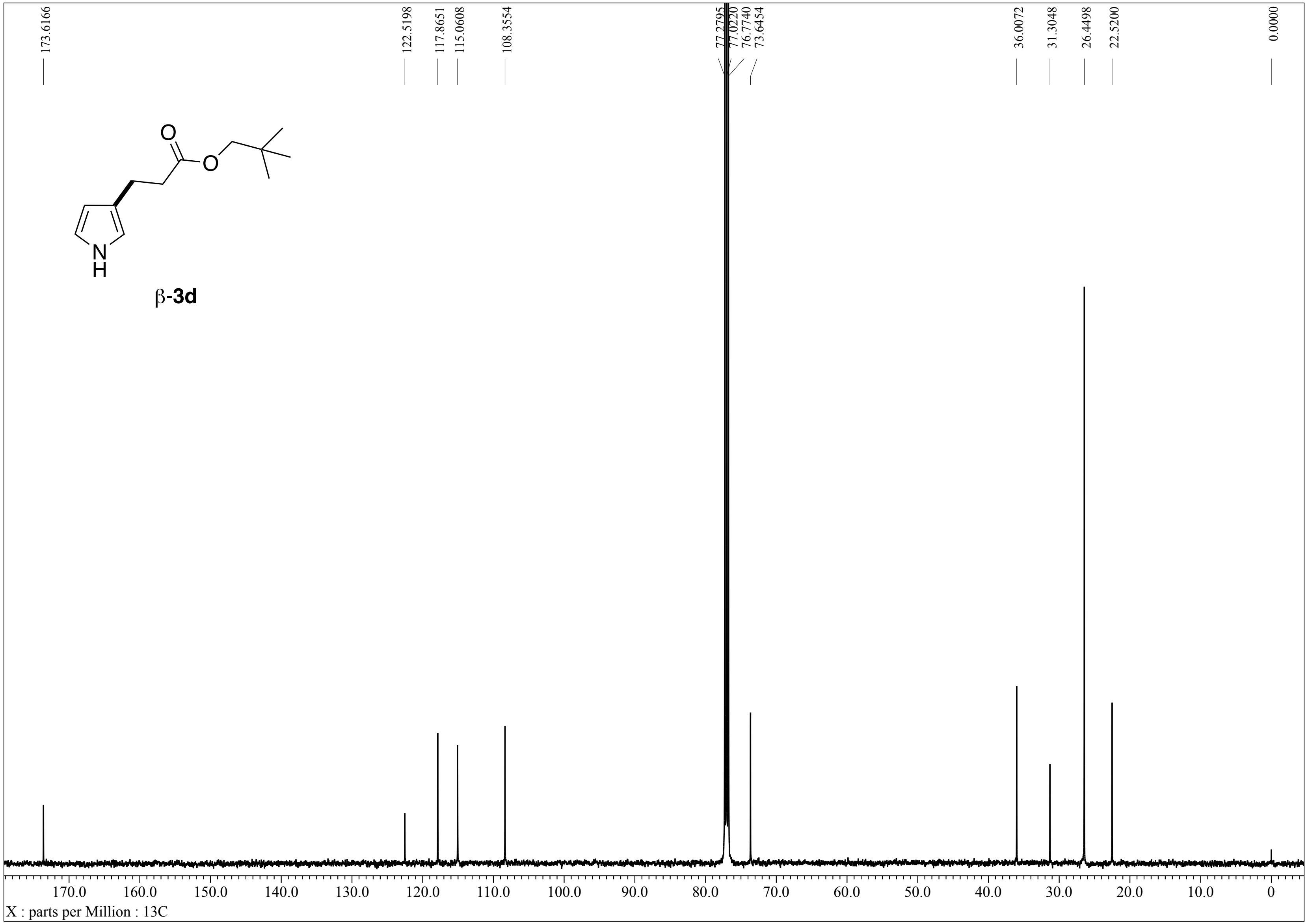

^{1}H NMR (400 MHz, $CDCl_3$)

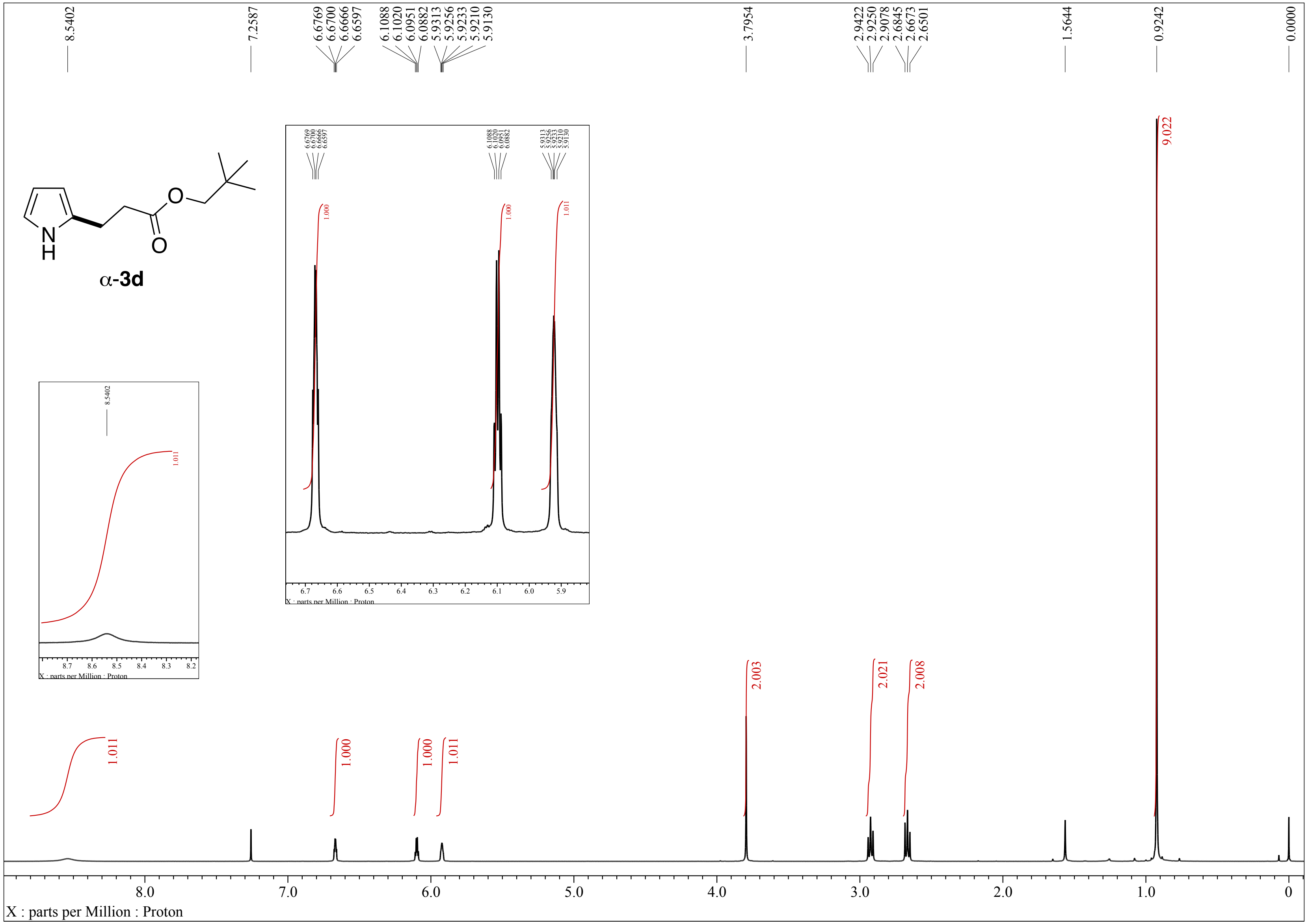

$^{13}C\{^{1}H\}$ NMR (100 MHz, $CDCl_3$)

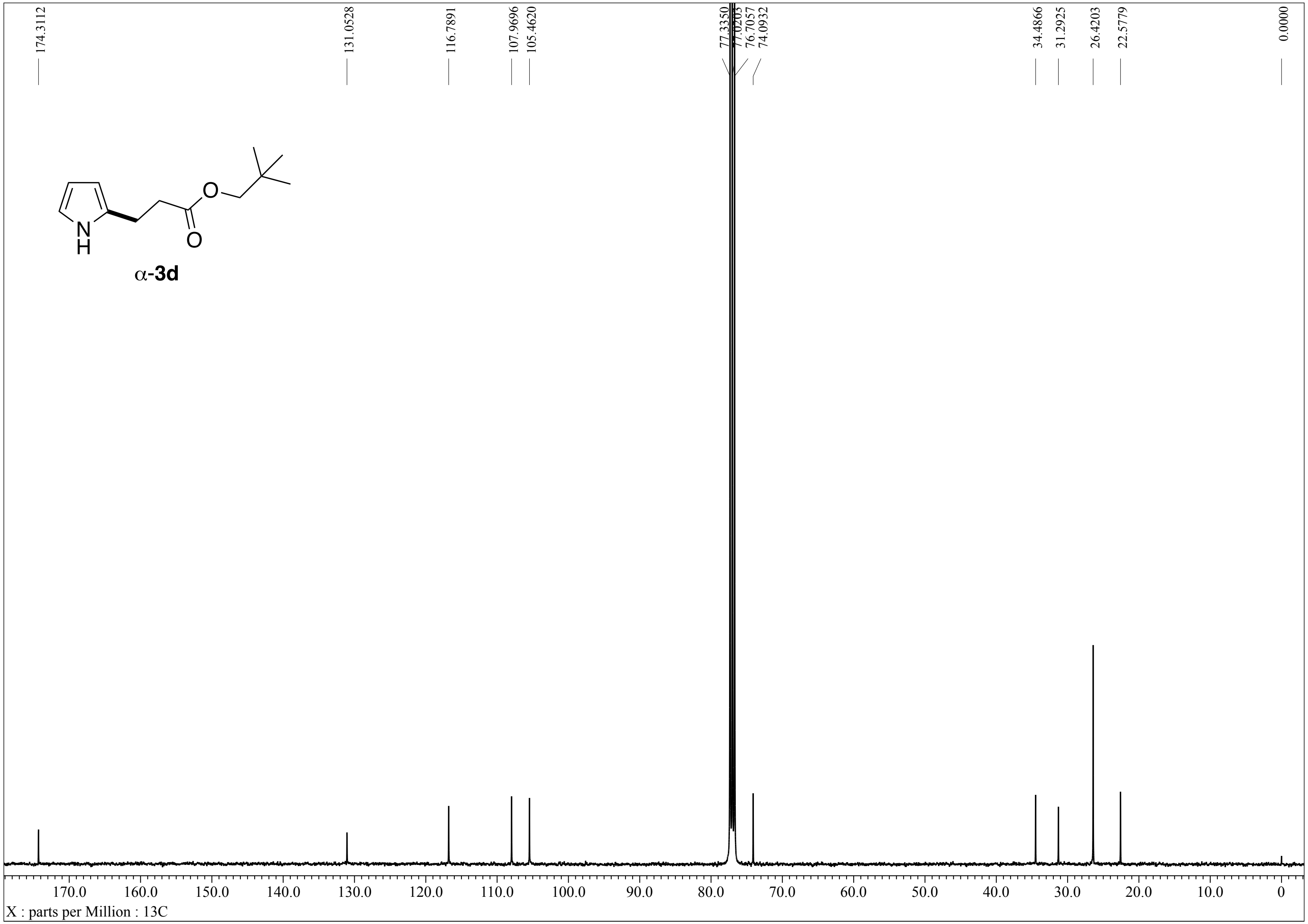

^{1}H NMR (500 MHz, $CDCl_3$)

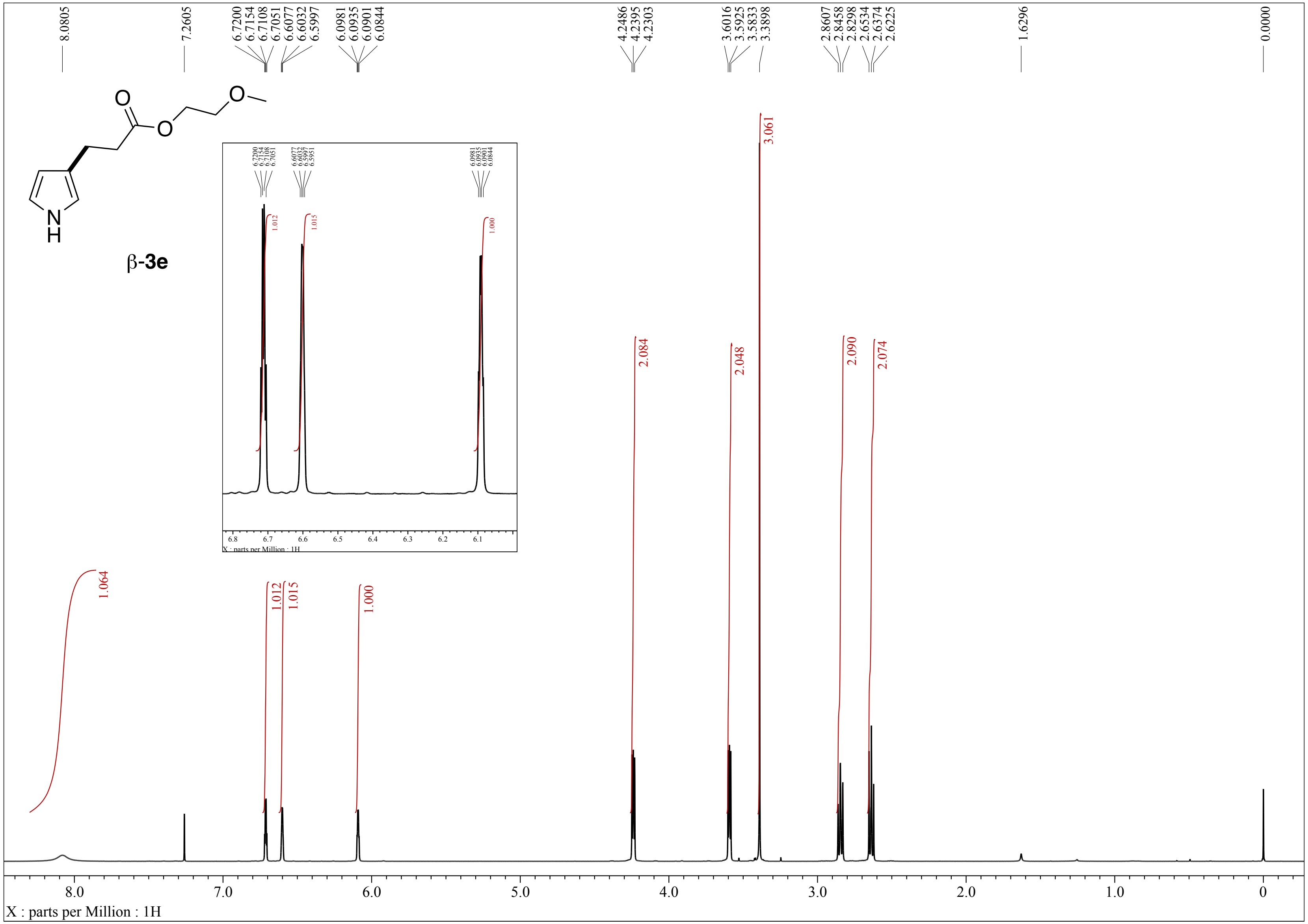

$^{13}C\{^{1}H\}$ NMR (125 MHz, $CDCl_3$)

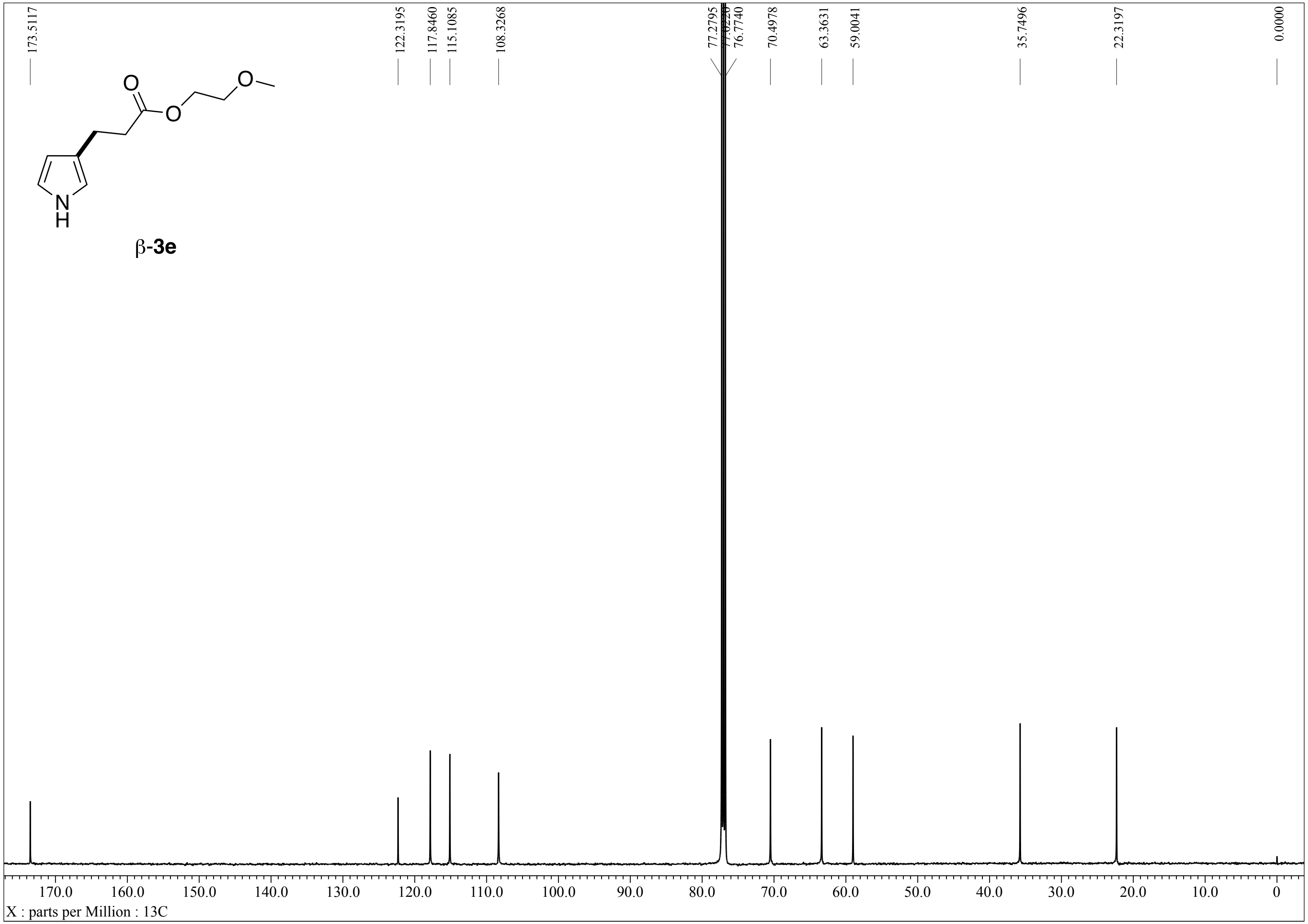

^{1}H NMR (400 MHz, $CDCl_3$)

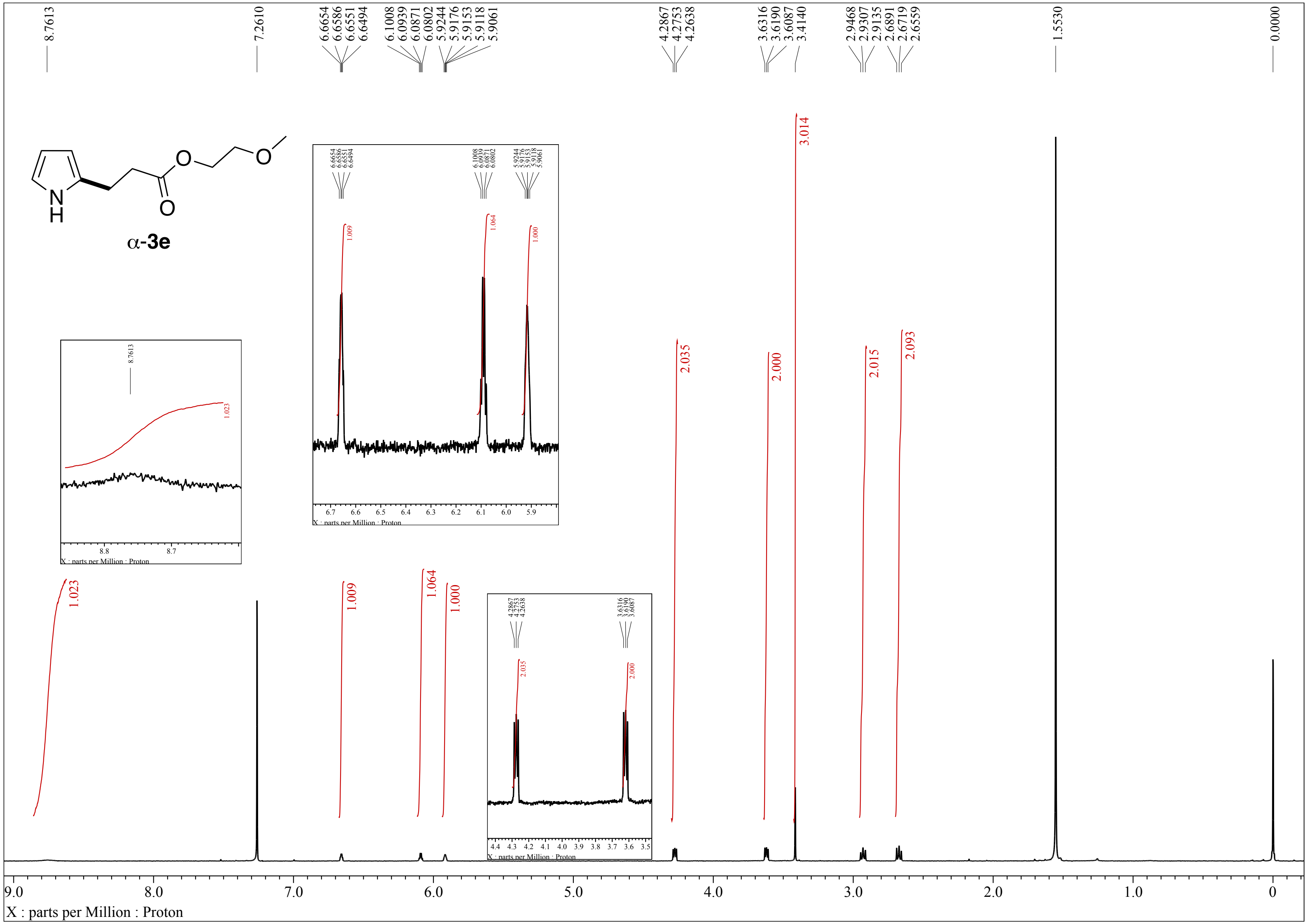

$^{13}C\{^{1}H\}$ NMR (125 MHz, $CDCl_3$)

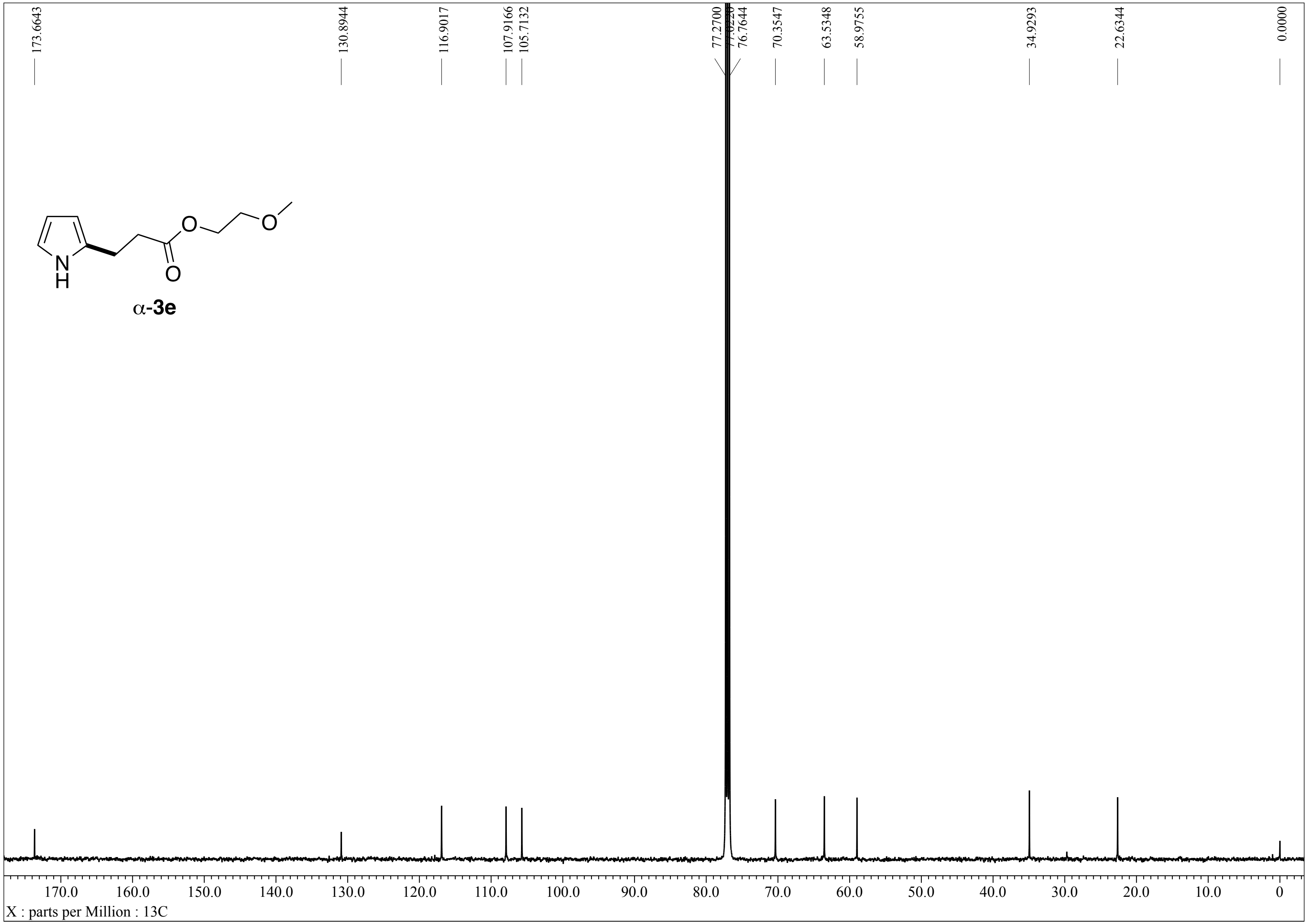

^{1}H NMR (500 MHz, $CDCl_3$)

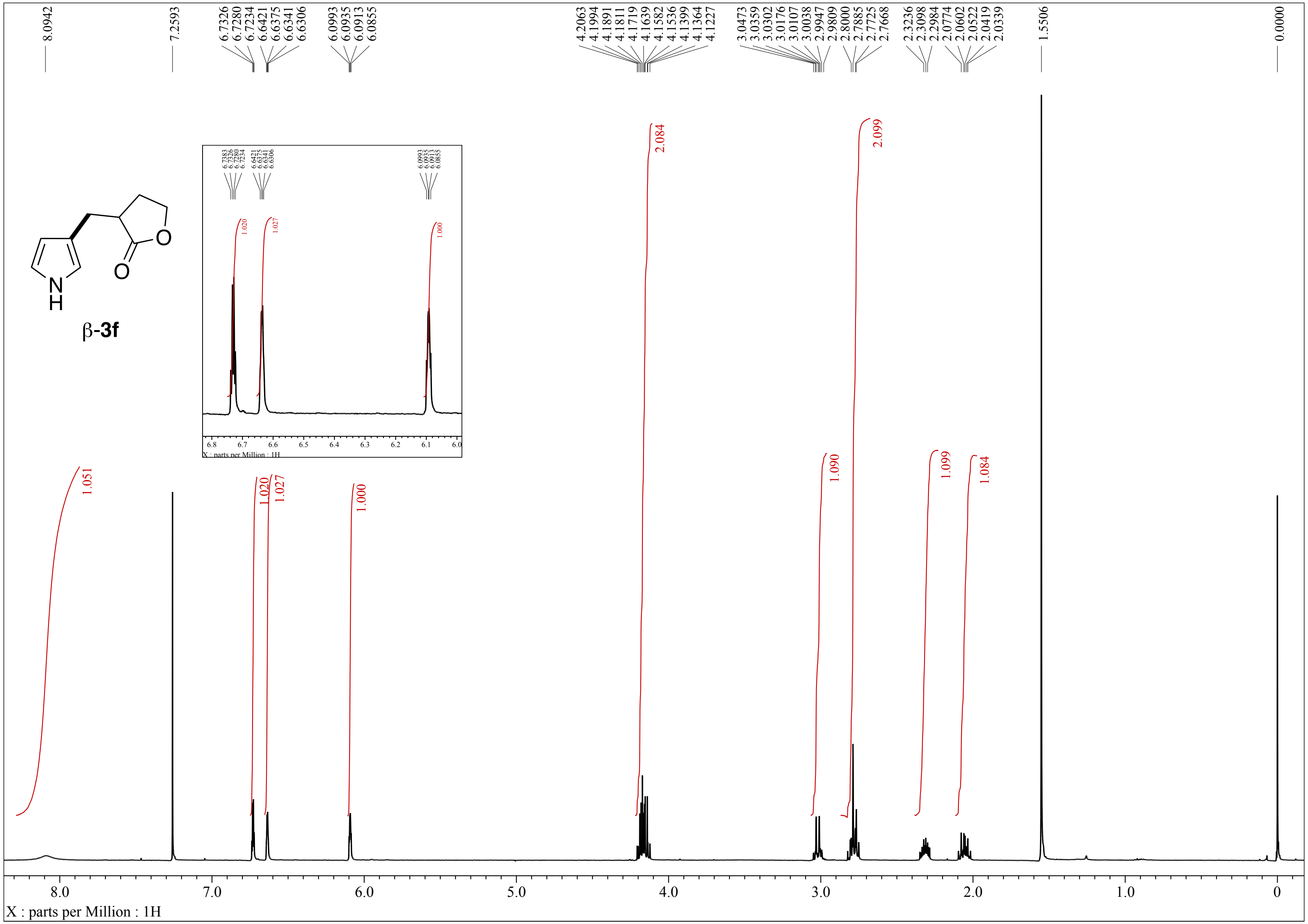

$^{13}C\{^{1}H\}$ NMR (125 MHz, $CDCl_3$)

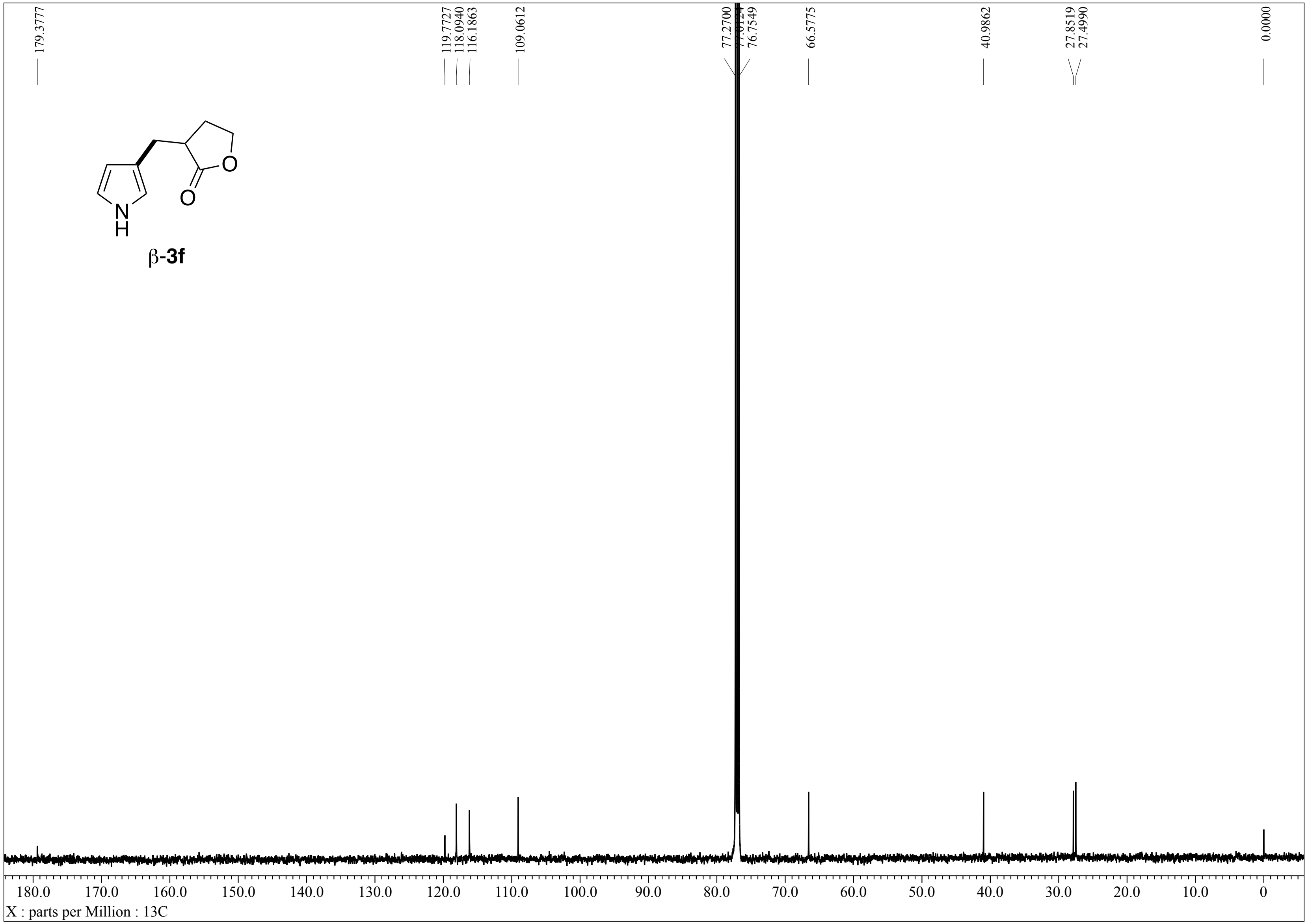

^{1}H NMR (500 MHz, $CDCl_3$)

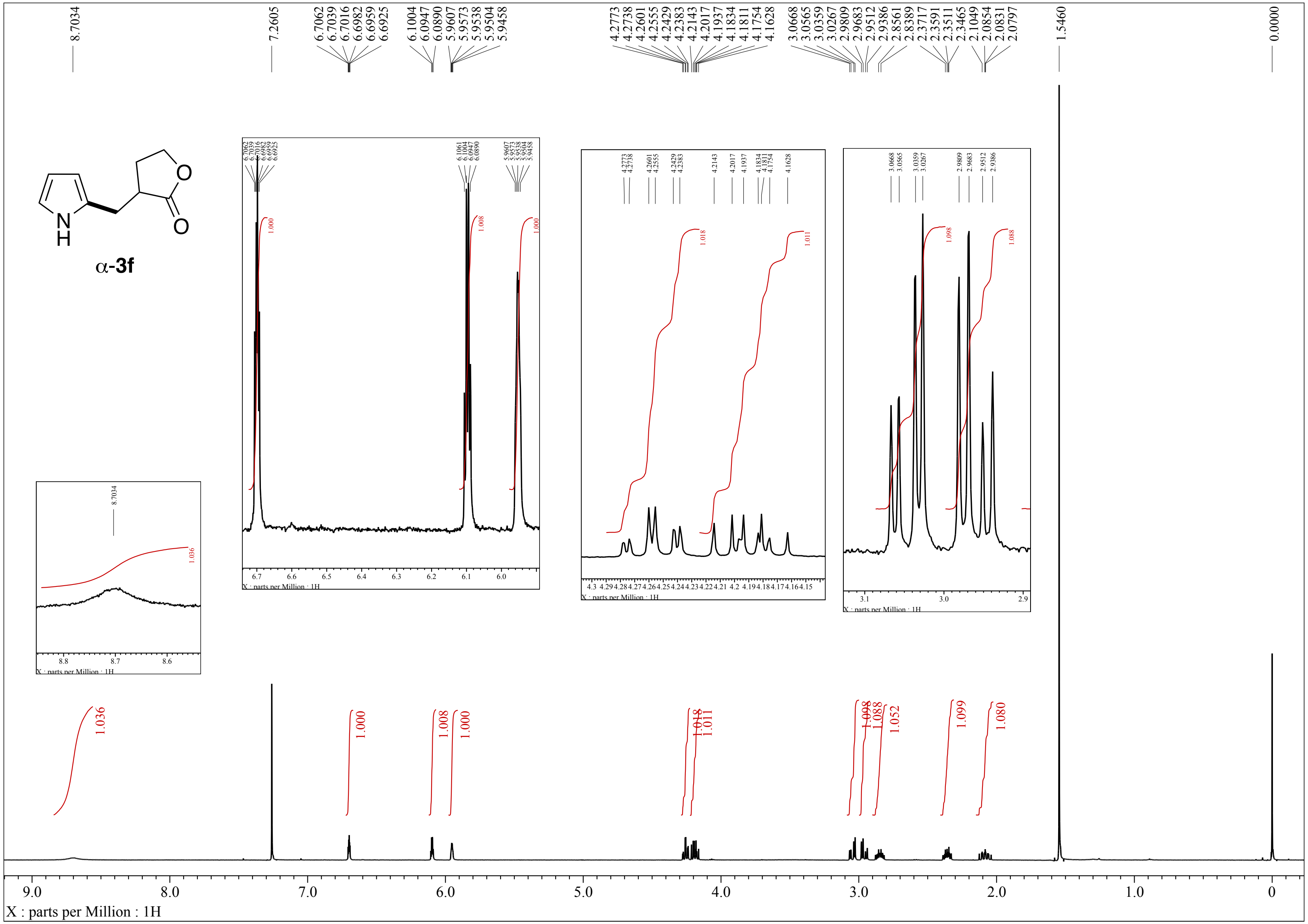

$^{13}C\{^{1}H\}$ NMR (125 MHz, $CDCl_3$)

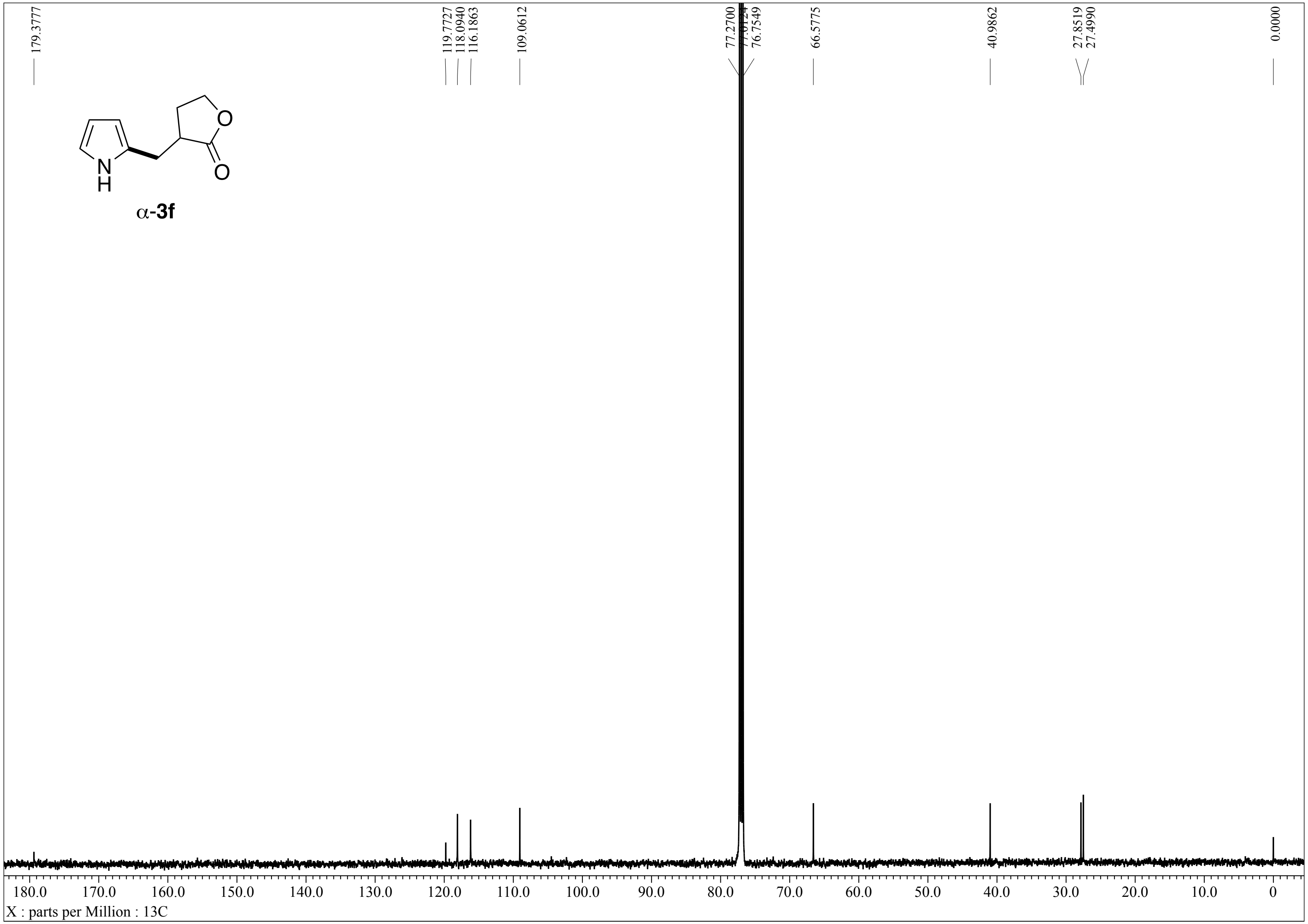

^{1}H NMR (500 MHz, $CDCl_3$)

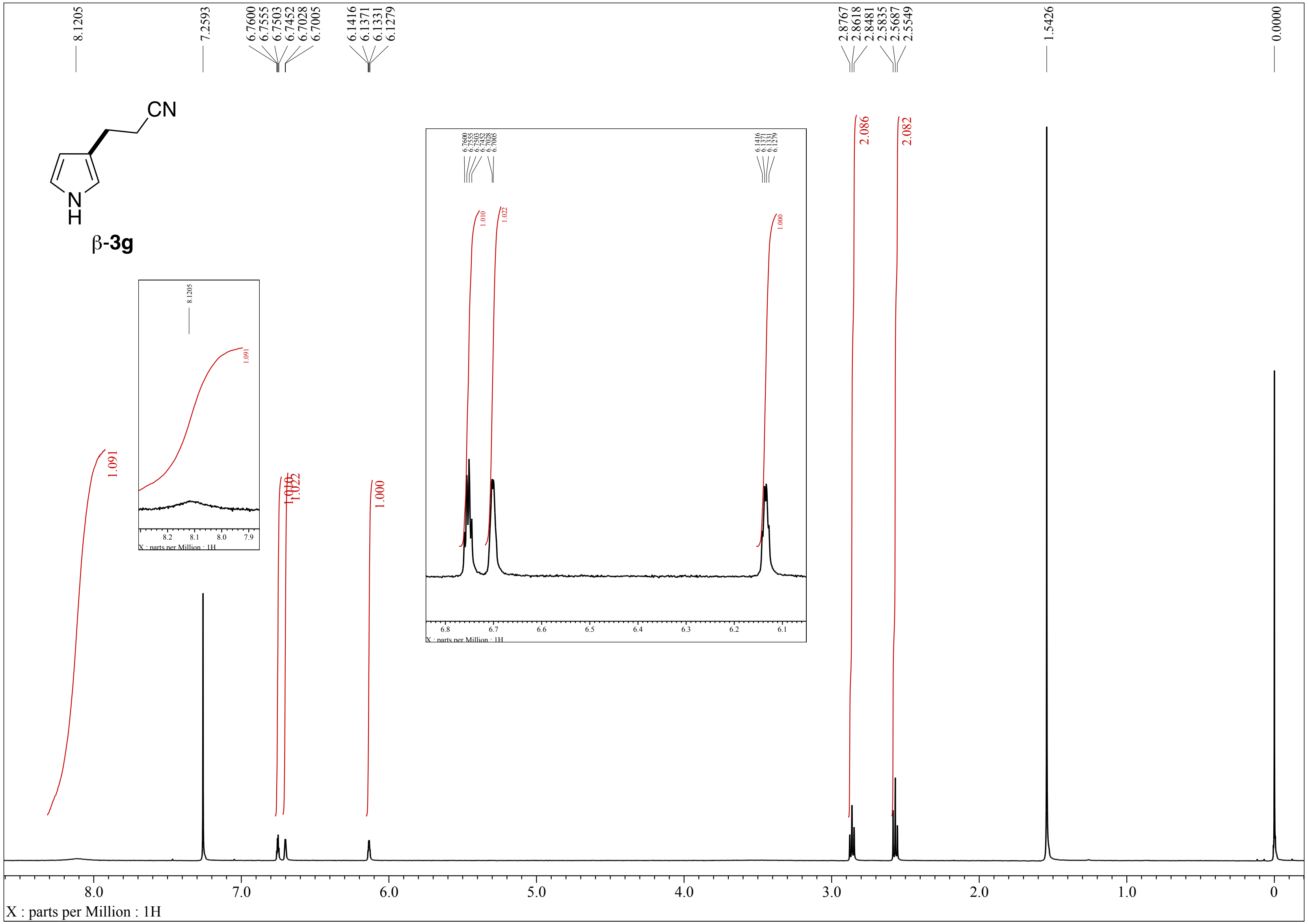

$^{13}C\{^{1}H\}$ NMR (125 MHz, $CDCl_3$)

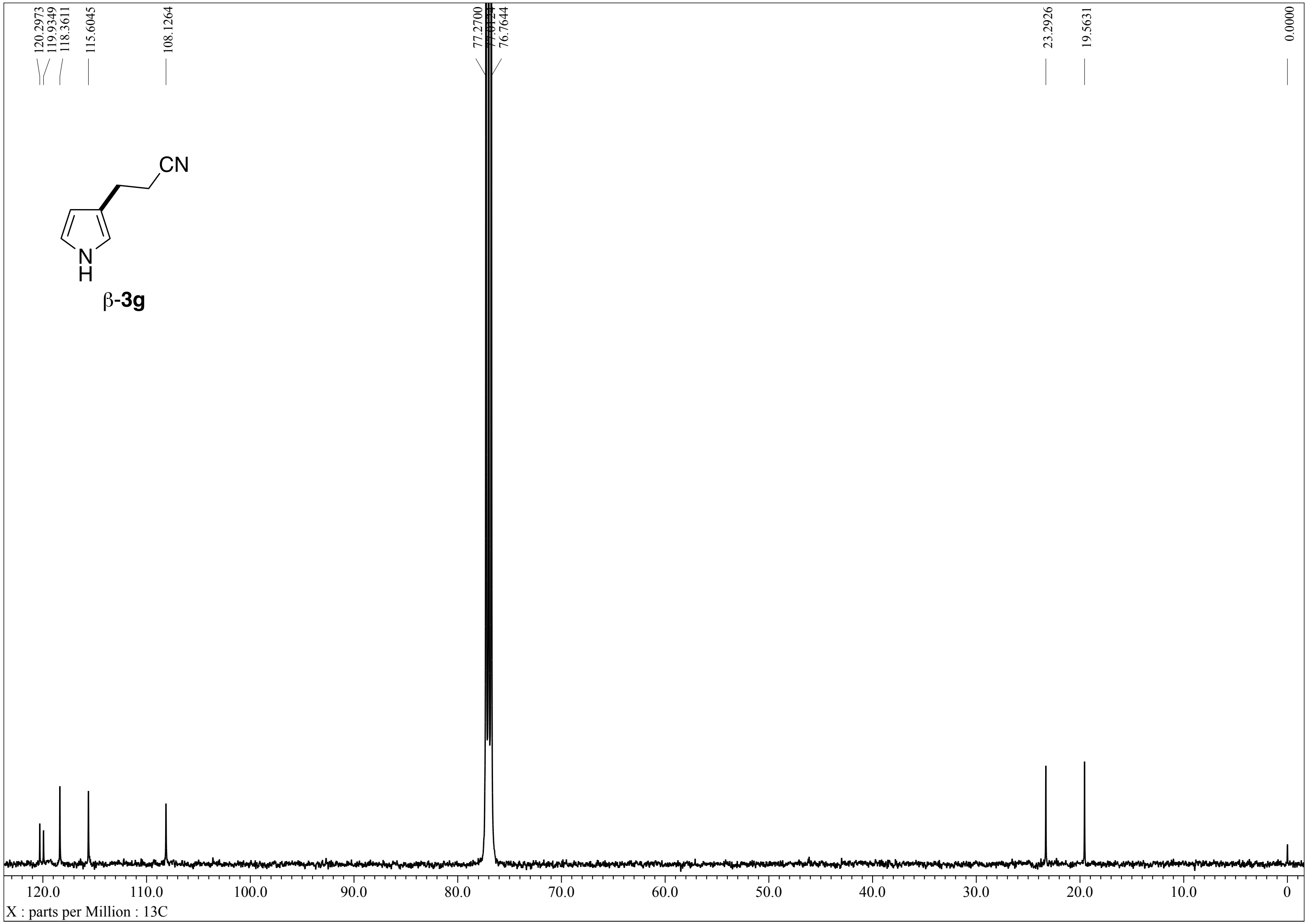

^{1}H NMR (400 MHz, $CDCl_3$)

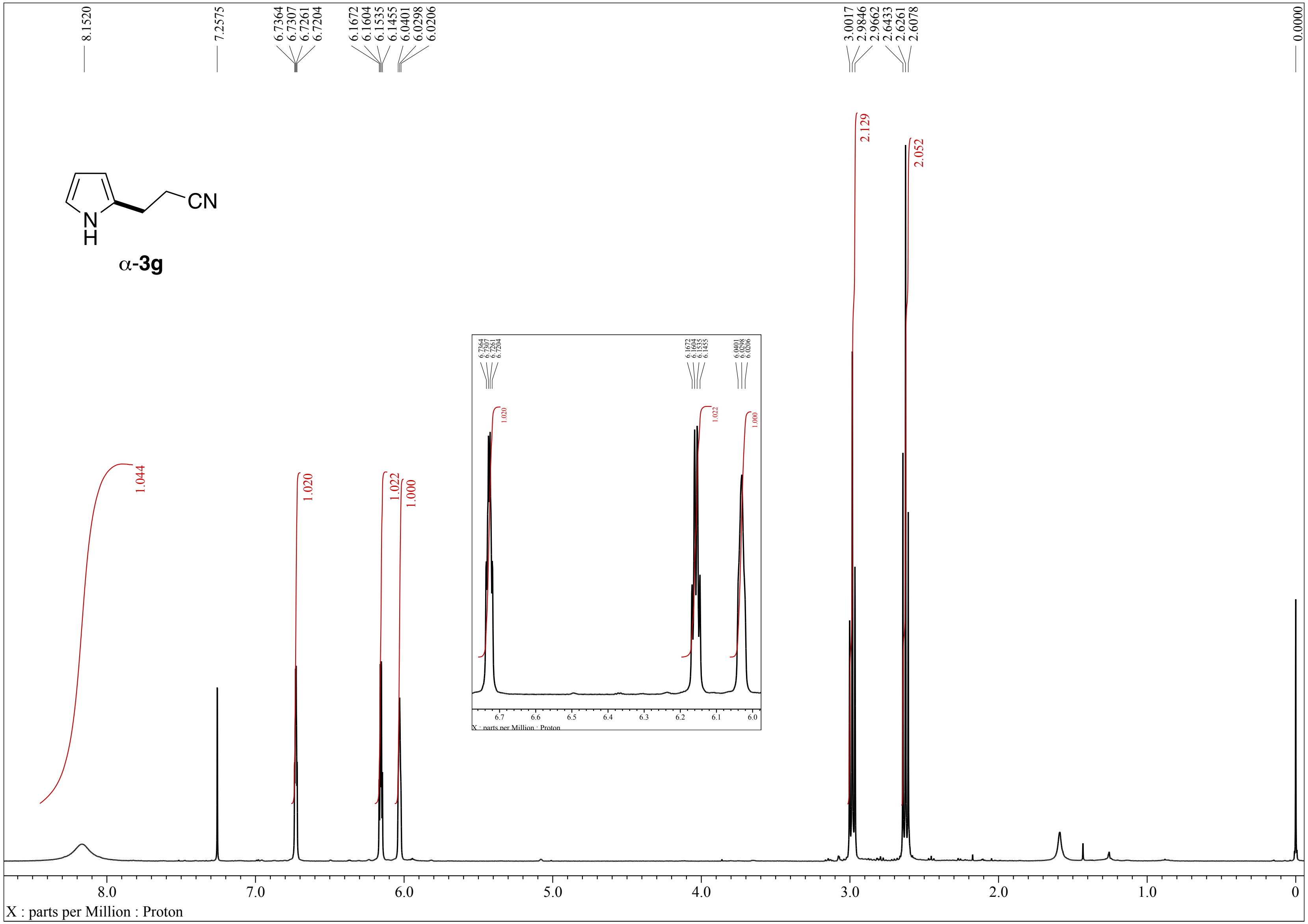

$^{13}C\{^{1}H\}$ NMR (125 MHz, $CDCl_3$)

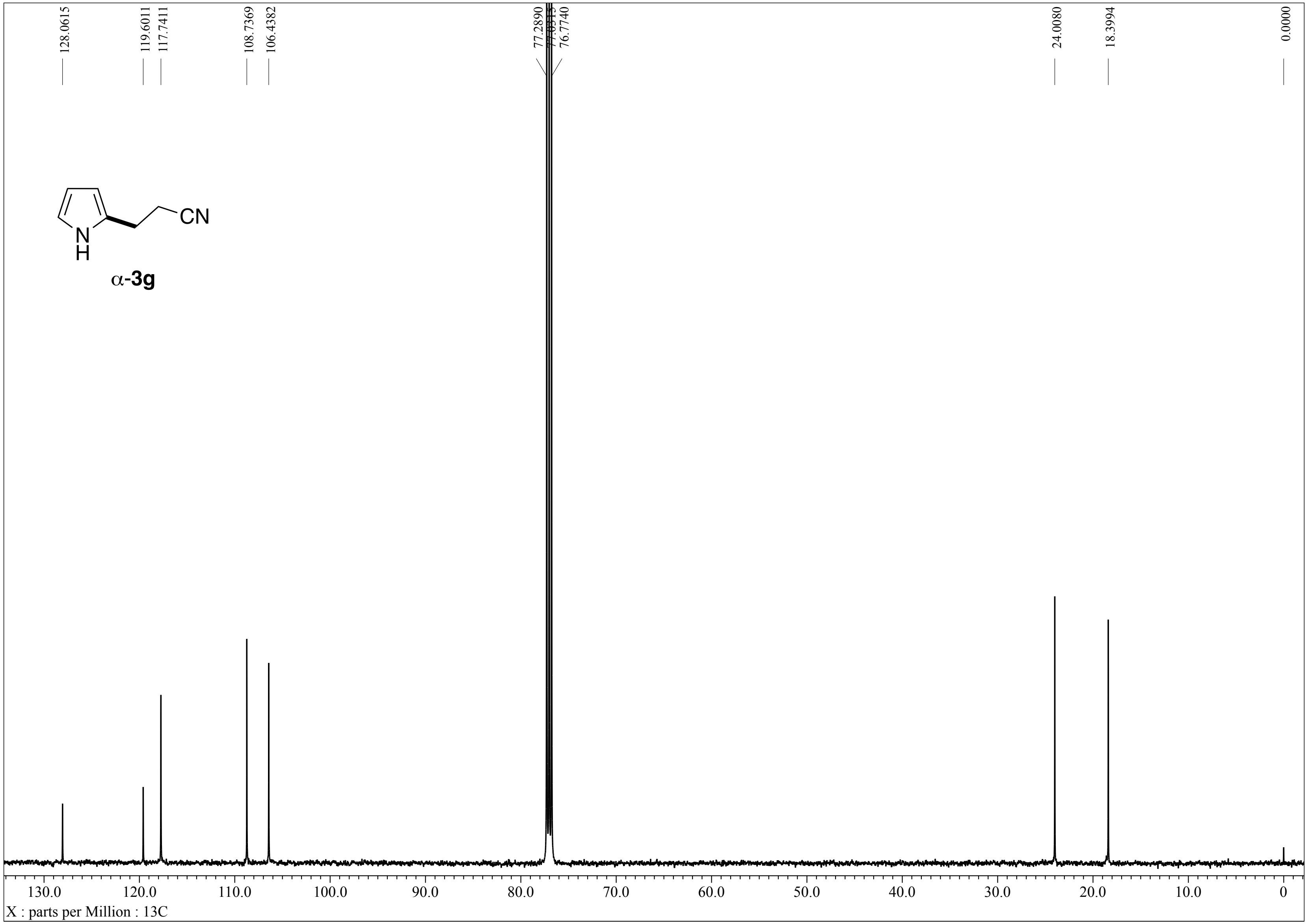

^1H NMR (400 MHz, $CDCl_3$)

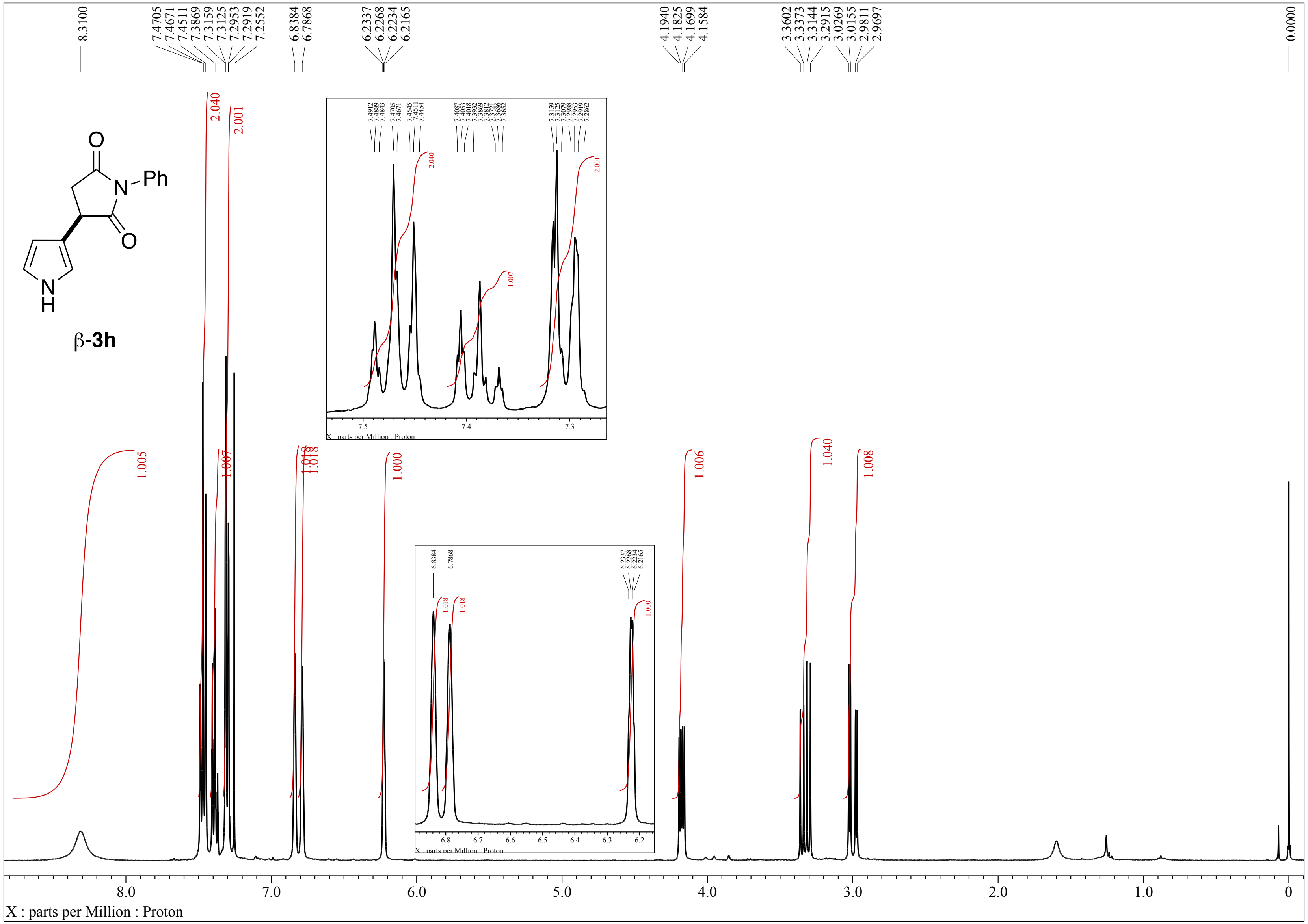

$^{13}C\{^{1}H\}$ NMR (100 MHz, $CDCl_3$)

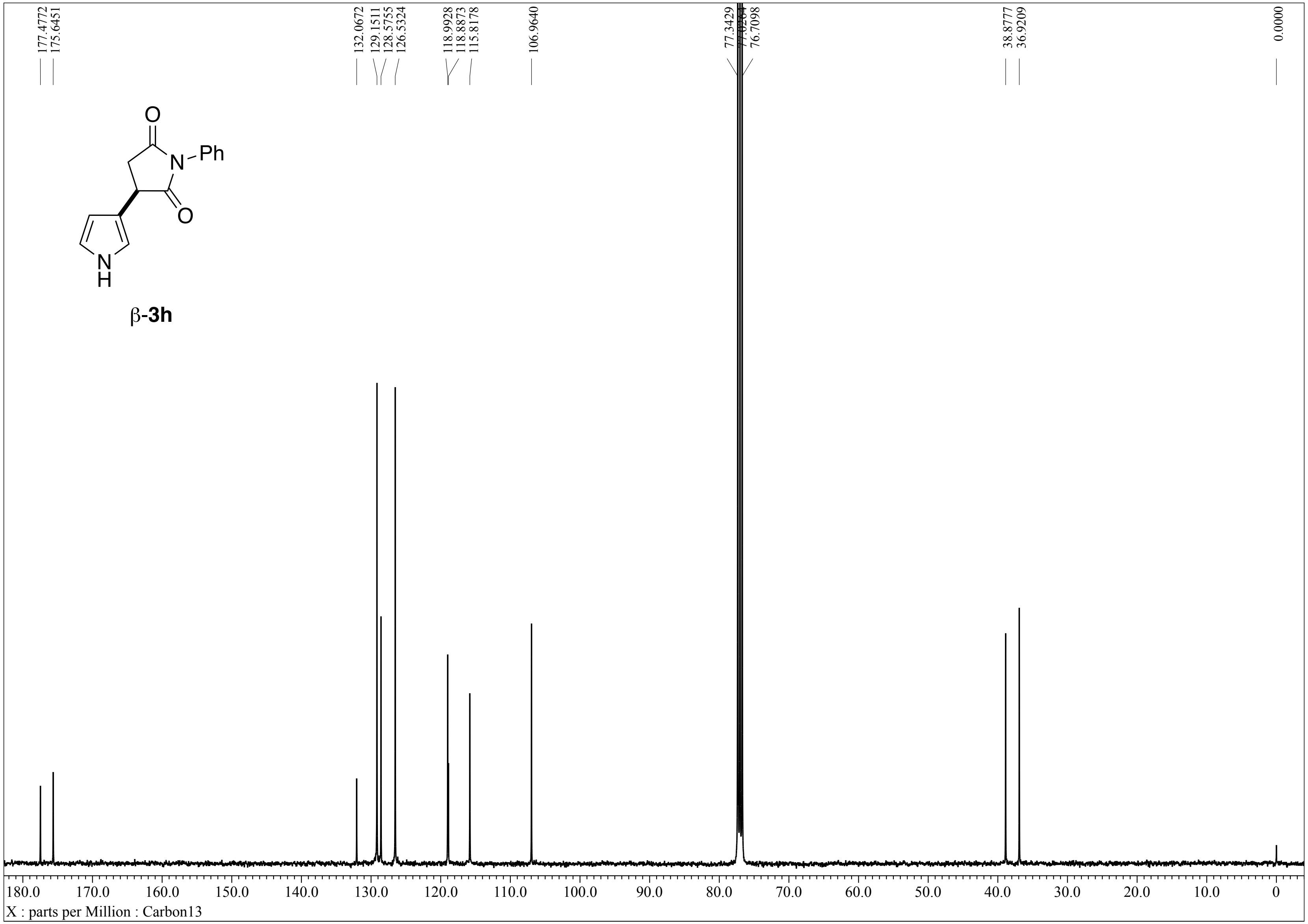

^{1}H NMR (400 MHz, $CDCl_3$)

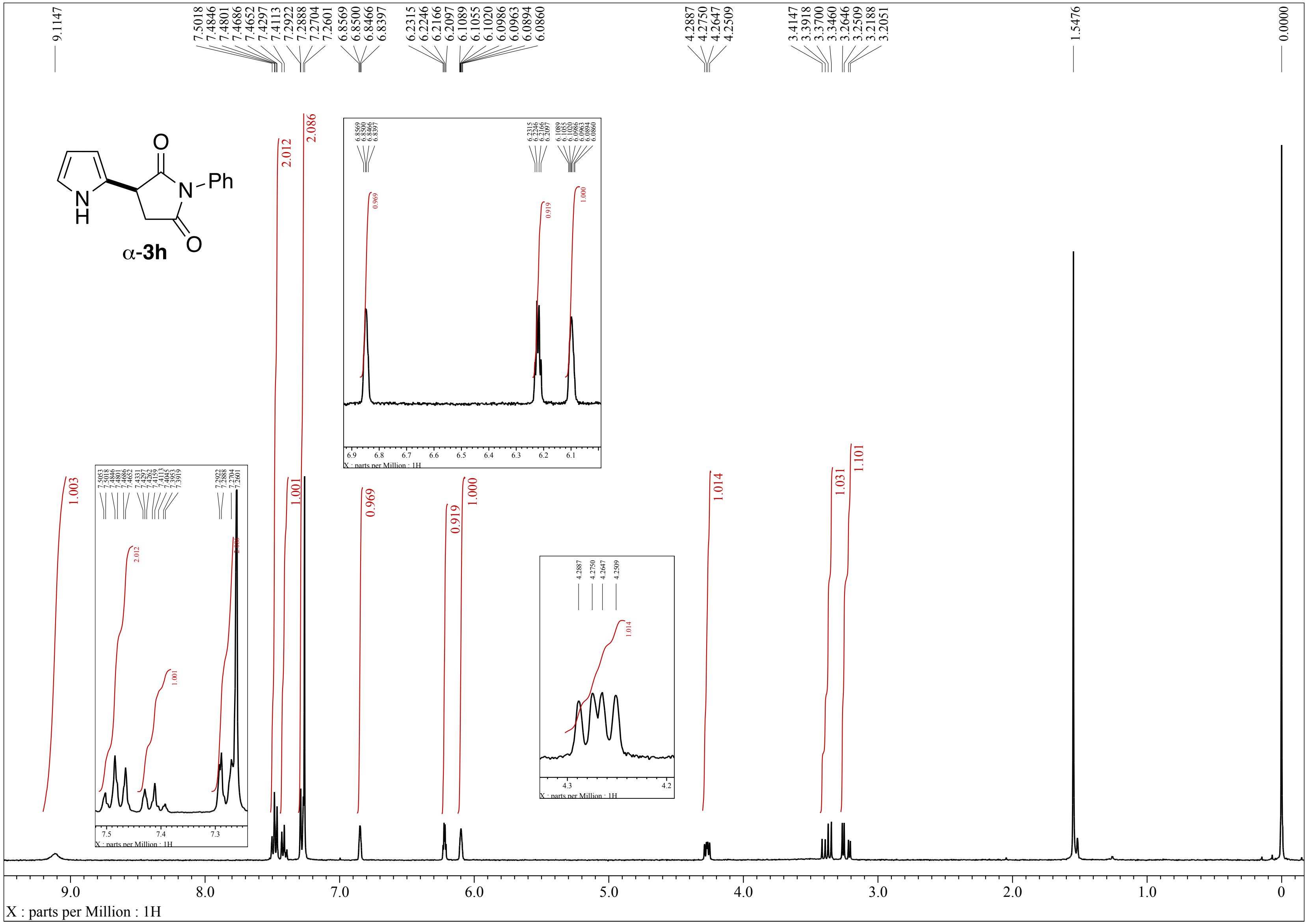

^{1}H NMR (500 MHz, CD_2Cl_2)

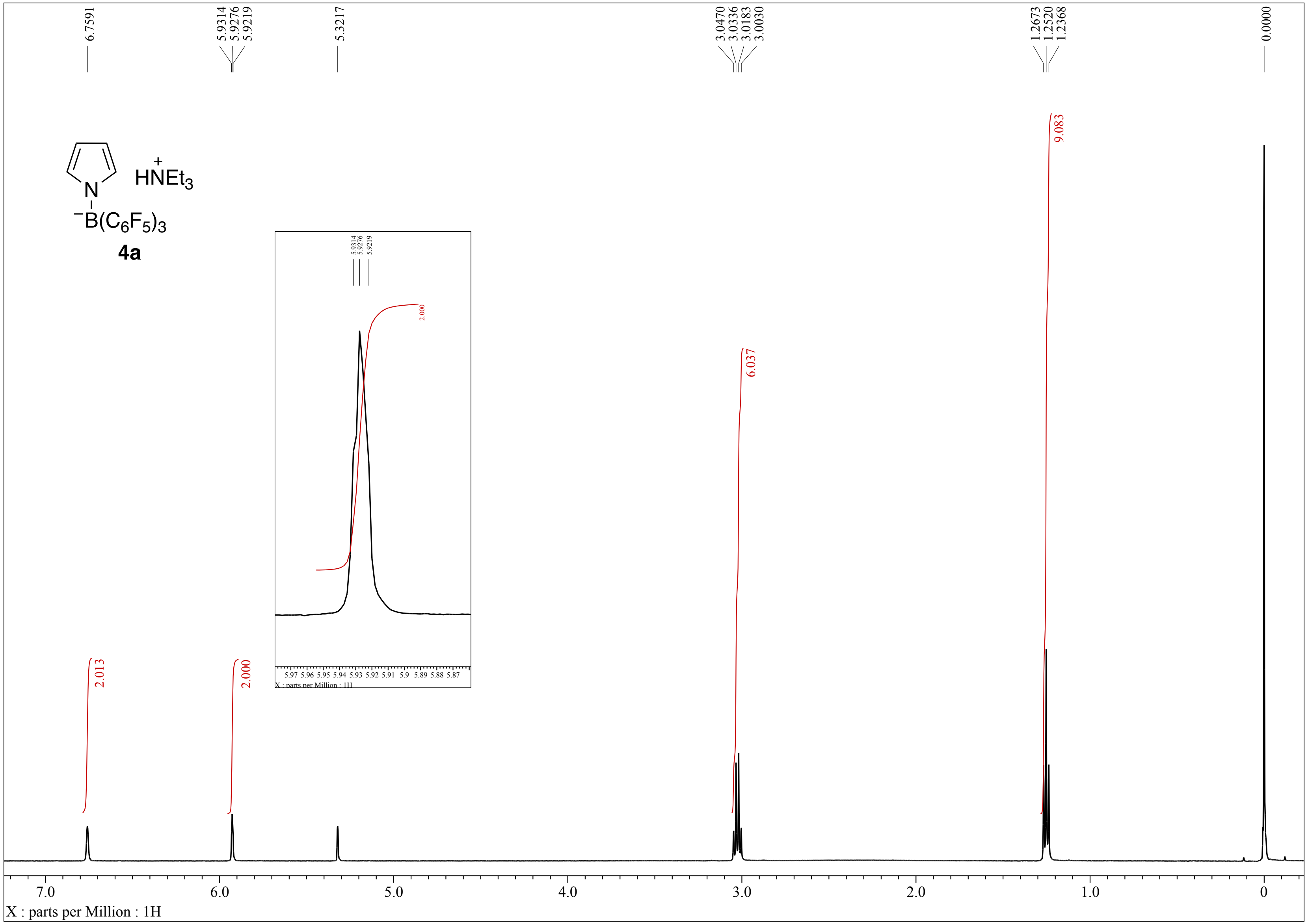

$^{13}C\{^1H\}$ NMR (125 MHz, CD_2Cl_2)

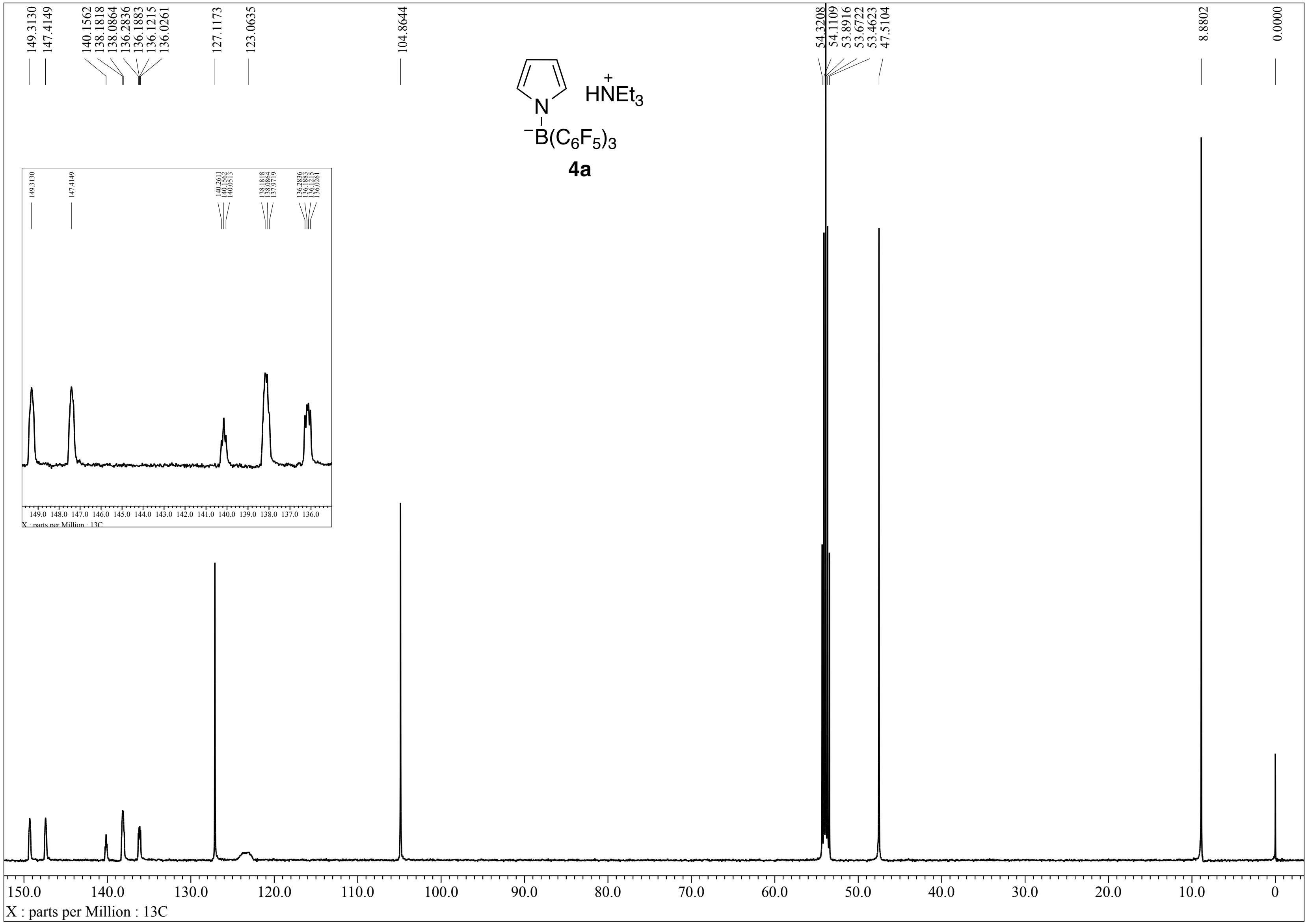

^{19}F NMR (376 MHz, CD_2Cl_2)

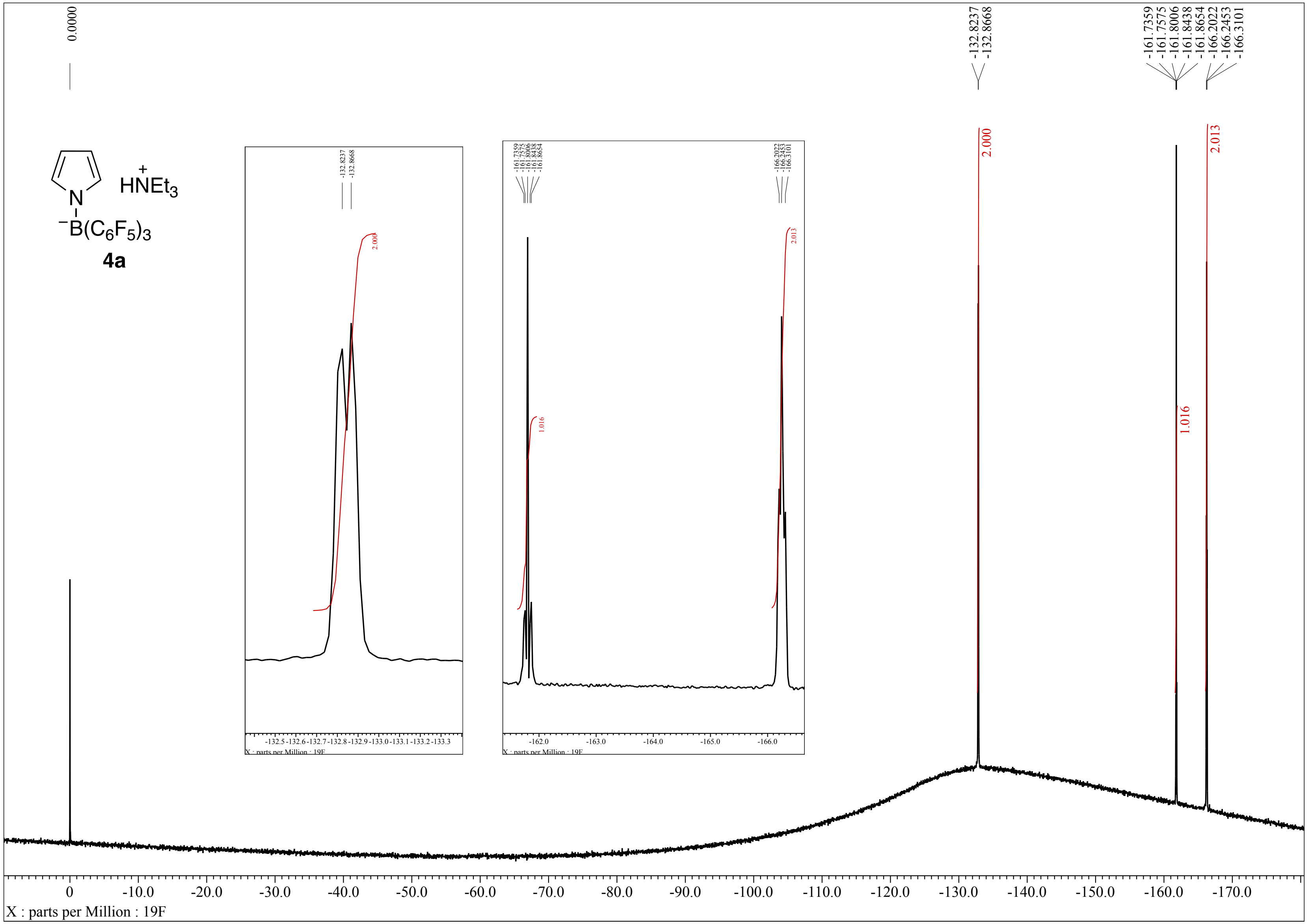

^{11}B NMR (128 MHz, CD_2Cl_2)

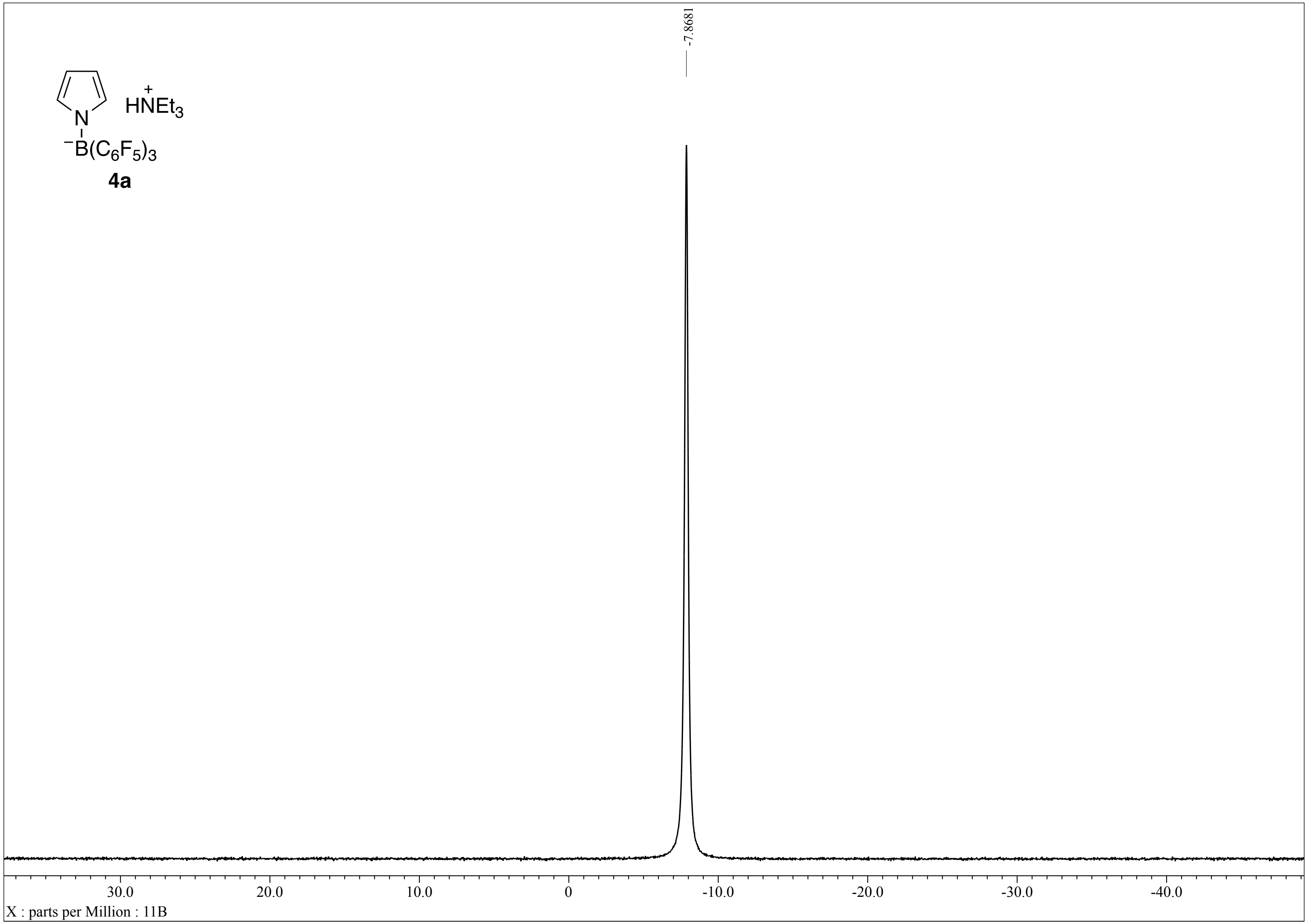

^{1}H NMR (500 MHz, CD_2Cl_2)

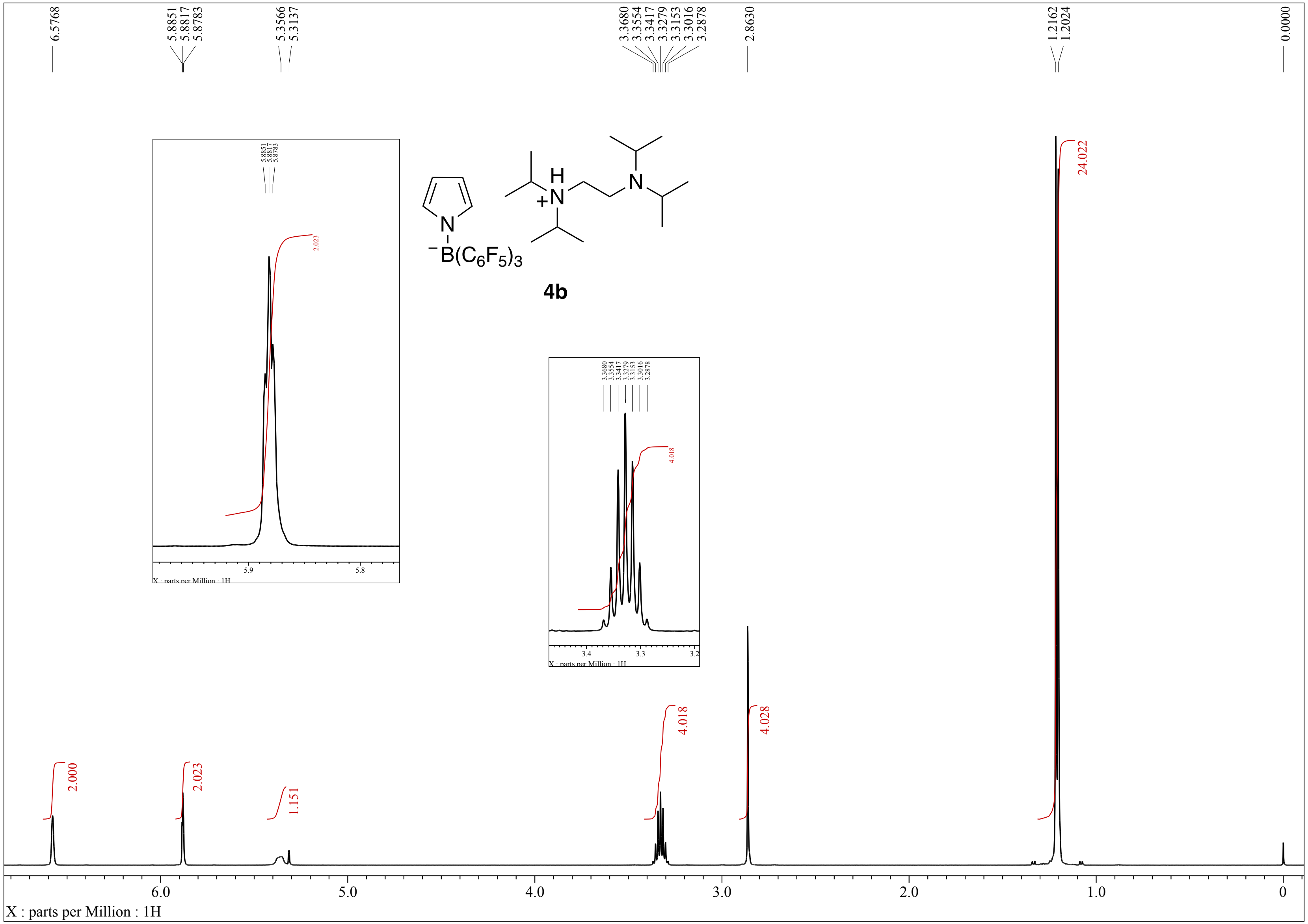

$^{13}C\{^1H\}$ NMR (125 MHz, CD_2Cl_2)

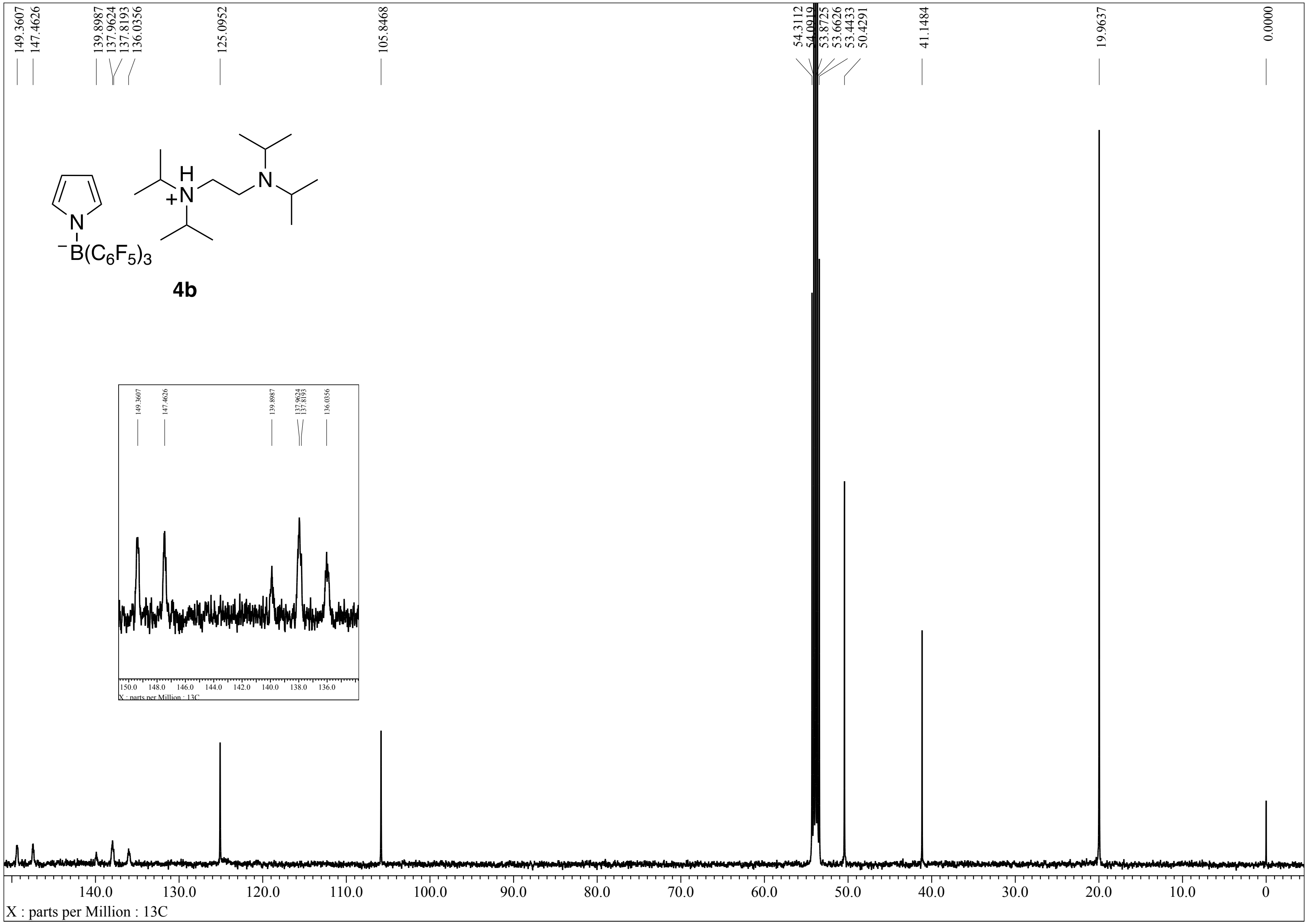

^{19}F NMR (376 MHz, CD_2Cl_2)

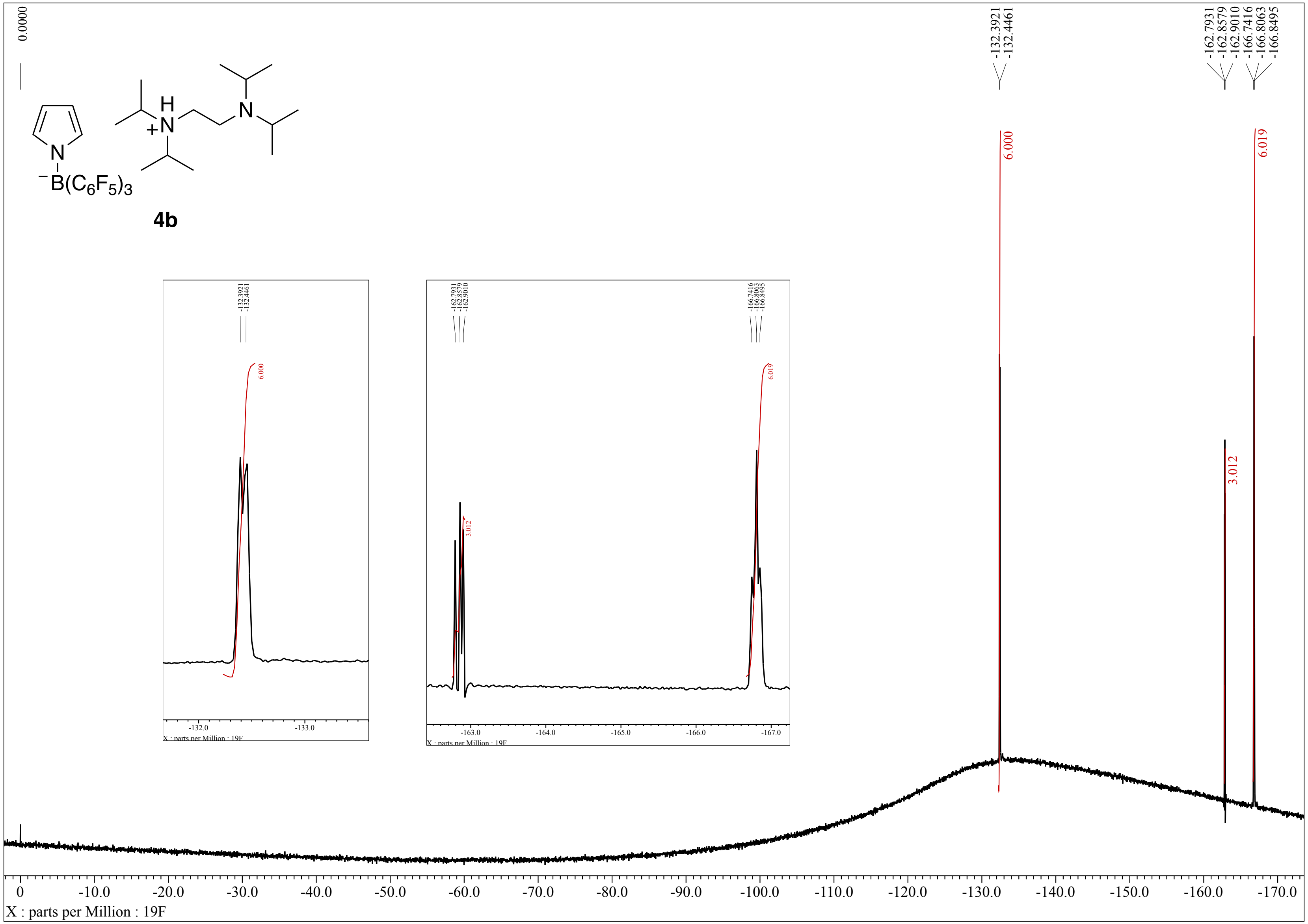

^{11}B NMR (128 MHz, CD_2Cl_2)

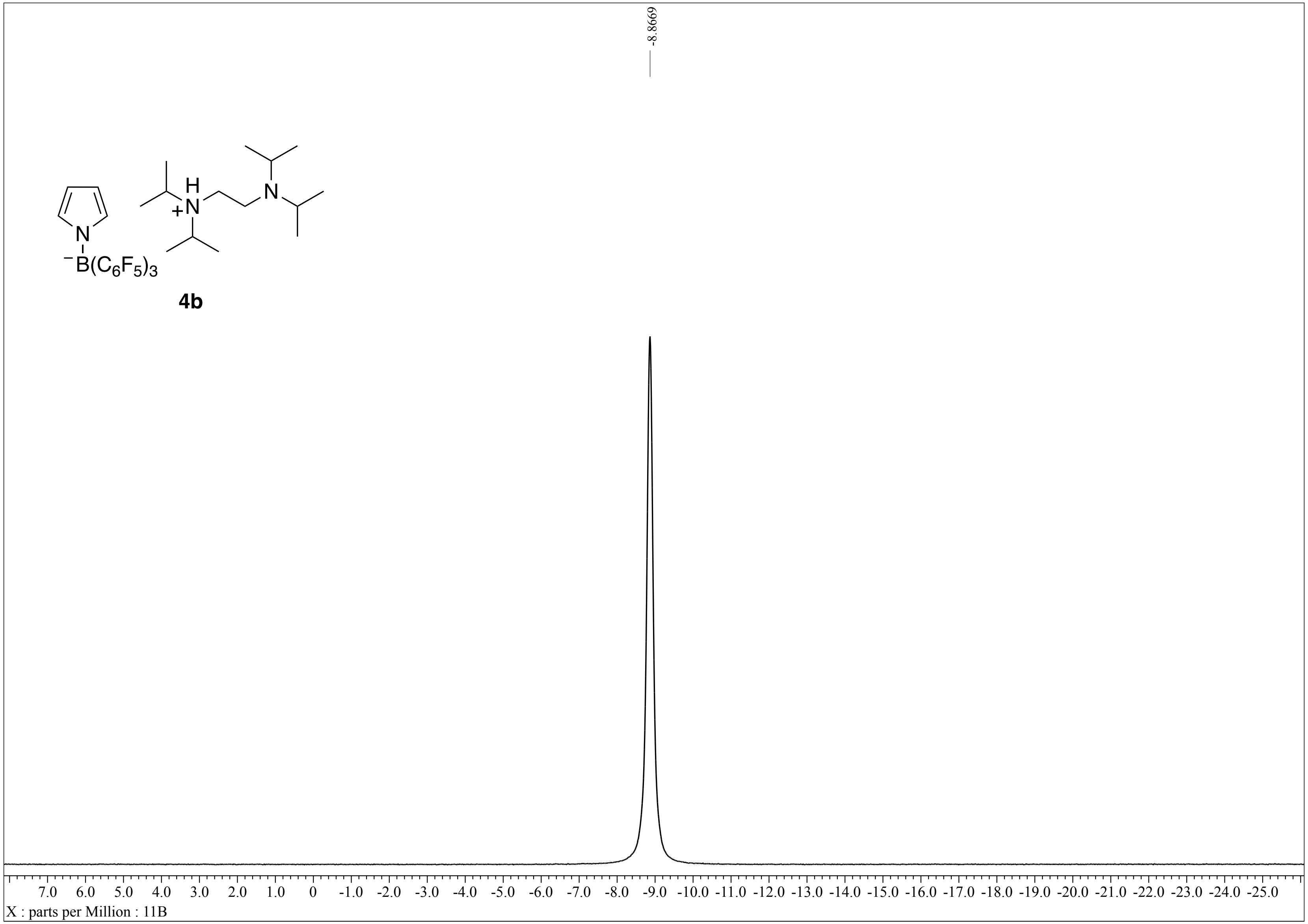

^{1}H NMR (500 MHz, CD_2Cl_2)

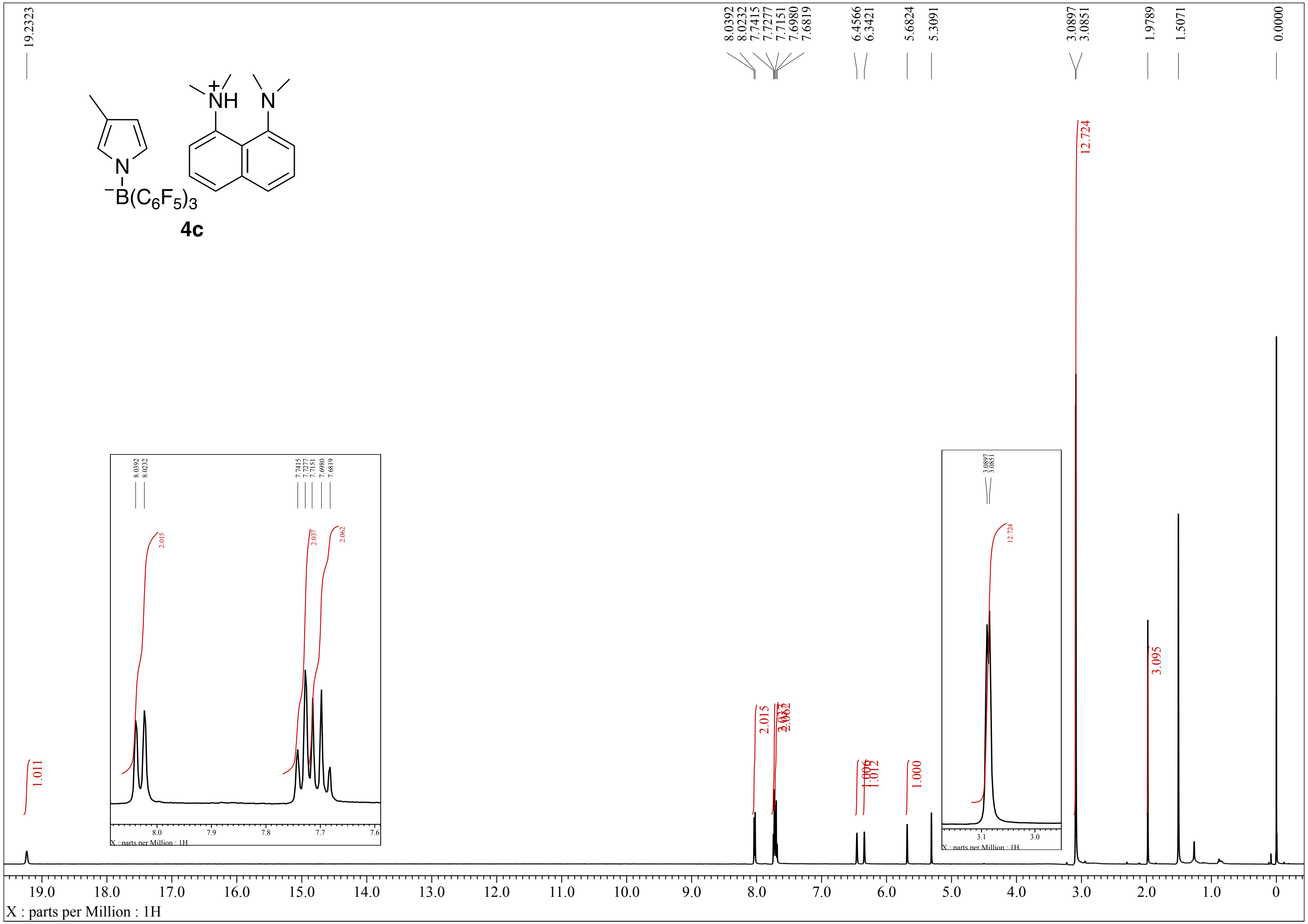

$^{13}C\{^1H\}$ NMR (125 MHz, CD_2Cl_2)

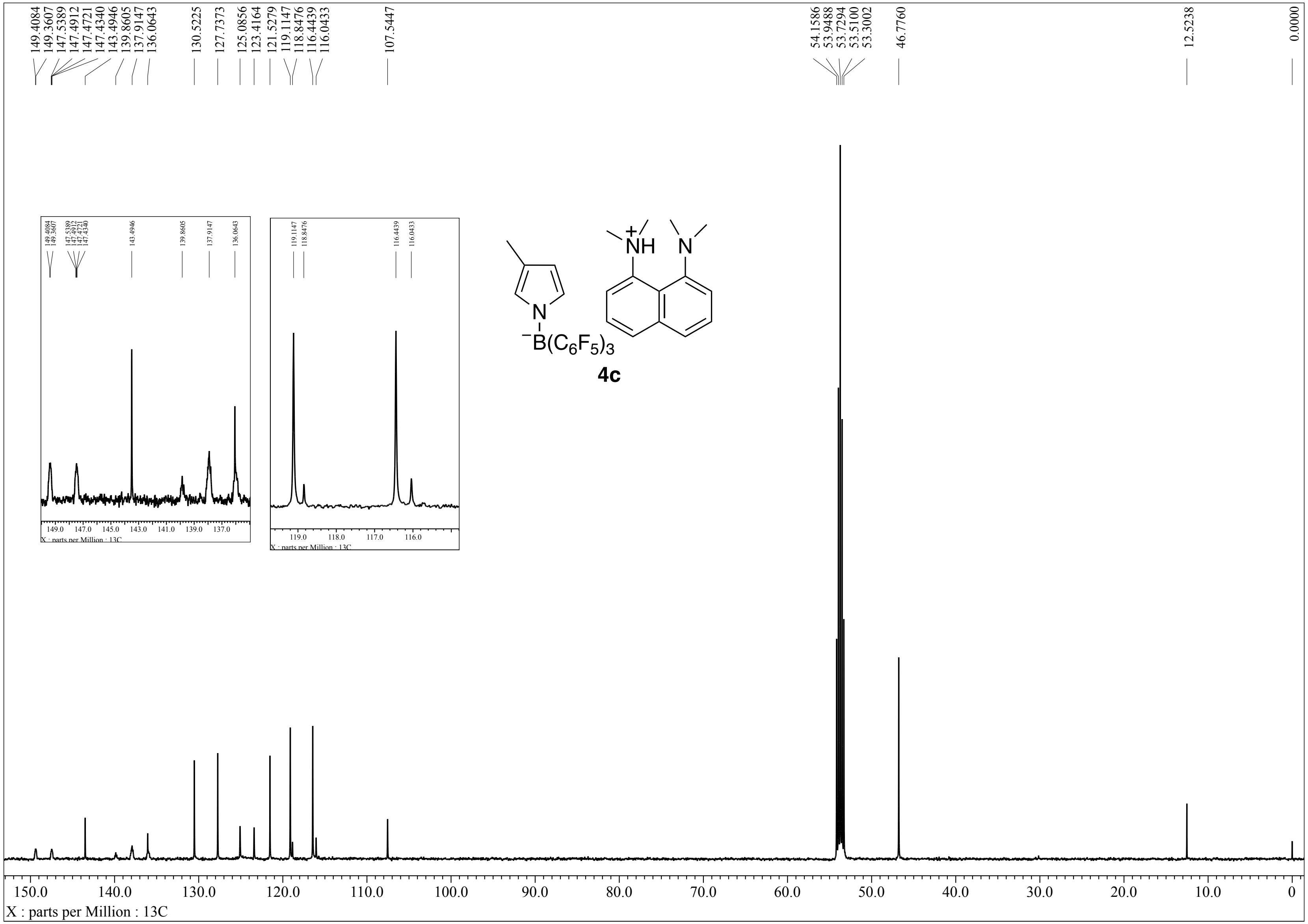

^{19}F NMR (471 MHz, CD_2Cl_2)

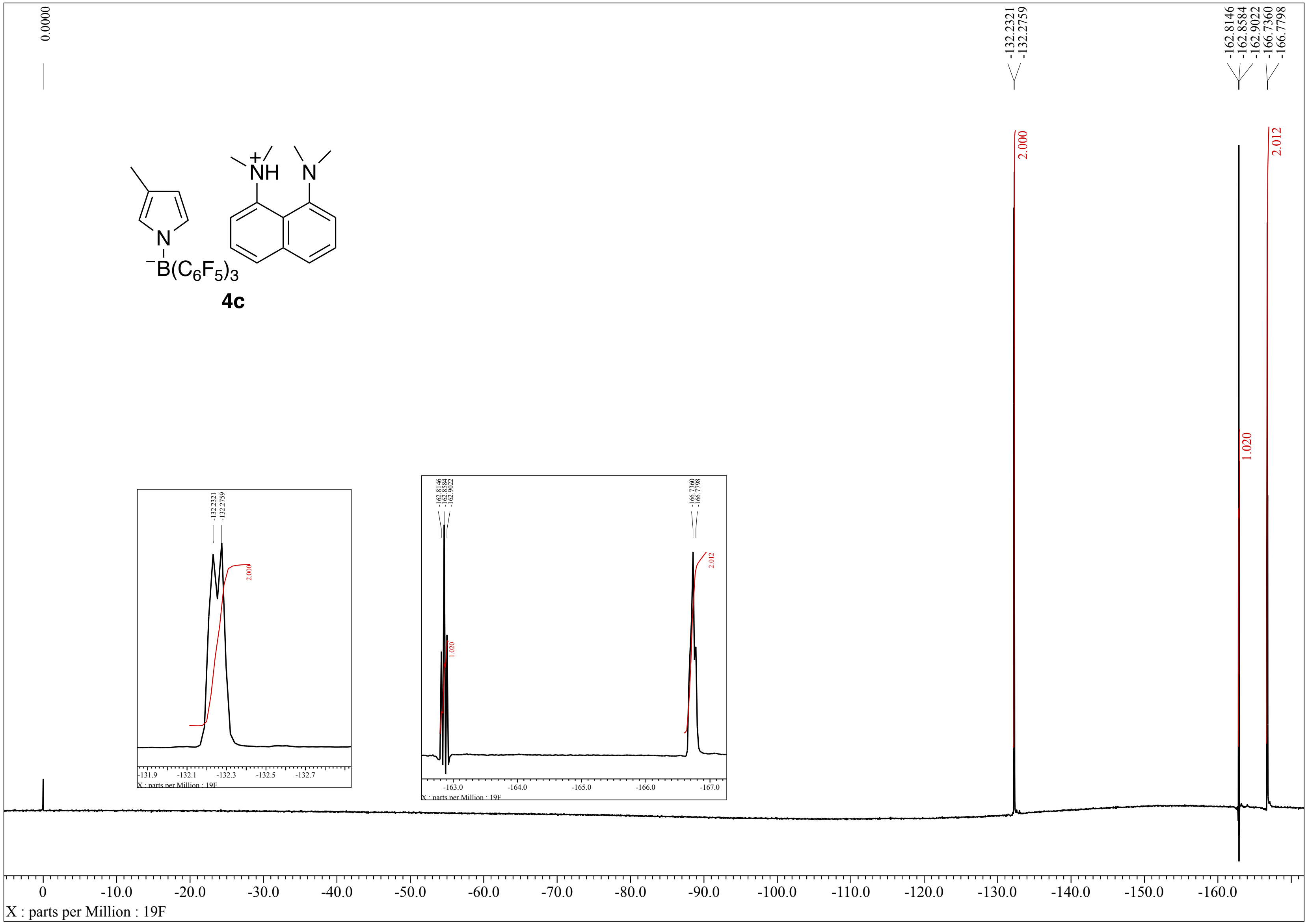

^{11}B NMR (128 MHz, CD_2Cl_2)

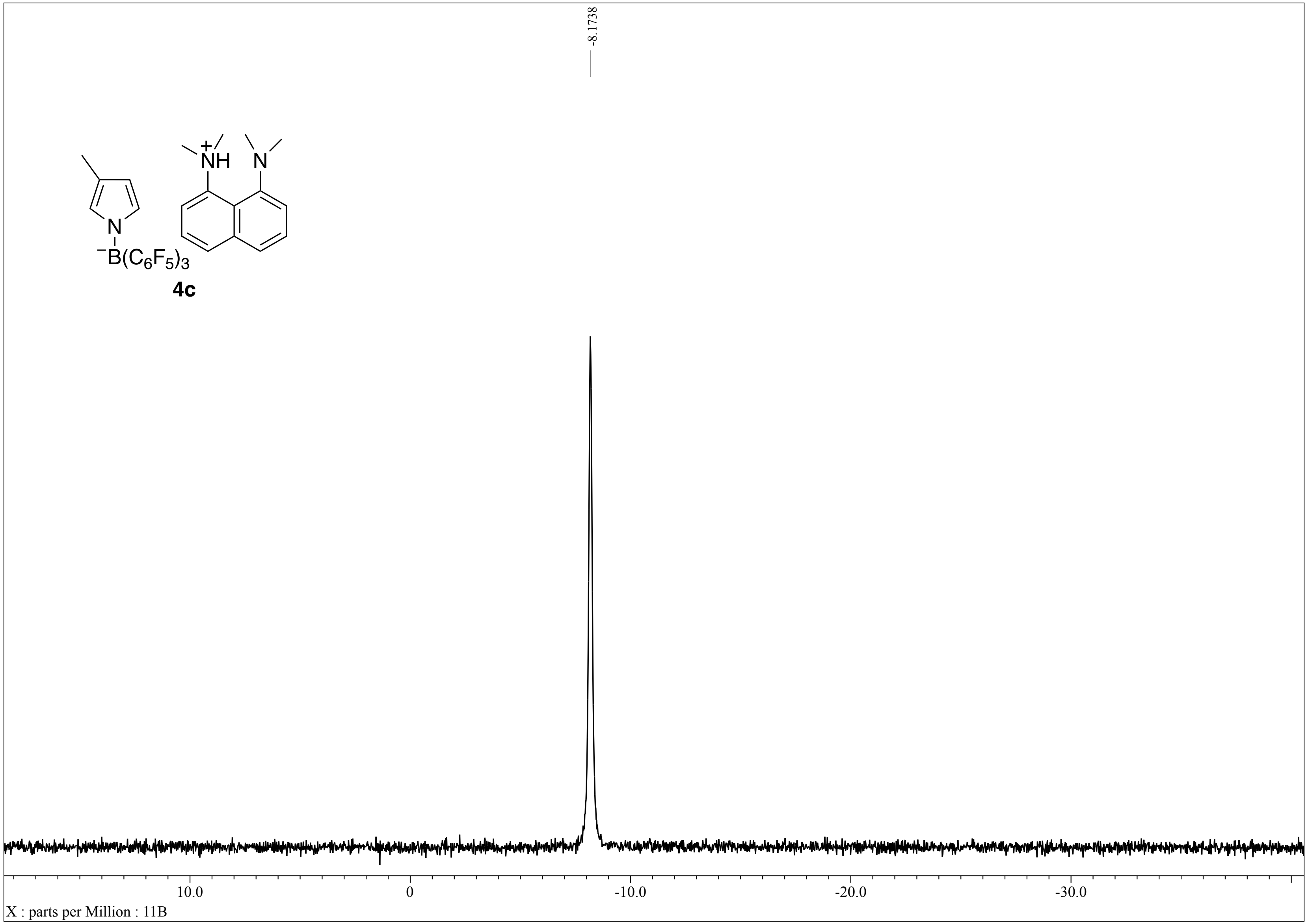

^{1}H NMR (400 MHz, CD_2Cl_2)

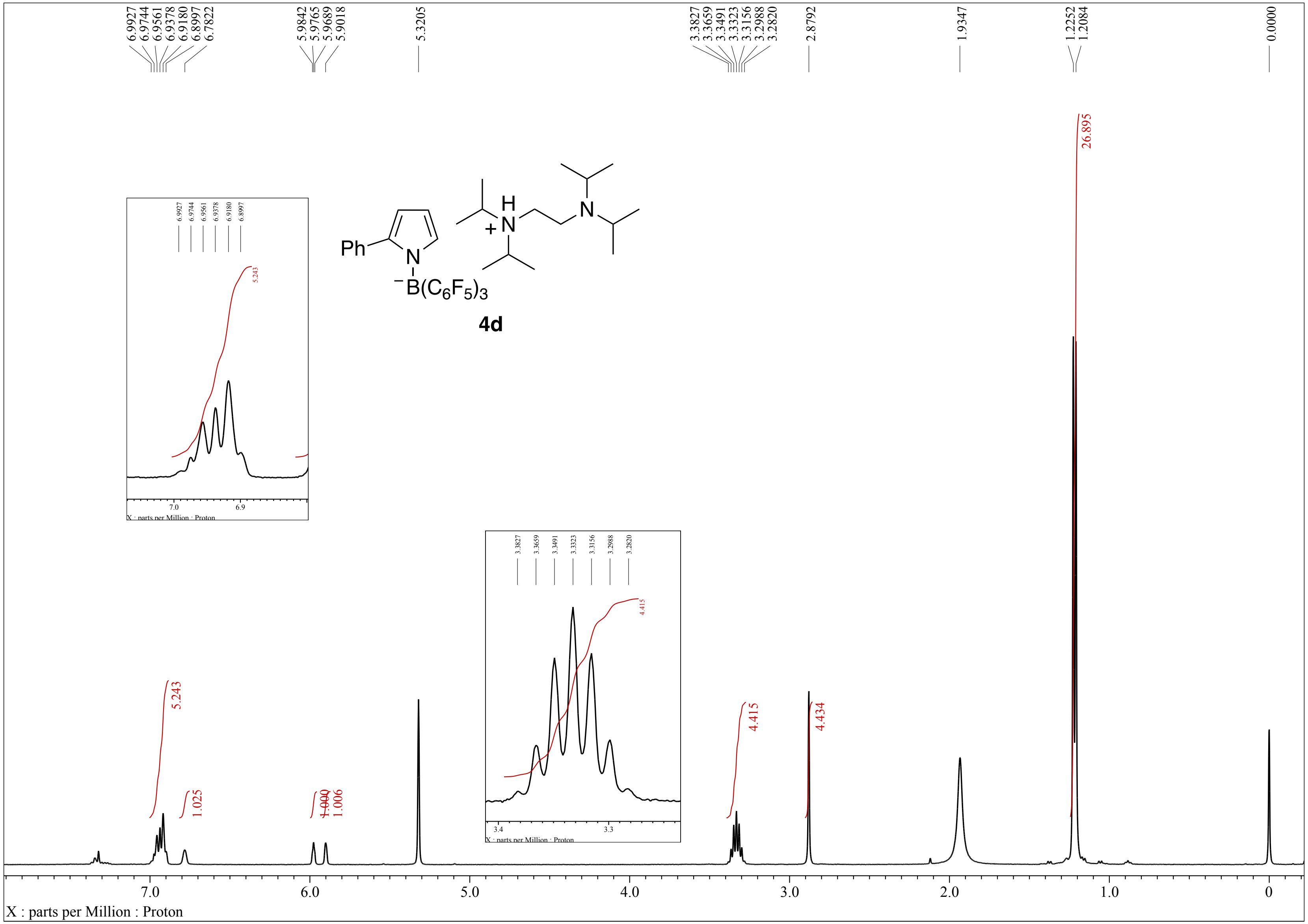

$^{13}C\{^1H\}$ NMR (100 MHz, CD_2Cl_2)

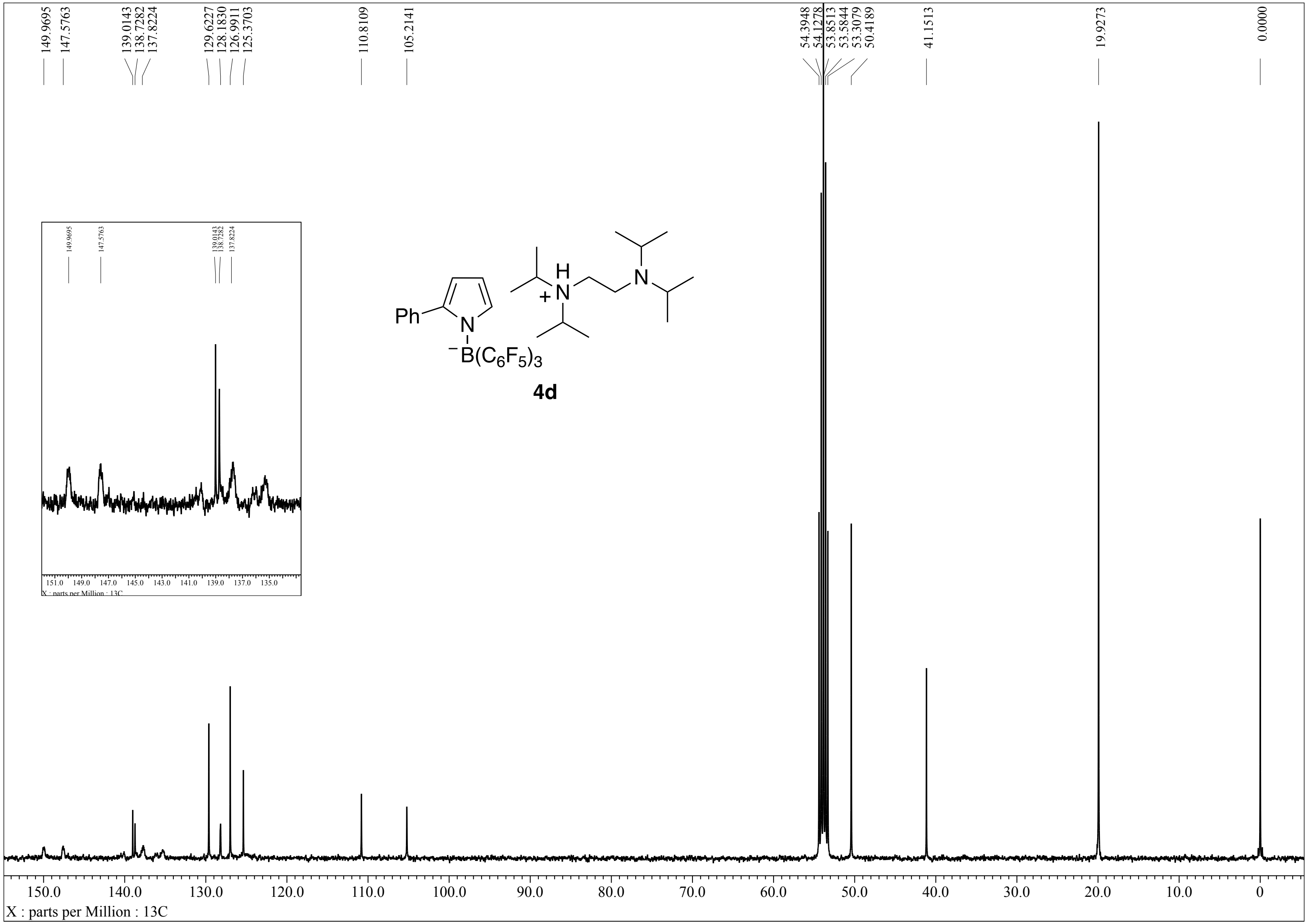

4d

^{19}F NMR (376 MHz, CD_2Cl_2)

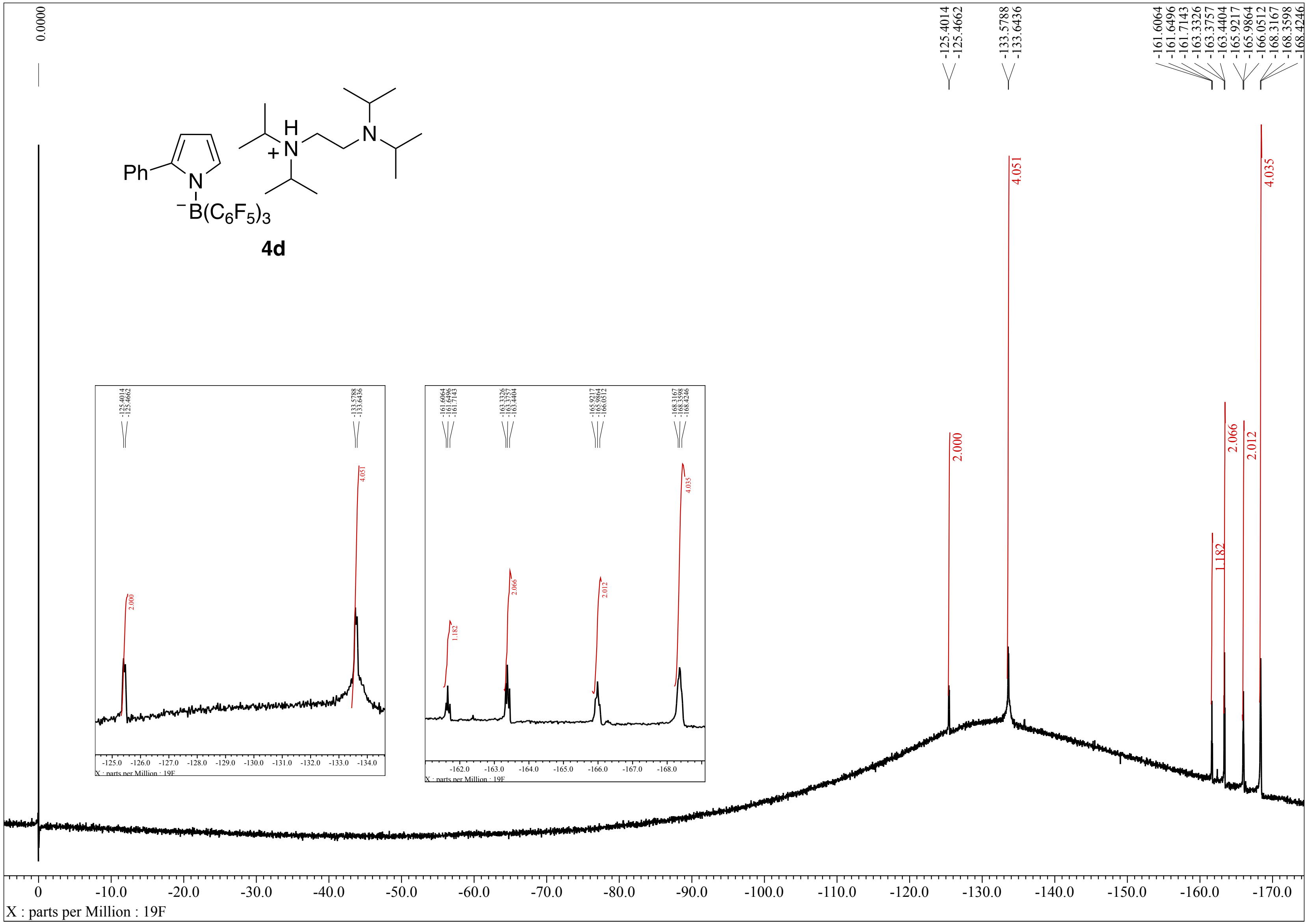

^{11}B NMR (128 MHz, CD_2Cl_2)

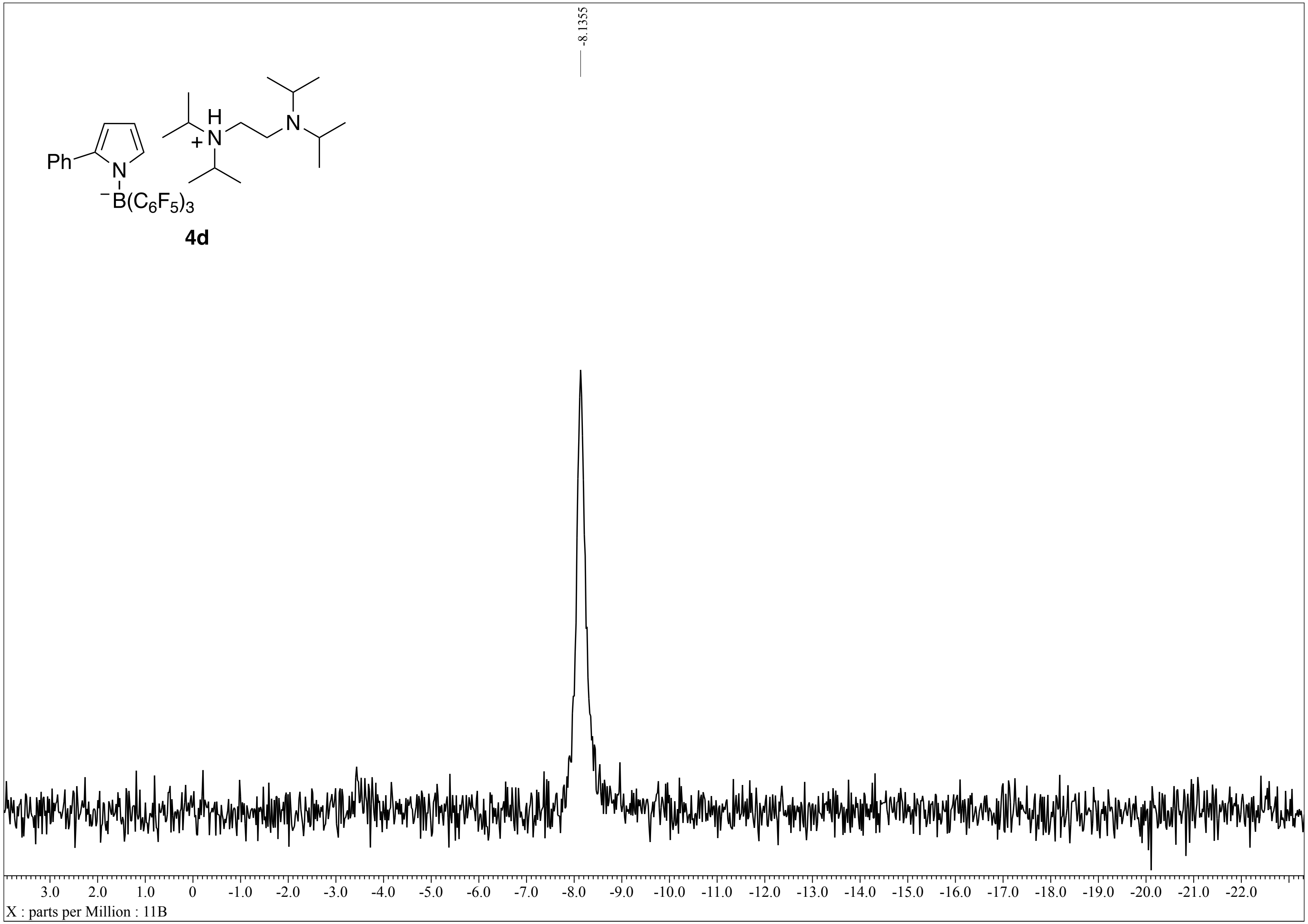

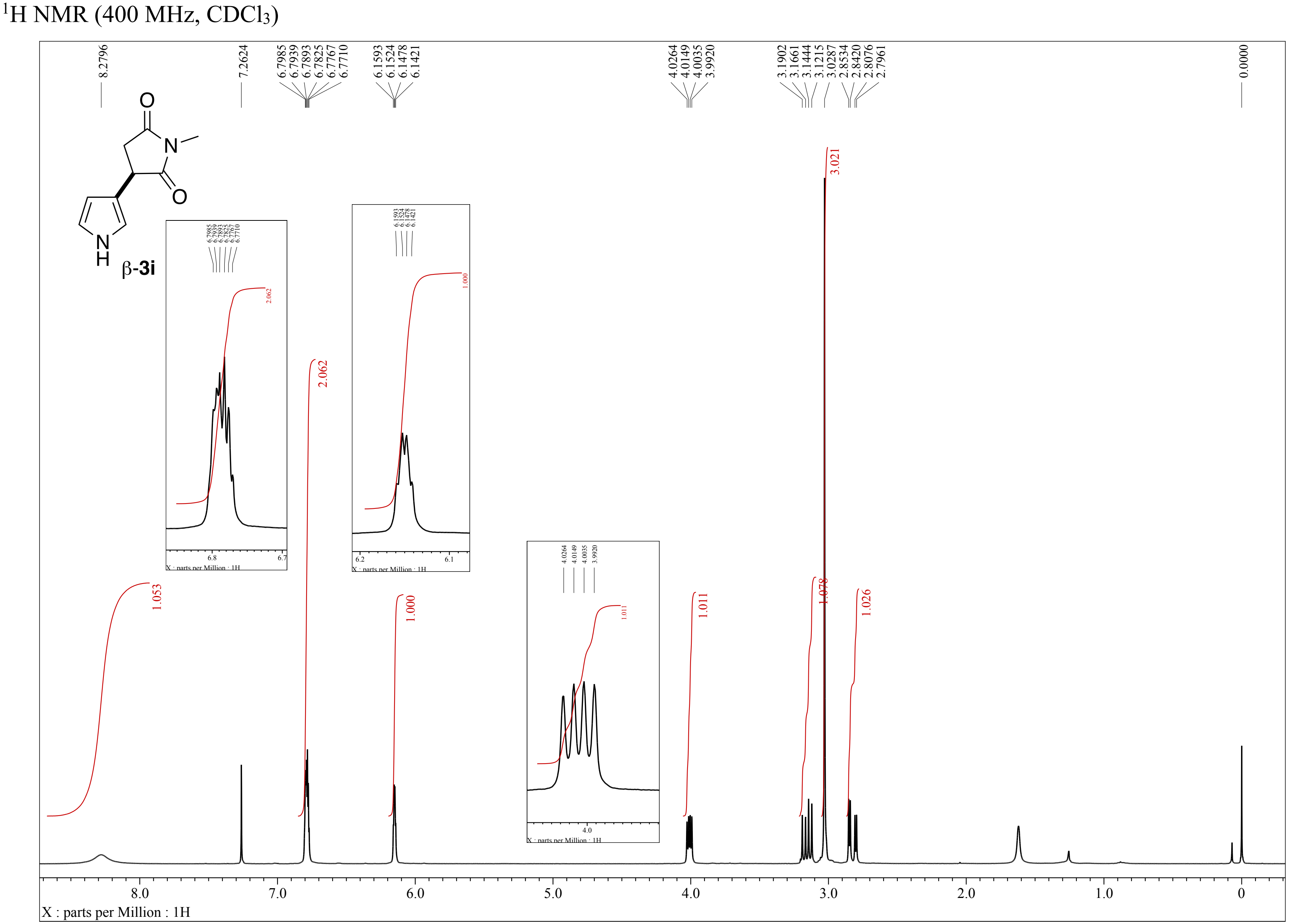

1H NMR (400 MHz, CDCl3)
8.2796
7.2624
6.7985
6.7939
6.7893
6.7825
6.7767
6.7710
6.1593
6.1524
6.1478
6.1421
4.0264
4.0149
4.0035
3.9920
3.1902
3.1661
3.1444
3.1215
3.0287
2.8534
2.8420
2.8076
2.7961
0.0000
β-3i
1.053
2.062
1.000
1.011
1.078
3.021
1.026
X : parts per Million : 1H

$^{13}C\{^{1}H\}$ NMR (100 MHz, $CDCl_3$)

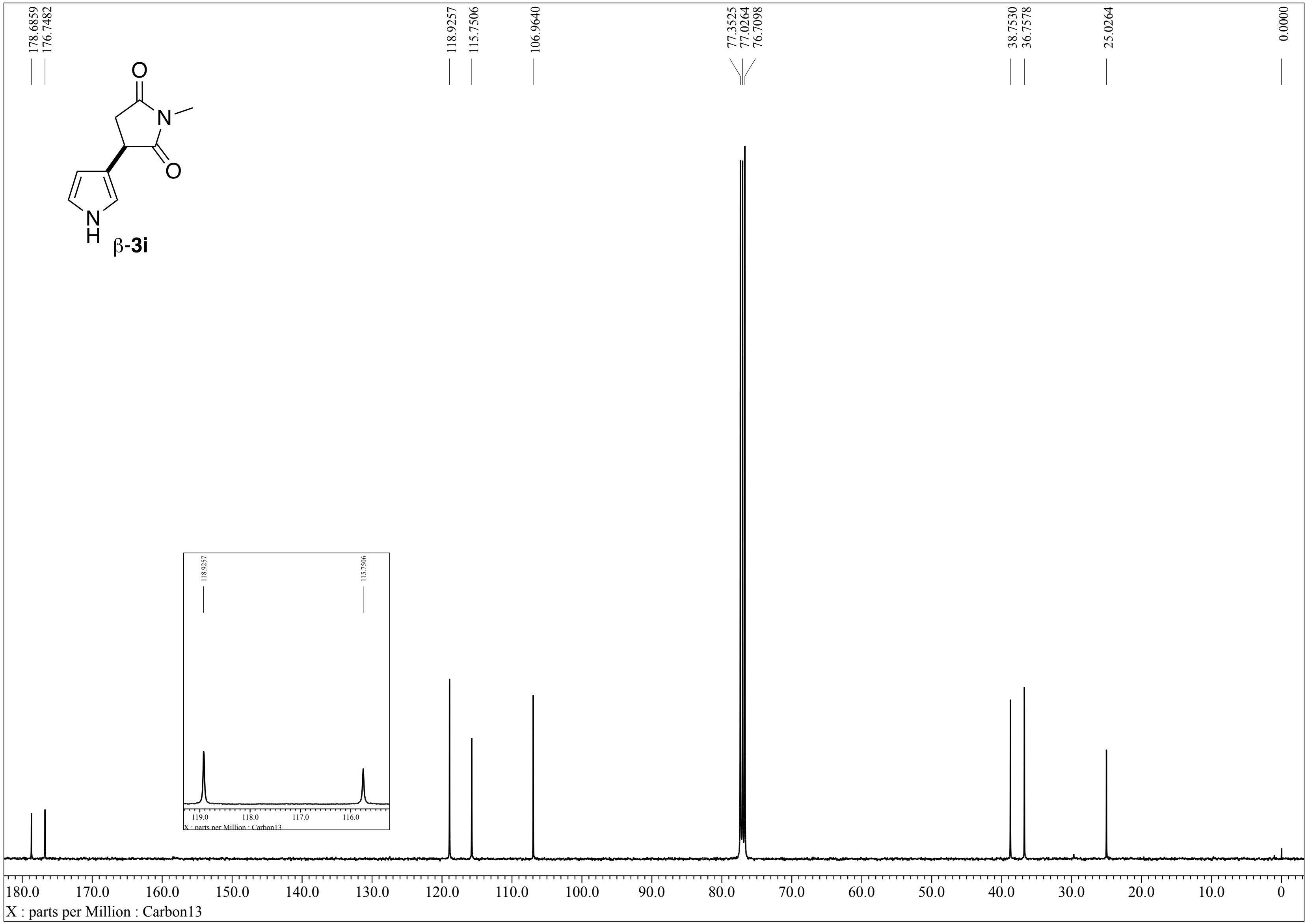

^1H NMR (500 MHz, CD_2Cl_2)

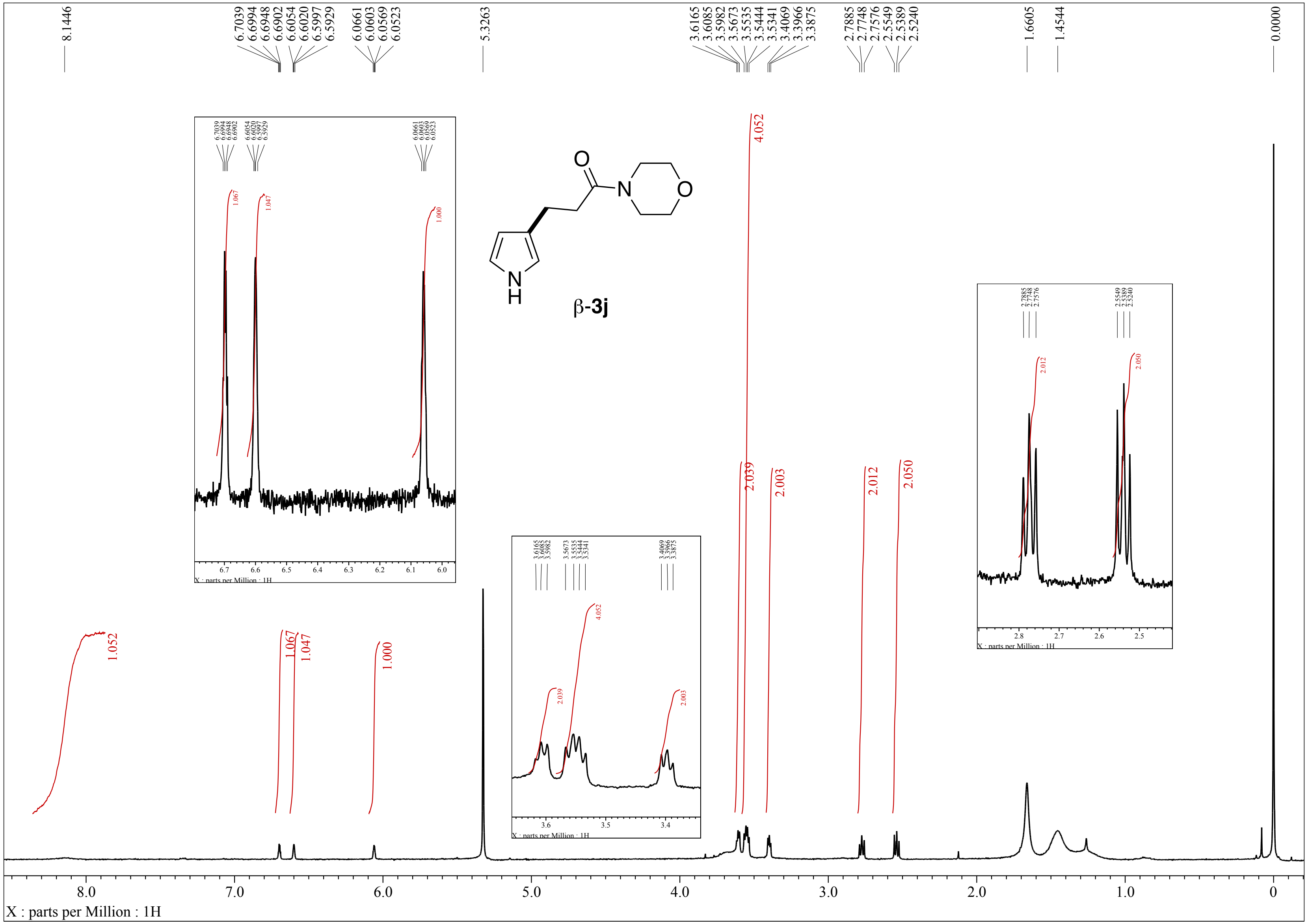

$^{13}C\{^1H\}$ NMR (100 MHz, CD_2Cl_2)

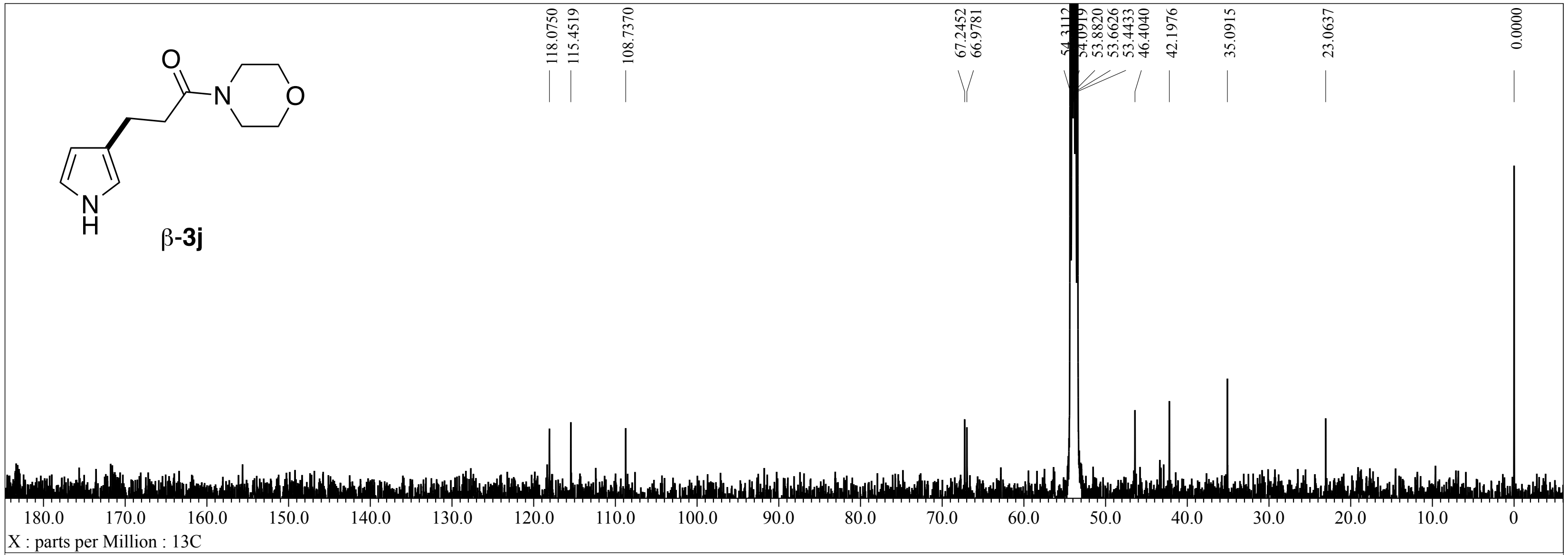

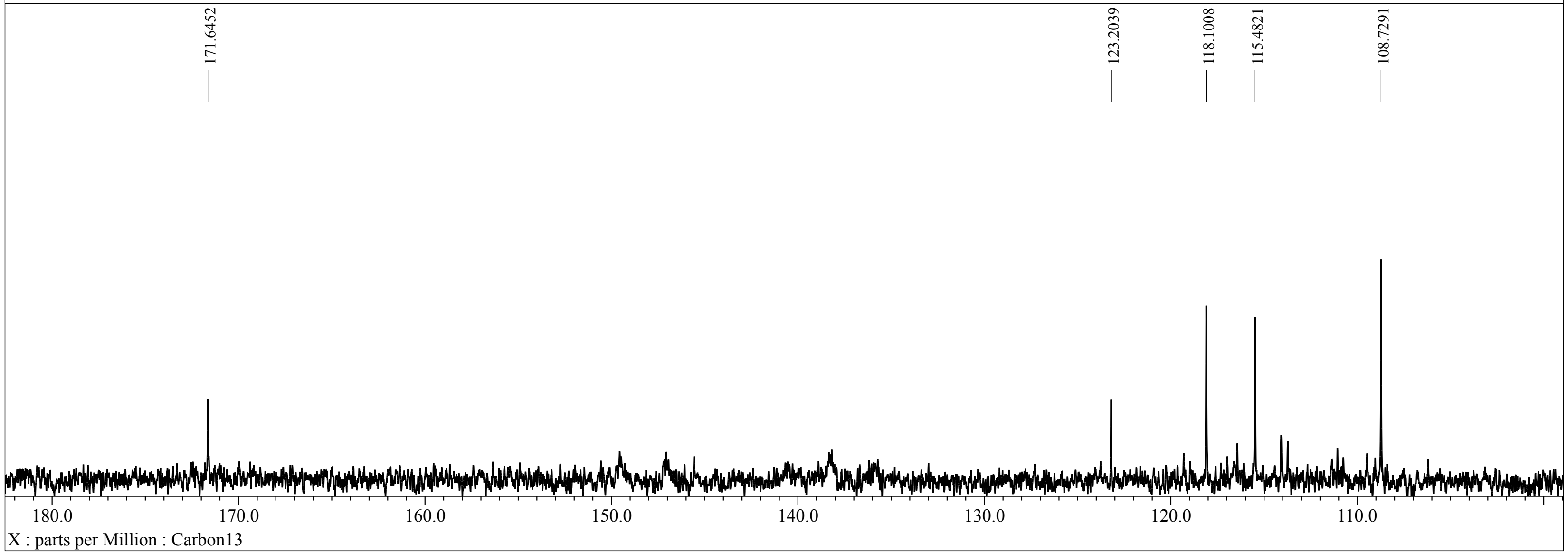

^{1}H NMR (400 MHz, $CDCl_3$)

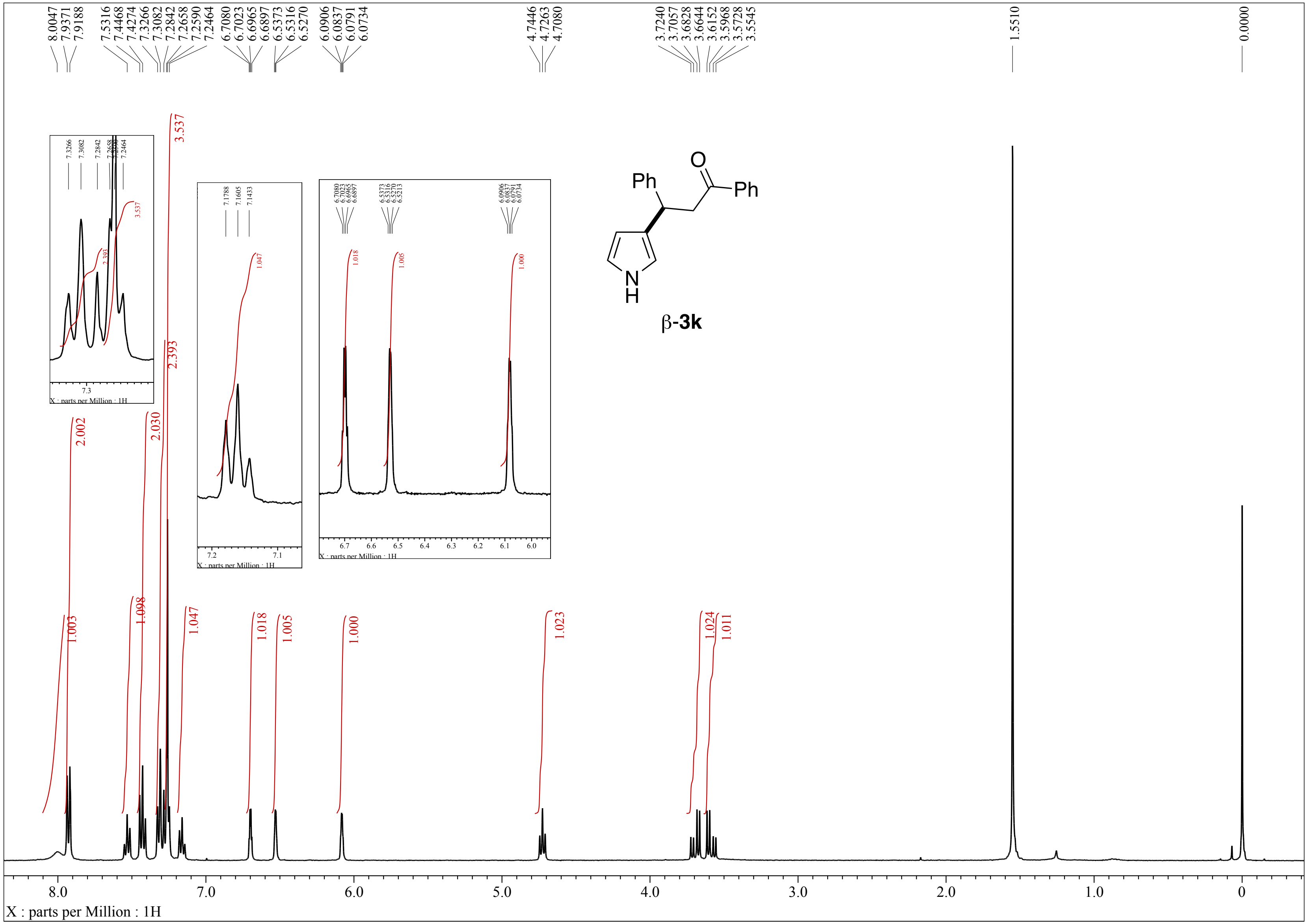

$^{13}C\{^{1}H\}$ NMR (100 MHz, $CDCl_3$)

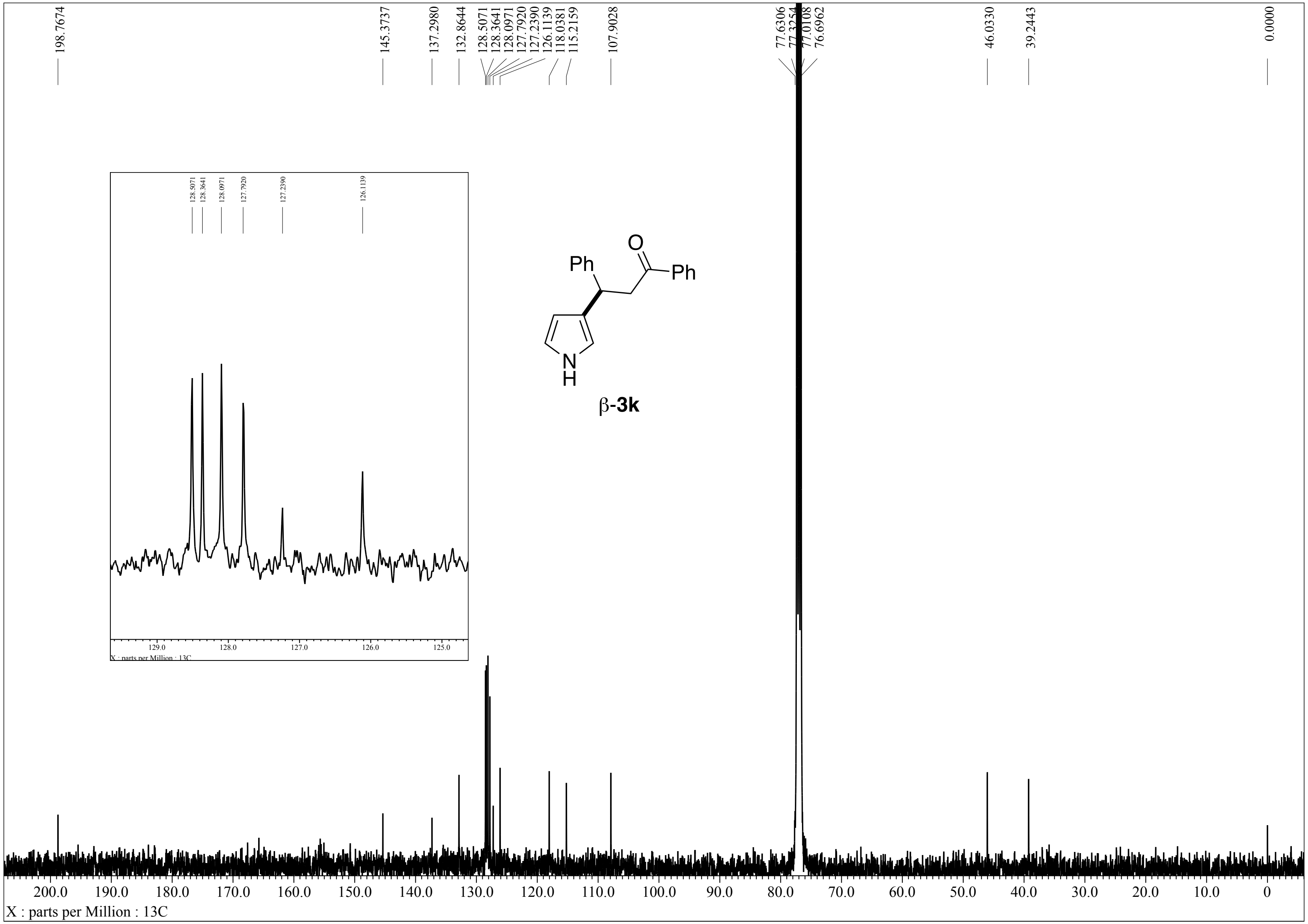

^{1}H NMR (400 MHz, $CDCl_3$)

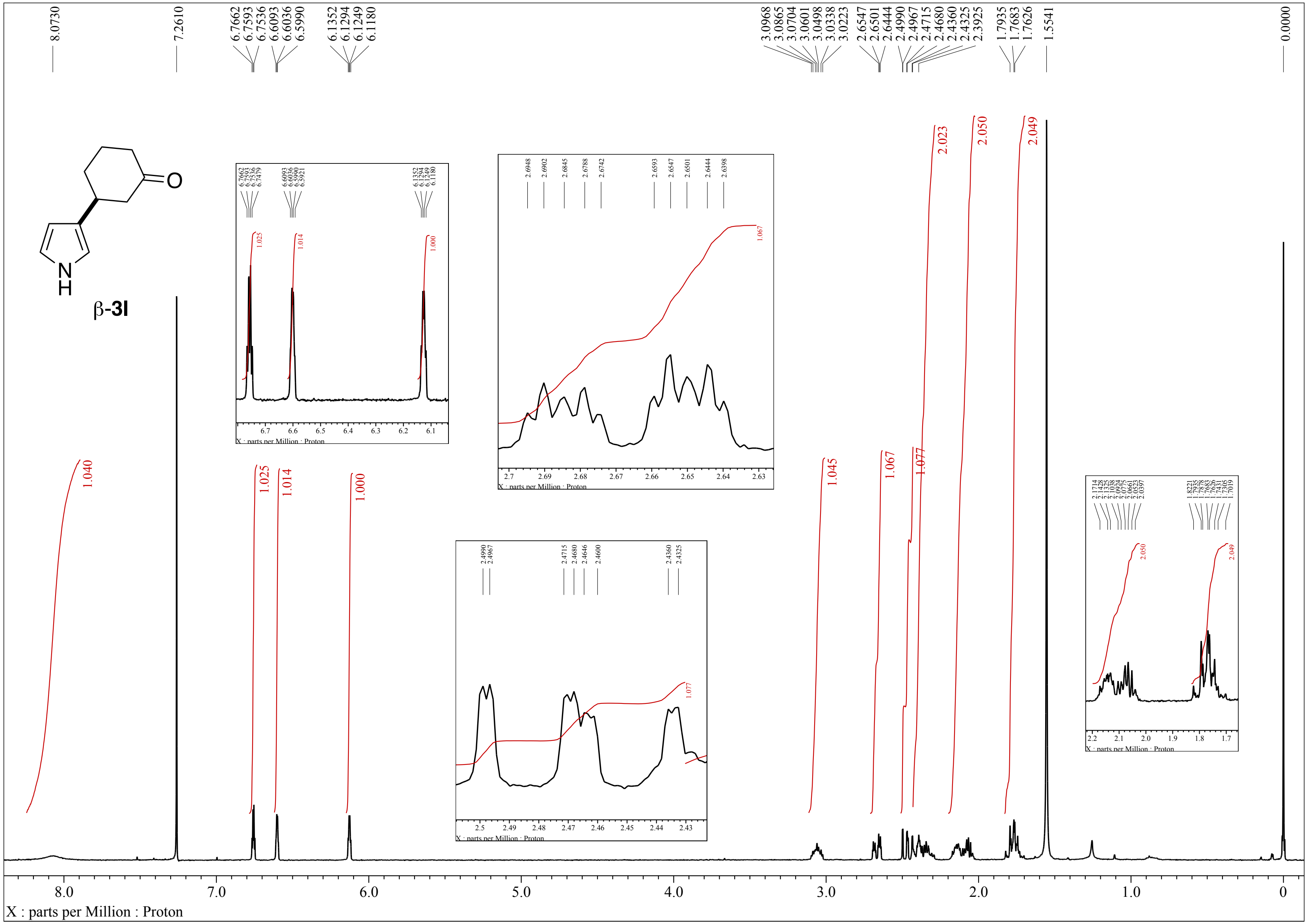

$^{13}C\{^{1}H\}$ NMR (100 MHz, $CDCl_3$)

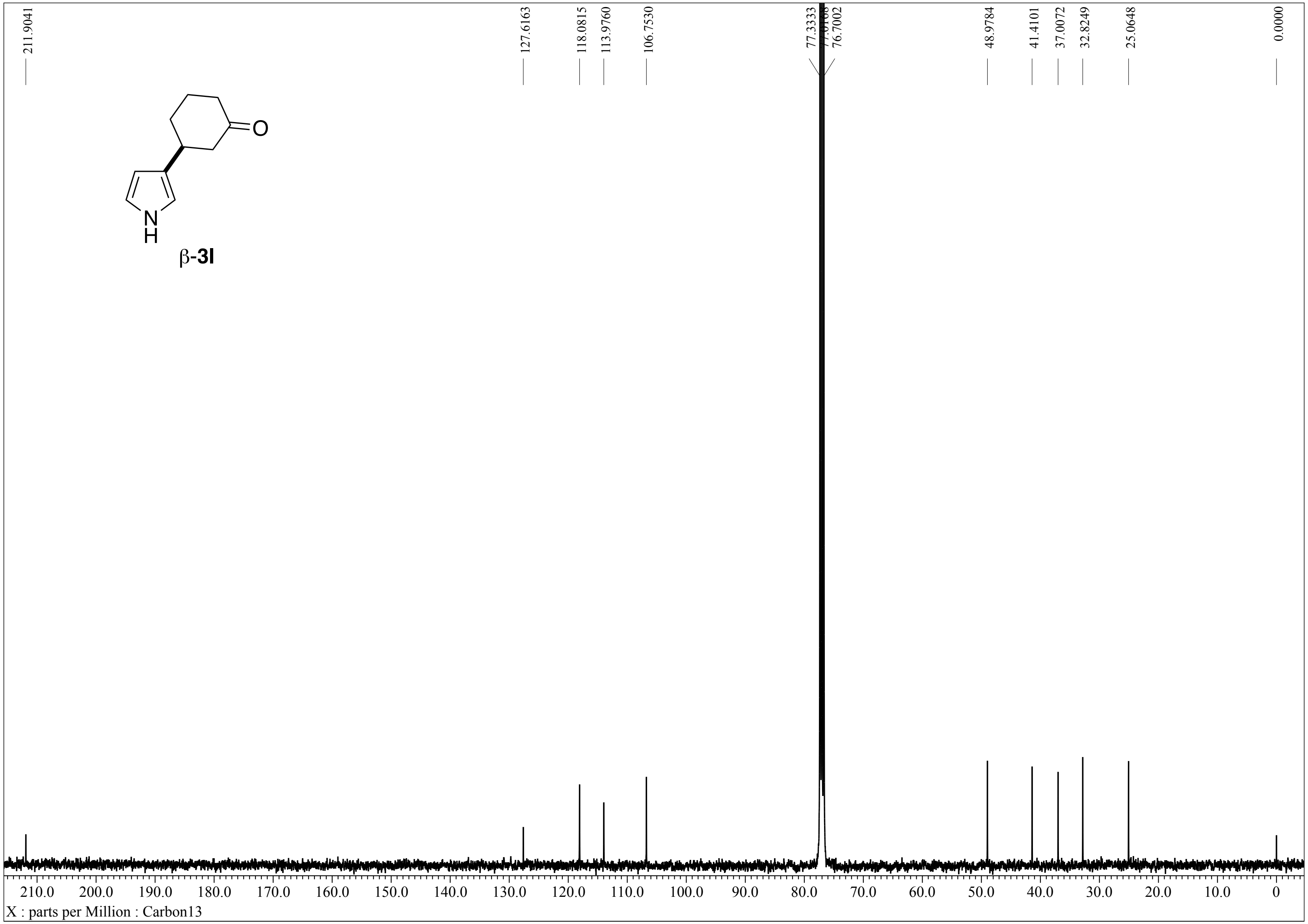

^{1}H NMR (500 MHz, $CDCl_3$)

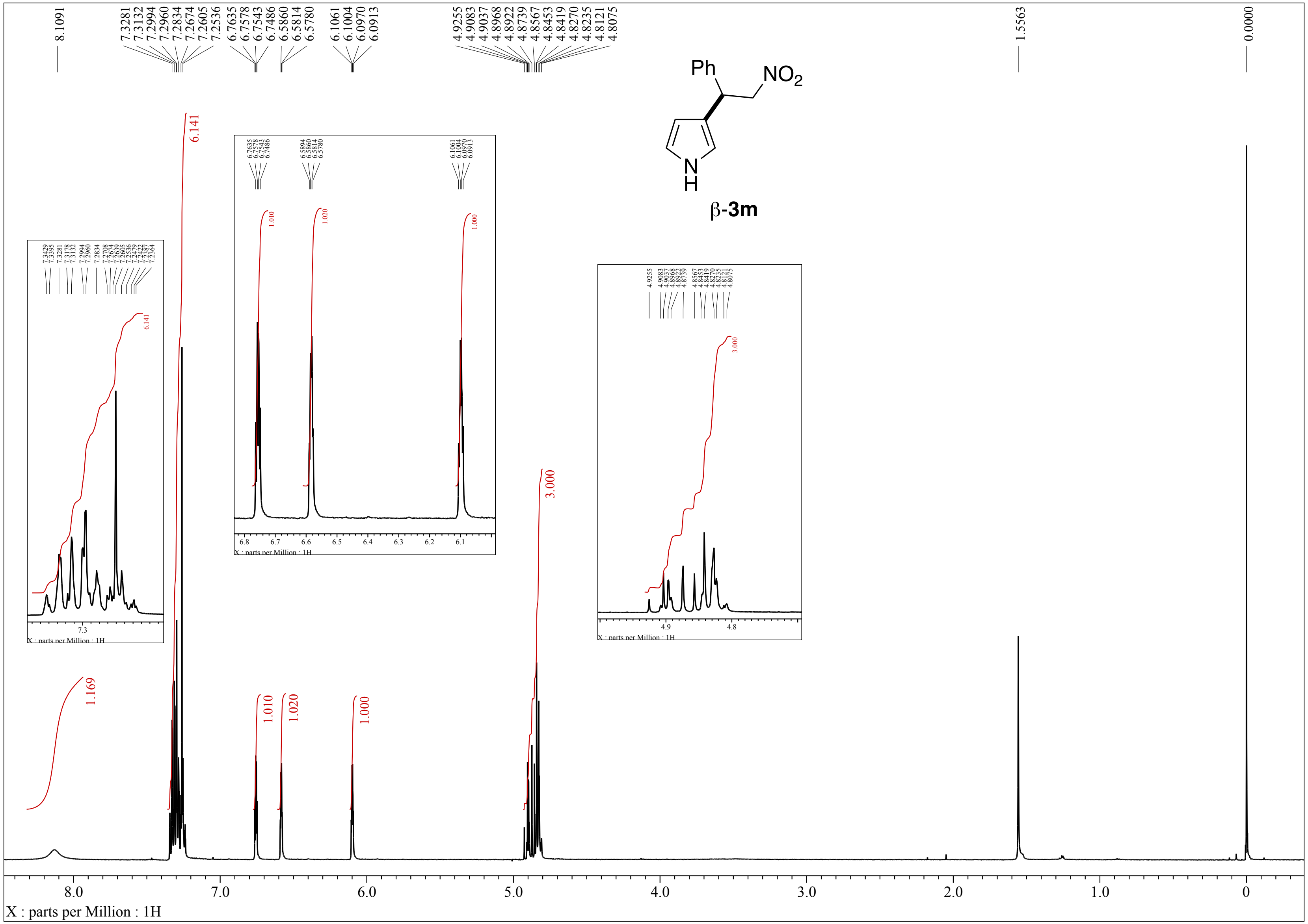

$^{13}C\{^{1}H\}$ NMR (125 MHz, $CDCl_3$)

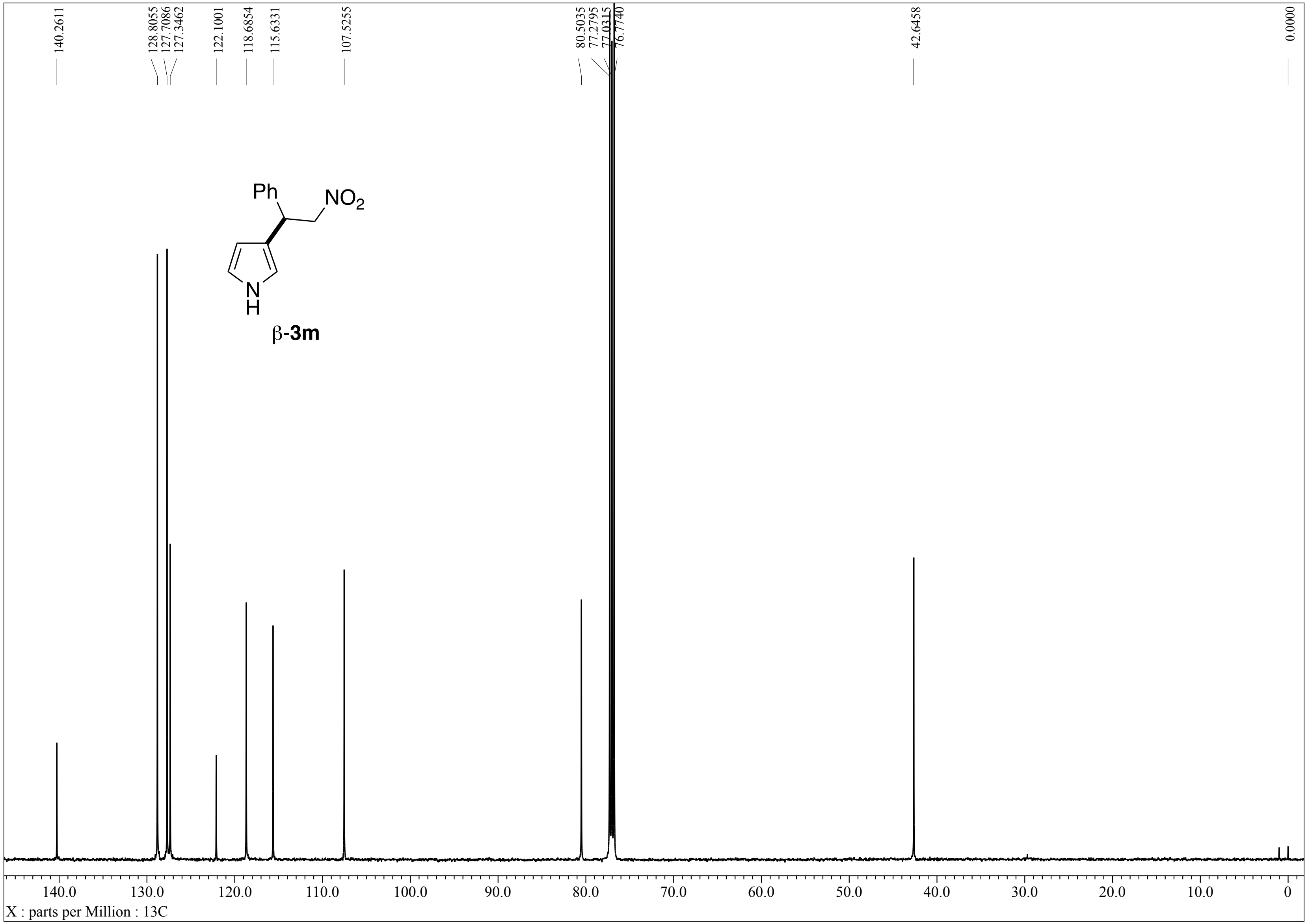

^{1}H NMR (500 MHz, $CDCl_3$)

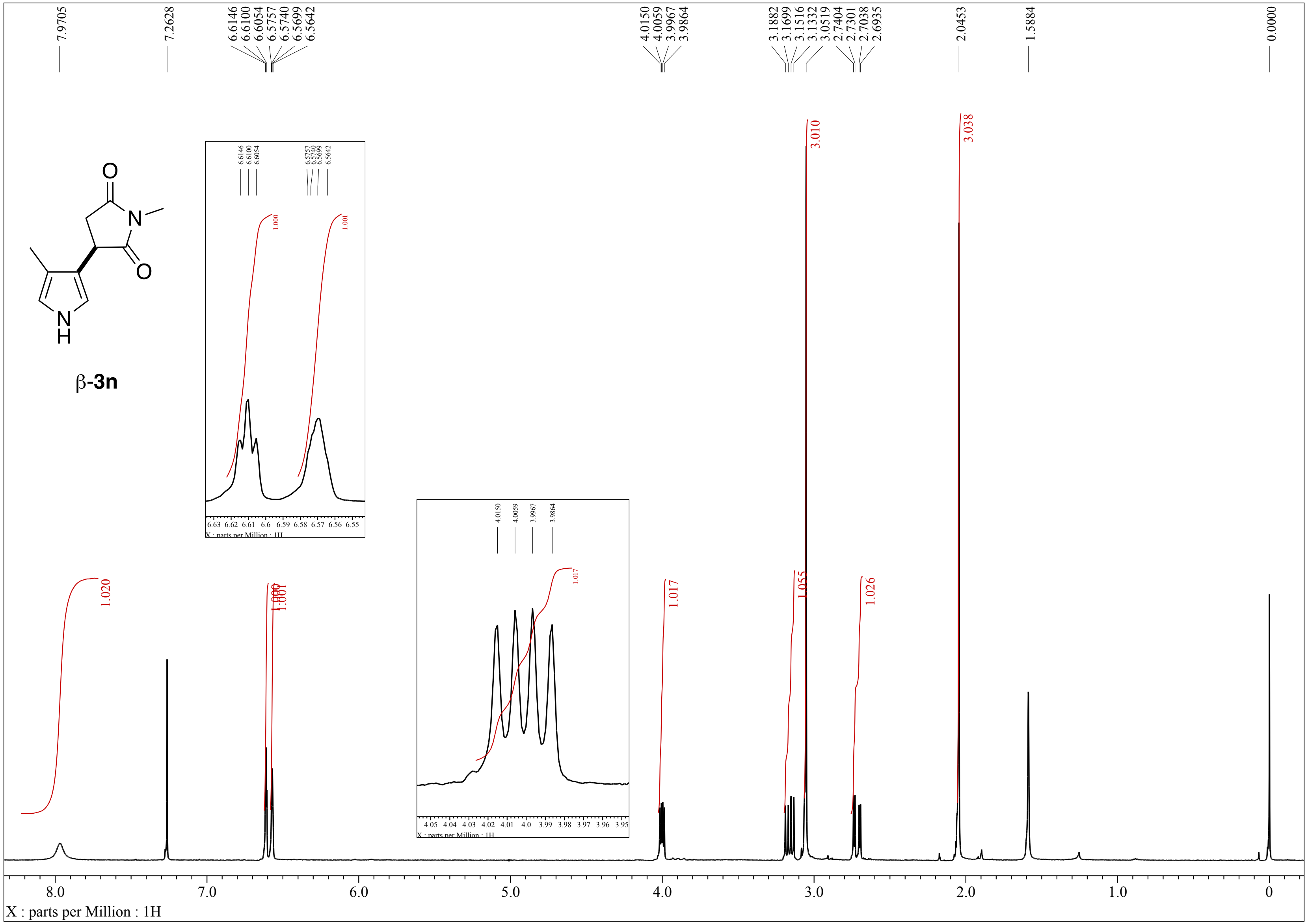

$^{13}C\{^1H\}$ NMR (100 MHz, $CDCl_3$)

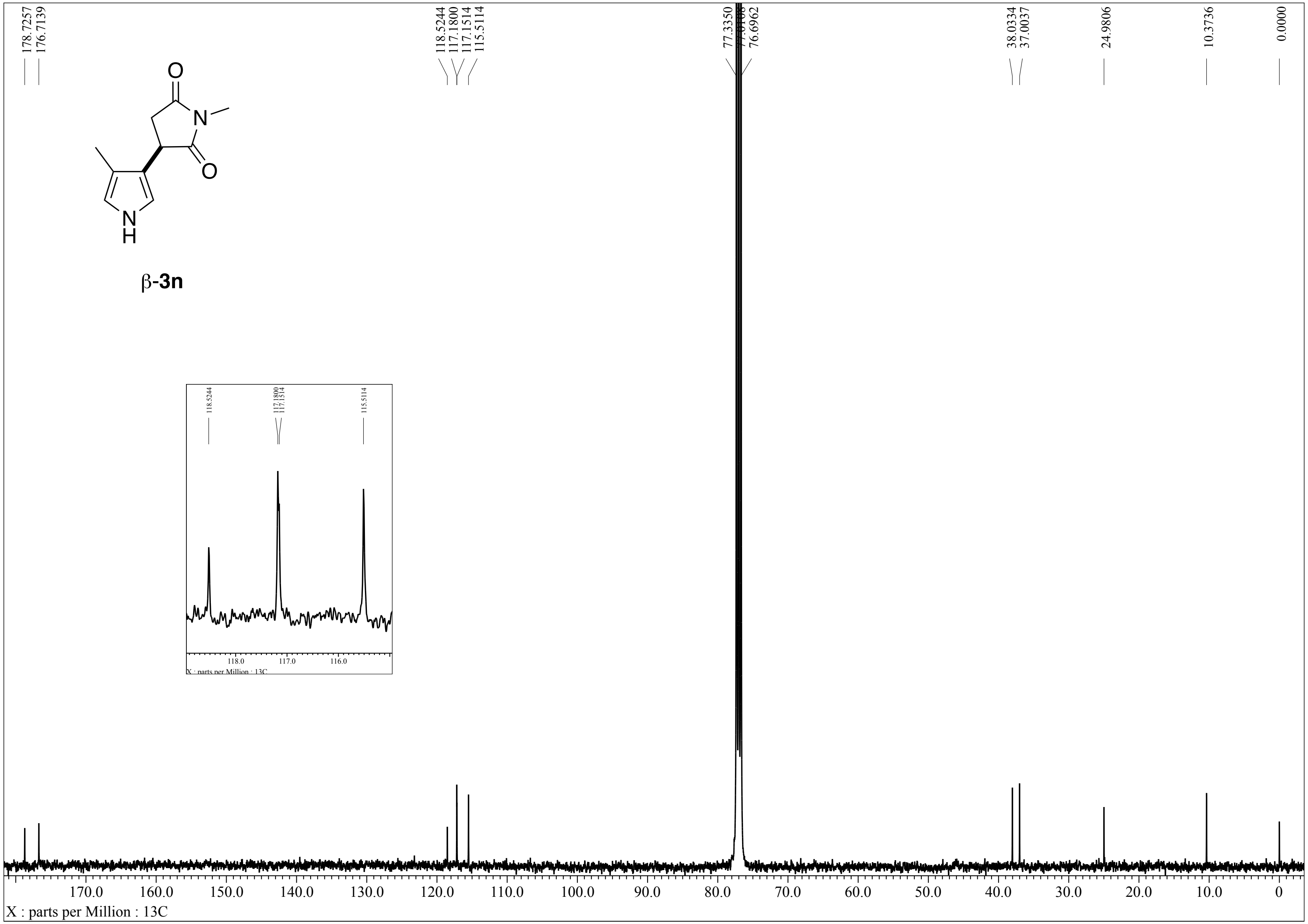

^{1}H NMR (500 MHz, $CDCl_3$)

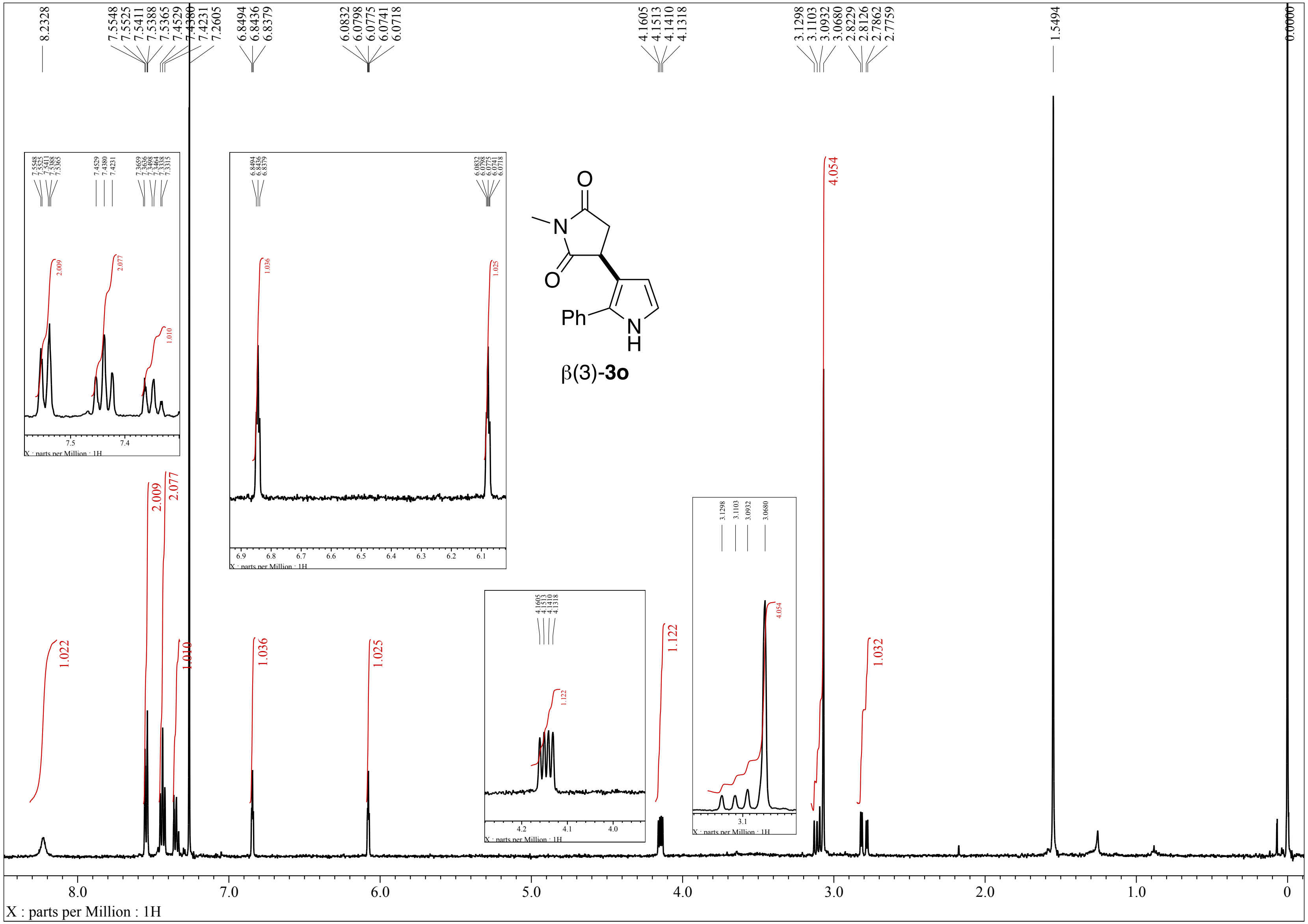

$^{13}C\{^1H\}$ NMR (100 MHz, $CDCl_3$)

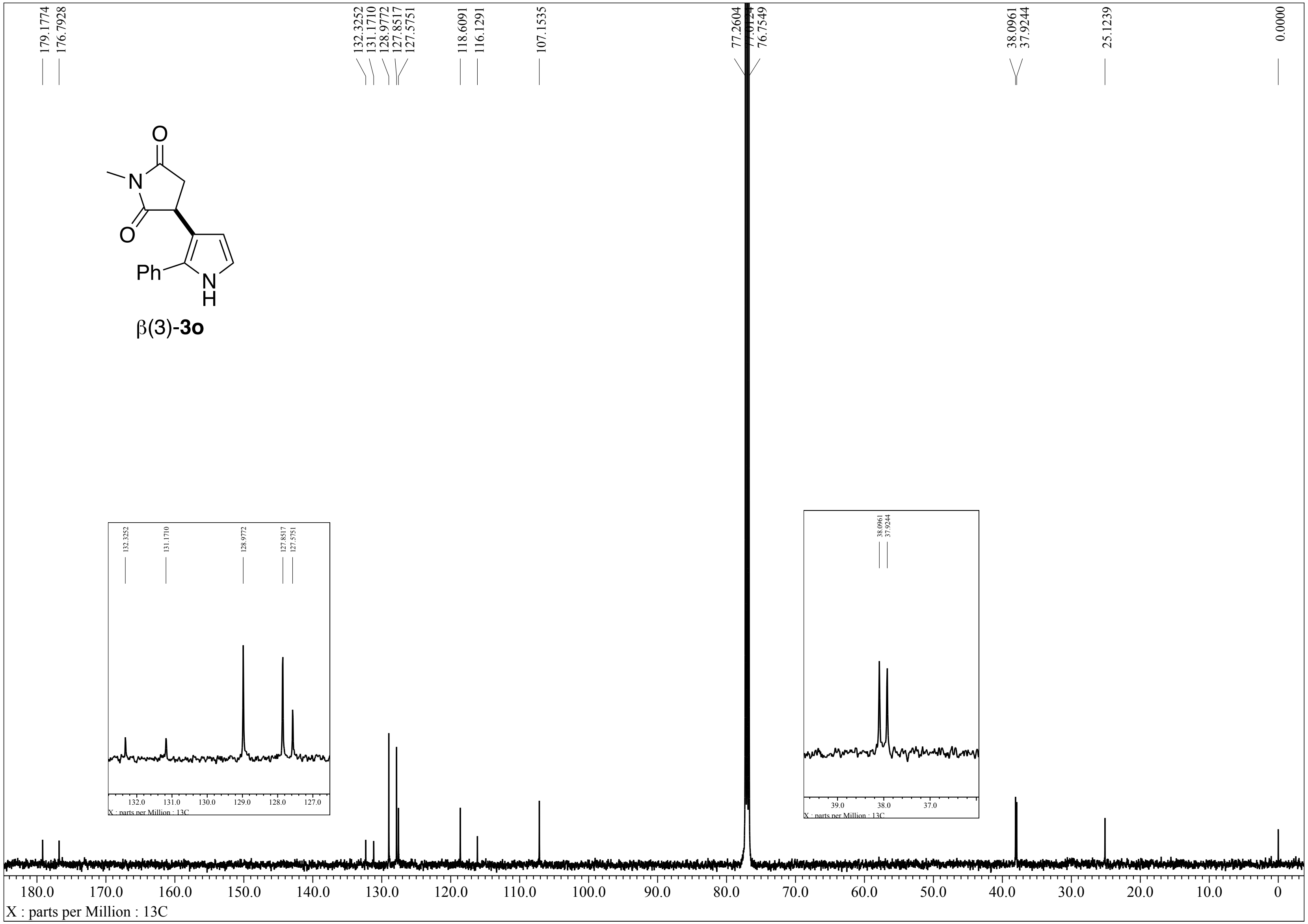

^{1}H NMR (500 MHz, $CDCl_3$)

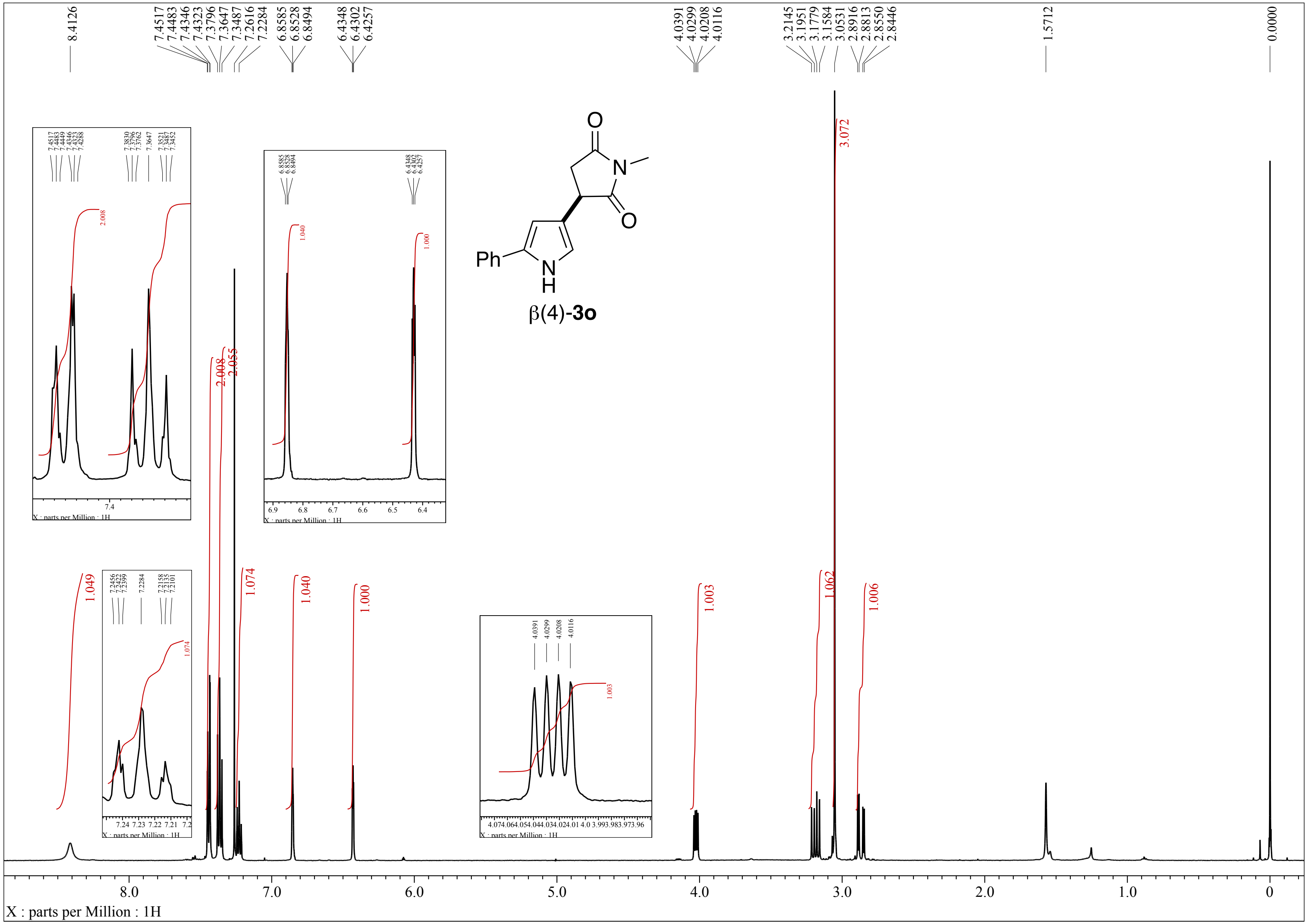

$^{13}C\{^{1}H\}$ NMR (100 MHz, $CDCl_3$)

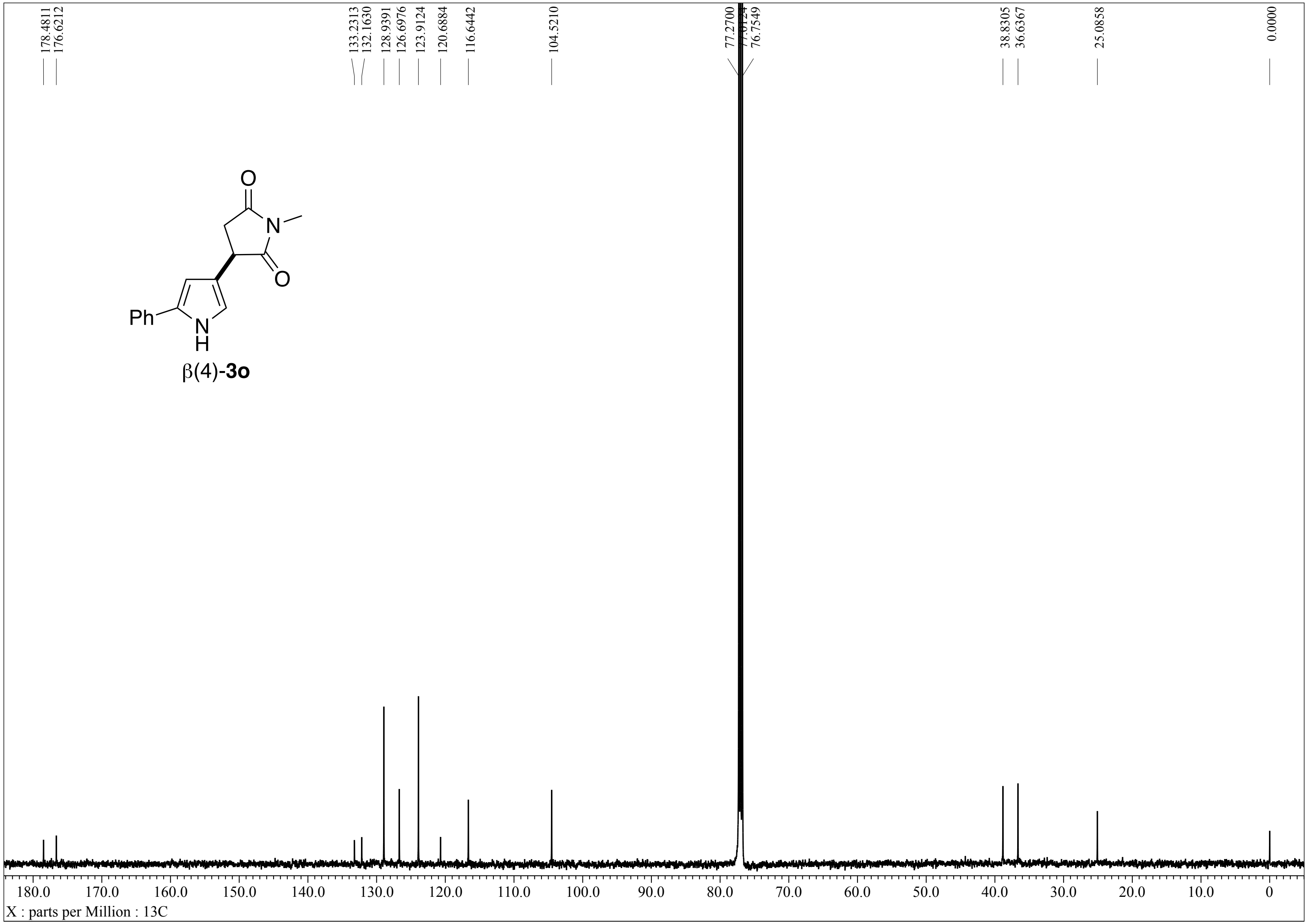

^{1}H NMR (500 MHz, $CDCl_3$)

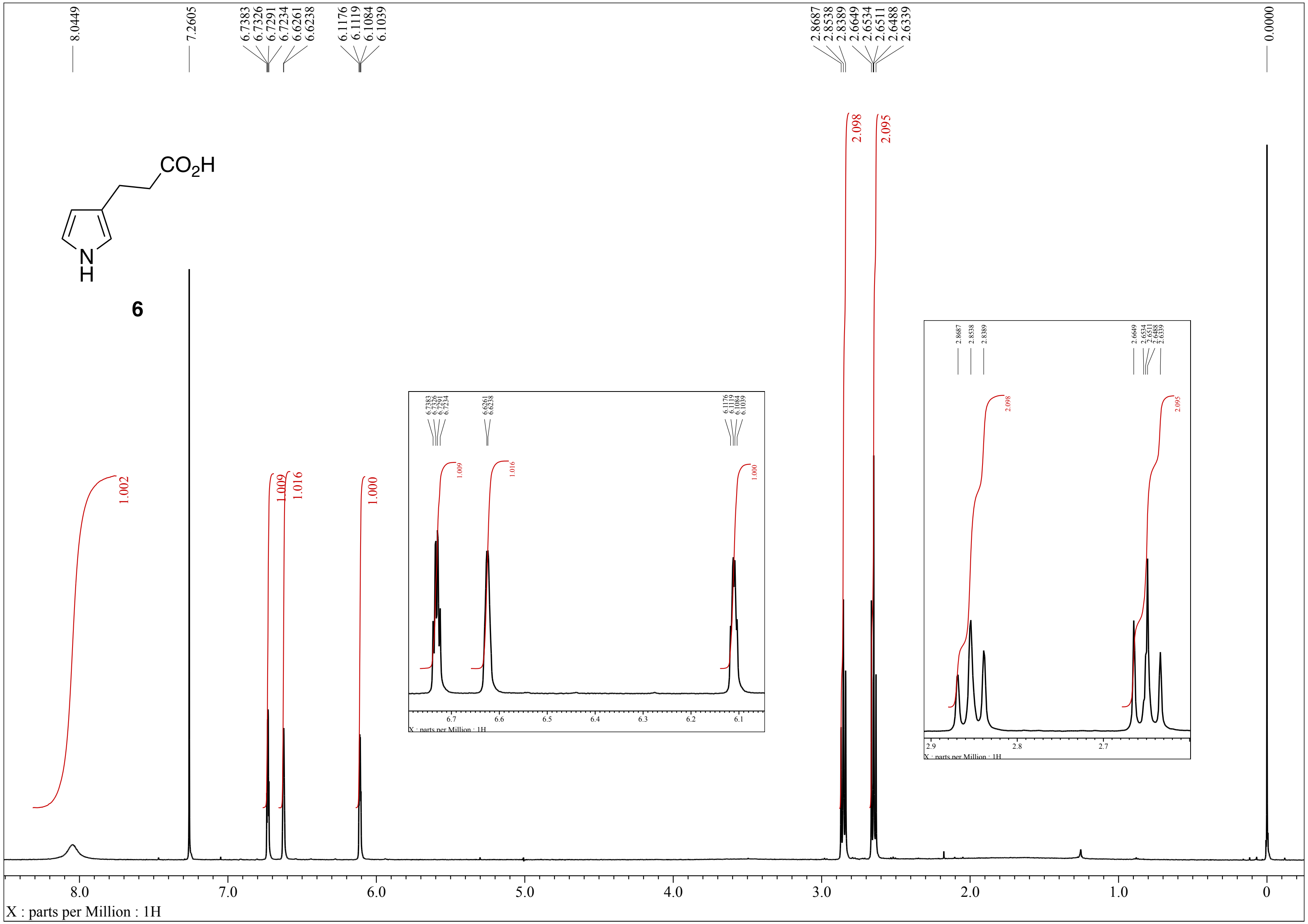

$^{13}C\{^{1}H\}$ NMR (125 MHz, $CDCl_3$)

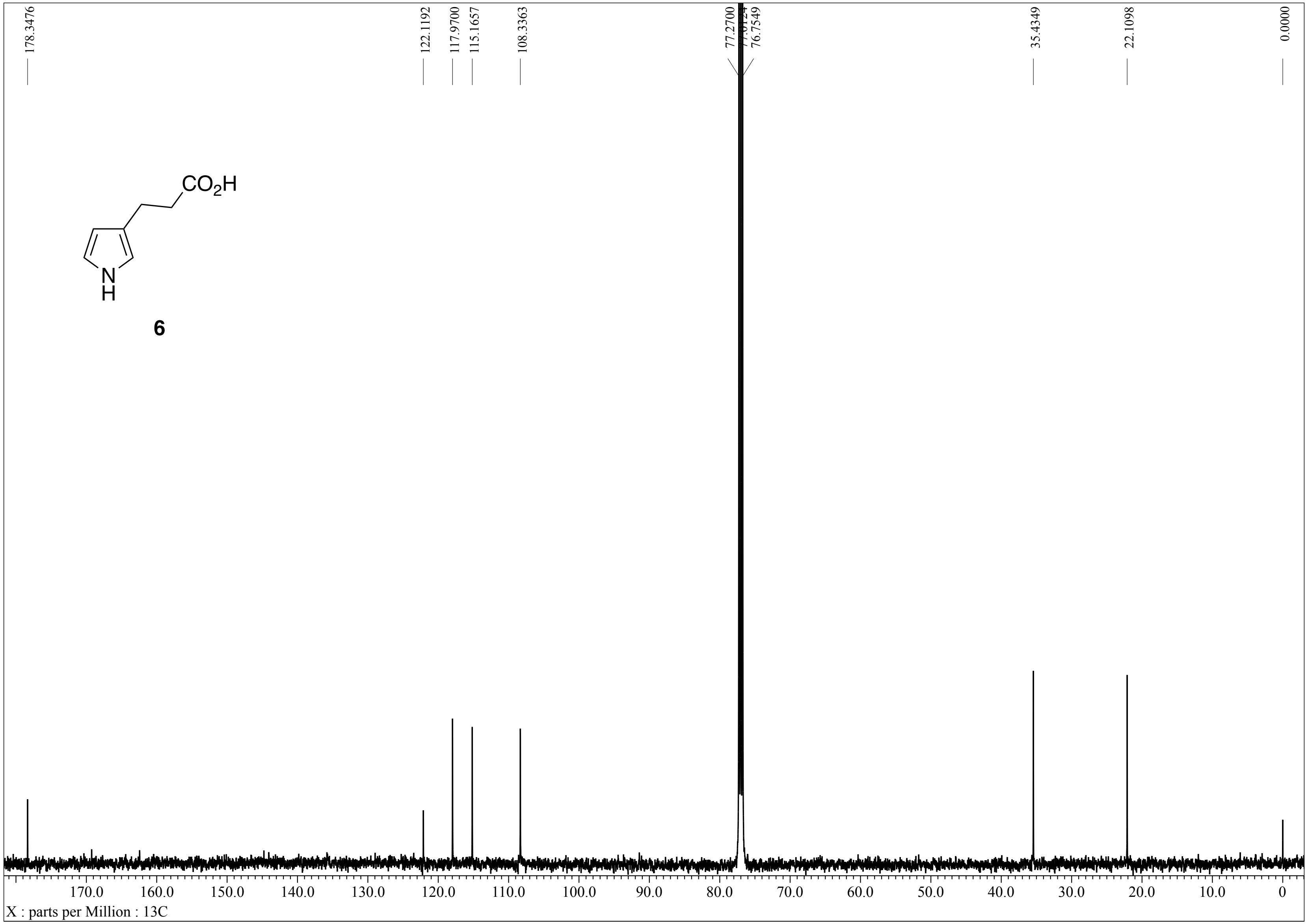

^{1}H NMR (400 MHz, $CDCl_3$)

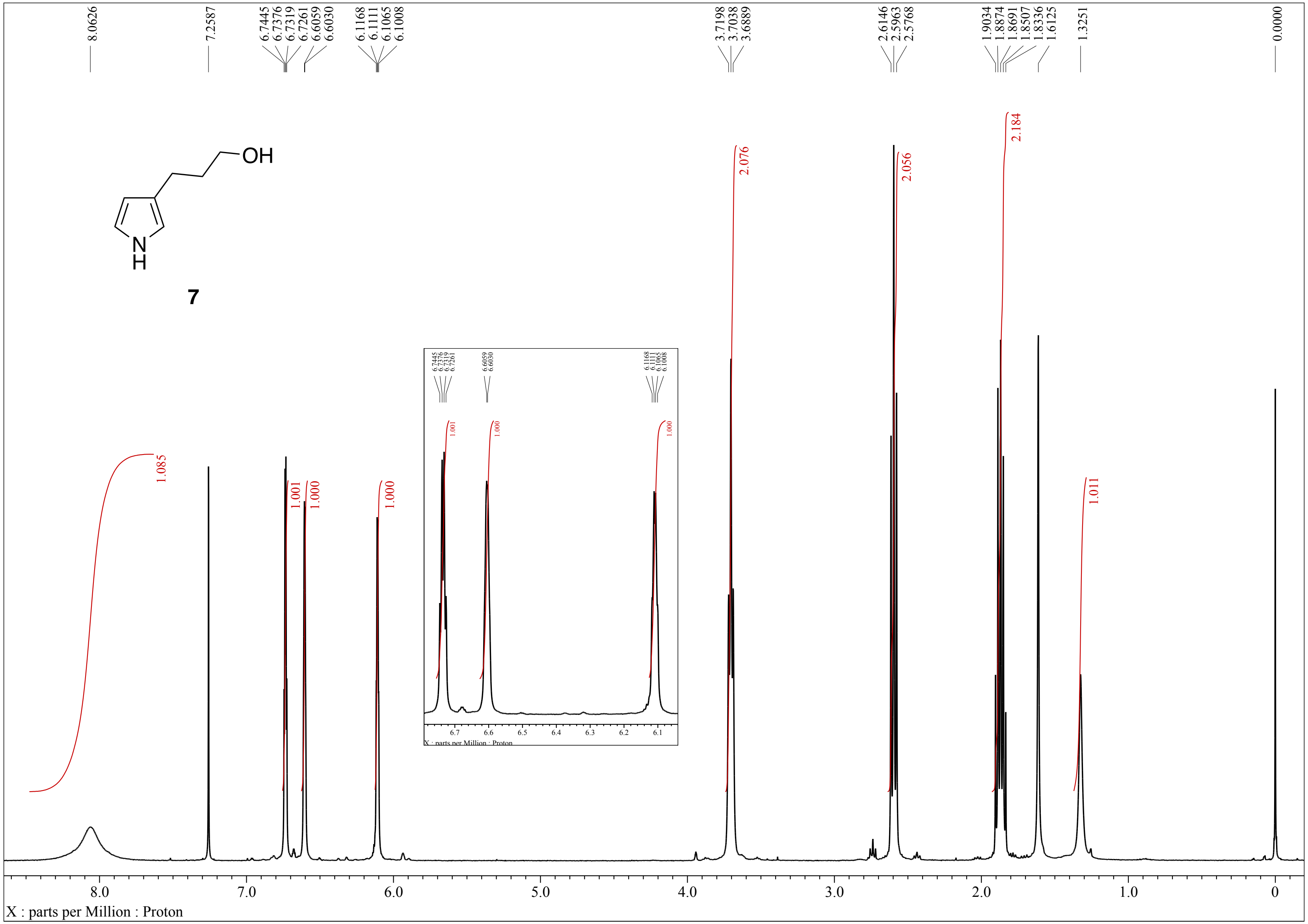

$^{13}C\{^{1}H\}$ NMR (125 MHz, $CDCl_3$)

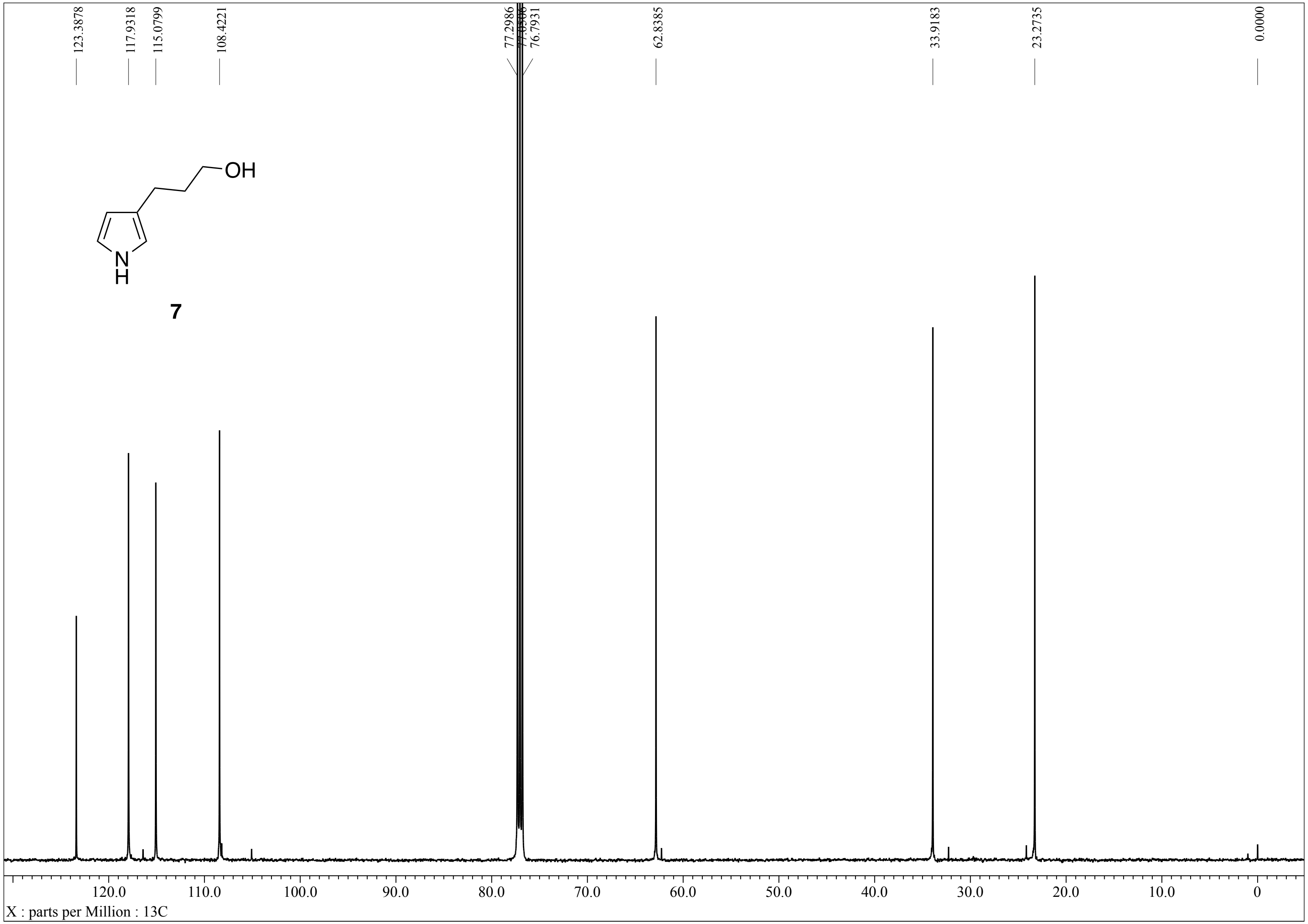

^{1}H NMR (400 MHz, $CDCl_3$)

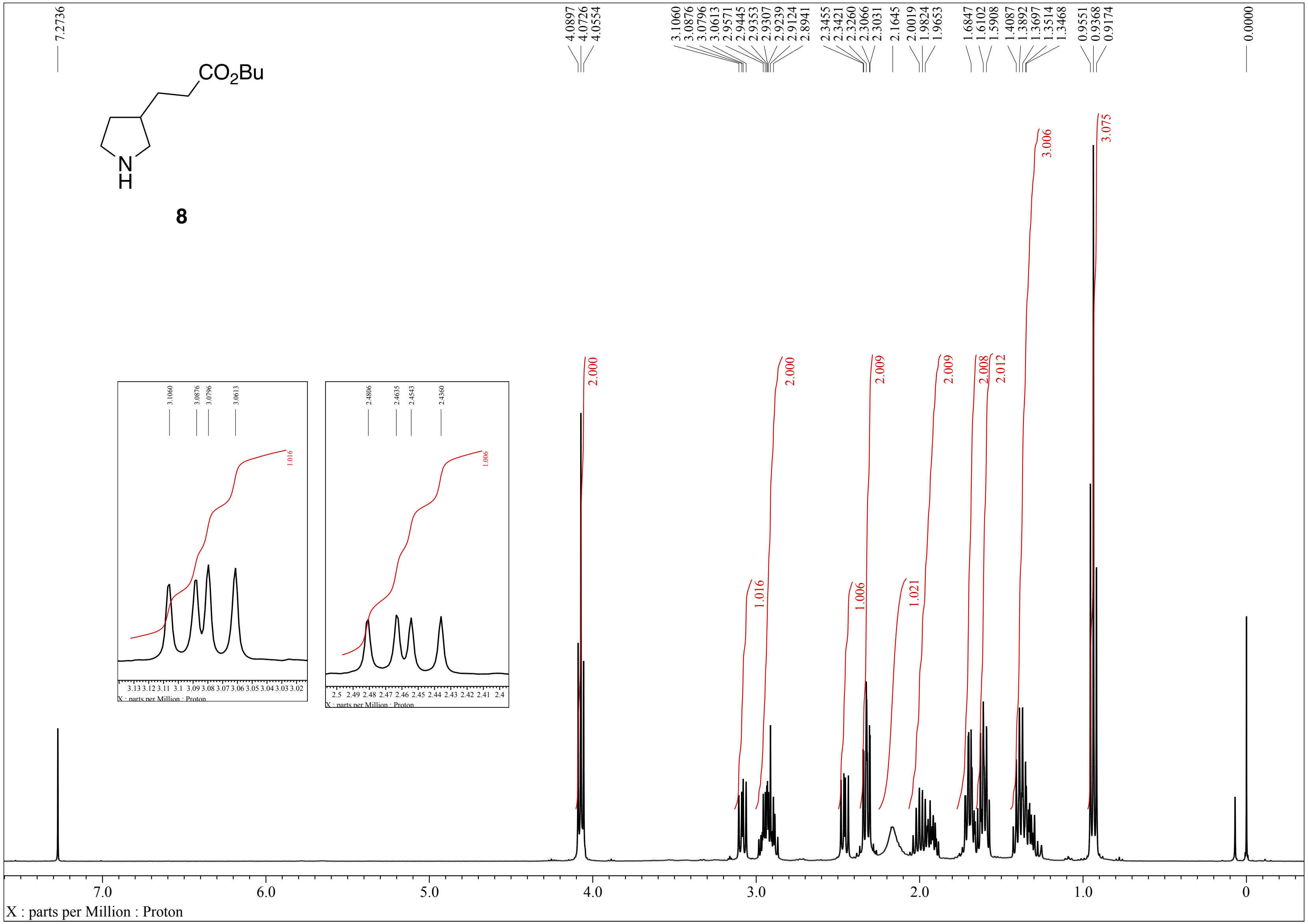

$^{13}C\{^{1}H\}$ NMR (100 MHz, $CDCl_3$)

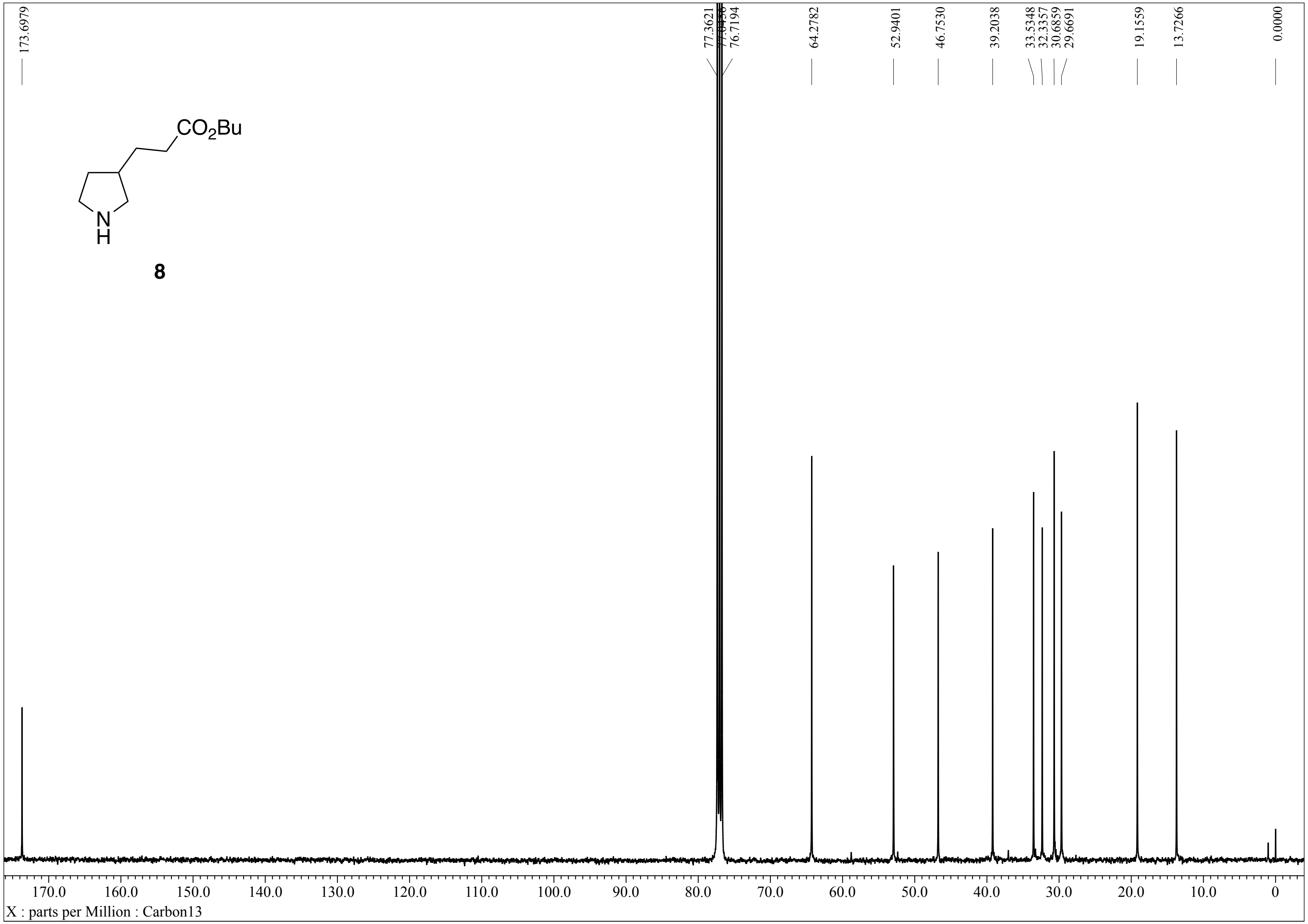

^{1}H NMR (500 MHz, THF-d_8)

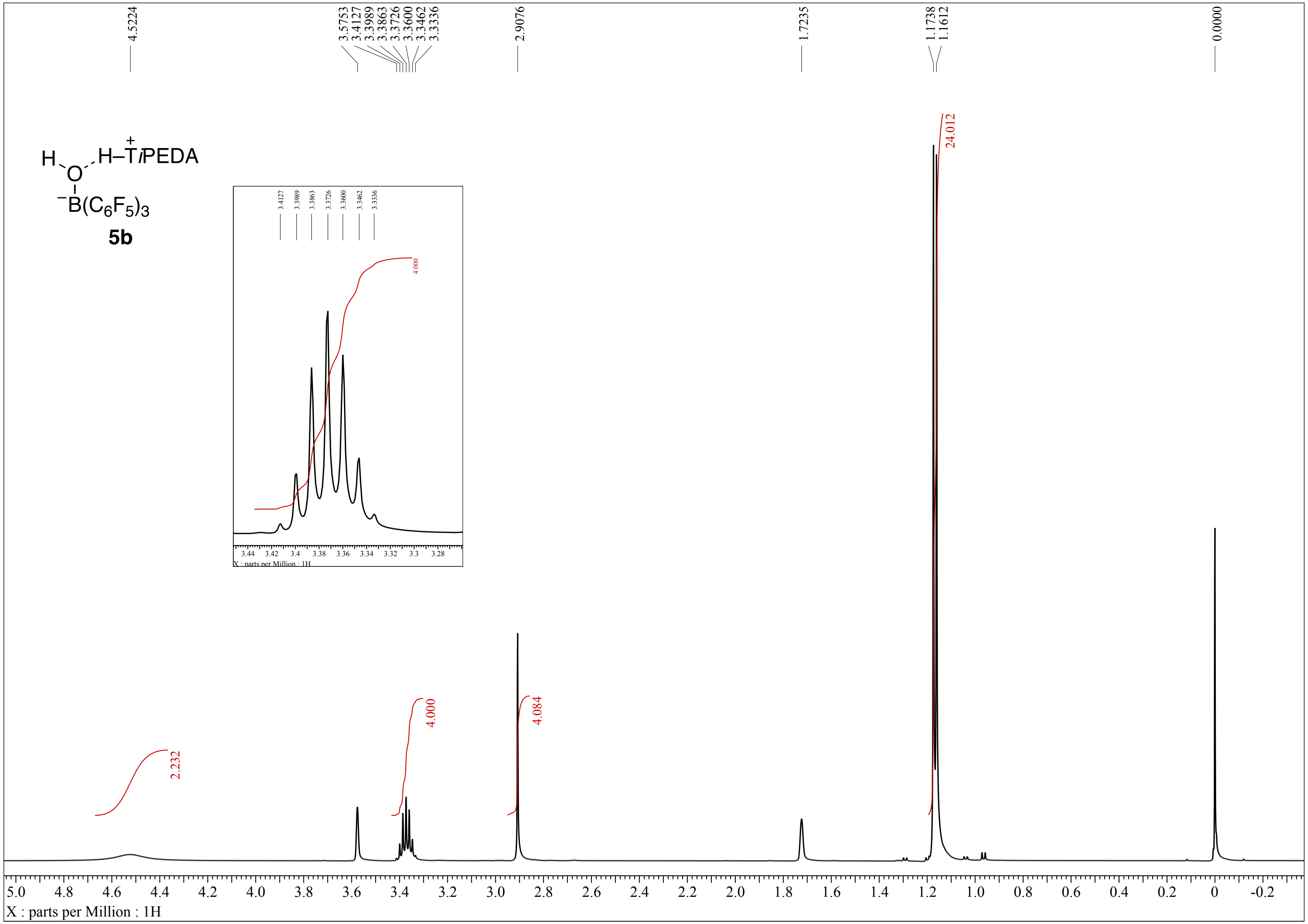

$^{13}C\{^1H\}$ NMR (125 MHz, THF-d_8)

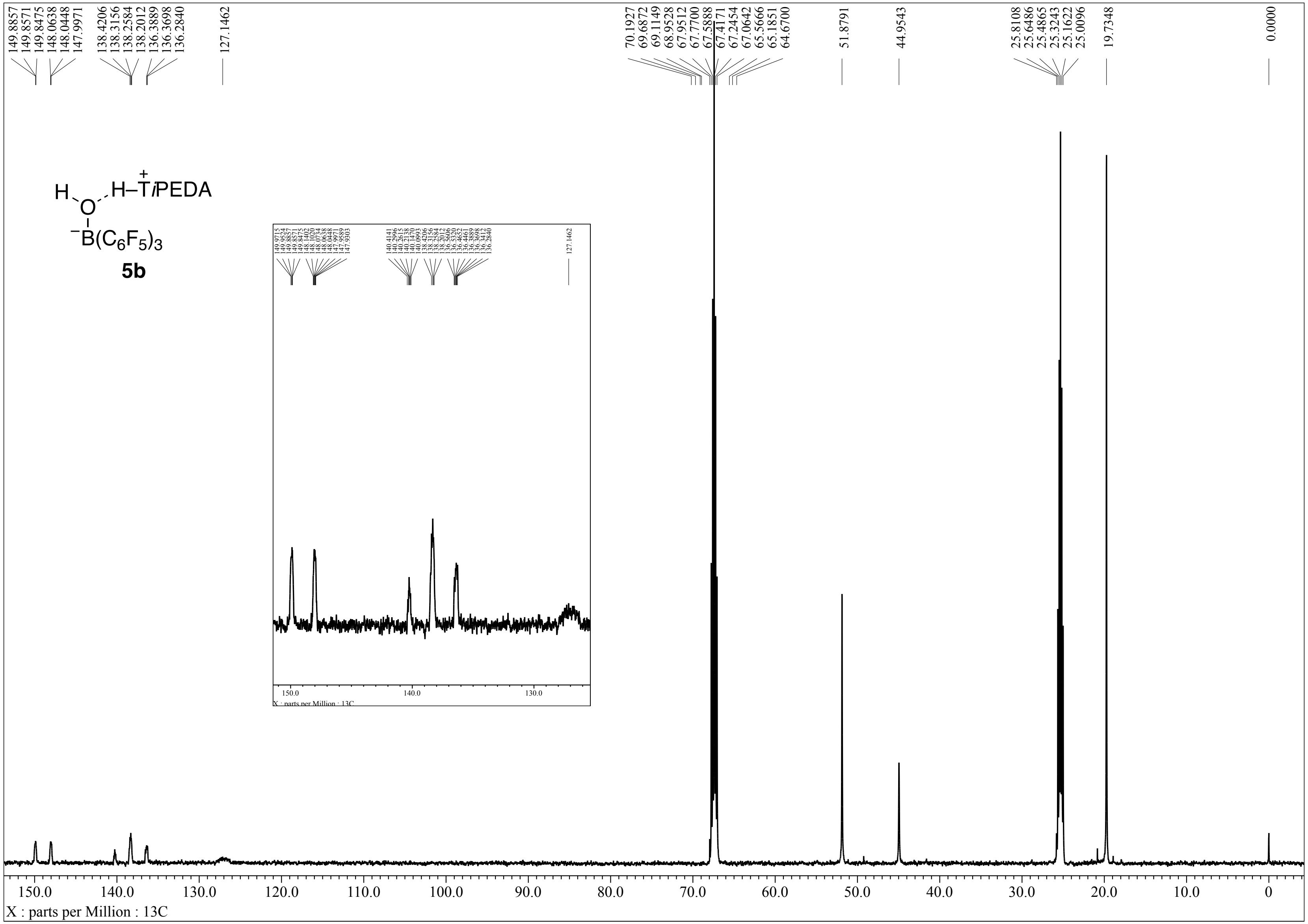

^{19}F NMR (471 MHz, THF-d_8)

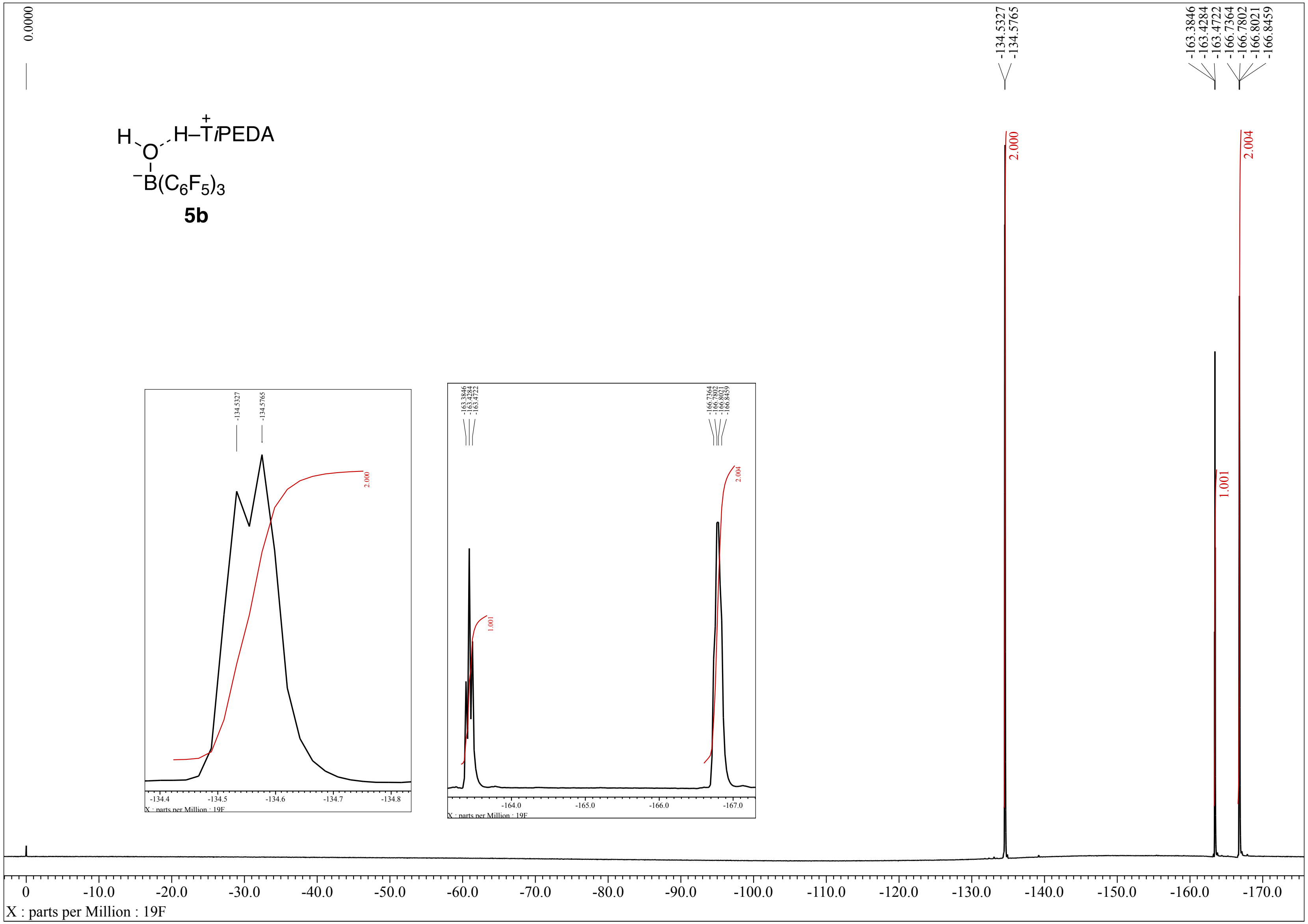

^{11}B NMR (128 MHz, THF-d_8)

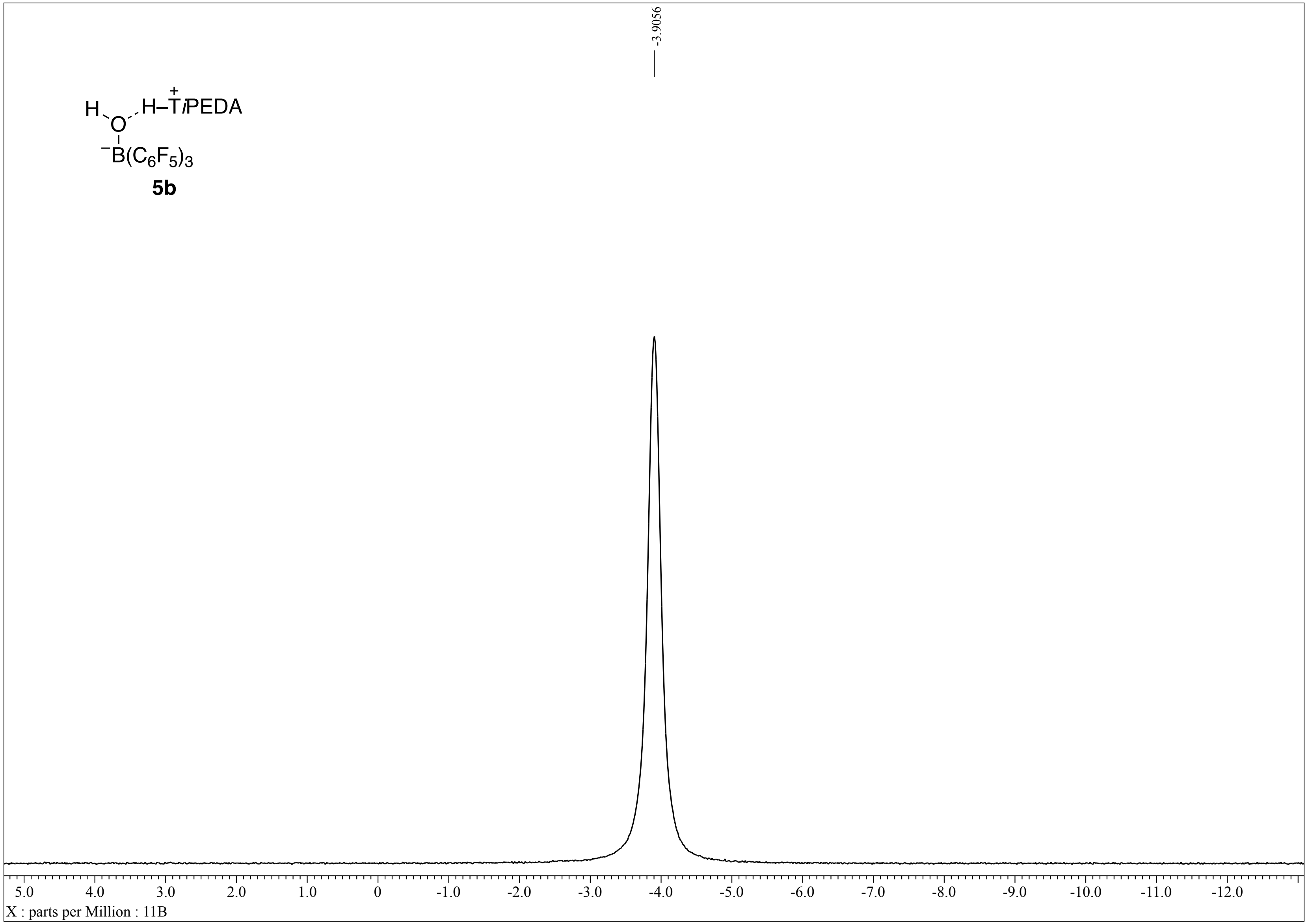

Supplement: Supplementary file 1 [file ao5c00371_si_001.pdf]
